# A systematic review of the biopsychosocial dimensions affected by chronic pain in children and adolescents: identifying reliable and valid pediatric multidimensional chronic pain assessment tools

**DOI:** 10.1097/PR9.0000000000001099

**Published:** 2023-11-28

**Authors:** Megan J. Greenough, Lindsay Jibb, Krystina B. Lewis, Tracey Bucknall, Christine Lamontagne, Melissa Demery Varin, Ashley Sokalski, Janet Elaine Squires

**Affiliations:** a School of Nursing, University of Ottawa, Ottawa, ON, Canada; b Chronic Pain Services at The Children's Hospital of Eastern Ontario, Ottawa, ON, Canada; c Bloomberg Faculty of Nursing, University of Toronto, Toronto, ON, Canada; d Pediatric Nursing Research, SickKids Hospital, Toronto, ON, Canada; e School of Nursing, Deakin University, Burwood Victoria, Melbourne, Australia; f Centre for Quality and Patient Safety Research, Institute for Health Transformation, Geelong, Australia; g Department of Anesthesiology and Pain Medicine, University of Ottawa, Ottawa, ON, Canada; h University Research Chair in Health Evidence Implementation & School of Nursing, University of Ottawa, Ottawa, ON, Canada; i The Ottawa Hospital Research Institute, Ottawa, ON, Canada

**Keywords:** Chronic pain, Multidimensional tool, Biopsychosocial, Pediatric, Psychometric, Pain interference, Primary chronic pain

## Abstract

Pediatric chronic pain is a complex experience that is often challenging to describe and measure. Multidimensional tools that evaluate the biopsychosocial impact of chronic pain in pediatric patients can help clinicians to prioritize and tailor interdisciplinary pain care; yet, the psychometric value and clinical utility of such tools has not yet been systematically studied in the literature. The purpose of this review was to identify multidimensional biopsychosocial tools used in pediatric chronic pain, synthesize their reliability and validity evidence, and draw on this evidence to describe the relationships between chronic pain and biopsychosocial domains. The search involved 2 phases to (1) identify eligible tools and (2) conduct a measured forward citation search of tool development articles. Tool eligibility was guided by the *Multidimensional Biobehavioral Model of Pediatric Pain* and study eligibility was focused on primary chronic pain diagnoses unrelated to disease. Data extraction was focused on reliability and validity evidence of eligible tools, guided by the *Standards for Educational and Psychological Testing*. Results yielded 6 tools that included 64 eligible studies, highlighting 84 significant relationships between pain and functional interference across 11 biopsychosocial variables. All tools were shown to have good internal consistency and evidence of validity, primarily through relationships to other variables. Of the 6 tools, the most brief and easy to use were the most under studied. Further psychometric research is warranted for these tools to investigate their clinical utility and psychometric properties in guiding and prioritizing pain care for children and adolescents.

## Abstract 2

Supplemental Digital Content is Available in the Text.

A synthesis of reliable and valid multidimensional tools for children and adolescents with chronic pain that robustly emphasize the relationships between pain and biopsychosocial domains.

## 1. Introduction

Chronic pain in pediatric patients is a multidimensional experience, involving interplay between nociceptive processing, affect, sociocultural context, and behavioral and cognitive mechanisms.^28,84^ Consequently, a variety of biopsychosocial variables including depression, anxiety, low self-esteem, sleep disturbances, fatigue, and decreased physical functioning interact to affect the functioning and health of children and adolescents living with chronic pain.^23,31,38,42,47,75^ Because the pediatric chronic pain population often receive a variety of ineffective treatments by nonspecialized providers,^84^ it is probable that treatment failure is related to limited tailoring of biopsychosocial needs. For these reasons, multimodal biopsychosocial approaches are considered gold standard for chronic pain treatment, thus interdisciplinary chronic pain programs are an ideal setting for this patient population.^65^ Because the spectrum of disability between pediatric patients with chronic pain widely varies,^78^ accurate measurement of the biopsychosocial impact of pain may help clinicians with referral processes, prioritization of care, and ongoing assessment of patient response to treatment within such chronic pain programs.

Existing systematic reviews have highlighted the psychometric qualities of single-item pain intensity scales,^7,43,66^ observational pain measures for children and adolescents,^76^ and tools that measure parent response to their child's pain.^33^ Reviews have also shown that most adolescent chronic pain assessment tools focus solely on the psychological domain. A comprehensive understanding of multi-item tools that measure the biopsychosocial impact of pain across multiple domains of the pediatric chronic pain experience is lacking. Such a review is needed to illuminate the ways in which biopsychosocial variables are interpreted and weighed in predicting patient complexity in the pediatric population with chronic pain, which may help to inform prioritization of care into and within interdisciplinary pediatric chronic pain programs.

The *Multidimensional Biobehavioral Model of Pediatric Pain*^73^ is a framework to support the consideration of chronic pain as a biopsychosocial phenomenon. The model was specifically developed to account for the wide variability of pain perception, pain behavior, and functional status.^75^ It has been used to identify the factors associated with pain intensity and functional disability in a variety of pediatric chronic pain disorders.^75^ The model categorizes variables of the pain experience into the following domains: (1) *precipitants*, including pain related diagnosis or disease, injury, stress, and/or pain-producing procedures; (2) *intervening* variables, including biological predispositions, family environment, cognitive appraisal, coping strategies, and perceived social support; (3) *pain perception and behavior*; and (4) *functional status*, including activities of daily living, school attendance, depressive symptoms, anxious symptoms, behavioral problems, and interpersonal relations.

### 1.1. Objectives

The specific objectives of this review were to (1) identify multidimensional biopsychosocial assessment tools used in the pediatric (ie, ages 2–18 years) population with chronic pain; (2) describe the relationships between chronic pain and the biopsychosocial domains (precipitant variables, intervening variables, and functional status variables) measured in each tool, as defined by the *Multidimensional Biobehavioral Model of Pain*; and (3) review the reliability and validity evidence of such tools and their biopsychosocial domains in the pediatric population with chronic pain.

## 2. Methods

### 2.1. Design and reporting

We conducted a systematic review in accordance with the Preferred Reporting Items for Systematic Reviews and Meta-Analyses (PRISMA) 2020 checklist^54^ which is outlined in Supplementary File 1 and Supplementary File 2 (available at http://links.lww.com/PR9/A207).

### 2.2. Eligibility criteria

Multidimensional biopsychosocial assessment tools included in this review were those that (1) included variables reflecting at least 2 of the *Multidimensional Biobehavioral Model of Pain* domains of pain, precipitant variables, intervening variables, and functional status variables; (2) were developed specifically to measure the impact of pain rather than general functional interference not specific to pain; and (3) intended for use in patients with primary chronic pain diagnoses (as defined by the International Classification for Disease (*ICD-11*) classification).^72^ We excluded disease-specific tools, such as those for children with sickle cell disease or cancer and parent proxy measures.

Our inclusion criteria for studies describing these tools included the following: (1) The population exclusively involved pediatric patients (ages 2–18) with primary idiopathic chronic pain diagnoses/locations. (2) The outcomes of the study focused on the relationship between pain intensity and the multidimensional biopsychosocial items captured by the tool under investigation. (3) We excluded systematic reviews, meta-analyses, case studies, abstracts, and qualitative studies that did not include a psychometric outcome, such as a Cronbach alpha. We also excluded studies where the participant group included 50% or more of youth with secondary pain diagnoses related to an organic disease process such as cancer, sickle cell disease, juvenile rheumatoid arthritis, neurofibromatosis, or postsurgical pain.

### 2.3. Search strategy and information sources

The search strategy included 2 phases. Phase 1 searching focused on tool identification and was conducted in 2 measurement databases: PsychTEST and Health and Psychosocial Instruments (HAPI). Phase 2 searching focused on study identification, which involved a measure-forward search through 2 citation databases: Scopus and Web of Science. After eligible tools were selected in phase 1, a citation search of their development article was then conducted during phase 2. This search strategy was led by a librarian employed at The Children's Hospital of Eastern Ontario and was PRESS reviewed by a librarian employed at The Ottawa Hospital Research Institute. Both phases of this search were first conducted in February 2020 and repeated in February 2022. Details of the search strategy can be found in Supplementary File 3, available at http://links.lww.com/PR9/A207.

### 2.4. Study selection

A detailed instruction manual was developed based on the study eligibility criteria to guide the screening and retrieval process for phase 1 and phase 2 searches. One reviewer screened all results from the phase 1 search and a second reviewer confirmed inclusion or exclusion decisions made. All tools identified in the reference articles were listed in a Microsoft Excel document, which organized tools based on eligibility and included data on tool properties (ie, tool name, development reference, biopsychosocial domains/variables, and how they mapped to the *Multidimensional Biobehavioral Model of Pediatric Pain*).

After the citation analysis of the development articles for each included tool conducted in phase 2, all articles were uploaded to Covidence software to their respective project (ie, each tool represented its own project in Covidence). Two reviewers independently screened titles and abstracts. Full-text citations that met eligibility were then independently reviewed by 2 reviewers who further searched the text for additional eligible tools that may have been missed in the phase 1 search. Reviewers met on a biweekly basis to discuss discrepancies in eligibility assessments. All discrepancies were considered minor and were resolved. Details justifying elimination for excluded tools are listed in Supplementary File 4 and for excluded citations in Supplementary File 5 (available at http://links.lww.com/PR9/A207).

### 2.5. Data collection

A detailed data extraction instruction manual and data collection form was developed based on the study outcomes and contextual data. The data extraction process and form were piloted by 2 reviewers on 5 studies to ensure reliability of the data extraction instructions. Minor revisions were made. Two reviewers independently extracted the data based on explicit reporting of the following:

(1) Study characteristics, which included authors' names, year of publication, study purpose, population demographics, and methods (ie, study design).
(2) Reliability evidence (as defined by *the Standards for Educational and Psychological Testing*).^1^ All reliability evidence per the standards was possible for extraction.
(3) Validity evidence (as defined by *the Standards for Educational and Psychological Testing*).^1^ All validity evidence per the standards was possible for extraction.
(4) Clinical utility, as defined by Smart, 2006,^63^ which included data on tool appropriateness, accessibility, practicality, and acceptability.

Results from data extraction were compiled into summary tables, which were iteratively refined to best prepare for data synthesis and narrative description.

### 2.6. Methodological quality

Methodological quality and bias of all studies were assessed by 2 reviewers independently based on their study design and was guided respectively by the *Quality Assessment and Validity Tool for Cross-sectional Studies,*^19^ the *Quality Assessment and Validity Tool for Before and After/Cohort Design Studies,*^65^ and the *Revised Cochrane Risk of Bias Tool for Randomized Trials*.^35^ Studies were concluded to be weak, moderate, or strong based on the quality assessment tools.

### 2.7. Synthesis

The results from this review were synthesized descriptively. A meta-analysis was not appropriate because of the heterogeneity of studies describing the psychometric qualities of included multidimensional tools. Our synthesis involved a description of the relationships between pain and the biopsychosocial domains and subsequent variables that were measured across multidimensional tools. Our analysis focused on the validity evidence of each tool as it related to other variables. Significant bivariate and multivariate relationships were highlighted and tabulated across each pain domain and subsequent variable defined by the *Multidimensional Biobehavioral Model of Pediatric Pain*.^73^ Reported measures of tool reliability were tabulated in a descriptive form. Cronbach alphas were considered adequate if over and/or above 0.70.^25^ A sensitivity analysis was not appropriate because only 1 weak study was included in this review, and results were unlikely to change based on its removal.

## 3. Results

### 3.1. Tool and study selection

**Figure 1.**
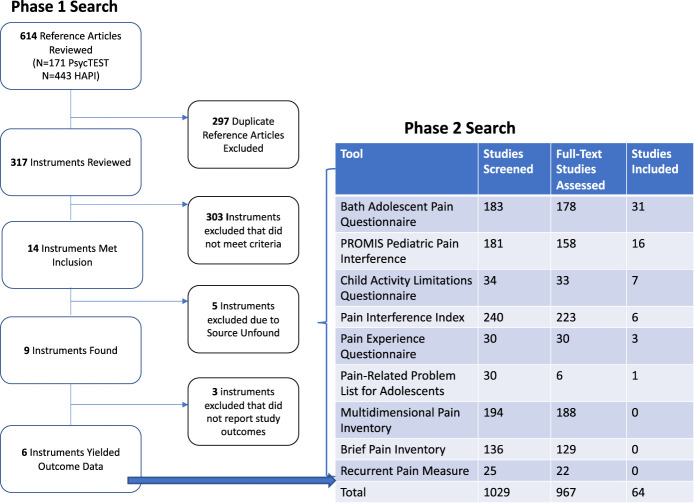
Search results. HAPI, Health and Psychosocial Instruments; PROMIS, Patient-Reported Outcomes Measurement Information System.

In the phase 1 search, 614 reference articles were screened for eligible tools. From this, 14 of 93 identified tools met eligibility criteria. Five of those tools were excluded because we could not locate the tool despite attempts to contact original authors. Details justifying exclusion for all tools reviewed can be found in Supplementary File 4 (available at http://links.lww.com/PR9/A207). In the phase 2 search, 1029 titles and abstracts, and 973 full-text articles were screened across 9 tools. This led to further exclusion of 3 tools that did not yield outcome data. Therefore, 6 tools were included in our synthesis, which generated a total of 64 eligible studies. Details of search results can be found in Figure 1. Details justifying exclusion of studies can be found in Supplementary File 5 (available at http://links.lww.com/PR9/A207).

### 3.2. Included tools

A summary of tool characteristics, including number of variables and domains as well as respective mapping to the *Multidimensional Biobehavioral Model of Pediatric Pain*^73^ is shown in Table 1. Variables reflecting the precipitant pain experience domain were not included in any of the 6 included tools. Results did not generate significant outcomes regarding clinical utility of tools, and thus information pertaining to clinical utility is described narratively below.

**Table 1:** Tool characteristics.

Tool	Development article	No. of items	No. of domains	Scoring method	Biopsychosocial domains		
					Precipitant variables	Intervening variables	Functional variables
BATH Adolescent Pain Questionnaire (BAPQ)	Eccleston et al.^16^	61	7	5-point Likert scale	0	44 items across 5 domains	17 items across 3 domains
PROMIS Pediatric Pain Interference Scale (PPPI)	Varni et al.^74^	8	N/A	4-point Likert scale	0	3 items	5 items
Child Activity Limitations Questionnaire (CALQ)	Hainsworth et al.^32^	21	N/A	5-point Likert scale	0	4 items	18 items
Pain Interference Index (PII)	Wicksell et al.^80^	6	N/A	7-point Likert scale	0	2 items	4 items
Pain Experience Questionnaire (PEQ)	Hermann et al.^34^	15	N/A	7-point Likert scale	0	3 items	12 items
Pain-Related Problem List for Adolescents (PRBL-A)	Weel et al.^82^	18	4	3-point Likert scale	0	5 items across 1 domain	13 items across 3 domains

### 3.3. Included studies

Among the 64 included studies, 46 were cross-sectional studies, 9 were cohort studies, 5 were randomized controlled trials, 3 were nonrandomized before and after studies, and 1 was a qualitative study. Most studies were conducted in the setting of tertiary-level outpatient pediatric chronic pain programs (n = 39) and included intensive inpatient pediatric chronic pain programs (n = 5), other speciality clinics (n = 8), research clinics (n = 1), schools (n = 2), and a data registry (n = 1). Nine studies did not specify their setting. Six studies were found to be secondary analyses of other included studies, and therefore, sample characteristics were not duplicated in our synthesis. Across the 64 studies included, a total of 19,429 participants aged between 6 and 19 years. Most participants across studies that reported sex and ethnicity were female (median 73%) and Caucasian (median 85%). Most participants in all studies had primary chronic pain diagnoses unrelated to an underlying condition, whereas 11 studies included less than 50% of participants with secondary chronic pain diagnoses and 7 studies included a description of comorbid mental health diagnoses. Study characteristics are reported in Table 2.

**Table 2:** Study characteristics.

Tool	Study characteristics				Sample characteristics							
	Article	Study type	Setting	Sample size	Age (y) Mean (SD)	Age (y) Range	Female (%)	Male (%)	Ethnicity (%)	Chronic pain diagnoses/location of pain (%)	Comorbid medical diagnoses (%)	Comorbid mental health diagnoses (%)
BATH Adolescent Pain Questionnaire (BAPQ)	Atikinson-Jones et al.^2^	Cross-sectional	Outpatient pain program (1 site)	68	12 (2.03)	11–18	73	27	Caucasian (91) Pakistani (6) Asian (1.5) Indian (1.5)	Widespread (66) CRPS (15) Back (6) Knee (4) Headache (4) Foot/leg (3) Hand/arm (1.5)	Hypermobility (33) Asthma (24) Spinal conditions (14) Cerebral palsy (5) Congenital heart disease (5) Vascular malformation (5)	Mental health unspecified (33) Anxiety and depression (36) ASD (9) ADHD (4.5) Tourette syndrome (4.5)
	Benore et al.^4^	Cohort	Outpatient pain program (1 site)	119	15.4 (2.67)	NR	75	25	Caucasian (94)	CRPS (34), Abdominal (21) Other (21) Headache (17) Back (7)	NR	NR
	Benore et al.^5^	Cohort	Outpatient pain programs (registry)	135	15.2 (2.2)	NR	74	26	Caucasian (96) African American (2) Hispanic (1) Asian (1)	Headaches and migraines (100)	NR	NR
	Caes et al.^8^	Cohort	Research clinic (1 site)	856	NR	17	65.5	34.5	NR	Back (54.5) Leg/foot (34.5) Knee (32) Shoulder (28) Abdominal (24) Buttock (23) Hip (20.5) Arm/hand (17) Headaches (14) Neck (13) Torso/sternum (10)	NR	NR
	Cohen et al.^10,11^	Cross-sectional	Outpatient pain program (2 sites)	222	14.8 (1.9)	10–18	75	25	Caucasian (99)	Whole-body (43) Limb (37) Back (8) Headaches (5) Abdominal pain (4) Hip (1) Chest (0.5)	NR	NR
	Connolly et al.^12^	Cross-sectional	Various outpatient pain and speciality clinics (number of sites NR)	128	14.4 (1.4)	12–18	68	32	NR	Unspecified primary chronic pain	NR	NR
	Eccleston et al.^16,17^	Cross-sectional	Outpatient pain programs (2 sites)	222	14.8 (1.9)	11–19	75	25	NR	Widespread (41) Limb (38) Back (9) Head (5) Abdomen (4) Hip (1) Chest (0.5)	Juvenile idiopathic arthritis (21)	NR
	Ecceleston et al.^18^	Cross-sectional	Outpatient pain program (1 site)	110	15.1 (1.9)	11–18	73	27	NR	Widespread (55) Limb (20 Back (10) Head (10) Abdomen (9) Hip (1)	NR	NR
	Fales et al.^21^	Cross-sectional	Various outpatient pain and speciality clinics (number of sites NR)	210	14.2 (1.6)	10–17	74	26	Caucasian (84) African American (5) Alaskan Native (3) Asian (1) Mixed (3) Other (3)	Musculoskeletal (81) Abdominal (32.5) Headaches (26)	NR	NR
	Fisher et al.^24^	Cohort	Research clinic (1 site)	855	13 and 17	NA	66	34	NR	Unspecified primary chronic pain (100%)	NR	NR
	Gagnon et al.^26^	Cross-sectional	Outpatient pain program (1 site)	202	14.1 (2.5)	8–18	77	23	NR	Headache (18) Central sensitization (12) CRPS (12) Abdominal (9) Joint (8) Back (8) Other (2)	Fibromyalgia (6) Scoliosis (1) EDS (8)	Functional neurological disorder (5)
	Gauntlett-Gilbert et al.^30^	Cross-sectional	Outpatient pain program (1 site)	110	15.1 (1.9)	NR	73	27	NR	Widespread (43) CRPS (37) Headache (8) Back (6) Abdominal (6)	NR	NR
	Gauntlett-Gilbert et al.^29^	Cross-sectional	Inpatient pain program (1 site)	355	15.6 (1.8)	10–19	77	23	NR	Widespread (34) CRPS (22) Back (13) Abdominal (10) Joint (8) Headaches (12)	Obesity (100) Hypermobility (8)	NR
	Harrison et al.^33^	Cross-sectional	Research database	196 (pain condition group)	NR	NR	67	33	Caucasian (88) Other (12)	Chronic primary pain unspecified (100%)	NR	NR
	Jordan et al.^39^	Qualitative	Inpatient pain program (1 site)	14	NR	12–16	93	7	Caucasian (88) Indian (1) African American (0.5)	Joint (21) Widespread (43) CRPS (29) Back (7)	Hypermobility (21)	NR
	Kemani et al.^40^	Nonrandomized clinical trial	Inpatient pain program (1 site)	164	15.5 (1.8)	11–18	77	22	NR	Widespread (66) CRPS (42) Back (17) Abdominal (13) Joint (10) Other (15)	Hypermobility (10)	NR
	Kersche et al.^41^	Cross-sectional	Outpatient pain program (1 site)	98	13.1 (2.4)	7–18	66	34	NR	Musculoskeletal (38) Visceral (30) CRPS (12) Widespread (12) Headache (8)	NR	NR
	Liam et al.^44^	Cross-sectional	Outpatient pain program	60	13.3 (2.5)	6–19	80	20	NR	Abdominal (38) Limb (35) Head/neck (22) Back (5)	NR	NR
	McCracken et al.^48^	Cross-sectional	Outpatient pain program (1 site)	122	15.2 (2.0)	10–18	75	25	NR	Chronic primary pain unspecified	NR	NR
	McGarrigle et al.^49^	Cross-sectional	Outpatient pain programs (multisite, not reported)	129	14.5 (1.4)	12–18	68	32	NR	Musculoskeletal (47) Headache (34) Abdominal (12) Other (7)	NR	NR
	Murray et al.^52^	RCT	Outpatient pain programs (15 sites)	273	14.7 (1.6)	11–17	75	25	Caucasian (85) African American (5) Hispanic (4) Other (5) NR (1.5)	Widespread (40) Musculoskeletal (42) Abdominal (11) Headaches (7)	NR	NR
	Palermo et al.^55^	RCT	Outpatient pain programs (2 sites)	61	14.3 (1.9)	10–17	80	20	Caucasian (90) African American (2) Asian (2) Other (6)	Musculoskeletal (39) Headache (29.5) Abdominal (29.5) Missing 92)	NR	NR
	Revivo et al.^57^	Cohort	Intensive pain program (1 site)	30	14 (2.8)	9–18	90	10	NR	Diffuse joint (100%)	Hypermobility (100%)	NR
	Robinson et al.^58^	Cross-sectional	Inpatient intensive pain program (1 site)	59	14.4 (2.6)	NR	73	27	Caucasian (86) African American (12) Other (2)	CRPS (32) Abdominal (22) Widespread (25)	EDS (15) Sickle cell disease (5) Concussion (3)	Conversion disorder (8.5)
	Sinclair et al.^62^	Cross-sectional	Outpatient pain program (site number not reported)	70	15.6 (1.2)	13–18	90	10	NR	Headache (30) Limb (24) Widespread (17) Back (14) Abdominal (8.5) Hip (3) CRPS (3)	NR	NR
	Vowles et al.^77^	Cross-sectional	Outpatient pain program	222	4.3 (4.1)	10–18	75	25	Caucasian (99)	Widespread (44) Limb (39) Back (8) Headaches (5) Abdominal/chest (5)	NR	NR
	Waldron et al.^79^	Cross-sectional	Outpatient pain program (3 sites)	148	14.6 (1.3)	13–17	72	28	Caucasian (94)	Chronic primary pain unspecified (100%)	NR	NR
PROMIS Pediatric Pain Questionnaire	Bhandari et al.^6^	Cohort	Outpatient pain program (1 site)	328	14.7 (2.3)	8–17	72	28	Non-Hispanic (63) Hispanic (28) Unknown (12)	Musculoskeletal (22) Headache (21) Abdominal (14) CRPS (6) Fibromyalgia (4)	EDS (2) Rheumatological condition (1.5) Sickle cell disease (0.3)	Primary psychological diagnosis (1.2)
	Birnie et al.^7^	Cross-sectional	Outpatient pain program (1 site)	806	14.5 (2.4)	8–17	72	28	European American (60) Asian (9) African American (3) Alaskan (0.5) Native Hawaiian (0.4) Other (16) Declined to report (12)	Musculoskeletal (24) Headache (24) Abdominal (16) CRPS (7) Widespread (3)	EDS (1.7) Rheumatological condition (1)	NR
	Chan et al.^13^	Cross-sectional	Outpatient pain clinic (1 site)	172	14.8 (1.7)	12–18	76	24	Caucasian (92) African American (4) Other (4)	Chronic pain including widespread pain, regional pain syndromes, headaches, and abdominal pain (frequencies not provided)	NR	NR
	Dutta et al.^15^	Cohort	Outpatient pain program (1 site)	64	15 (1.7)	11–19	84	16	Caucasian (84) African American (6) Hispanic (2) Asian (2) Mixed (5) Other (2)	Chronic pain unspecified	NR	NR
	Feinstein et al.^23^	Cross-sectional	Data registry	325	15.5 (1.4)	8–17	74	26	Caucasian (67) Asian (7) African American (3) Alaskan (1) Other (11) Declined to state (2) Missing (6)	Musculoskeletal (37) Headache (18) Abdominal (15) CRPS (10) Fibromyalgia (5.5) Other (9.5) Missing (2)	Rheumatological condition (0.9) EDS (0.9)	Primary psychological diagnosis (1)
	Feinstein et al.^22^	Cross-sectional	Outpatient pain program (1 site)	324	14.7 (2.1)	10–17	73	27	Hispanic (19) Non-Hispanic (69) Missing (39)	Musculoskeletal (39) Abdominal (19) Headache (24) CRPS (5) Fibromyalgia (1.5) Other (4) Missing (1)	EDS (0.3) Rheumatological condition (3)	Primary psychological diagnosis (2.5)
	Gamwell et al.^27^	Cross-sectional	Gastroenterology clinics (several, unspecified)	5281	13 (3)	8–18	60	40	Caucasian (91) African American (6) Asian (1) American Indian (0.1) Middle Eastern (0.04) Other (1.1) Not reported (0.8)	Abdominal (100)	Gastroesophageal reflex (5) Irritable bowel syndrome (4)	NR
	Kersch et al.^41^	Cross-sectional	Outpatient pain program (1 site)	98	13.1 (2.4)	7–18	66	34	NR	Musculoskeletal (42) Visceral (29) CRPS (12) Widespread (8) Headache (8)	NR	NR
	Morris et al.^51^	RCT	Not specified	278	14.6 (2)	11–17	65	35	NR	Functional abdominal pain (100)	NR	NR
	Ross et al.^59^	Cross-sectional	Outpatient pain program (1 site)	328	14.9 (1.5)	12–18	79	21	Caucasian (62) Hispanic (21) Asian (10) African American (4)	Headache (18) Abdominal (17) Joint (12.5) Back (12) CRPS (7) Fibromyalgia/pain amplification (5) Lower extremity (5) Other (23)	NR	NR
	Salamon et al.^60^	Cohort	Outpatient pain program (1 site)	94	14.6 (1.8)	9–18	74	26	Caucasian (85) African American (7) Hispanic (3) Multiple (3) Asian (2)	Headache (44) Back (32) Joint (20) Abdominal (19)	Chronic fatigue syndrome (9) POTS (6) Sickle cell disease (1) Other unspecified (7)	NR
	Sánchez-Rodríguez et al.^61^	Cross-sectional	Schools (3 sites)	218	14.4 (1.8)	12–18	62	38	NR	Headaches (73) Back (57) Abdominal/pelvis (57) Leg (44) Neck (39) Feet (31) Shoulder (28) Arm (20) Chest/breast (19)	NR	NR
	Soltani et al.^64^	Cross-sectional	Outpatient pain program (1 site)	145	13.3 (2.6)	NR	67	33	Caucasian (81) Multiple (8) Asian (4) Latin American (3.5) Asian Other (3) Declined (0.7)	Headaches (61) Complex pain (38) Abdominal (1)	NR	NR
	Stoner et al.^67^	Cross-sectional	Gastroenterology clinic (1 site)	138	14.2 (1.8)	NR	68	32	NR	Abdominal (100)	NR	NR
	Stone et al.^68^	Cohort	Gastroenterology clinic (1 site)	278	NR	11–17	66	34	Caucasian (86)	Functional abdominal pain (100)	NR	NR
	Yoon et al.^83^	Cross-sectional	Outpatient pain program (1 site)	285	13.9 (2.5)	8–17	71	29	Caucasian (58) Non-Hispanic (62)	Musculoskeletal (30) Headache (24) Abdominal (22) CRPS (5) Neuropathic (4) Fibromyalgia (2)	Rheumatological conditions (1.8)	NR
Child Activity Limitations Questionnaire (CALQ)	Cunningham et al.^14^	Cross-sectional	Study 1: outpatient pain program (1 site) Study 2: outpatient pain program (1 site)	959 207	14.0 (2.4) 14.2 (2.6)	NR NR	70 77	30 23	Caucasian (79) African American (8) Hispanic (5) Asian (0.2) Native American (0.03) Multiple (4) Other (1) Not reported (0.5) Caucasian (87) African American (4) Hispanic (1) Asian (1) Native American (1) Multiple (2) Other (0.5) Not reported (3)	Head (41) Extremities (17.5) Back (13) Abdominal (13) Joint (13) Generalized (8) Other (6) Back (30) Abdominal (23) Joint (17) Generalized (11) Extremities (10) Head (3) Other (3.5)	NR NR	NR NR
	Evans et al.^20^	Cross-sectional	Outpatient pain program (1 site)	310	15.2 (1.5)	12–18	68	32	Caucasian (78)	Headache (34) Back (18) Lower extremity (15) Abdominal (12.5)	NR	NR
	Hainsworth et al.^32^	Cross-sectional	Outpatient pain program (1 site)	62	14.0 (2.7)	8–18	82	18	Caucasian (42) African American (7) Multiple (7) Latino (4)	Headaches (43) Migraines (10) Abdominal (17) Back (10) Neuropathic (10) Joint (7) Chest (2) Foot (2)	NR	NR
	Jagpal et al.^37^	Cross-sectional	Outpatient pain program (1 site)	770	14.2 (2.4)	8–18	70	30	Caucasian (80)	Headaches (41) Trunk (17) Extremities (17)	NR	NR
	Salamon et al.^60^	Cross-sectional	Outpatient pain program (1 site)	278	14.1 (2.6)	8–18	69	31	Caucasian (77) African American (9) Multiple (4) Hispanic (5) American Indian (1) Missing (4)	Head (37) Abdomen (16) Back (16) Lower extremity (12) Upper extremity (6) Generalized (6) Other (6)	NR	NR
	Stoner et al.^67^	Cross-sectional	Outpatient pain program (1 site)	461	13.9 (2.4)	8–18	73	27	Caucasian (84) African American (7) Multiple (4) Hispanic (3) Other (0.9) Native American (0.4) Middle Eastern (0.2) Asian (0.1)	Headaches (41) Extremities (17) Abdominal (15) Trunk (16) Other (12)	Obesity (24.5)	NR
	Tran et al.^71^	Cross-sectional	Outpatient pain program (1 site)	725	14.1 (2.5)	8–18	69	31	Caucasian (75) African American (9) Latino (5) Biracial/Other (11)	Headaches (37) Abdominal (15) Lower extremity (15) Back (14)	NR	NR
Pain Interference Index (PII)	Balter et al.^3^	Noncontrolled before and after study	Outpatient pain program (1 site)	47	14.8 (2.2)	9–19	70	30	NR	Chronic pain unspecified	NR	Autistic traits (81) ADHD symptoms (85)
	Holmström et al.^36^	Cross-sectional	Outpatient pain program (1 site)	163	14.1 (2.2)	7–18	74	26	NR	Chronic pain unspecified	NR	NR
	Lipsker et al.^45^	Cross-sectional	Outpatient pain program (1 site)	146	14.5 (2.4)	NR	70	30	NR	Headache (80) Abdominal (60) Leg (60)	NR	NR
	Wicksell et al.^80^	RCT (secondary analysis)	Behavior medicine treatment service (1 site)	32	14.6 (SD NR)	10–18	77	23	NR	Back/neck (22) Headache (19) Widespread (19) CRPS (19) Visceral (6) Lower extremity (6) Postherpetic (3)	NR	NR
	Wicksell et al.^81^	Cross-sectional	Outpatient pain program (1 site)	163	14.1 (2.6)	NR	74	26	NR	Headache (66) Abdominal (41) Back (31) Joint (23) Widespread (12)	NR	NR
	Zetterqvist et al.^85^	Nonrandomized pilot study	Outpatient pain program (1 site)	28	15.4 (1.3)	13–17	100	0	NR	Headache (75) Abdominal (39) Back (46) Leg (46) Throat (4) Chest (7) Arm/hand (25) Teeth (7) Ear (7) Pelvis/hip (14) Menstrual (36) Neck (7) Joint (7) Foot (7)	NR	NR
Pain Experience Questionnaire (PEQ)	Calvano et al.^9^	Cross-sectional	Gastroenterology clinics (16 sites)	151	10.9 (2.6)	6–17	65	35	NR	Abdominal (100)	NR	NR
	Hermann et al.^34^	Cross-sectional	NR	111	11.5 (1.8)	7–18	42	58	NR	Headache (66) Abdominal (22) Fibromyalgia (13)	NR	NR
	Offenbächer et al.^53^	Cross-sectional	Rheumatology clinic (1 site)	329	13.9 (2.5)	NR	81	19	NR	Musculoskeletal (100)	NR	NR
Pain-Related Problem List for Adolescents (PRBL-A)	Weel et al.^82^	Cross-sectional	School (1 site)	129	15.1 (1.6)	12–18	70	30	NR	Headaches (30) Limb (19) Back (13) Abdominal (12) Neck (5)	NR	NR

CRPS, complex regional pain syndrome; EDS, Ehler–Danlos syndrome; NR, not reported.

### 3.4. Bath Adolescent Pain Questionnaire (n = 31 studies)

The Bath Adolescent Pain Questionnaire (BAPQ) was developed in 2005 and was designed specifically for use in adolescents with chronic pain.^16^ Development involved expert consultation and focus groups with adolescents with chronic pain.^16^ Authors for all included BAPQ citations in this study referenced the BAPQ as appropriate for use in adolescents with chronic pain, noting its reliability and validity. Eccleston et al.^18^ demonstrated clinical utility of the BAPQ parent proxy for use with populations who cannot complete the questionnaire. A noted limitation of the BAPQ was that it does not address nonadolescent age groups.^46^

### 3.5. Patient-Reported Outcomes Measurement Information System Pediatric Pain Interference Scale (n = 16 studies)

The Patient-Reported Outcomes Measurement Information System (PROMIS) Pediatric Pain Interference Scale (PPPI) was developed in 2010 through the National Institutes of Health (NIH) PROMIS initiative, with the intention of developing item banks and computerized adaptive tests (CATs) that are applicable for a wide variety of chronic pain disorders.^50^ Within this initiative, the PROMIS Pediatric Cooperative Group had developed the pediatric self-report item banks, which included the PPPI that was guided by item response theory (IRT) to analyze scale dimensionality, item local dependence, and differential item functioning.^74^ Authors of 5 PPPI-cited studies in this review emphasized its strengths related to CAT and IRT by means of improving precision. It has been highlighted that CAT is an approach by which items are selected based on responses to previously administered items, which ultimately reduces respondent burden and optimizes scale completion.^22^ The PPPI has also been used in a variety of chronic pain conditions, widening its utility across a more generalized pain population.^6^ No studies in this review noted PPPI limitations.

### 3.6. Child Activity Limitations Questionnaire (n = 7 studies)

The Child Activity Limitations Questionnaire (CALQ) was adapted from the Child Activity Limitations Interview (CALI) in 2007 which was designed to assess functional impairment in children and adolescents with chronic pain.^32^ Four studies referenced good reliability and validity of the tool in measuring functional interference in the pediatric population with chronic pain.^20,32,37,67^ The clinical utility of the tool was considered enhanced over the CALI because it includes a written self-report version rather than an interview, reducing the administration time to approximately 2 to 3 minutes.^32^ Child Activity Limitations Questionnaire–focused studies in this review did not discuss its limitations.

### 3.7. Pain Interference Index (n = 6 studies)

The Pain Interference Index (PII) was developed to evaluate the influence of pain on functioning in the pediatric population with chronic pain in clinical assessments, treatment evaluations, and research studies. The PII was shown to have good reliability and validity and excellent completion rates.^47^ A cited strength of the PII is its focus on the impact of pain interference compared with more general functioning.^80^ Citations included in the PII review did not discuss limitations of the tool; however, it is important to recognize that only 6 of 240 citations screened for the PII met eligibility for this review.

### 3.8. Pain Experience Questionnaire (n = 3 studies)

The Pain Experience Questionnaire (PEQ) was originally adapted from the German Multidimensional Pain Inventory for Adults^25^ in 2008, intended to assess the psychosocial impact of chronic pain in children and adolescents.^34^ All 3 studies included in the review for the PEQ emphasized its good reliability and validity, and appropriateness for use in the pediatric population with chronic pain. Limitations were not mentioned in studies included in the PEQ review.

### 3.9. Pain-Related Problem List for Adolescents (n = 1 study)

The Pain-Related Problem List for Adolescents (PRBL-A) was developed in 2005 to measure personal pain-related problems in adolescents with chronic pain.^82^ The only citation that met eligibility for this review showed high internal consistency, good convergent and divergent validity, and good anticipated clinical utility, as it focuses on what patients believe are their main pain-related problems.^82^

Reliability and validity evidence captured through this review are presented in Table 3.

**Table 3:** Reliability and validity evidence of included tools in the pediatric populations with chronic pain.

Tool	Article	Reliability evidence *Cronbach* ∝ *Test–retest (r)*	Validity evidence												
			Evidence based on internal structure *CFA/EFA* *Subgroup comparison* *Comparison with other measures*	Evidence based on relationships to other variables Between pain (intensity/frequency/duration/modulation) and biopsychosocial domains *(Correlation Measurement; Magnitude/Significance)*											
				Pain and intervening variables				Pain and functional variables							Pain and general interference
				Pain catastrophizing/adaptability	Social Support/Dysfunction	Family support/Dysfunction	Develop mental functioning	Activity/school functioning	Physical functioning	Concentration	Affective distress	Anxiety	Pain anxiety	Depression/Mood	
Bath Adolescent Pain Questionnaire (BAPQ)	Atkinson-Jones et al.^2^	∝ 0.85–0.92	—	—	—	—	—	—	—	—	—	—	—	—	—
	Benore et al.^4^	Test–retest *r* 0.64–0.81 ∝ 0.80–0.83	—	— **β 0.568**	— —	— —	— —	— —	— —	— —	—	***r* 0.41** β 0.129	***r* 0.436** **β 0.443**	— —	— —
	Benore et al.^5^	∝ 0.80 (total)	—	—	—	—	—	—	—	—	—	—	—	—	—
	Caes et al.^8^	∝ 0.87 (total)	—	—	**β (−) 0.165**	—	—	**β 0.193**	—	—	—	—	—	—	**β 0.349**
	Cohen et al.^10^	∝ 0.79–0.89	—	—	***r* 0.31**	—	—	—	***r* 0.46**	—	—	—	—	—	—
	Cohen et al.^9^	∝ 0.80–0.85	—	—	—	—	—	—	—	—	—	—	—	—	—
	Connolly et al.^12^	∝ 0.80–0.87	—	—	—	—	—	—	—	—	—	—	—	—	—
	Eccleston et al.^16^	∝ 0.80–0.85 Test–retest *r* 0.64–1.00	EFA/CFA χ^2^ 62.66 RMSEA 0.071 GFI 0.90	—	***r* 0.26**	***r* 0.22**	***r* 0.40**	—	***r* 0.34**	—	—	***r* 0.22**	***r* 0.28**	***r* 0.40**	—
	Eccleston et al.^17^	∝ 0.82–0.93	—	—	*r* 0.07	*r* 0.11	*r* 0.13	—	***r* 0.21**	—	—	*r* 0.15	*r* 0.05	***r* 0.18**	—
	Eccleston et al.^18^	—	—	—	***r* 0.24**	***r* 0.24**	—	—	—	—	—	***r* 0.22**	***r* 0.29**	***r* 0.40**	
	Fales et al.^21^	∝ 0.88 (total)	—	—	—	—	—	—	—	—	—	—	—	—	—
	Fisher et al.^24^	∝ 0.75–0.86	—	—	—	—	—	—	—	—	—	—	—	—	—
	Gagnon et al.^26^	∝ 0.94 (total)	—	—	***r* (−) 0.28**	***r* (−) 0.26**	*r* (−) 0.11	—	***r* (−) 0.30**	—	—	***r* (−) 0.21**	***r* (−) 0.33**	***r* (−) 0.19**	—
	Gauntlett-Gilbert et al.^30^	∝ >0.80 (all subscales)	—	—	—	—	—	—	—	—	—	—	—	—	—
	Gauntlett-Gilbert et al.^29^	—	—	—	β 0.00	β (−) 0.02	β 0.07	—	**β 0.18**	—	—	β 0.02	β 0.01	β 0.01	—
	Harrison et al.^33^	∝ 0.87 (1 subscale)	—	—	—	—	—	—	—	**OR 1.79**	—	—	**OR 2.19**	**OR 2.04**	**OR 3.09**
	Jordan et al.^39^	∝ 0.76–0.91	—	—	—	—	—	—	—	—	—	—	—	—	—
	Kermani et al.^40^	∝ 0.64–0.85	—	—	—	—	—	—	—	—	—	—	—	—	—
	Kersch et al.^41^	—	—	—	—	—	—	—	—	—	—	—	***r* 0.388**	***r* 0.308**	—
	Lim et al.^44^	—	—	**β 0.313**	—	—	—	—	—	—	—	*r* 0.238	***r* 0.327**	*r* 0.203 **β 0.501**	—
	McCracken et al.^48^	—	—	—	*r* 0.049	*r* 0.12	*r* 0.13	—	***r* 0.26**	—	—	*r* 0.12	*r* 0.063	***r* 0.23**	—
	McGarrigle et al.^49^	∝ 0.85–0.86	—	—	—	—	—	—	—	—	—	***r* 0.42**	—	***r* 0.49**	—
	Murray et al.^52^	∝ 0.83–0.84	—	—	—	—	—	—	—	—	—	—	—	—	—
	Murray et al.^52^	∝ 0.83–0.84	—	—	—	—	—	—	—	—	—	—	—	—	—
	Palermo et al.^55^	∝ 0.83–0.84	—	—	—	—	—	—	—	—	—	—	—	—	—
	Palermo et al.^56^	∝ 0.85–0.88	—	—	—	—	—	—	—	—	—	—	—	—	—
	Revivo et al.^57^	Test-retest *r* 0.60–0.94	—	—	—	—	—	—	—	—	—	—	—	—	—
	Robins et al.^58^	∝ 0.82–0.84	—	—	—	—	—	—	—	—	—	—	—	—	—
	Sinclair et al.^62^	—	**-**	***r* 0.25**	**β (−) 2.01**	—	—	β (−) 1.97	**β (−) 5.79**	—	***-***	—	—	**β (−) 3.84**	**β 4.19**
	Vowles et al.^77^	—	—	—	—	—	—	—	—	—	—	—	—	—	—
	Waldron et al.^79^	∝ >0.66	—	—	*r* 0.14	—	—	—	***r* 0.35**	—	—	—	—	*r* 0.02	—
PROMIS Pediatric Pain Interference (PII) scale	Bhandari et al.^6^	—	—	—	—	—	—	—	—	—	—	—	—	—	***r* 0.377**
	Birnie et al.^7^	—	—	—	—	—	—	—	—	—	—	—	—	—	ß 0.02
	Chan et al.^13^	∝ 0.88 (total)	—	—	—	—	—	—	—	—	—	—	—	—	***r* 0.39**
	Dutta et al.^15^	∝ 0.73 (total)	—	—	—	—	—	—	—	—	—	—	—	—	*r* 0.21
	Feinstein et al.^23^	—	—	—	—	—	—	—	—	—	—	—	—	—	***r* 0.448 (children)** ***r* 0.42 (adolescents)**
	Feinstein et al.^22^	—	—	—	—	—	—	—	—	—	—	—	—	—	***r* 0.384**
	Gamwell et al.^27^	∝ 0.92 (total)	—	—	—	—	—	—	—	—	—	—	—	—	***r* 0.501**
	Kersch et al.^41^	—	—	—	—	—	—	—	—	—	—	—	—	—	***r* 0.321**
	Morris et al.^51^	—	—	—	—	—	—	—	—	—	—	—	—	—	ß 0.393 (baseline) **ß −0.108 (overtime)**
	Ross et al.^59^	—	—	—	—	—	—	—	—	—	—	—	—	—	***r* 0.38** **ß 0.33**
	Salamon et al.^60^	∝ 0.84–0.90	—	—	—	—	—	—	—	—	—	—	—	—	—
	Sánchez-Rodríguez et al.^61^	∝ 0.85 (total)	—	—	—	—	—	—	—	—	—	—	—	—	***r* 1.11** **β 0.32** **PC 1.10–1.13**
	Soltani et al.^64^	∝ 0.84 (total)	—	—	—	—	—	—	—	—	—	—	—	—	***r* 0.54**
	Stone et al.^69^	—	—	—	—	—	—	—	—	—	—	—	—	—	***r* 0.48**
	Stone et al.^68^	∝ 0.85 (total)		—	—	—	—	—	—	—	—	—	—	—	—
	Yoon et al.^83^	—	—	—	—	—	—	—	—	—	—	—	—	—	***r* 0.283** **PC 0.105–0.116**
Child Activity Limitations Questionnaire (CALQ)	Cunningham et al.^14^	—	—	—	—	—	—	—	—	—	—	—	—	—	***r* 0.41 (study 1)** ***r* 0.41 (study 2)**
	Evans et al^20^	∝ 0.93 (total)	—	—	—	—	—	—	—	—	—	—	—	—	—
	Hainsworth et al.^32^	—	—	—	—	—	—	—	—	—	—	—	—	—	***r* 0.31–0.40**
	Jagpal et al.^37^	∝ 0.89 (total)	—	—	—	—	—	—	—	—	—	—	—	—	**ß 4.74**
	Salamon et al.^60^	∝ 0.96 (total)	—	—	—	—	—	—	—	—	—	—	—	—	***r* 0.23–0.40**
	Stoner et al.^67^	∝ 0.95 (total)	—	—	—	—	—	—	—	—	—	—	—	—	—
	Tran et al.^71^	—	—	—	—	—	—	—	—	—	—	—	—	—	***r* 0.41**
Pain Interference Index (PII)	Balter et al.^3^	∝ 0.83 (total)	—	—	—	—	—	—	—	—	—	—	—	—	—
	Holmström et al.^36^	∝ 0.86 (total)	Good construct validity (through comparison with FDI and CES-DC & hierarchical regression analyses)	—	—	—	—	—	—	—	—	—	—	—	***r* 0.381**
	Lipsker et al.^45^	∝ 0.86 (total)	—	—	—	—	—	—	—	—	—	—	—	—	***r* 0.267**
	Wicksell et al.^80^	∝ 0.84 (total)	—	—	—	—	—	—	—	—	—	—	—	—	**PC 0.52–0.60**
	Wicksell et al.^81^	∝ 0.86 (total)	—	—	—	—	—	—	—	—	—	—	—	—	***r* 0.39** **PC 1.28–1.29**
	Zetterqvist et al.^85^	∝ 0.81 (total)	—	—	—	—	—	—	—	—	—	—	—	—	—
Pain Experience Questionnaire (PEQ)	Calvano and Warschburger^9^	∝ 0.89 (total)	—	—	—	—	—	—	—	—	—	—	—	—	***r* 0.302**
	Hermann et al.^34^	∝ 0.71–0.87	EFA/CFA χ^2^ 603.34 GFI 0.90 CFI 0.96 RMSEA 0.06 External validity demonstrated through subgroup comparison	—	*r* (−) 0.06–0.10	—	—	—	—	—	***r* 0.28** **−0.35**	—	—	—	***r* 0.34–0.35**
	Offenbächer et al.^53^	—	—	—	—	—	—	—	—	—	—	—	—	—	***r* 0.50**
Pain-Related Problem List for Adolescents (PRBL-A) (n = 1 article)	Weel et al.^82^	∝ 0.71–0.86	Good construct validity through comparison with CPDI, PedMIDAS and PAQoL-A	—	—	—	—	—	***r* 0.28–0.43**	***r* 0.03–0.37**	***r* −0.03** to **0.24**	—	—	***r* 0.03–0.21**	***r* 0.08–0.48**

Bolded = Significant.

−, negative relationship; CFA, confirmatory factor analysis; EFA, exploratory factor analysis; OR, odds ratio; PC, path coefficient; *r*, Spearman correlation (through test–retest); α, Cronbach alpha; β, unstandardized beta.

### 3.10. Reliability evidence

The reliability evidence captured in this review included internal consistency coefficients and test–retest coefficients. Other reliability coefficients (eg, alternate-form coefficients or generalizability coefficients) were not revealed in included studies. Most studies provided reliability evidence on the tool under study, including 43 studies that reported Cronbach alphas to be acceptable (ie, over 0.70). Of those, 41 studies reported Cronbach alphas to be >0.80 across various domains and total values. Two studies reported inadequate Cronbach alphas for 2 domains within the BAPQ—namely, 0.66 for the social domain^40^ and 0.66 for the physical functioning variable.^79^ Across all 6 tools, 3 studies reporting on the BAPQ^4,16,57^ measured test–retest reliability and showed it to be adequate in all cases. Other reliability coefficients (eg, alternate-form coefficients or generalizability coefficients) were not measured in included studies. The amount of reliability evidence found reflected the amount of eligibility articles found per tool. The BAPQ review contained most of the reliability evidence (n = 26 studies), followed by the PPPI (n = 7 studies), the PPI (n = 6 studies), the CALQ (n = 4 studies), the PEQ (n = 3 studies), and the PRBL-A (n = 1 study).

### 3.11. Validity evidence

The validity evidence captured in this review included structural validity identified through confirmatory and exploratory factor analyses and convergent validity identified through relationships to other variables. Results focused mainly on relationships to other variables and were found through both bivariate and multivariate relationships between pain and biopsychosocial domains highlighted within tool items. This review did not yield results on response process, content validity, or cross-cultural validity. Four studies across 4 tools (BAPQ, PII, PEQ, or PRBL-A) reported validity evidence based on the internal structure. These studies used exploratory and confirmatory factor analyses (n = 2 studies), comparison with other measures (n = 2 studies), and subgroup comparison (n = 1 study), which all showed satisfactory results. No studies in the review assessed response process, content validity, or cross-cultural validity. All studies offered validity evidence for the tools based on relationships to other pain-related variables because this was a main outcome of the review. A total of 84 of 112 relationships (75%) measured between biopsychosocial variables and pain were found to be significant. Of those, 20 of 29 (69%) were multivariate relationships measured through unstandardized betas (n = 16 relationships) and path coefficients (n = 4 relationships), and 65 of 84 (77%) were bivariate relationships measured through correlation coefficients (n = 61 relationships) and odds ratios (n = 4 relationships).

Across all 6 tools, 30 studies measured 37 relationships between pain and *general functional interference*. Of those, 34 relationships were found to be significant that included 10 multivariate correlations and 24 bivariate correlations.

### 3.12. Intervening variables

Four variables were mapped within the intervening variables domain across 13 studies. The BAPQ provided most data within this domain, reporting on all 4 of the variables, followed by the PEQ reporting on 1 variable. Across the 13 studies, most significant relationships were between pain and *social functioning* (n = 6/11, 55% significant relationships), followed by pain and *family functioning* (n = 3/6, 50% significant relationships), pain and *pain catastrophizing/adaptability* (n = 3/3, 100% significant relationships), and pain and *developmental functioning* (n = 1/5, 20% significant relationships).

### 3.13. Functional variables

Seven variables were mapped within the functional variable domain across 18 studies, involving the BAPQ, the PEQ, and the PRBL-A. Across the 18 studies, most (n = 11/14, 78.5% significant relationships) were found between pain and *depression/mood*, followed by pain and *physical functioning* (n = 9/9, 100% significant relationships), pain and *pain-related anxiety* (n = 8/11, 73% significant relationships), pain and *anxiety* (n = 5/10, 50% significant relationships), pain and *affective distress* (n = 2/2, 100% significant relationships)*,* pain and *concentration* (n = 2/2, 100% significant relationships), and pain and *activity/school functioning* (n = 1/1, 50% significant relationships).

### 3.14. Methodological quality

Table 4 presents the methodological quality of studies as it pertains to each included tool. Most studies received a quality rating of moderate or strong with only a single study being rated as weak.

**Table 4:** Methodological quality of studies.

Tool	Article	Methodological quality
Bath Adolescent Pain Questionnaire (BAPQ)	Atkinson-Jones et al.^2^ Benore et al.^4^ Benore et al.^5^ Caes et al.^8^ Cohen et al.^10^ Cohen et al.^11^ Connolly et al.^12^ Eccleston et al.^16^ Eccleston et al.^17^ Eccleston et al.^18^ Fales et al.^21^ Fisher et al.^24^ Gagnon et al.^26^ Gauntlett-Gilbert et al.^30^ Gauntlett-Gilbert et al.^29^ Harrison et al.^33^ Jordan et al.^39^ Kermani et al.^40^ Kersch et al.^41^ Liam et al.^44^ McCracken et al.^48^ McGarrigle et al.^49^ Murray et al.^52^ Murray et al.^52^ Palermo et al.^55^ Palermo et al.^55^ Revivo et al.^57^ Robinson et al.^58^ Sinclair et al.^62^ Vowles et al.^77^ Waldron et al.^79^	Moderate Moderate Moderate Moderate Moderate Moderate Strong Moderate Moderate Moderate Strong Moderate Moderate Moderate Moderate Moderate Moderate Moderate Moderate Moderate Moderate Moderate Moderate Strong Strong Moderate Moderate Moderate Moderate Moderate Strong
PROMIS Pediatric Pain Interference (PII) scale	Bhandari et al.^6^ Birnie et al.^7^ Chan et al.^13^ Dutta et al.^15^ Feinstein et al.^23^ Feinstein et al.^22^ Gamwell et al.^27^ Kersch et al.^41^ Morris et al.^51^ Ross et al.^59^ Salamon et al.^60^ Sánchez-Rodríguez et al.^61^ Soltani et al.^64^ Stone et al.^68^ Stone et al.^69^ Yoon et al.^83^	Moderate Moderate Moderate Moderate Moderate Moderate Moderate Moderate Moderate Strong Strong Strong Moderate Moderate Moderate Moderate
Child Activity Limitations Questionnaire (CALQ)	Cunningham et al.^14^ Evans et al.^20^ Hainsworth et al.^32^ Jagpal et al.^37^ Salamon et al.^60^ Stoner et al.^67^ Tran et al.^71^	Strong Moderate Moderate Moderate Moderate Moderate Moderate
Pain Interference Index (PII)	Balter et al.^3^ Holmström et al.^36^ Lipsker et al.^45^ Wicksell et al.^80^ Wicksell et al.^81^ Zetterqvist et al.^85^	Moderate Moderate Strong Moderate Moderate Weak
Pain Experience Questionnaire (PEQ)	Calvano and Warschburger^9^ Hermann et al.^34^ Offenbächer et al.^53^	Moderate Moderate Moderate
Pain-Related Problem List for Adolescents (PRBL-A)	Weel et al.^82^	Moderate

## 4. Discussion

Through this review, we aimed to identify multidimensional tools that specifically measure the biopsychosocial impact of chronic pain in the pediatric population. Our strategy has guided a systematic analysis of the relationship between pain and biopsychosocial variables, which help to describe the significant effect that pain has on the pediatric chronic pain experience. Results from this review revealed 6 reliable and valid multidimensional biopsychosocial tools for use in children and adolescents living with chronic pain, described in 64 studies. Furthermore, our findings highlight 11 biopsychosocial variables across 2 domains of the pediatric chronic pain experience that feature the ways in which biopsychosocial variables may be interpreted and weighed in predicting patient complexity in the pediatric population with chronic pain. This knowledge may ultimately empower pediatric chronic pain programs to better prioritize care and tailor interdisciplinary pain management according to individualized biopsychosocial needs.

### 4.1. Psychometric relevance

Although several related tools used in the pediatric population with chronic pain have been identified, only 6 offer a holistic biopsychosocial pain assessment in chronic pain not specific to an underlying disease. Our reliability evidence primarily demonstrated internal consistency data measured through Cronbach alphas, presenting the relationships among items or subsets of items within each tool. Our findings provide confidence in the homogeneity among items within each tool because all 6 tools have exhibited acceptable internal consistency. Our validity findings focused predominantly on validity evidence assessed as relationships between pain and other related variables, which revealed the degree to which these relationships are consistent with the construct (pain interference) underlying the proposed test score interpretations.^70^ All 6 tools demonstrated good convergent validity, highlighting evidence of several multivariate and bivariate relationships between pain and specific biopsychosocial variables, as well as pain and general functional interference. Further illuminating our findings, it is noteworthy that only 1 of 64 studies was considered weak, reflecting confidence in our synthesis and conclusions drawn.

### 4.2. Clinical relevance

Our findings show that the biopsychosocial domains comprising intervening variables and functional variables are associated with chronic pain and are important to assess in children and adolescents. Furthermore, our review simultaneously offers 6 reliable and valid multidimensional tools that specifically measure the impact of chronic pain in pediatric patients. Although it was our intention to report on the clinical utility of selected tools, our review did not generate substantial data in that regard. The BAPQ and the PPPI are the most studied tools found within our review; however, their usability and efficiency are limited. The BAPQ is lengthy with 61 items across 7 domains, and the PPPI, although brief in nature, poses scoring complexity by means of T-score metrics that is not clearly explained in the literature and may deter use. Regarding underrepresented tools in the literature, the PEQ offers biopsychosocial representation through 15 items; however, its scoring instructions are not explicit. It is also important to note that only 30 studies were identified at the screening phase in the PEQ review, highlighting its limited psychometric evaluation in the literature. The CALQ offers an efficient and clear administration and scoring system by simple summation of the scoring on 21 items on a 5-point Likert scale and identifying the 8 most bothersome activities. Similarly, the PRBL-A involves a total of 18 items across 4 relevant biopsychosocial domains and is scored by averaging the items within the domains and then summing the domain scores. Further research into the CALQ and PRBL-A in pediatric chronic pain practice is warranted to support understanding of tool clinical utility and psychometric value. Of the 6 tools reviewed, the PII offers the shortest multidimensional assessment platform that inclusively captures pain interference in general activities, physical activities, friendships, school, and sleep in 6 short questions. Furthermore, the PII involves a simple scoring system that includes calculating the average of completed items. These usability advantages may be considered more appealing to referring providers who wish to offer a quick biopsychosocial assessment for patients being referred to interdisciplinary pediatric chronic pain programs. The multidimensional nature of the tool provides a holistic assessment that may efficiently guide prioritization of care and tailoring of pain interventions based on the biopsychosocial needs.

### 4.3. Limitations

Despite developing a comprehensive search strategy that was formally PRESS reviewed and involved 2 iterative phases including relevant psychometric databases, it is possible that not all eligible instruments were identified. The phase 1 search strategy involved a tedious manual search of eligible tools within research articles where potential tools may have been missed during the review of articles. This risk was mitigated through use of 2 independent reviewers. An additional layer to this limitation includes the fact that several tools have been referenced using various names, causing confusion. For example, the terms CALQ and CALI, though distinct tools, were used interchangeably between articles. We intended for our study to be focused on pediatric patients with primary chronic pain diagnoses; however, strictly limiting this criterion would have omitted significant findings. Therefore, a small number of articles including a small representation of secondary chronic pain diagnoses were included. The availability of multidimensional biopsychosocial tools measuring the impact of chronic pain in pediatric patients is relatively scarce, and thus we believe including these studies would provide more advantage than disadvantage when synthesizing the literature on this topic. Consequently, our synthesis cannot confirm generalizability to patients solely with primary chronic pain diagnoses. It is important to note that the definitions within the *Multidimensional Biobehavioral Model of Pediatric Pain* domains are ambiguous and there was an element of subjectivity in how the biopsychosocial variables within each tool were mapped to the model's domains.

### 4.4. Future directions

At this time, the biopsychosocial variables identified through this review to be significantly affected by chronic pain could be assessed at various time points across the pediatric chronic pain journey, which may ultimately inform clinical practice. Although this review offers promising multidimensional biopsychosocial pain tools that specifically measure the impact of chronic pain and are supported by reliability and validity evidence, further psychometric and clinical utility research is warranted to confidently make recommendations for tool use in guiding clinical decision making in pediatric interdisciplinary chronic pain programs. Research on these tools could be expanded specifically for use in a triage assessment to help prioritize access to interdisciplinary pediatric pain care, considering the current limited availability of interdisciplinary pediatric chronic pain programs worldwide. This would provide assurance of the most psychometrically robust, rapidly administered and clinically useful tools that can be easily administered by referring providers before the triage assessment. We believe this would enhance the validity and reliability of triage assessments and improve prioritization of interdisciplinary pain care for patients based on the holistic impact of their chronic pain experience. With recall bias in mind, the clinical use of such tools could be expanded further to assess baseline and ongoing patient needs during intake and follow-up assessments. This could help clinicians efficiently tailor the most appropriate interdisciplinary pain interventions and assess effectiveness of proposed interventions.

## 5. Conclusion

This review is the first of its kind to systematically retrieve pain focused reliable and valid multidimensional tools that holistically measure biopsychosocial interference in children and adolescents with chronic pain. Furthermore, this review has capitalized on the psychometric strengths of the tools to robustly emphasize the significant relationships between pain and the biopsychosocial domains and subsequent variables of the pediatric chronic pain experience. Findings from this review emphasize the strength of the relationship between pain and functional interference and offer a categorized and objective overview of 11 biopsychosocial variables across 2 domains that are significantly affected by chronic pain in children and adolescents. This review offers enhanced guidance regarding biopsychosocial assessments for children and adolescents referred to interdisciplinary chronic pain programs and details avenues for further psychometric and clinical utility research. We believe that the understudied tools found in this review are promising and could be easily adopted by both referring providers and chronic pain providers. Such tools may improve prioritization of access to interdisciplinary chronic pain programs worldwide and tailor interdisciplinary care within such programs based on biopsychosocial needs.

## Disclosures

The authors declare that the research was conducted in the absence of any commercial or financial relationships that could be construed as a potential conflict of interest.

## Appendix A. Supplemental digital content

A supplemental appendix is available at http://links.lww.com/PR9/A207.
