# Supplementary material for: A systematic review of the biopsychosocial dimensions affected by chronic pain in children and adolescents: identifying reliable and valid pediatric multidimensional chronic pain assessment tools
Source: Pain Rep. 2023 Nov 28;8(6):e1099. doi: 10.1097/PR9.0000000000001099 (PMC10686605; doi:10.1097/PR9.0000000000001099)
Supplement: Supplementary file 1 [file painreports-8-e1099-s001.pdf]

List of Supplementary Files

Supplementary File 1 – PRISMA-P Checklist
Supplementary File 2 – PRISMA-P Flow Diagrams
Supplementary File 3 – Search Strategy and PRESS Review
Supplementary File 4 – Reasons for Tool Exclusion
Supplementary File 5 – Reasons for Citation Exclusion

Supplementary File 1: PRISMA-P Checklist

Section and Topic	Item #	Checklist item	Location where item is reported
**TITLE**			
Title	1	Identify the report as a systematic review.	Title page
**ABSTRACT**			
Abstract	2	See the PRISMA 2020 for Abstracts checklist.	Pg 2
**INTRODUCTION**			
Rationale	3	Describe the rationale for the review in the context of existing knowledge.	Pg 3-4
Objectives	4	Provide an explicit statement of the objective(s) or question(s) the review addresses.	Pg 4
**METHODS**			
Eligibility criteria	5	Specify the inclusion and exclusion criteria for the review and how studies were grouped for the syntheses.	Pg 5
Information sources	6	Specify all databases, registers, websites, organisations, reference lists and other sources searched or consulted to identify studies. Specify the date when each source was last searched or consulted.	Pg 5-6 Supplementary File 2
Search strategy	7	Present the full search strategies for all databases, registers and websites, including any filters and limits used.	Pg 5-6 Supplementary File 2
Selection process	8	Specify the methods used to decide whether a study met the inclusion criteria of the review, including how many reviewers screened each record and each report retrieved, whether they worked independently, and if applicable, details of automation tools used in the process.	Pg 6-7
Data collection process	9	Specify the methods used to collect data from reports, including how many reviewers collected data from each report, whether they worked independently, any processes for obtaining or confirming data from study investigators, and if applicable, details of automation tools used in the process.	Pg 6-7
Data items	10a	List and define all outcomes for which data were sought. Specify whether all results that were compatible with each outcome domain in each study were sought (e.g. for all measures, time points, analyses), and if not, the methods used to decide which results to collect.	Pg 7
	10b	List and define all other variables for which data were sought (e.g. participant and intervention characteristics, funding sources). Describe any assumptions made about any missing or unclear information.	Pg 7
Study risk of bias assessment	11	Specify the methods used to assess risk of bias in the included studies, including details of the tool(s) used, how many reviewers assessed each study and whether they worked independently, and if applicable, details of automation tools used in the process.	Pg 7-8
Effect measures	12	Specify for each outcome the effect measure(s) (e.g. risk ratio, mean difference) used in the synthesis or presentation of results.	Pg 8

Section and Topic	Item #	Checklist item	Location where item is reported
Synthesis methods	13a	Describe the processes used to decide which studies were eligible for each synthesis (e.g. tabulating the study intervention characteristics and comparing against the planned groups for each synthesis (item #5)).	Pg 8
	13b	Describe any methods required to prepare the data for presentation or synthesis, such as handling of missing summary statistics, or data conversions.	Pg 8
	13c	Describe any methods used to tabulate or visually display results of individual studies and syntheses.	Pg 8
	13d	Describe any methods used to synthesize results and provide a rationale for the choice(s). If meta-analysis was performed, describe the model(s), method(s) to identify the presence and extent of statistical heterogeneity, and software package(s) used.	Pg 8
	13e	Describe any methods used to explore possible causes of heterogeneity among study results (e.g. subgroup analysis, meta-regression).	N/A, addressed on Pg 8
	13f	Describe any sensitivity analyses conducted to assess robustness of the synthesized results.	N/A, addressed on Pg 8
Reporting bias assessment	14	Describe any methods used to assess risk of bias due to missing results in a synthesis (arising from reporting biases).	Pg 7
Certainty assessment	15	Describe any methods used to assess certainty (or confidence) in the body of evidence for an outcome.	Pg 7
**RESULTS**			
Study selection	16a	Describe the results of the search and selection process, from the number of records identified in the search to the number of studies included in the review, ideally using a flow diagram.	Pg 8-9<br>Fig 1
	16b	Cite studies that might appear to meet the inclusion criteria, but which were excluded, and explain why they were excluded.	Supplementary Files 3 & 4
Study characteristics	17	Cite each included study and present its characteristics.	Citation List Pg 27-38<br>Table 2
Risk of bias in studies	18	Present assessments of risk of bias for each included study.	Table 4
Results of individual studies	19	For all outcomes, present, for each study: (a) summary statistics for each group (where appropriate) and (b) an effect estimate and its precision (e.g. confidence/credible interval), ideally using structured tables or plots.	Table 3
Results of syntheses	20a	For each synthesis, briefly summarise the characteristics and risk of bias among contributing studies.	Table 2 & 4
	20b	Present results of all statistical syntheses conducted. If meta-analysis was done, present for each the summary estimate and its precision (e.g. confidence/credible interval) and measures of statistical heterogeneity. If comparing groups, describe the direction of the effect.	Table 3
	20c	Present results of all investigations of possible causes of heterogeneity among study results.	N/A

Section and Topic	Item #	Checklist item	Location where item is reported
	20d	Present results of all sensitivity analyses conducted to assess the robustness of the synthesized results.	N/A
Reporting biases	21	Present assessments of risk of bias due to missing results (arising from reporting biases) for each synthesis assessed.	Table 4
Certainty of evidence	22	Present assessments of certainty (or confidence) in the body of evidence for each outcome assessed.	Table 4
**DISCUSSION**			
Discussion	23a	Provide a general interpretation of the results in the context of other evidence.	Pg 15-19
	23b	Discuss any limitations of the evidence included in the review.	Pg 17-18
	23c	Discuss any limitations of the review processes used.	Pg 17-18
	23d	Discuss implications of the results for practice, policy, and future research.	Pg 18-19
**OTHER INFORMATION**			
Registration and protocol	24a	Provide registration information for the review, including register name and registration number, or state that the review was not registered.	Pg 19
	24b	Indicate where the review protocol can be accessed, or state that a protocol was not prepared.	Not prepared
	24c	Describe and explain any amendments to information provided at registration or in the protocol.	Pg 19-20
Support	25	Describe sources of financial or non-financial support for the review, and the role of the funders or sponsors in the review.	Pg 20
Competing interests	26	Declare any competing interests of review authors.	Pg 20
Availability of data, code and other materials	27	Report which of the following are publicly available and where they can be found: template data collection forms; data extracted from included studies; data used for all analyses; analytic code; any other materials used in the review.	Not yet publically available

Supplementary File 2 – PRISMA-P Flow Diagrams

BATH Adolescent Pain Questionnaire

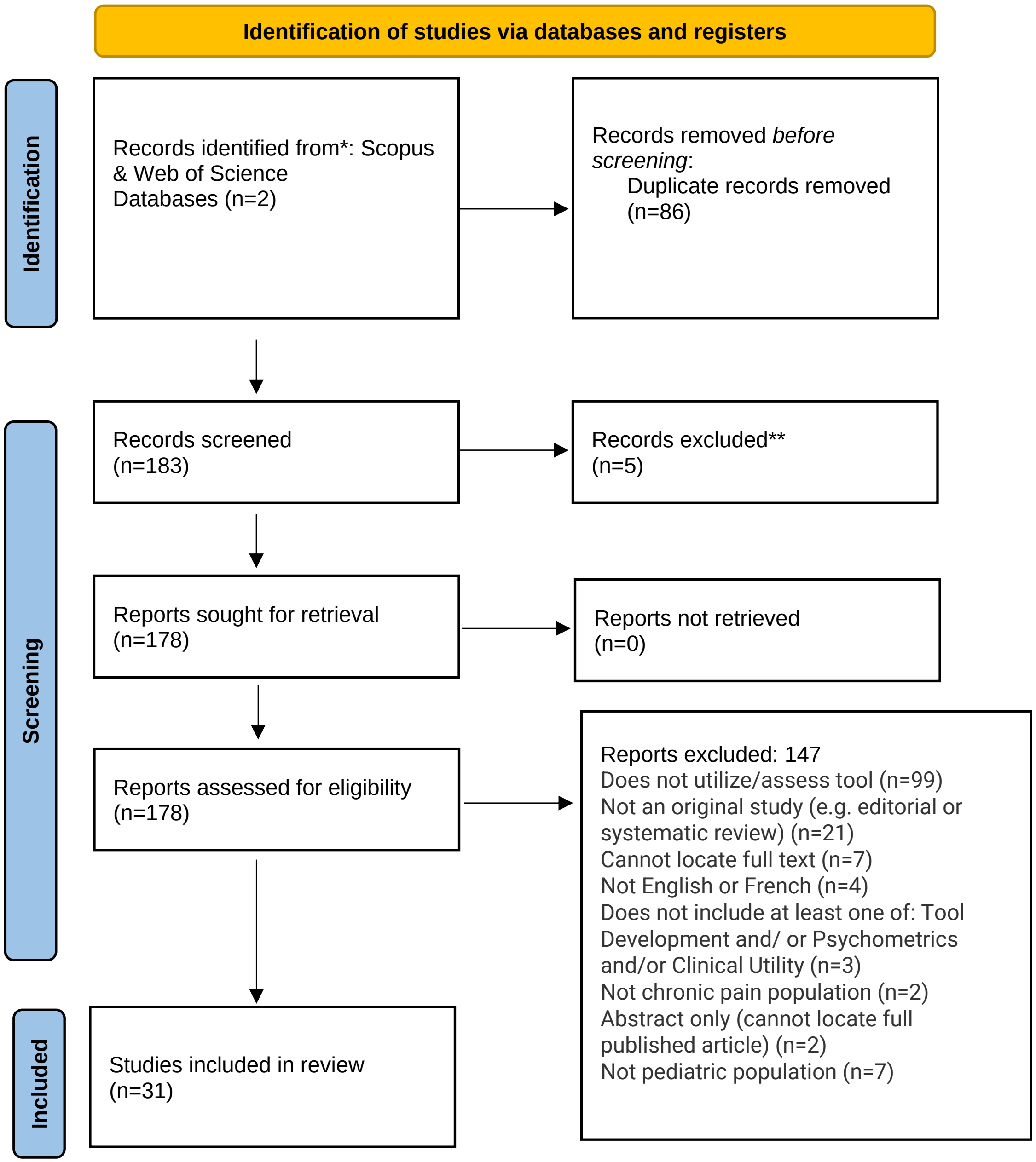

PROMIS Pain Interference Scale

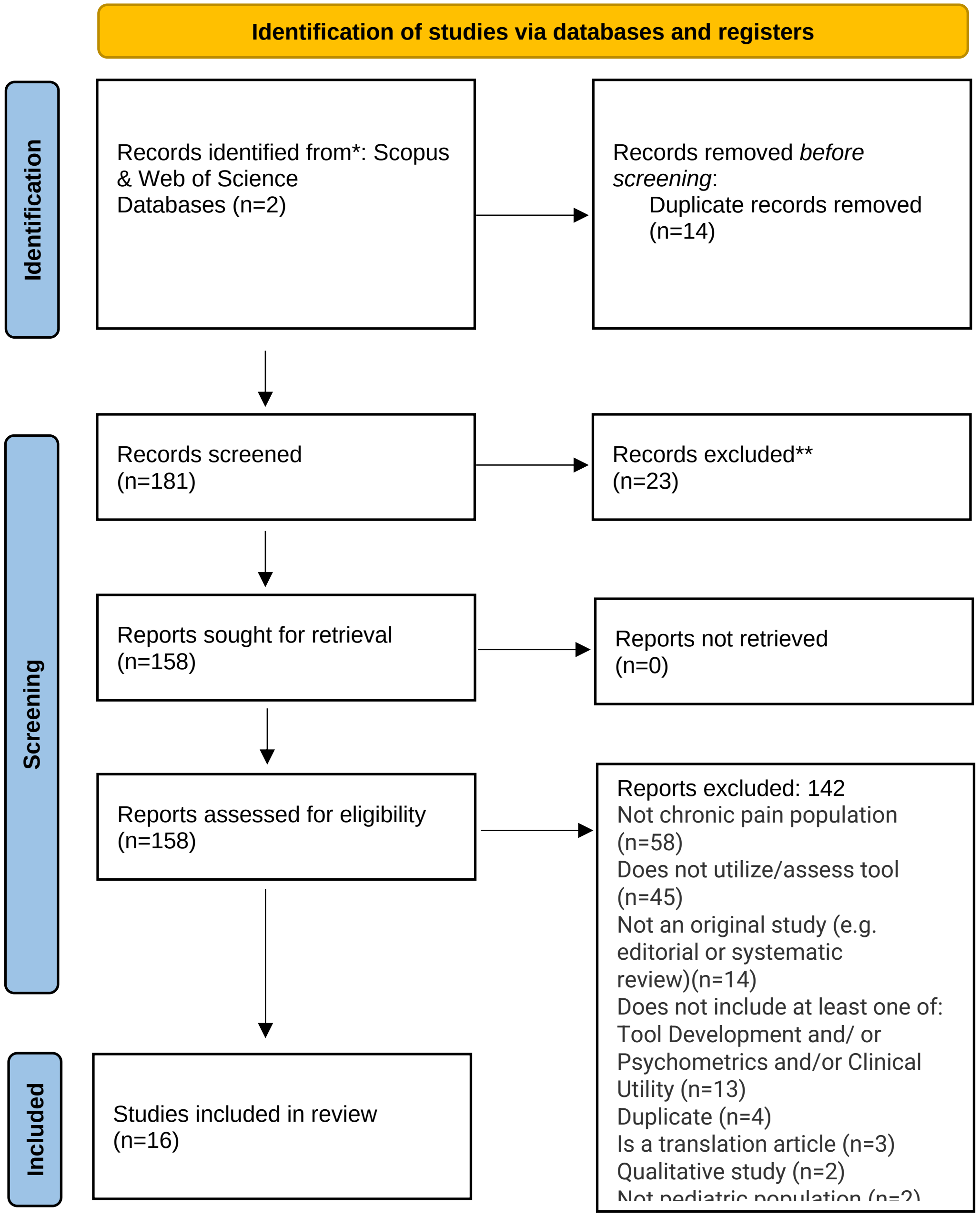

Child Activities Limitations Questionnaire (CALQ)

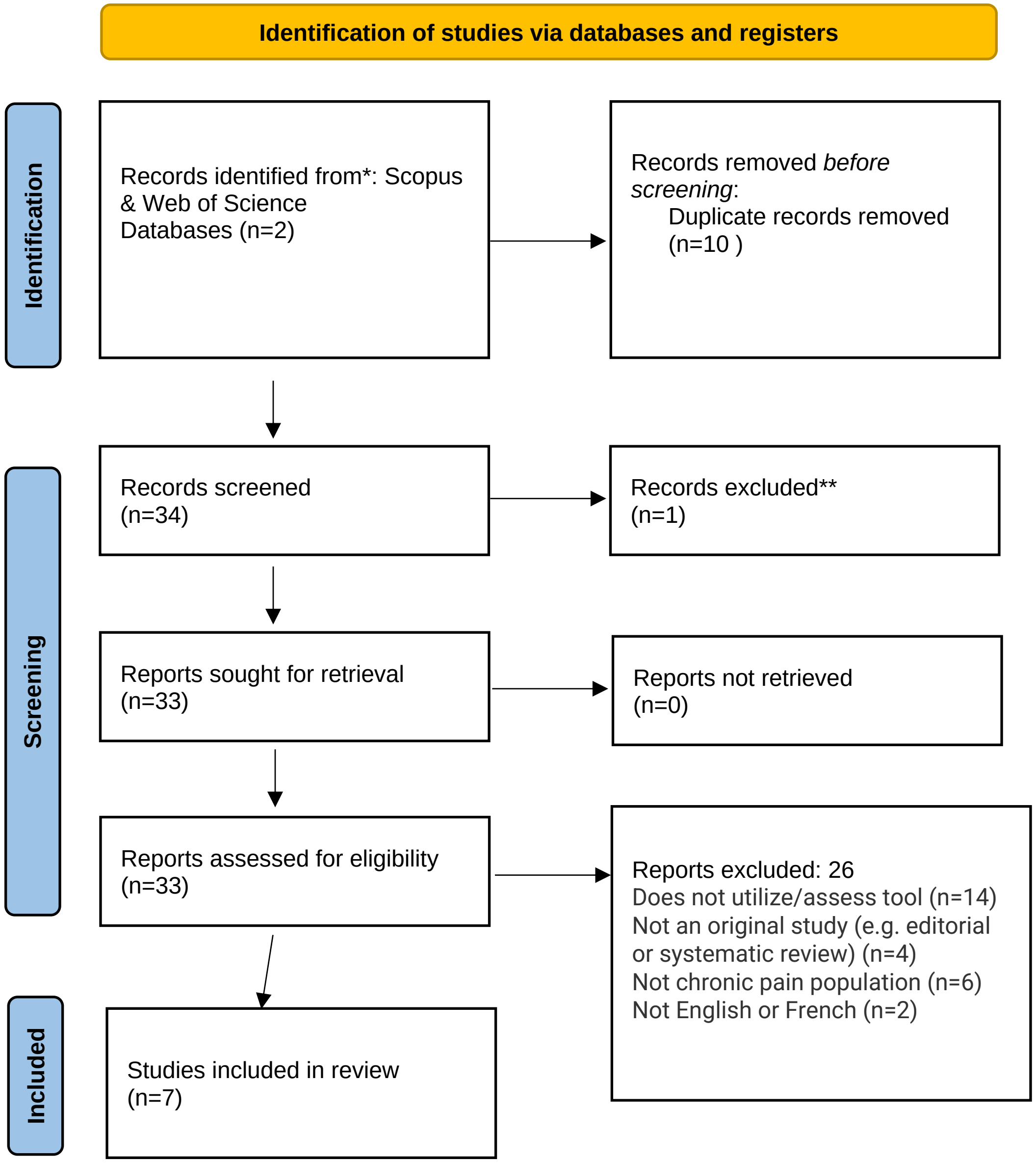

Pain Interference Index (PII)

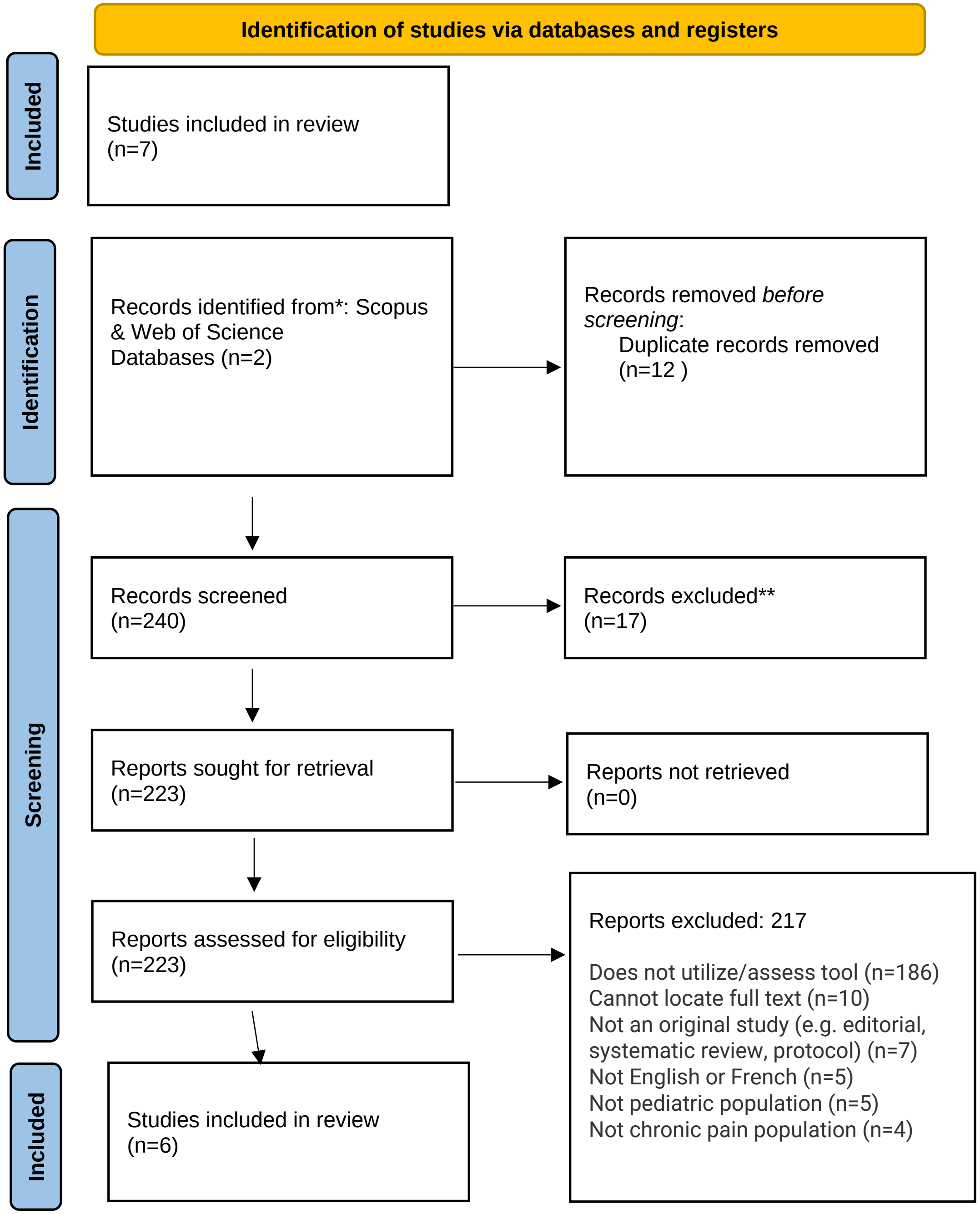

Pain Experience Questionnaire (PEQ)

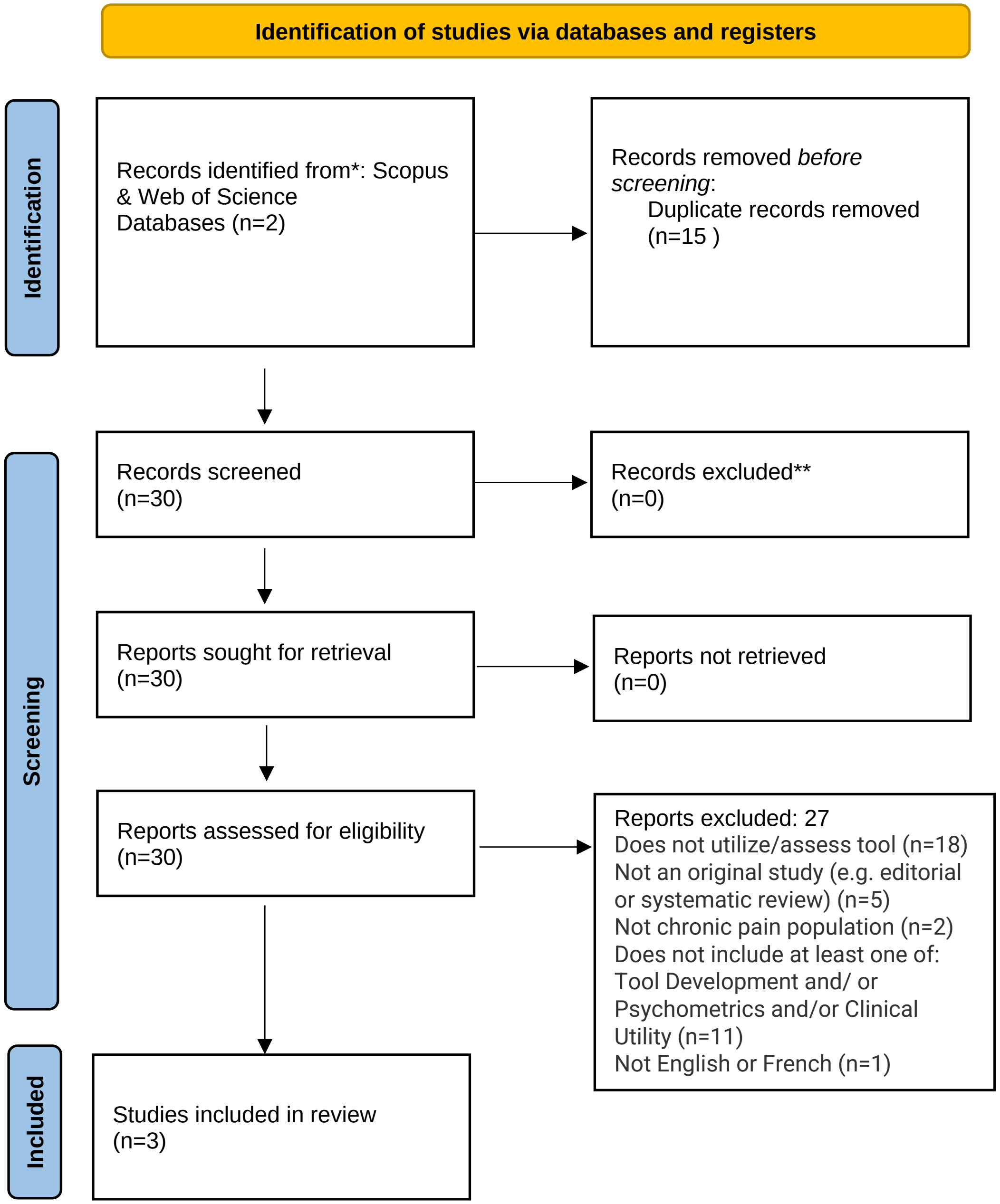

Pain Related Problem List for Adolescents (PRBL-A)

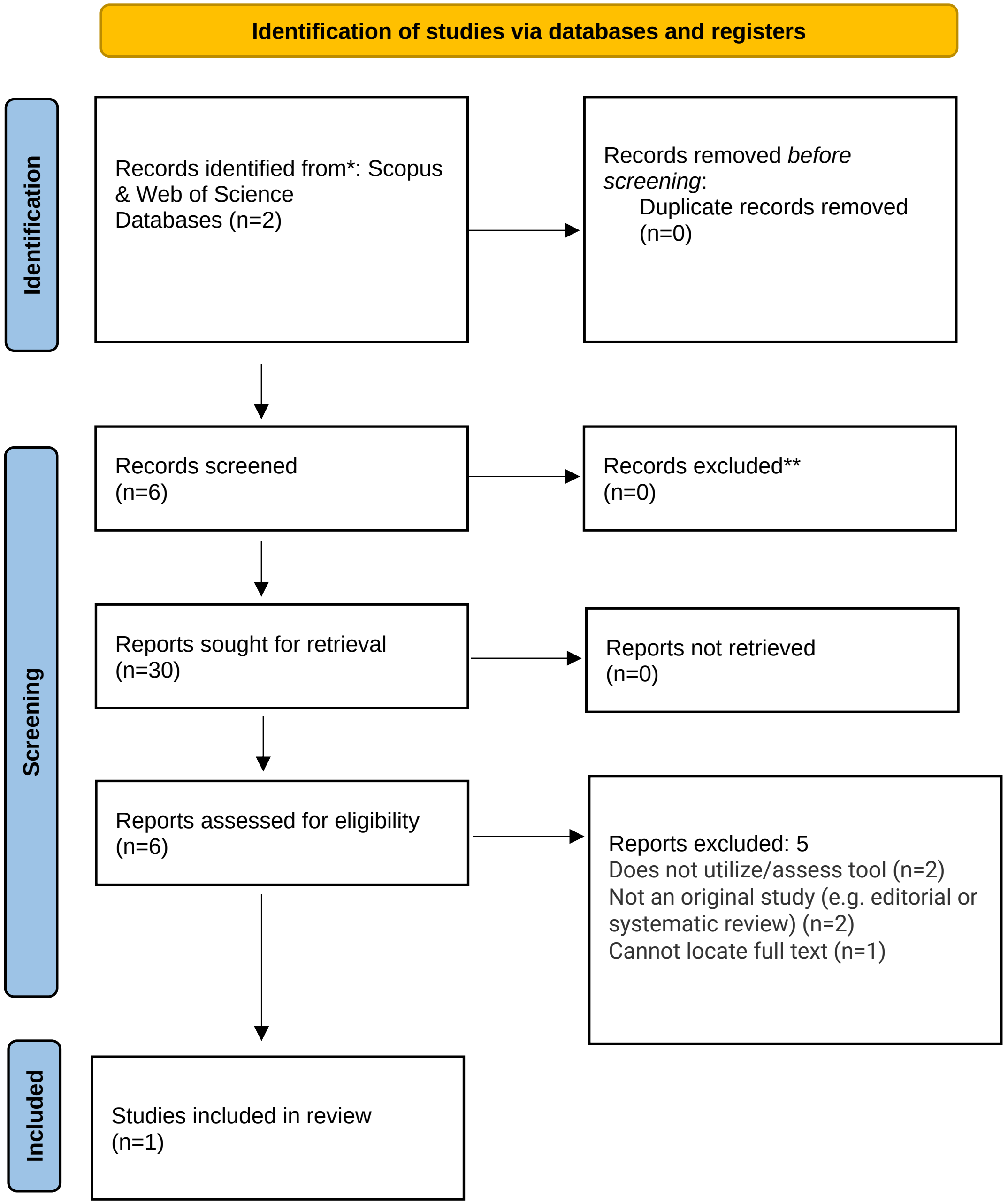

Supplementary File 3 – Search Strategy and PRESS Review

PEER REVIEW ASSESSMENT: THIS SECTION TO BE FILLED IN BY THE REVIEWER (Tamara R)

1. PsycTEST

(results need to be reviewed online)
Suggested search;

pain
and
biopsychosocial* or bio-psycho-social* or biobehav* or bio-behav* or psychosocial* or psycho-social* or multidimension* or dimension* or interdisciplin* or multidisciplin* or multi-disciplin* or function* or dysfunction* or distress or coping or experience or burden or limitation*
And from the pulldowns:
Age Group: Childhood (birth-12 yrs) OR Adolescence (13-17 yrs)

This yields 142 records. If you just search Pain and the age group filter, omitting the biopsychosocial terms, you will get 228 records

PychTEST does not seem to have a way limit by language.

2. Health and Psychosocial Instruments (HAPI)

Results can be exported to a citation manager, but you typically lose a lot of detail on the test as there are many fields beyond the normal fields in a citation database.
Note that if you change the first line from pain.mp. (where *mp* means search all fields) to pain.ti. (where *ti* means search in the title) your final numbers drop from 406 to 73. Omit the last row if you want to consider all languages.

1	pain.mp.	8699
2	(biopsychosocial* or bio-psycho-social* or biobehav* or bio-behav* or psychosocial* or psycho-social* or multidimension* or dimension* or interdisciplin*	37253

	or multidisciplin* or multi-disciplin* or function* or dysfunction* or distress or coping or experience or burden or limitation*).mp.	
3	(toddler* or child* or school* or adolescen* or juvenil* or youth* or teen* or pubescen* or pediatric* or paediatric* or peadiatric*).mp.	60875
4	1 and 2 and 3	523
5	limit 4 to english language	406

Biopsychosocial terms:
biopsychosocial* or bio-psycho-social* or biobehav* or bio-behav* or psychosocial* or psycho-social* or multidimension* or dimension* or interdisciplin* or multidiscipline* or multi-disciplin* or function* or dysfunction* or distress or coping or experience or burden or limitation*

Child term – (add or omit the shaded terms, depending on your lower age of interest):
Infan* OR newborn* OR new-born* OR perinat* OR neonat* OR baby OR baby* OR babies OR toddler* or child* or school* or adolescen* or juvenil* or youth* or teen* or pubescen* or pediatric* or paediatric* or peadiatric*

Reviewer: Tamara	Email: tamara.rader@gmail.com Date completed: 5 Oct 2020

1. TRANSLATION

A ---No revisions	X
B --- Revision(s) suggested	☐
C --- Revision(s) required	☐

2. BOOLEAN AND PROXIMITY OPERATORS

A ---No revisions	X
B --- Revision(s) suggested	☐
C --- Revision(s) required	☐

3. SUBJECT HEADINGS

A ---No revisions	X
B --- Revision(s) suggested	☐
C --- Revision(s) required	☐

4. TEXT WORD SEARCHING

A ---No revisions	☐
B --- Revision(s)suggested	X
C --- Revision(s) required	☐

5. SPELLING, SYNTAX, AND LINE NUMBERS

A ---No revisions	X
B --- Revision(s)suggested	☐
C --- Revision(s) required	☐

6. LIMITS AND FILTERS

A ---No revisions	X
B --- Revision(s) suggested	☐
C --- Revision(s) required	☐

OVERALL EVALUATION (Note: If one or more "revision required" is noted above, the response below must be "revisions required".)

A ---No revisions	☐

B --- Revision(s) suggested	X
C --- Revision(s) required	☐

Supplemental File 4 - Reasons for Tool Exclusion

Tool	Tool Not Intended to Measure Impact of Chronic Pain Interference	Tool Focused on Specific Disease Process or Pain Location	Unidimensional	No evidence for use in pediatrics	Does not Provide Quantitative Measure	Insufficient Details Available
	(i.e., general health status tools, tools focused on pain coping/ perception/ focus on acute pain)	(i.e., Headache specific tools, Sickle Cell specific tool, etc.)	(i.e., did not fit criteria defined by the Multidimensional Biobehavioral Model of Pediatric Pain)	(i.e., within the ages of 2-18 years, neonatal/ infant tools excluded)	(i.e., interviews, etc.)	Cannot Locate Tool PDF/ Insufficient Description of Tool
Pain Discomfort Scale (Jensen, Turner, Romano & Strom, 1995)			X			
Pain Impairment and Relationship Scale (Slater, Hall, Atkinson & Garfin, 1991)	X		X			
Chronic Pain Values Inventory (McCracken & Yang, 2006)	X		X			
Recurrent Pain Measure (Vaalamo et al., 2002)						X
Varni/ Thompson Pediatric Pain Questionnaire (Varni, Wilcox, Handon & Birk, 1988)			X			
Biopsychosocial Perspective of Pain Measure (Guite et al., 2014)						X
Structured Pain Interview (Gil, Abrams, Phillips & Williams, 1992)					X	
Daily Pain and Activity Diary (Powers et al., 2002)					X	

Psychosocial Pain Inventory (Heaton et al., 1982)						X
Pain and Discomfort Module (Mason, Skevington & Obsnorn, 2004)						X
Pain Status Form (Lester, Lefebvre & Keefe, 1996)						X
Chronic Pain Grade Questionnaire (Smith et al., 1997)				X		
Comprehensive Pain Evaluation Questionnaire (Birch & Jamison, 1998)				X		
Dartmouth Pain Questionnaire (Corson & Schneider, 1984)				X		
Wisconsin Brief Pain Questionnaire (Daut, Cleeland & Flanery, 1983)				X		
Euro-QOL 5D (Strong et al., 2008)				X		
SF-Health Survey (Ware et al., 1993)	X			X		
Pain Coping Questionnaire (Kleinke, 1992)	X		X			
Pediatric Quality of Life Cancer Module (Varni et al., 2002)		X				
Oxford Ankle Foot Questionnaire for Children (Morris et al., 2010)		X				
Pain Catastrophizing Scale for Children (Crombez et al., 2003)	X		X			
Self-Assessment of Role Performance and ADLs (Watkins, Lounsbury & Fleming-Castaldy, 2016)	X					X
Functional Disability Inventory (Walker & Greene, 1991)	X		X			
Parent Fear of Pain Questionnaire (Simons, LE, Smith, Kaczynski & Basch 2015)	X		X			
Scoliosis Research Society-7 Questionnaire (Almarani et al., 2021)		X				
Youth Acute Pain Functional Ability Questionnaire (Zempsky et al., 2014)	X					
Sickle Cell Disease Pain Burden Interview for Youth (Zempsky et al., 2013)		X				

Pain Stages of Change Questionnaire for Adolescents (Lorig et al., 2014)	X		X			
Fear of Pain Questionnaire – Child Report (MacNeil & Rainwater, 1998)	X		X			
Pain Reactivity Scale (Wicksell, Olsson & Hayes, 2011)	X		X			
Batten’s Observational Pain Scale (Breau, Camfield & Camfield, 2010)	X					
Pain-Related Parent Behavior Inventory (Hermann, Zohsel, Hohmesiter & Flor, 2008)			X			
The Inventory of Parent Responses to Child’s Pain (Huguet, Miro & Nieto, 2008)	X		X			
Pain Expression Scale (Kuttner & LePage, 1989)	X		X			
Pain-Related Cognitions Questionnaire for Children (Hermann et al., 2007)	X		X			
Child Self-Efficacy Scale (Bandura, 2006)	X		X			
Adolescent Duke Health Profile (Guillemin et al., 1997)	X					
Pain Response Inventory for Children (Walker et al, 1997)	X		X			
Waldron/ Varni Pedatric Pain Coping Inventory (Varni et al., 1996)	X		X			
Children’s Somatization Inventory (Walker & Greene, 1989)	X		X			
Abu-Saad Pediatric Pain Assessment Tool (Abu-Saad, Kroonen & Halfens, 1990)			X			
Observational Scale of Behavioral Distress (Elliott, Jay & Woody, 1987)	X		X			
International Pain Outcomes Questionnaire (Rothaug et al., 2013)	X					
Quality of Life Instrument for Chronic Diseases (Megari, 2013)	X					

The Children's Hospital of Eastern Ontario Pain Scale (Crellin et al., 2007)			X			
Ways of Coping Questionnaire (Folkman & Lazarus, 1988)	X		X			
Child Pain Scale (Gauvin-Piquard et al., 1987)			X			
Symptom Experience Index (Fu, McDaniel & Rhodes, 2007)	X					
Functional Status II Revised (Stein & Jessop, 1990)	X					
Migraine Specific Quality of Life Questionnaire (Jhingran et al., 1988)		X				
Arthritis Impact Measurement Scales (Meenan, Gertman & Mason, 1980)			X			
Clinical Outcome Variables Scale (Seaby & Torrance, 1989)	X		X			
Childhood Health Assessment Questionnaire (Singh et al., 1994)	X					
MOS 36-Item Short Form Health Survey (Ware JE & Sherbourne, 1992)	X					
Child Activities of Daily Living Scale (Fries et al., 1980)	X					
Menstrual Distress Questionnaire (Moos, 1968)		X				
Karnofsky Performance Status (Karnofsky & Burchenal, 1949)	X					
Brief Symptom Inventory (Derogatis & Melisaratos, 1983)	X					
Abdominal Pain Measure (Walker et al., 1997)		X				
Adolescent Pediatric Pain Tool (Savedra et al., 1992)			X			
Pain Response Inventory (Walker et al., 1997)	X		X			
Pain Beliefs Questionnaire (Edwards & Pearce, 1994)	X		X			
Health Utilities Index (Torrance et al., 1996)	X					
Headache Questionnaire (Raieli et al., 1995)		X				

McGill Pain Questionnaire (Melzack, 1975)			X			
Child Reported Pain Measure (Williamson, Walters & Shaffer, 2002)			X			
Headache Impact Questionnaire (Steward et al., 1998)		X				
Pain Experience History (Hester & Barcus, 1986)						X
Pain Related Control Scale (Flor & Turk, 1988)	X		X			
Pain Related Self-Statements Scale (Flor, Behle & Birbaumer, 1993)	X		X			
Nottingham Health Profile (Hunt et al., 1980)	X					
Roland Morris Disability Questionnaire (Roland & Morris, 1983)		X				

Excluded Tool References

Jensen MP, Turner JA, Romano JM & Strom SE. The Chronic Pain Coping Inventory: Development and preliminary validation. *Pain.* (1995). 60:2; 2013-216.

Slater MA, Hall HF, Hampton Atkinson J & Garfin SR. Pain and impairment beliefs in chronic low back pain: validation of the Pain and Impairment Relationship Scale (PAIRS). *Pain.* (1991). 51-56.

McCracken LM & Yang SY. The role of values in a contextual cognitive-behavioral approach to chronic pain. Pain. (2006). *Pain.* 123, 137-145.

Vaalamo I, Pulkkinen L, Kinnunen T, Kaprio J & Rose RJ. Interactive effects of internalizing and externalizing problem behaviors on recurrent pain in children. *Journal of Pediatric Psychology*. (2002). 27; 245-257.

Varni JW, Wilcox KT, Hanson V & Birk R. Chronic musculoskeletal pain and functional status in juvenile rheumatoid arthritis: An empirical model. *Pain*. (1988). 32; 1-7.

Guite JW, Kim S, Chen CP, Sherker JL, Sherry DD, Rose JB & Hwang WT. Treatment expectations among adolescents with chronic musculoskeletal pain and their parents before an initial pain clinic evaluation. *The Clinical Journal of Pain.* (2014). 30:1; 17-26.

Gil KM, Abrams MR, Phillips G & Williams DA. Sickle cell disease pain: Predicting health care use and activity level at 9-month follow-up. *Journal of Consulting Clinical Psychology.* (1992). 60; 267-273.

Powers SW, Mitchell MJ, Graumlich SE, Byars KC, Kalinyak KA. Longitudinal assessment of pain, coping, and daily functioning in children with sickle cell disease receiving pain management skills training. *Journal of Clinical Psychology in Medical Settings.* (2002). 9; 109-119.

Heaton RK, Getto CJ, Lehman RA, Fordyce WE, Brauer E & Groban SE. Standardized evaluation of psychosocial factors in chronic pain. *Pain.* (1982). 12; 165-174.

Mason VL, Skevington SM & Osborn M. Development of a Pain and Discomfort Module for use with the WHOQOL-100. *Quality of Life Research.* (2004). 13; 1139-1152.

Lester N, Lefebvre JC & Keefe FJ. Pain in young adults –III: Relationships of three pain-coping measures of pain and activity interference. (1996). *Clinical Journal of Pain.* 12; 291-300.

Smith BH, Penny KI, Purves A, Munro M, Wilson C, Grimshaw B, Chambers J, Smith WA. The Chronic Pain Grade questionnaire: Validation and reliability in postal research. (1997). *Pain.* 71; 141-147.

Birch S & Jamison RN. Controlled trial of Japanese acupuncture for chronic myofascial neck pain: Assessment of specific and nonspecific effects of treatment. *Clinical Journal of Pain*. (1998). 14; 248-255.
Corson JA & Schneider MJ. The Dartmouth Pain Questionnaire: An adjunct to the McGill Pain Questionnaire. *Pain.* (1984). 19:1; 59-69.

Daut, RL, Cleeland CS, Flanery RC. Development of the Wisconsin Brief Pain Questionnaire to assess pain in cancer and other diseases. *Pain.* (1983). 17; 197-210.

Strong V, Walters R, Hibberd C, Murray G, Wall L, Walker J, McHugh G, Walker A & Sharpe M. Management of depression for people with cancer (SMaRT oncology 1): A raondomised trial. *Lancet.* (2008). 372; 40-48.

Ware JE, Snow KK, Konsinski M et al. SF-36 Health Survey: Manual and Interpretation Guide. Boston, MA: The Health Institute, New England Medical Centre; 1993.

Kleinke CL. How chronic pain patients cope with pain: Relation to treatment outcome in a multidisciplinary pain clinic. Cognitive Therapy and Research. (1992). *Cognitive Therapy and Research*. 16:6; 669-685.

Varni JW, Burwinkle TM, Katz ER, Meeske K & Dickinson P. The PedsQL in pediatric cancer: Reliability and validity of the Pediatric Quality of Life Inventory Generic Core Scales, Multidimensional Fatigue Scale, and Cancer Module. *Cancer.* (2002). 94; 2090-2106.

Morris C, Doll H, Wainwright A, Davies N, Theologis T, Fitzpatrick R. The Oxford Ankle Foot Questionnaire for Children: Review of Development and Potential Applications. *Prosthetics and Orthotics International.* (2020). 34:3; 238-244.

Crombez G, Bijttebier P, Eccleston C, Mascagni T, Mertens G, Goubert L, Verstraeten K. The child version of the pain catastrophizing scale (PCS-C): a preliminary validation. *Pain.* (2003). 104:3; 639-646.

Watkins SL, Lounsbury PA & Fleming-Castaldy RP. The Self-Assessment of Role-Performance and Activities of Daily Living Abilities (SARA): Development and clinical utility of a client-centered screening tool and outcome measure. *Occupational Therapy Health Care.* (2016). 30:1; 42-57.

Walker LS & Greene JW. The Functional Disability Inventory: Measuring a neglected dimension of child health status. *Journal of Pediatric Psychology.* (1991). 16:1; 39-58.

Simons LE, Smith A, Kaczynski K & Basch M. Living in fear of your child's pain. Pain. (2015). 156:4; 694-702.

Alamrani S, Gardner A, Falla D, Russell E, Rushton AB, Heneghan NR. Content validity of Scoliosis Research Society Questionnaire – 22 revised (SRS-22r) for adolescents with idiopathic scoliosis: protocol for a qualitative study exploring patient's and practitioner's perspectives. *BMJ Open*. (2021). 11: e053911; 1-6.

Zempsky WT, O'Hara EA, Santanelli JP, New T, Smith-Whitley K, Casella J & Palermo TM. Development and validation of the Youth Acute Pain Functional Ability Questionnaire (YAPFAQ). *Journal of Pain.* (2014). 15:12; 1319-1327.

Zempsky WT, O'Hara EA, Santanelli JP, Palermo TM, New T, Smith-Whitley K, Casella JF. Validation of the sickle cell disease pain burden interview – youth. *Journal of Pain.* (2013). 14:9, 975-982.

Lorig K, Chastain RL, Ung E, Shoor S & Holman HR. Development and evaluation of a scale to measure perceived self-efficacy in people with arthritis. *Arthritis and Rheumatism.* (1989). 32:1; 37-44.

MacNeil DW & Rainwater AJ. Development of the Fear of Pain Questionnaire – III. *Journal of Behavioral Medicine.* (1998). 21:4; 389-410.

Wicksell RK, Olsson GL & Hayes SC. Mediators of change in acceptance and commitment therapy for pediatric chronic pain. Pain. (2011). 152:12; 2792-2801.

Breau L, Camfield C & Camfield P. Development and initial validation of the Batten's Observational Pain Scale: A preliminary study. *Journal of Pain Management.* (2010). 3:3; 283-292.

Hermann, C, Zohsel K, Hohmeister J & Flor H. Dimensions of pain—related parent behavior: development and psychometric evaluation of a new measure for children and their parents. *Pain.* (2008). 31:137; 689-699.

Huguet A, Miro J & Nieto R. The Inventory of Parent/ Caregiver Responses to the Children's Pain Experience (IRPEDNA): Development and preliminary validation. *Pain.* (2008). 134:1-2; 128-139.

Kuttner L & LePage T. Faces scales for the assessment of pediatric pain: A critical review. *Canadian Journal of Behavioral Science.* (1989). 21; 198-209.

Hermann C, Hohmeister J, Zohsel K, Ebinger F & Flor H. The assessment of pain coping and pain-related cognitions in children and adolsecents: current methods and further development. *Journal of Pain.* (2007). 8:10; 802-813.

Bandura A. Guide for constructing self-efficacy scales. Self-Efficacy Believes of Adolescents. (2006). 5; 307-337.

Guillemin F, Paul-Dauphin A, Virion JM, Bouchet C, Briancon S. The Duke Health Profile: a generic instrument to measure the quality of life tied to health. *Sante Publique.* (1997). 9; 35-44.

Walker LS, Smith CA, Garber J & Van Slyke DA. Development and validation of the Pain Response Inventory for Children. *Psychological Assessment.* (1997). 9:4; 392-405.

Varni JW, Waldron SA, Gragg RA, Rapoff MA, Bernstein BH, Lindsley CB & Newcomb MD. Development of the Waldron/ Varni Pediatric Pain Coping Inventory. *Pain.* 67; 141-150.

Walker LS & Greene JW. Children with recurrent abdominal pain and their parents: More somatic complaints, anxiety, and depression than other patient families. (1989). *Journal of Pediatric Psychology*. 14; 231-243.

Abu-Saad HH, Kroonen E & Halfens R. On the development of a multidimensional Dutch pain assessment tool for children. Pain. (1990). 43; 249-256.

Elliott CH, Jay SM & Woody P. An observational scale for measuring children's distress during medical procedures. (1987). *Journal of Pediatric Psychology.* 12; 543-551.

Rothaug J, Zaslansky R, Schwenkglenks M, Komann M, Allvin R, Backstrom R, Brill S, Buchholz I, Engel C, Fletcher D et al. Patients' perception of postoperative pain management: Validation of the International Pain Outcomes (IPO) questionnaire. *Journal of Pain.* (2013). 14; 1361-70.

Megari K. Quality of life in chronic disease patients. *Health Psychology Research.* (2013). 1:3; e27.

Crellin D, Sullivan TP, Babl FE, O'Sullivan R & Hutchinson A. Analysis of the validation of existing behavioral pain and distress scales for use in the procedural setting. *Journal of Pediatric Anesthesia.* (2007). 17; 720-733.

Folkman S & Lazarus RS. Coping as a mediator of emotion. *Journal of Personality and Social Psychology.* (1988). 54:3; 466-475.

Gauvaiin-Piquard A, Rodary C, Rezvani A & Lemerie J. Pain in children aged 2-6 years: A new observational rating scale elaborated in a pediatric oncology unit: Preliminary report. *Pain.* (1987). 31; 177-188.

Fu MR, McDaniel RW & Rhodes VA. Measuring symptom occurrence and symptom distress: Development of the Symptom Experience Index. *Journal of Advanced Nursing.* (2007). 59:6; 623-634.

Stein REK & Jessop DJ. Functional Status II Revised: A measure of child health status. *Medical Care. (*1990). 28; 1041-1055.

Jhingran P, Osterhaus JT, Miller DW, Lee JT, Kirchdoerfer L. Development and validation of the Migraine-Specific Quality of Life Questionnaire. *Headache.* (1998). 38; 295-302.

Meenan RF, Gertman PM & Mason JH. Measuring health status in arthritis: The Arthritis Impact Measurement Scales. *Arthritis and Rheumatism.* (1980). 23:2; 146-152.

Seaby L & Torrance G. Reliability of a physiotherapy functional assessment in a rehabilitation setting. *Physiotherapy Canada.* (1989). 41; 264-271.

Singh G, Athreya BH, Fries JF & Goldsmith DP. Measurement of health status in children with juvenile rheumatoid arthritis. *Arthritis and Rheumatism.* (1994). 37:12; 1761-1769.

Ware JE & Sherbourne CD. The MOS 36-Item Short Form Health Survey (SF-36): Conceptual framework and item selection. *Medical Care.* (1992). 30:6; 473-483.

Fries JF, Spitz P, Kraines RG & Holman HR. Measurement of patient outcome in arthritis. *Arthritis and Rheumatism.* (1980). 23, 137-145.

Moos RH. The development of a Menstrual Distress Questionnaire. *Psychosomatic Medicine.* (1968). 30:6; 853-867.

Karnofsky DA & Burchenal JH. The clinical evaluation of chemotherapy agents in cancer. In C.M. MacLeod (Ed.), Evaluation of chemotherapeutic agents (pp. 191-205). New York, NY: Columbia University Press.

Derogatis LR & Melisaratos N. The Brief Symptom Inventory: An introductory report. *Psychological Medicine.* (1983). 13; 595-605.

Walker LS, Smith CA, Garber J & Van Slyke DA. Development and validation of the Pain Response Inventory for Children. *Psychological Assessment.* (1997). 9; 392-405.

Savedra MC, Tesler MD, Holzemer WL & Ward JA. Adolescent Pediatric Pain Tool (APPT). (1992). San Francisco: University of California, School of Nursing.

Walker LS, Smith CA, Garber J & Van Slyke DA. Development and validation of the Pain Response Inventory for children. *Psychological Assessment.* (1997). 9:4; 392-405.

Edwards LC & Pearce SA. Word completion in chronic pain: Evidence for schematic representation of pain? *Journal of Abnormal Psychology*. (1994). 103; 379-382.

Torrance GW, Feeny DH, Furlong WJ, Barr RD, Zhang Y & Wang Q. Multiattribute utility function for a comprehensive health status classification system: Health Utilities Index Mark 2. *Medical Care*. (1996). 34:7; 702-722.

Raieli V, Raimondo D, Cammalleri R & Camarda R. Migraine headaches in adolescents: A student population-based study in Monreale. *Cephalagia.* (1995). 15; 5-12.

Melzack R. The McGill Pain Questionnaire: Major properties and scoring methods. *Pain.* 1:3; 277-299.

Williamson GM, Walters AS & Shaffer DR. Caregiver models of self and others, coping and depression: Predictors of depression in children with chronic pain. Health Psychology. (2002). 21; 405-410.

Stewart WF, Lipton RB, Simon D, Von Korff M & Liberman J. Reliability of an illness severity measure for headache in a population sample of migraine sufferers. *Cephalalgia.* (1998). 18; 44-51.

Hester NP & Barcus CS. Assessment and management of pain in children. *Pediatrics.* (1986). 1; 1-8.

Flor H & Turk DC. Chronic back pain and rheumatoid arthritis: Predicting pain and disability from cognitive variables. *Journal of Behavioral Medicine.* (1988). 11:3; 251-265.

Flor H, Behle D & Birbaumer N. Assessment of pain-related cognitions in chronic pain patients. *Behavior Research and Therapy.* (1993). 31; 63-67.

Hunt SM, McKenna SP, McEwen J, Backett EM, Williams J & Papp E. A quantitative approach to perceived health status: A validation study. *Journal of Epidemiology and Community Health.* (1980). 34:4; 281-286.

Roland M & Morris R. A study of the natural history of back pain. Part 1: Development of a reliable and sensitive measure of disability in low-back pain. *Spine*. (1983). 8:2; 141-144.

Supplementary File 5 – Reasons for Citation Exclusion

Tool 1: The Bath Adolescent Pain Questionnaire (n=140 citations excluded)

Citation Excluded	Reason for Exclusion
Double-blind, placebo-controlled randomized trial with adalimumab for treatment of juvenile onset ankylosing spondylitis (JoAS): Significant short term improvement<br>Horneff, G; Fitter, S; Foeldvari, I; Minden, K; Kuemmerle-Deschner, J; Tzaribacev, N; Thon, A; Borte, M; Ganser, G; Trauzeddel, R; Huppertz, H.-I.<br>Arthritis Research and Therapy // 2012;14(5):<br>General Pediatrics, Asklepios Clinics, Arnold Janssen Str. 29, Sankt Augustin, 53757, Germany 2012 //<br>DOI: 10.1186/ar4072	Does not utilize or assess tool
Clinical aspects of itch in adult atopic dermatitis patients<br>Chrostowska-Plak, D; Salomon, J; Reich, A; Szepietowski, J C<br>Acta Dermato-Venereologica // 2009;89(4):379-383<br>Department of Dermatology, Venereology and Allergology, Wroclaw Medical University, Wroclaw, Poland 2009 //<br>DOI: 10.2340/00015555-0676	Does not utilize or assess tool<br>Not pediatric population<br>Not chronic pain population
Psychological effects of chronic pain: An overview<br>McCracken, L M<br>Clinical Pain Management: Chronic Pain, Second Edition // 2008;():169-177<br>2008 //	Cannot locate full text<br>Not an original study
Patients with chronic pain exhibit a complex relationship triad between pain, resilience, and within- and cross-network functional connectivity of the default mode network<br>Hemington, K S; Rogachov, A; Cheng, J C; Bosma, R L; Kim, J A; Osborne, N R; Inman, R D; Davis, K D<br>Pain // 2018;159(8):1621-1630<br>Institute of Medical Science, University of Toronto, Toronto, ON, Canada Lippincott Williams and Wilkins 2018 //<br>DOI: 10.1097/j.pain.0000000000001252	Does not utilize or assess tool
The prevalence, impact and cost of chronic non-cancer pain in Irish primary schoolchildren (PRIME-C): Protocol for a longitudinal school-based survey<br>O'Higgins, S; Doherty, E; NicGabhainn, S; Murphy, A; Hogan, M; O'Neill, C; McGuire, B E<br>BMJ Open // 2015;5(5):<br>Centre for Pain Research, School of Psychology, National University of Ireland, Galway, Ireland BMJ Publishing Group 2015 //<br>DOI: 10.1136/bmjopen-2014-007426	Not original study
Pediatric Pain Measurement, Assessment, and Evaluation	Not an original study

Manworren, R C B; Stinson, J<br>Seminars in Pediatric Neurology // 2016;23(3):189-200<br>Acute Pain Management Program, Ann & Robert H. Lurie Children's Hospital of Chicago, Northwestern University, Chicago, IL, United States W.B. Saunders 2016 //<br>DOI: 10.1016/j.spen.2016.10.001	
Validation of a self-report questionnaire version of the Child Activity Limitations Interview (CALI): The CALI-21<br>Palermo, T M; Lewandowski, A S; Long, A C; Burant, C J<br>Pain // 2008;139(3):644-652<br>Department of Anesthesiology and Peri-Operative Medicine, Oregon Health and Science University, 3181 SW Sam Jackson Park Road, Portland, OR 97239-3098, United States 2008 //<br>DOI: 10.1016/j.pain.2008.06.022	Does not utilize or assess tool
Chronic idiopathic pain syndromes<br>Sen, D; Christie, D<br>Best Practice and Research: Clinical Rheumatology // 2006;20(2):369-386<br>Department of Rheumatology, Great Ormond Street Hospital NHS Trust, 40-50 Tottenham Street, London W1T 4NJ, United Kingdom 2006 //<br>DOI: 10.1016/j.berh.2005.11.009	Not an original study
The impact of chronic pain in children and adolescents: Development and initial validation of a child and parent version of the Pain Experience Questionnaire<br>Hermann, Christiane; Hohmeister, Johanna; Zohsel, Katrin; Tuttas, Marie-Luise; Flor, Herta<br>;():<br>DOI: 10.1016/j.pain.2007.06.002	Does not utilize or assess tool
Development and Preliminary Validation of the Child Pain Anxiety Symptoms Scale in a Community Sample<br>Page, M Gabrielle; Fuss, Samantha; Martin, Andrea L; Escobar, E Manolo Romero; Katz, Joel<br>JOURNAL OF PEDIATRIC PSYCHOLOGY // 2010;35(10):1071-1082<br>JOURNALS DEPT, 2001 EVANS RD, CARY, NC 27513 USA OXFORD UNIV PRESS INC 2010 //<br>DOI: 10.1093/jpepsy/jsq034	Does not utilize or assess tool
Psychological and nonpsychological interventions for chronic pediatric pain<br>Hermann, C<br>Pain 2012 Refresher Courses: 14th World Congress on Pain // 2015;():	Cannot locate full text

Department of Clinical Psychology and Psychotherapy, Justus-Liebig University, Otto-Behaghel-Str. 10F, Giessen, D-35394, Germany Wolters Kluwer Health Adis (ESP) 2015 //	
Improvement in patient-reported outcomes for patients with ankylosing spondylitis treated with etanercept 50 mg once-weekly and 25 mg twice-weekly<br>Braun, J; McHugh, N; Singh, A; Wajdula, J S; Sato, R<br>Rheumatology // 2007;46(6):999-1004<br>Rheumatology Medical Center, Ruhrgebeit, Ruhr-University, Bochum, Germany 2007 //<br>DOI: 10.1093/rheumatology/kem069	Does not utilize or assess tool
Quality of life and related variables in patients with ankylosing spondylitis.<br>Bodur, H; Ataman, S; Rezvani, A; Buğdayci, D S; Cevik, R; Birtane, M; Akinci, A; Altay, Z; Günaydin, R; Yener, M; Koçyiğit, H; Duruöz, T; Yazgan, P; Cakar, E; Aydin, G; Hepgüler, S; Altan, L; Kirnap, M; Olmez, N; Soydemir, R; Kozanoğlu, E; Bal, A; Sivrioğlu, K; Karkucak, M; Günendi, Z<br>Quality of life research : an international journal of quality of life aspects of treatment, care and rehabilitation // 2011;20(4):543-549<br>Ankara Numune Training & Research Hospital, Mürsel Uluç M, 937.S, 35/17, 06450, Ankara, Turkey. 2011 //<br>DOI: 10.1007/s11136-010-9771-9	Does not utilize or assess tool
Perceived trigger factors of seizures in persons with epilepsy<br>Balamurugan, E; Aggarwal, M; Lamba, A; Dang, N; Tripathi, M<br>Seizure // 2013;22(9):743-747<br>All India Institute of Medical Sciences, College of Nursing, India 2013 //<br>DOI: 10.1016/j.seizure.2013.05.018	Does not utilize or assess tool<br>Not chronic pain population
Measuring Pain in Adolescents<br>Ameringer, S<br>Journal of Pediatric Health Care // 2009;23(3):201-204<br>2009 //<br>DOI: 10.1016/j.pedhc.2009.01.006	Not an original study
Personal Hygiene and Vulvovaginitis in Prepubertal Children<br>Cemek, F; Odabaş, D; Şenel, U; Kocaman, A T<br>Journal of Pediatric and Adolescent Gynecology // 2016;29(3):223-227<br>Department of Pediatrics, Basaksehir State Hospital, Istanbul, Turkey Elsevier USA 2016 //<br>DOI: 10.1016/j.jpag.2015.07.002	Does not utilize or assess tool
A case of childhood-onset ankylosing spondylitis: Diagnosis and treatment	Does not utilize or assess tool

Burgos-Vargas, R<br>Nature Clinical Practice Rheumatology // 2009;5(1):52-57<br>Rheumatology Department of Hospital General de México, Faculty of Medicine, Universidad Nacional Autónoma de México, Mexico City, Mexico 2009 //<br>DOI: 10.1038/ncprheum0971	
Deep brain stimulation as a treatment for neuropathic pain: A longitudinal study addressing neuropsychological outcomes<br>Gray, A M; Pounds-Cornish, E; Eccles, F J R; Aziz, T Z; Green, A L; Scott, R B<br>Journal of Pain // 2014;15(3):283-292<br>Headwise Ltd, Innovation Centre, Longbridge Technology Park, Birmingham, United Kingdom 2014 //<br>DOI: 10.1016/j.jpain.2013.11.003	Does not utilize or assess tool
Spondyloarthritis Research Consortium of Canada (SPARCC) enthesitis index in turkish patients with ankylosing spondylitis: Relationship with disease activity and quality of life<br>Zahiroglu, Y; Ulus, Y; Akyol, Y; Tander, B; Durmus, D; Bilgici, A; Kuru, O<br>International Journal of Rheumatic Diseases // 2014;17(2):173-180<br>Department of Physical Medicine and Rehabilitation, Medical Faculty, Ondokuz Mayis University, Samsun, Turkey 2014 //<br>DOI: 10.1111/1756-185X.12067	Does not utilize or assess tool
Pain in children: Recent advances and ongoing challenges<br>Walker, S M<br>British Journal of Anaesthesia // 2008;101(1):101-110<br>Portex Department of Anaesthesia, UCL Institute of Child Health, Great Ormond Street Hospital NHS Trust, 30 Guilford Street, London WC1N 1EH, United Kingdom Oxford University Press 2008 //<br>DOI: 10.1093/bja/aen097	Not an original study
Development and psychometric evaluation of The Bath Adolescent Pain Questionnaire (BAPQ)<br>Jordan, A; Eccleston, C; McCracken, L; Sleed, M; Connell, H; Sourbut, C; Clinch, J<br>ANNALS OF THE RHEUMATIC DISEASES 07// 2005;64(3):75-75<br>BRITISH MED ASSOC HOUSE, TAVISTOCK SQUARE, LONDON WC1H 9JR, ENGLAND BMJ PUBLISHING GROUP 2005 07//	Cannot find full text
Epidemiological assessment of Schistosoma haematobium-induced kidney and bladder pathology in rural Zimbabwe<br>Brouwer, K C; Ndhlovu, P D; Wagatsuma, Y; Munatsi, A; Shiff, C J<br>Acta Tropica // 2003;85(3):339-347<br>W. Harry Feinstone Dept. Molec. M., Bloomberg School of Public Health, Johns Hopkins University, 615 N. Wolfe Street, Baltimore, MD 21205, United States 2003 //	Does not utilize or assess tool

DOI: 10.1016/S0001-706X(02)00262-0	
Reliability and validity of the Brazilian-Portuguese version of the Burns Specific Pain Anxiety Scale (BSPAS)<br>Echevarria-Guanilo, M E; Dantas, R A S; Farina, J A; Faber, A W; Alonso, J; Rajmil, L; Rossi, L A<br>International Journal of Nursing Studies // 2011;48(1):47-55<br>Escola de Enfermagem de Ribeirão Preto da Universidade de São Paulo, Brazil 2011 //<br>DOI: 10.1016/j.ijnurstu.2010.05.015	Does not utilize or assess tool
Men with pelvic pain: Perceived helpfulness of medical and self-management strategies<br>Turner, J A; Ciol, M A; Korff, M V; Liu, Y.-W.; Berger, R<br>Clinical Journal of Pain // 2006;22(1):19-24<br>Department of Psychiatry and Behavioral Sciences, University of Washington School of Medicine, Seattle, WA, United States 2006 //<br>DOI: 10.1097/01.ajp.0000148630.15369.79	Does not utilize or assess tool<br>Not a pediatric population
The relationship between disease activity measured by the BASDAI and psychological status, stressful life events, and sleep quality in ankylosing spondylitis<br>Jiang, Y; Yang, M; Wu, H; Song, H; Zhan, F; Liu, S; Gao, G; Liu, Z; Hu, Z; He, P; Zhang, S; Lin, Z; Zhang, Y; Li, Y; Shen, L; Huang, A; Liao, Z; Cao, S; Wei, Y; Li, L; Li, Q; Lv, Q; Qi, J; Huang, J; Li, T; Jin, O; Pan, Y; Gu, J<br>Clinical Rheumatology // 2015;34(3):503-510<br>Department of Rheumatology and Immunology, The Third Affiliated Hospital of Sun Yat-sen University, 600 Tianhe Road, Guangzhou, 510630, China Springer London 2015 //<br>DOI: 10.1007/s10067-014-2688-x	Does not utilize or assess tool
The relationship between disease activity measured by the BASDAI and psychological status, stressful life events, and sleep quality in ankylosing spondylitis<br>Jiang, Y; Yang, M; Wu, H; Song, H; Zhan, F; Liu, S; Gao, G; Liu, Z; Hu, Z; He, P; Zhang, S; Lin, Z; Zhang, Y; Li, Y; Shen, L; Huang, A; Liao, Z; Cao, S; Wei, Y; Li, L; Li, Q; Lv, Q; Qi, J; Huang, J; Li, T; Jin, O; Pan, Y; Gu, J<br>Clinical Rheumatology // 2015;34(3):503-510<br>Department of Rheumatology and Immunology, The Third Affiliated Hospital of Sun Yat-sen University, 600 Tianhe Road, Guangzhou, 510630, China Springer London 2015 //<br>DOI: 10.1007/s10067-014-2688-x	Does not utilize or assess tool
The educational needs of patients with undifferentiated spondyloarthritis: Validation of the ENAT questionnaire and needs assessment<br>Bremander, A; Haglund, E; Bergman, S; Ndosi, M<br>Musculoskeletal Care // 2018;16(2):313-317	Does not utilize or assess tool

Department of Clinical Sciences, Section of Rheumatology, Lund University, Lund, Sweden John Wiley and Sons Ltd 2018 //<br>DOI: 10.1002/msc.1231	
The treatment experience questionnaire: Development and validation of a questionnaire assessing the individual's emotional, perceptual, and cognitive reactions to alternative, physical, and dental treatments<br>Blasche, G; Marktl, W; Eisenwort, B; Skolka, A; Pichlhöfer, O<br>Forschende Komplementarmedizin // 2013;20(3):205-212<br>Department of Environmental Hygiene, Center for Public Health, Medical University of Vienna, Kinderspitalgasse 15, 1090 Vienna, Austria 2013 //<br>DOI: 10.1159/000351456	Does not utilize or assess tool
Effect of age at disease onset in the clinical profile of spondyloarthritis: A study of 1424 Brazilian patients<br>Skare, T L; Leite, N; Bortoluzzo, A B; Gonçalves, C R; da Silva, J A B; Ximenes, A C; Bértolo, M B; Ribeiro, S L E; Keiserman, M; Menin, R; Carneiro, S; Azevedo, V F; Vieira, W P; Albuquerque, E N; Bianchi, W A; Bonfiglioli, R; Campanholo, C; Carvalho, H M S; Costa, I P; Duarte, A P; Gavi, M B O; Kohem, C L; Lima, S A L; Meirelles, E S; Pereira, I A; Pinheiro, M M; Polito, E; Resende, G G; Rocha, F A C; Santiago, M B; Sauma, M.D.F.L.C.; Sampaio-Barros, P D<br>Clinical and Experimental Rheumatology // 2012;30(3):351-357<br>Hospital Evangélico de Curitiba, Curitiba, Brazil 2012 //	Does not utilize or assess tool
Assessing knowledge, attitudes, and behavior of adolescent girls in suburban districts of Tehran about dysmenorrhea and menstrual hygiene<br>Poureslami, M; Osati-Ashtiani, F<br>Journal of International Women's Studies // 2002;3(2):1-11<br>Iran University of Medical Sciences, School of Medicine, Department of Community and Preventive Medicine, Tehran, Iran 2002 //	Does not utilize or assess tool
Evaluation of the effects of Global Postural Reeducation in patients with ankylosing spondylitis<br>Silva, E M; Andrade, S C; Vilar, M J<br>Rheumatology International // 2012;32(7):2155-2163<br>Universidade Federal do Rio Grande do Norte (UFRN), Natal, Brazil 2012 //<br>DOI: 10.1007/s00296-011-1938-3	Does not utilize or assess tool
Diet, disease activity, and gastrointestinal symptoms in patients with ankylosing spondylitis<br>Sundström, B; Wållberg-Jonsson, S; Johansson, G<br>Clinical Rheumatology // 2011;30(1):71-76<br>Department of Public Health and Clinical Medicine, Division of Rheumatology, Umeå University, Umeå, Sweden 2011 //<br>DOI: 10.1007/s10067-010-1625-x	Does not utilize or assess tool
Prevalence and factors associated with disturbed sleep in outpatients with ankylosing spondylitis	Does not utilize or assess tool

Nie, A; Wang, C; Song, Y; Xie, X; Yang, H; Chen, H<br>Clinical Rheumatology // 2018;37(8):2161-2168<br>West China School of Nursing and Department of Nursing, West China Hospital, Sichuan University, Chengdu, Sichuan Province, China Springer London 2018 //<br>DOI: 10.1007/s10067-018-4190-3	
Culture, bathing and hydrotherapy in labor: An exploratory descriptive pilot study<br>Benfield, R; Heitkemper, M M; Newton, E R<br>Midwifery // 2018;64():110-114<br>College of Nursing, Health Sciences Building, East Carolina University, Greenville, NC 27834, United States Churchill Livingstone 2018 //<br>DOI: 10.1016/j.midw.2018.06.005	Does not utilize or assess tool<br>Not pediatric population<br>Not chronic pain population
Knowledge on postnatal care among postpartum mothers during discharge in maternity hospitals in Asmara: A cross-sectional study<br>Beraki, G G; Tesfamariam, E H; Gebremichael, A; Yohannes, B; Haile, K; Tewelde, S; Goitom, S<br>BMC Pregnancy and Childbirth // 2020;20(1):<br>Department of Nursing, Orotta College of Medicine and Health Sciences, Asmara, Eritrea BioMed Central Ltd. 2020 //<br>DOI: 10.1186/s12884-019-2694-8	Does not utilize or assess tool<br>Not pediatric population<br>Not chronic pain population
Integrating preferences into health status assessment for amyotrophic lateral sclerosis: The ALS Utility Index<br>Beusterein, K; Leigh, N; Jackson, C; Miller, R; Mayo, K; Revicki, D<br>Amyotrophic Lateral Sclerosis and Other Motor Neuron Disorders // 2005;6(3):169-176<br>MEDTAP International, 7101 Wisconsin Avenue, Bethesda, MD 20814, United States 2005 //<br>DOI: 10.1080/14660820510021339	Does not utilize or assess tool
A new approach to defining functional ability in ankylosing spondylitis: The development of the bath ankylosing spondylitis functional index<br>Calin, A; Garrett, S; Whitelock, H; O'Hea, J; Mallorie, P; Jenkinson, T<br>Journal of Rheumatology // 1994;21(12):2281-2285<br>RNHRD, Upper Borough Walls, Bath BA1 1RL, United Kingdom 1994 //	Does not utilize or assess tool
Prevalence and risk factors of contact dermatitis among clothing manufacturing employees in Beijing: A cross-sectional study<br>Chen, Y.-X.; Cheng, H.-Y.; Li, L.-F.<br>Medicine (United States) // 2017;96(12):<br>Department of Dermatology, Beijing Friendship Hospital, Capital Medical University, 95 Yong An Road, Xicheng District, Beijing, 100050, China Lippincott Williams and Wilkins 2017 //	Does not utilize or assess tool<br>Not chronic pain population

Is the Children's Depression Inventory Short version a valid screening tool in pediatric care? A comparison to its full-length version<br>Allgaier, A.-K.; Frühe, B; Pietsch, K; Saravo, B; Baethmann, M; Schulte-Körne, G<br>Journal of Psychosomatic Research // 2012;73(5):369-374<br>Department of Child and Adolescent Psychiatry, Psychosomatics and Psychotherapy, Ludwig-Maximilians-University Munich, Germany 2012 //<br>DOI: 10.1016/j.jpsychores.2012.08.016	Does not utilize or assess tool
Parental catastrophizing about their child's pain. The parent version of the Pain Catastrophizing Scale (PCS-P): A preliminary validation<br>Goubert, L; Eccleston, C; Vervoort, T; Jordan, A; Crombez, G<br>Pain // 2006;123(3):254-263<br>Department of Experimental-Clinical and Health Psychology, Ghent University, Belgium 2006 //<br>DOI: 10.1016/j.pain.2006.02.035	Does not utilize or assess tool
Assessment of pain anxiety, pain catastrophizing, and fear of pain in children and adolescents with chronic pain: A systematic review and meta-analysis<br>Fisher, E; Heathcote, L C; Eccleston, C; Simons, L E; Palermo, T M<br>Journal of Pediatric Psychology // 2018;43(3):314-325<br>Center for Child Health, Behavior, and Development, Seattle Children's Research Institute, Seattle, WA, United States Oxford University Press 2018 //<br>DOI: 10.1093/jpepsy/jsx103	Not an original study
Effectiveness of saltwater baths in the treatment of epidermolysis bullosa<br>Petersen, B W; Arbuckle, H A; Berman, S<br>Pediatric Dermatology // 2015;32(1):60-63<br>Department of Dermatology, Warren Alpert Medical School, Brown University, 216 Howell Street, Apt #1, Providence, RI 80203, United States Blackwell Publishing Inc. 2015 //<br>DOI: 10.1111/pde.12409	Does not utilize or assess tool<br>Not chronic pain population
Diffuse noxious inhibitory control function in women with provoked vestibulodynia<br>Sutton, K S; Pukall, C F; Chamberlain, S<br>Clinical Journal of Pain // 2012;28(8):667-674<br>Department of Psychology, Queen's University, Kingston General Hospital, 62 Arch Street, Kingston, ON, K7L 3N6, Canada 2012 //<br>DOI: 10.1097/AJP.0b013e318243ede4	Does not utilize or assess tool<br>Not chronic pain population<br>Not pediatric population

Global consensus from clinicians regarding low back pain outcome indicators for older adults: Pairwise wiki survey using crowdsourcing<br>Wong, A Y L; Lauridsen, H H; Samartzis, D; Macedo, L; Ferreira, P H; Ferreira, M L<br>Journal of Medical Internet Research // 2019;21(1):<br>Department of Sports Science and Clinical Biomechanics, University of Southern Denmark, Odense, Denmark Journal of Medical Internet Research 2019 //<br>DOI: 10.2196/11127	Does not utilize or assess tool<br>Not pediatric population
Gender and disease features in Moroccan patients with ankylosing spondylitis<br>Ibn Yacoub, Y; Amine, B; Laatiris, A; Hajjaj-Hassouni, N<br>Clinical Rheumatology // 2012;31(2):293-297<br>Department of Rheumatology (Pr N. Hajjaj-Hassouni), University Hospital of Rabat-Sale, El Ayachi Hospital, Sale 11000, Morocco 2012 //<br>DOI: 10.1007/s10067-011-1819-x	Does not utilize or assess tool
The Severity of Chronic Pediatric Pain: An Epidemiological Study<br>Huguet, A; Miró, J<br>Journal of Pain // 2008;9(3):226-236<br>Department of Psychology, Rovira i Virgili University, Catalonia, Spain 2008 //<br>DOI: 10.1016/j.jpain.2007.10.015	Does not utilize or assess tool
Evaluation of Psychometric and Linguistic Properties of the Italian Adolescent Pain Assessment Scales: A Systematic Review<br>Marti, Flavio; Paladini, Antonella; Varrassi, Giustino; Latina, Roberto<br>PAIN AND THERAPY 06// 2018;7(1):77-104<br>GEWERBESTRASSE 11, CHAM, CH-6330, SWITZERLAND SPRINGER INTERNATIONAL PUBLISHING AG 2018 06//<br>DOI: 10.1007/s40122-018-0093-x	Not an original study
Biomarkers, imaging and disease activity indices in patients with early axial spondyloarthritis: The Italian arm of the SpondyloArthritis-Caught-Early (SPACE) Study<br>Lorenzin, M; Ortolan, A; Vio, S; Favero, M; Oliviero, F; Zaninotto, M; Cosma, C; Lacognata, C; Punzi, L; Ramonda, R<br>Reumatismo // 2017;69(2):65-74<br>Rheumatology Unit, Department of Medicine - DIMED, University of Padova, Italy Page Press Publications 2017 //<br>DOI: 10.4081/reumatismo.2017.977	Does not utilize or assess tool
Systematic review of family functioning in families of children and adolescents with chronic pain<br>Lewandowski, A S; Palermo, T M; Stinson, J; Handley, S; Chambers, C T<br>Journal of Pain // 2010;11(11):1027-1038	Not an original study

Oregon Health and Science University, Portland, OR, United States 2010 // DOI: 10.1016/j.jpain.2010.04.005	
Quality of life in patients with ankylosing spondylitis: Relationships with spinal mobility, disease activity and functional status<br>Özdemir, O<br>Rheumatology International // 2011;31(5):605-610<br>Department of Physical Medicine and Rehabilitation, Ataturk Training and Research Hospital, Ankara, Turkey 2011 //<br>DOI: 10.1007/s00296-009-1328-2	Does not utilize or assess tool
Clinical and ultrasonographic enthesopathy in inflammatory rheumatic diseases: Is MASEI or only calcaneal enthesitis sufficient?<br>Süleyman, E; Nas, K; Harman, H; Kaban, N<br>Zeitschrift fur Rheumatologie // 2018;77(8):719-726<br>Faculty of Medicine, Division of Rheumatology, Department of Physical Medicine and Rehabilitation, Sakarya University, Sakarya, 54100, Turkey Dr. Dietrich Steinkopff Verlag GmbH and Co. KG 2018 //<br>DOI: 10.1007/s00393-017-0405-2	Did not utilize or assess tool
A simplified version of Ankylosing Spondylitis Disease Activity Score (ASDAS) in patients with ankylosing spondylitis<br>Sommerfleck, F A; Schneeberger, E E; Buschiazzo, E E; Maldonado Cocco, J A; Citera, G<br>Clinical Rheumatology // 2012;31(11):1599-1603<br>Section of Rheumatology, Instituto de Rehabilitación Psicofísica, Echeverría 955, 1428 Buenos Aires, Argentina 2012 //<br>DOI: 10.1007/s10067-012-2056-7	Did not utilize or assess tool
Assessment of fatigue in patients with ankylosing spondylitis<br>Turan, Y; Duruöz, M T; Bal, S; Guvenc, A; Cerrahoglu, L; Gurgan, A<br>Rheumatology International // 2007;27(9):847-852<br>Department of Physical Medicine and Rehabilitation, Atatürk Research and Education Hospital, Izmir, Turkey 2007 //<br>DOI: 10.1007/s00296-007-0313-x	Did not utilize or assess tool
Characterization of pain in patients with Barth syndrome<br>Taylor, D; Brady, J E; Li, G; Sonty, N; Saroyan, J M<br>Children's Health Care // 2016;45(2):192-203<br>Department of Palliative Medicine, Memorial Sloan Kettering Cancer Center, New York, NY, United States Routledge 2016 //<br>DOI: 10.1080/02739615.2014.996882	Did not utilize or assess tool

Symptomatic dermographism: An inadequately described disease<br>Schoepke, N; Młynek, A; Weller, K; Church, M K; Maurer, M<br>Journal of the European Academy of Dermatology and Venereology // 2015;29(4):708-712<br>Department of Dermatology and Allergy, Allergie-Centrum-Charité Charité - Universitätsmedizin, Berlin, Germany Blackwell Publishing Ltd 2015 //<br>DOI: 10.1111/jdv.12661	Did not utilize or assess tool
Attitude and knowledge of medical students of Isra University about dysmenorrhoea and its treatment.<br>Parveen, N; Majeed, R; Zehra, N; Rajar, U; Munir, A A<br>Journal of Ayub Medical College, Abbottabad : JAMC // 2009;21(3):159-162<br>Department of Obstetrics & Gynaecology, Liaquat University of Medical and Health Sciences, Jamshoro, Pakistan. 2009 //	Did not utilize or assess tool
Sleep in ankylosing spondylitis and non-radiographic axial spondyloarthritis: associations with disease activity, gender and mood<br>Wadeley, A; Clarke, E; Leverment, S; Sengupta, R<br>Clinical Rheumatology // 2018;37(4):1045-1052<br>College of Liberal Arts: Culture and Environment, Bath Spa University, Bath, BA9 2BN, United Kingdom Springer London 2018 //<br>DOI: 10.1007/s10067-018-3984-7	Did not utilize or assess tool
A hot topic for health: Results of the Global Sauna Survey<br>Hussain, J N; Greaves, R F; Cohen, M M<br>Complementary Therapies in Medicine // 2019;44():223-234<br>School of Health & Biomedical Sciences, RMIT University – Bundoora Campus, Melbourne, 3083, Australia Churchill Livingstone 2019 //<br>DOI: 10.1016/j.ctim.2019.03.012	Did not utilize or assess tool
Effects of aerobic training in patients with ankylosing spondylitis<br>Jennings, F; Oliveira, H A; De Souza, M C; Cruz, V D G; Natour, J<br>Journal of Rheumatology // 2015;42(12):2347-2353<br>Rheumatology Division, Universidade Federal de Sao Paulo, Brazil Journal of Rheumatology 2015 //<br>DOI: 10.3899/jrheum.150518	Did not utilize or assess tool
Work productivity in a population-based cohort of patients with spondyloarthritis<br>Haglund, E; Bremander, A; Bergman, S; Jacobsson, L T H; Petersson, I F<br>Rheumatology (United Kingdom) // 2013;52(9):1708-1714<br>Spenshult Research and Development Center, Spenshult, Oskarström, Sweden 2013 //<br>DOI: 10.1093/rheumatology/ket217	Did not utilize or assess tool

A new approach to defining disease status in ankylosing spondylitis: The bath ankylosing spondylitis disease activity index<br>Garrett, S; Jenkinson, T; Kennedy, L G; Whitelock, H; Gaisford, P; Calin, A<br>Journal of Rheumatology // 1994;21(12):2286-2291<br>RNHRD, Upper Borough Walls, Bath BA1 1RL, United Kingdom 1994 //	Did not utilize or assess tool
Yoga for youth in pain: The UCLA pediatric pain program model<br>Evans, S; Moieni, M; Sternlieb, B; Tsao, J C I; Zeltzer, L K<br>Holistic Nursing Practice // 2012;26(5):262-271<br>Pediatric Pain Program, David Geffen School of Medicine, University of California, 10833 Le Conte Ave, Los Angeles, CA 90095, United States 2012 //<br>DOI: 10.1097/HNP.0b013e318263f2ed	Did not utilize or assess tool
Functional impairment in spondyloarthropathy and fibromyalgia<br>Heikkilä, S; Ronni, S; Kautiainen, H J; Kauppi, M J<br>Journal of Rheumatology // 2002;29(7):1415-1419<br>Vasaratie 22 D 36, FIN-37100 Tampere, Finland 2002 //	Did not utilize or assess tool
Chronic pain assessment tools for cerebral palsy: A systematic review<br>Kingsnorth, S; Orava, T; Provvidenza, C; Adler, E; Ami, N; Gresley-Jones, T; Mankad, D; Slonim, N; Fay, L; Joachimides, N; Hoffman, A; Hung, R; Fehlings, D<br>Pediatrics // 2015;136(4):e947-e960<br>Evidence to Care, Holland Bloorview Kids Rehabilitation Hospital, 150 Kilgour Rd, Toronto, ON M4G 1R8, Canada American Academy of Pediatrics 2015 //<br>DOI: 10.1542/peds.2015-0273	Not an original study
Attitudes of female adolescents about dysmenorrhea and menstrual hygiene in Tehran suburbs<br>Poureslami, M; Osati-Ashtiani, F<br>Archives of Iranian Medicine // 2002;5(4):219-224<br>Dept. of Community/Preventive Med., Iran University of Medical Sciences, West Abrar St., South Sohrevardi Ave, Tehran 15796, Iran 2002 //	Does not utilize or assess tool
Patient-reported outcome in psoriatic arthritis: A comparison of web-based versus paper-completed questionnaires<br>MacKenzie, H; Thavaneswaran, A; Chandran, V; Gladman, D D<br>Journal of Rheumatology // 2011;38(12):2619-2624<br>Psoriatic Arthritis Program, Toronto Western Hospital, University of Toronto, 399 Bathurst St., 1E410B, Toronto, ON M5T 2S8, Canada 2011 //<br>DOI: 10.3899/jrheum.110165	Does not utilize or assess tool

Validity and reliability study of the Turkish version of Spinal Cord Independence Measure-III<br>Unalan, H; Misirlioglu, T O; Erhan, B; Akyuz, M; Gunduz, B; Irgi, E; Arslan, H E; Baltaci, A; Aslan, S; Palamar, D; Kutlu, A; Majlesi, J; Akarirmak, U; Karamehmetoglu, S S<br>Spinal Cord // 2015;53(6):455-460<br>Department of Physical Medicine and Rehabilitation, Istanbul University, Cerrahpasa Medical Faculty, Istanbul, Turkey Nature Publishing Group 2015 //<br>DOI: 10.1038/sc.2014.249	Does not utilize or assess tool
Accelerating the drug delivery pipeline for acute and chronic pancreatitis-knowledge gaps and research opportunities: Overview summary of a National Institute of Diabetes and Digestive and Kidney Diseases workshop<br>Uc, A; Andersen, D K; Borowitz, D; Glesby, M J; Mayerle, J; Sutton, R; Pandol, S J<br>Pancreas // 2018;47(10):1180-1184<br>2018 //<br>DOI: 10.1097/MPA.0000000000001176	Does not utilize or assess tool<br>Not chronic pain population
Enthesitis and its relationships with disease parameters in Moroccan patients with ankylosing spondylitis<br>Laatiris, A; Amine, B; Yacoub, Y I; Hajjaj-Hassouni, N<br>Rheumatology International // 2012;32(3):723-727<br>Department of Rheumatology, Ayachi Hospital, University Hospital of Rabat-Sale, 11000 Sale, Morocco 2012 //<br>DOI: 10.1007/s00296-010-1658-0	Does not utilize or assess tool
Effects of Multidisciplinary Team-Based Nurse-led Transitional Care on Clinical Outcomes and Quality of Life in Patients With Ankylosing Spondylitis<br>Liang, L; Pan, Y; Wu, D; Pang, Y; Xie, Y; Fang, H<br>Asian Nursing Research // 2019;13(2):107-114<br>Department of Nursing, The Third Affiliated Hospital of Sun Yat-sen University, Guangzhou, China Korean Society of Nursing Science 2019 //<br>DOI: 10.1016/j.anr.2019.02.004	Does not utilize or assess tool
Evaluation of the Turkish version of the Bath Ankylosing Spondylitis Patient Global Score (BAS-G)<br>Ozer, H T E; Sarpel, T; Gulek, B; Alparslan, Z N; Erken, E<br>Clinical Rheumatology // 2006;25(2):136-139<br>Department of Medicine, Rheumatology-Immunology Division, Cukurova University, Balcali, Adana 01330, Turkey 2006 //<br>DOI: 10.1007/s10067-005-1129-2	Does not utilize or assess tool

Sleep disturbances are associated with increased pain, disease activity, depression, and anxiety in ankylosing spondylitis: A case-control study<br>Li, Y; Zhang, S; Zhu, J; Du, X; Huang, F<br>Arthritis Research and Therapy // 2012;14(5):<br>Department of Rheumatology, Chinese PLA General Hospital, 28 Fuxing Road, Beijing 100853, China 2012 //<br>DOI: 10.1186/ar4054	Does not utilize or assess tool
Evaluating the reliability of Persian version of ankylosing spondylitis quality of life (ASQoL) questionnaire and related clinical and demographic parameters in patients with ankylosing spondylitis<br>Fallahi, S; Jamshidi, A R; Bidad, K; Qorbani, M; Mahmoudi, M<br>Rheumatology International // 2014;34(6):803-809<br>Internal Medicine Division, Baharloo Hospital, Tehran University of Medical Sciences, Behdari Street, South Kargar Street, 1339973111 Tehran, Iran Springer Verlag 2014 //<br>DOI: 10.1007/s00296-013-2888-8	Does not utilize or assess tool
Itch characteristics in Chinese patients with atopic dermatitis using a new questionnaire for the assessment of pruritus<br>Yosipovitch, G; Goon, A T J; Wee, J; Chan, Y H; Zucker, I; Goh, C L<br>International Journal of Dermatology // 2002;41(4):212-216<br>National Skin Center, 1 Mandalay Road, Singapore 308205, Singapore 2002 //<br>DOI: 10.1046/j.1365-4362.2002.01460.x	Does not utilize or assess tool
Validity and reliability of the Turkish version of the Health Assessment Questionnaire for the Spondyloarthropathies<br>Ozcan, E; Yilmaz, O; Tutoglu, A; Bodur, H<br>Rheumatology International // 2012;32(6):1563-1568<br>Department of Physical Medicine and Rehabilitation, Midyat Government Hospital, Mardin, Turkey 2012 //<br>DOI: 10.1007/s00296-011-1795-0	Does not utilize or assess tool
Chronic musculoskeletal pain in children: Assessment and management<br>Clinch, J; Eccleston, C<br>Rheumatology // 2009;48(5):466-474<br>Bath Centre for Pain Services, Royal National Hospital for Rheumatic Diseases, Upper Borough Walls, Bath BA1 4RL, United Kingdom 2009 //<br>DOI: 10.1093/rheumatology/kep001	Not an original study
Evaluation of Treatments for Pruritus in Epidermolysis Bullosa<br>Danial, C; Adeduntan, R; Gorell, E S; Lucky, A W; Paller, A S; Bruckner, A L; Pope, E; Morel, K D; Levy, M L; Li, S; Gilmore, E S; Lane, A T	Does not utilize or assess tool

Pediatric Dermatology // 2015;32(5):628-634<br>Department of Dermatology, School of Medicine, Stanford University, 700 Welch Road, Palo Alto, CA 94304, United States<br>Blackwell Publishing Inc. 2015 //<br>DOI: 10.1111/pde.12486	
Disabilities, access to medical care, and way of life of adults with cerebral palsy. APIB study: first results<br>Dauvergne, F; Eon, Y; Gallien, P; Bouric, S; Duruflé-Tapin, A; Cambla, N; Nicolas, B<br>Annales de Readaptation et de Medecine Physique // 2007;50(1):20-27<br>Réseau Breizh IMC, centre MPR Notre-Dame-de-Lourdes, 54, rue Saint-Hélier, 35000 Rennes, France 2007 //<br>DOI: 10.1016/j.annrmp.2006.06.008	Does not utilize or assess tool<br>Not pediatric population
Paediatric yellow flags and early identification of psychosocial factors in paediatric patients with unexplained musculoskeletal disorders<br>Ciara, C; Susan, H; Joanne, Q<br>Physiotherapy Practice and Research // 2011;32(1):19-23<br>Physiotherapy Department, Our Lady's Children's Hospital, Crumlin, Dublin, Ireland 2011 //<br>DOI: 10.3233/PPR-2011-32104	Cannot locate full text
Adolescent chronic pain and disability: A review of the current evidence in assessment and treatment<br>Eccleston, C; Clinch, J<br>Paediatrics and Child Health // 2007;12(2):117-120<br>Bath Pain Management Unit, Royal National Hospital for Rheumatic Diseases NHS Trust, Bath, United Kingdom Pulsus Group Inc. 2007 //<br>DOI: 10.1093/pch/12.2.117	Not an original study
Pain management<br>Dhal, A; Mehta, M; Sagar, R<br>A Practical Approach to Cognitive Behaviour Therapy for Adolescents // 2015;():179-189<br>Global Health Strategies Emerging Economies Pvt. LtdNew Delhi, India Springer India 2015 //<br>DOI: 10.1007/978-81-322-2241-5_9	Not an original study
The mechanisms of pain tolerance and pain-related anxiety in acute pain<br>Cimpean, A; David, D<br>Health Psychology Open // 2019;6(2):<br>Doctoral School Evidence-Based Assesment and Psychological Interventions, Babes-Bolyai University, Romania SAGE Publications Inc. 2019 //<br>DOI: 10.1177/2055102919865161	Does not utilize or assess tool
Development and Validation of the Youth Acute Pain Functional Ability Questionnaire (YAPFAQ)	Does not utilize or assess tool

Zempsky, William T; O'Hara, Emily A; Santanelli, James P; New, Tamara; Smith-Whitley, Kim; Casella, James; Palermo, Tonya M<br>Journal of Pain // 2014;15(12):1319-1327<br>2014 //<br>DOI: 10.1016/j.jpain.2014.09.008	
Measurement and Assessment of Pain in Pediatric Patients<br>Stinson, J N; McGrath, P J<br>Clinical Pain Management: A Practical Guide // 2010;():64-71<br>Child Health Evaluative Sciences, Department of Anesthesia and Pain Medicine, The Hospital for Sick Children, Toronto, Canada Wiley-Blackwell 2010 //<br>DOI: 10.1002/9781444329711.ch8	Not an original study
The assessment and management of chronic and recurrent pain in adolescents<br>Walco, G A; Rozelman, H; Maroof, D A<br>Behavioral Approaches to Chronic Disease in Adolescence: A Guide to Integrative Care // 2009;():163-175<br>David Center for Children's Pain and Palliative Care, Hackensack University Medical Center, Hackensack, NJ, United States Springer New York 2009 //<br>DOI: 10.1007/978-0-387-87687-0_14	Not an original study
Distinct influences of anxiety and pain catastrophizing on functional outcomes in children and adolescents with chronic pain<br>Tran, S T; Jastrowski Mano, K E; Hainsworth, K R; Medrano, G R; Khan, K A; Weisman, S J; Davies, W H<br>Journal of Pediatric Psychology // 2015;40(8):744-755<br>Division of Behavioral Medicine and Clinical Psychology, Cincinnati Children's Hospital Medical Center, 3333 Burnet Ave. MLC #3015, Cincinnati, OH 45229, United States Oxford University Press 2015 //<br>DOI: 10.1093/jpepsy/jsv029	Does not utilize or assess tool
Classifying the severity of paediatric chronic pain - An application of the chronic pain grading<br>Wager, J; Hechler, T; Darlington, A S; Hirschfeld, G; Vocks, S; Zernikow, B<br>European Journal of Pain (United Kingdom) // 2013;17(9):1393-1402<br>German Paediatric Pain Centre, Children's and Adolescents' Hospital, Datteln, Germany 2013 //<br>DOI: 10.1002/j.1532-2149.2013.00314.x	Does not utilize or assess tool
Daily changes in pain, mood and physical function in youth hospitalized for sickle cell disease pain<br>Zempsky, W T; Palermo, T M; Corsi, J M; Lewandowski, A S; Zhou, C; Casella, J F<br>Pain Research and Management // 2013;18(1):33-38<br>Connecticut Children's Medical Center, 282 Washington Street, Hartford, CT 06106, United States Hindawi Limited 2013 //	Does not utilize or assess tool

DOI: 10.1155/2013/487060	
Assessing back pain: Does the Oswestry disability questionnaire accurately measure function in ankylosing spondylitis?<br>O'Shea, F D; Riarh, R; Annepa, A; Inman, R D<br>Journal of Rheumatology // 2010;37(6):1211-1213<br>Division of Rheumatology, Toronto Western Hospital, Toronto Western Research Institute, Toronto, ON, Canada 2010 //<br>DOI: 10.3899/jrheum.091240	Does not utilize or assess tool
Efficacy and safety of ultrasound-guided local injections of etanercept into entheses of ankylosing spondylitis patients with refractory Achilles enthesitis<br>Huang, Z; Cao, J; Li, T; Zheng, B; Wang, M; Zheng, R<br>Clinical and Experimental Rheumatology // 2011;29(4):642-649<br>Department of Rheumatology, Third Affiliated Hospital of Sun Yat-Sen University, Guangzhou, China 2011 //	Does not utilize or assess tool
Efficacy and safety of etanercept in patients with the enthesitis-related arthritis category of juvenile idiopathic arthritis: Results from a phase III randomized, double-blind study<br>Horneff, G; Foeldvari, I; Minden, K; Trauzeddel, R; Kümmerle-Deschner, J B; Tenbrock, K; Ganser, G; Huppertz, H.-I.<br>Arthritis and Rheumatology // 2015;67(8):2240-2249<br>Asklepios Clinic Sankt Augustin, Department of General Pediatrics and Adolescent Medicine-astr-temp Arnold Janssen Street 29, Sankt Augustin, 53757, Germany John Wiley and Sons Inc. 2015 //<br>DOI: 10.1002/art.39145	Does not utilize or assess tool
The assessment of disability in children and adolescents with headache: Adopting PedMIDAS in an epidemiological study<br>Kröner-Herwig, B; Heinrich, M; Vath, N<br>European Journal of Pain // 2010;14(9):951-958<br>Georg-August-University Göttingen, Georg-Elias-Müller-Institute of Psychology, Dept. of Clinical Psychology and Psychotherapy, Gosslerstr. 14, 37073 Göttingen, Germany 2010 //<br>DOI: 10.1016/j.ejpain.2010.02.010	Does not utilize or assess tool
Validation of the Sickle Cell Disease Pain Burden Interview-Youth<br>Zempsky, William T; O'hara, Emily A; Santanelli, James P; Palermo, Tonya M; New, Tamara; Smith-Whitley, Kim; Casella, James F<br>// 2013;():<br>2013 //<br>DOI: 10.1016/j.jpain.2013.03.007	Does not utilize or assess tool
Paediatric chronic pain	Not an original study

Rolfe, P M<br>Anaesthesia and Intensive Care Medicine // 2019;20(10):539-542<br>NHS Foundation Trust, United Kingdom Elsevier Ltd 2019 //<br>DOI: 10.1016/j.mpaic.2019.07.010	
Effectiveness of interdisciplinary interventions in paediatric chronic pain management: a systematic review and subset meta-analysis<br>Liossi, C; Johnstone, L; Lilley, S; Caes, L; Williams, G; Schoth, D E<br>British Journal of Anaesthesia // 2019;123(2):e359-e371<br>University of Southampton, School of Psychology, Southampton, United Kingdom Elsevier Ltd 2019 //<br>DOI: 10.1016/j.bja.2019.01.024	Not an original study
Attitude, behaviour and knowledge regarding menarche and menstruation in adolescent schoolgirls in Kayseri<br>Şenol, V; Gündüz, E; Öztürk, A<br>Turkiye Klinikleri Jinekoloji Obstetrik // 2010;20(2):77-83<br>Halil Bayraktar Health Services Vocational College, University of Erciyes, Kayseri, Turkey 2010 //	Does not utilize or assess tool
Models of Care for addressing chronic musculoskeletal pain and health in children and adolescents<br>Stinson, J; Connelly, M; Kamper, S J; Herlin, T; Toupin April, K<br>Best Practice and Research: Clinical Rheumatology // 2016;30(3):468-482<br>The Hospital for Sick Children, Lawrence S. Bloomberg, Faculty of Nursing, University of Toronto, Peter Gilgan Centre for Research and Learning, 686 Bay Street, Room 069715, Toronto, ON M5G 0A4, Canada Bailliere Tindall Ltd 2016 //<br>DOI: 10.1016/j.berh.2016.08.005	Not an original study
Adaptation of Problem-Solving Skills Training (PSST) for parent caregivers of youth with chronic pain<br>Palermo, T M; Law, E F; Essner, B; Jessen-Fiddick, T; Eccleston, C<br>Clinical Practice in Pediatric Psychology // 2014;2(3):212-223<br>Department of Anesthesiology and Pain Medicine, University of Washington, Seattle Children's Hospital Research Institute, M/S CW8-6, Seattle, WA 98145, United States American Psychological Association Inc. 2014 //<br>DOI: 10.1037/cpp0000067	Not an original study
Pain and somatoform disorders<br>Palermo, T M; Krell, H; Janosy, N; Zeltzer, L K<br>Developmental-Behavioral Pediatrics: Evidence and Practice // 2008;():711-741<br>Department of Anesthesiology and Peri-Operative Medicine, Oregon Health and Science University, Portland, OR, United States Elsevier Inc. 2008 //<br>DOI: 10.1016/B978-0-323-04025-9.50024-6	Does not utilize or assess tool

Managing childhood fever and pain - The comfort loop<br>Clinch, J; Dale, S<br>Child and Adolescent Psychiatry and Mental Health // 2007;1():<br>Pain Management Unit, Southmead Hospital, Bristol, United Kingdom 2007 //<br>DOI: 10.1186/1753-2000-1-7	Does not utilize or assess tool
Latest developments in the assessment and management of chronic musculoskeletal pain syndromes in children<br>Connelly, M; Schanberg, L<br>Current Opinion in Rheumatology // 2006;18(5):496-502<br>Duke University Medical Center, Durham, NC, United States 2006 //<br>DOI: 10.1097/01.bor.0000240361.32089.97	Does not utilize or assess tool
Psychiatric symptoms in ankylosing spondylitis: their relationship with disease activity, functional capacity, pain and fatigue<br>Durmus, D; Sarisoy, G; Alayli, G; Kesmen, H; Çetin, E; Bilgici, A; Kuru, O; Ünal, M<br>Comprehensive Psychiatry // 2015;62():170-177<br>Department of Physical Medicine and Rehabilitation, Medical Faculty, Ondokuz Mayis University, Samsun, Turkey W.B. Saunders 2015 //<br>DOI: 10.1016/j.comppsych.2015.07.016	Does not utilize or assess tool
Pain in children<br>Cucchiaro, G<br>Biobehavioral Approaches to Pain // 2009;():149-194<br>Department of Anesthesia and Critical Care Medicine, Children's Hospital of Philadelphia, 34th. St and Civic Center Blvd., Philadelphia, PA 19104, United States Springer New York 2009 //<br>DOI: 10.1007/978-0-387-78323-9_8	Does not utilize or assess tool
Body mass in adolescents with chronic pain: Observational study<br>Gauntlett-Gilbert, J; Bhat, C; Clinch, J<br>Archives of Disease in Childhood // 2019;():<br>Bath Centre for Pain Services, Royal United Hospital Bath NHS Trust, Bath, BA1 3NG, United Kingdom BMJ Publishing Group 2019 //<br>DOI: 10.1136/archdischild-2019-317843	Does not include at least one of: Tool Development or Psychometric evaluation
Measuring musculoskeletal pain in infants, children, and adolescents<br>Michaleff, Z A; Kamper, S J; Stinson, J N; Hestbaek, L; Williams, C M; Campbell, P; Dunn, K M<br>Journal of Orthopaedic and Sports Physical Therapy // 2017;47(10):712-730	Not an original study

2017 //<br>DOI: 10.2519/jospt.2017.7469	
Wading pool water contaminated with both noroviruses and astroviruses as the source of a gastroenteritis outbreak<br>Maunula, L; Kalso, S; Von Bonsdorff, C.-H.; Pönkä, A<br>Epidemiology and Infection // 2004;132(4):737-743<br>HUCH Laboratory Diagnostics, Division of Virology, Haartmaninkatu 3, 00290 Helsinki, Finland 2004 //<br>DOI: 10.1017/S0950268804002249	Does not utilize or assess tool<br>Not chronic pain population
Quality of life and correlation with clinical and radiographic variables in patients with ankylosing spondylitis: A retrospective case series study<br>Huang, J.-C.; Qian, B.-P.; Qiu, Y; Wang, B; Yu, Y; Zhu, Z.-Z.; Hu, J; Qu, Z<br>BMC Musculoskeletal Disorders // 2017;18(1):<br>Department of Spine Surgery, Drum Tower Hospital, Affiliated Hospital of Nanjing, University Medical School, Zhongshan Road 321, Nanjing, 210008, China BioMed Central Ltd. 2017 //<br>DOI: 10.1186/s12891-017-1711-1	Does not utilize or assess tool
Physical and occupational therapy outcomes: Adolescents' change in functional abilities using objective measures and self-report<br>Kempert, H; Benore, E; Heines, R<br>Scandinavian Journal of Pain // 2017;14():60-66<br>Cleveland Clinic Children's Hospital for Rehabilitation, Therapy Services Department, United States Elsevier B.V. 2017 //<br>DOI: 10.1016/j.sjpain.2016.10.004	Does not utilize or assess tool
Validity and reliability of the Health Assessment Questionnaire among patients with spondyloarthritis in Singapore<br>Kwan, Y H; Fong, W; Lui, N L; Yong, S T; Cheung, Y B; Malhotra, R; Thumboo, J; Østbye, T<br>International Journal of Rheumatic Diseases // 2018;21(3):699-704<br>Program in Health Systems and Services Research, Duke-NUS Medical School, Singapore, Singapore Blackwell Publishing 2018 //<br>DOI: 10.1111/1756-185X.12989	Does not utilize or assess tool
The treatment of pain in neonatal and pediatric patients<br>Houck, C S; Tobias, J D; Tresgallo, M E; Anand, K J S; Schechter, W S<br>Cousins and Bridenbaugh's Neural Blockade in Clinical Anesthesia and Pain Medicine: Fourth Edition // 2012;():<br>Department of Anaesthesia, Harvard Medical School, Boston, MA, United States Wolters Kluwer Health Adis (ESP) 2012 //	Cannot locate full text

Clinical utility and validity of the Functional Disability Inventory among a multicenter sample of youth with chronic pain<br>Kashikar-Zuck, S; Flowers, S R; Claar, R L; Guite, J W; Logan, D E; Lynch-Jordan, A M; Palermo, T M; Wilson, A C<br>Pain // 2011;152(7):1600-1607<br>Division of Behavioral Medicine and Clinical Psychology, Cincinnati Children's Hospital Medical Center, University of Cincinnati College of Medicine, Cincinnati, OH, United States 2011 //<br>DOI: 10.1016/j.pain.2011.02.050	Does not utilize or assess tool
Social desirability response bias and self-report of psychological distress in pediatric chronic pain patients<br>Logan, D E; Claar, R L; Scharff, L<br>Pain // 2008;136(3):366-372<br>Children's Hospital Boston, Harvard Medical School, Pain Treatment Service, 333 Longwood Avenue, Boston, MA 02115, United States 2008 //<br>DOI: 10.1016/j.pain.2007.07.015	Does not utilize or assess tool
Adolescent-parent relationships in the context of adolescent chronic pain conditions<br>Logan, D E; Guite, J W; Sherry, D D; Rose, J B<br>Clinical Journal of Pain // 2006;22(6):576-583<br>Children's Hospital Boston, United States 2006 //<br>DOI: 10.1097/01.ajp.0000210900.83096.ca	Does not utilize or assess tool
Parent perceptions of adolescent pain expression: The adolescent pain behavior questionnaire<br>Lynch-Jordan, A M; Kashikar-Zuck, S; Goldschneider, K R<br>Pain // 2010;151(3):834-842<br>Department of Pediatrics, University of Cincinnati, Cincinnati Children's Hospital, Cincinnati, OH, United States 2010 //<br>DOI: 10.1016/j.pain.2010.09.025	Does not utilize or assess tool
Prevalence and perception of schistosomiasis in a periurban school area of Bamako in Mali<br>Sangho, H; Dabo, A; Coulibaly, H; Doumbo, O<br>Bulletin de la Societe de Pathologie Exotique // 2002;95(4):292-294<br>C. R., E. de D. pour la S. de l'E., BP. 2109, Bamako, Mali 2002 //	Cannot locate full text
Outcome measurement in chronic pain<br>Johnson, T<br>Clinical Pain Management: Chronic Pain, Second Edition // 2008;():178-189<br>Pain Management Manchester and Salford Pain Centre, Hope Hospital, Salford, United Kingdom CRC Press 2008 //	Cannot locate full text
A developmental arrest? Interruption and identity in adolescent chronic pain<br>Jordan, A; Noel, M; Caes, L; Connell, H; Gauntlett-Gilbert, J	Does not utilize or assess tool

Pain Reports // 2018;3(7):<br>Department of Psychology, Centre for Pain Research, University of Bath, Claverton Down, Bath, BA2 7AY, United Kingdom<br>Lippincott Williams and Wilkins 2018 //<br>DOI: 10.1097/PR9.0000000000000678	
A short-term efficacy and safety study of infliximab in active ankylosing spondylitis<br>Huang, F; Zhang, L Y; Zhang, J L; Zhang, F C; Liang, D F; Deng, X H; Guo, J H; Zhu, J; Zhao, W; Li, X F; Hou, Y<br>Zhonghua nei ke za zhi [Chinese journal of internal medicine] // 2006;45(2):122-126<br>Department of Rheumatology, Chinese PLA General Hospital, Beijing, 100853, China 2006 //	Not English or French
Development and psychometric evaluation of the Bath Adolescent Pain Questionnaire (BAPQ)<br>Jordan, A; Eccleston, C; McCracken, L; Connell, H; Clinch, J<br>RHEUMATOLOGY 04// 2006;45(1):I107-I107<br>GREAT CLARENDON ST, OXFORD OX2 6DP, ENGLAND OXFORD UNIV PRESS 2006 04//	Cannot locate full text
Somatoform disorders in childhood and adolescence: Aetiology, assessment and intervention from the perspective of behavioural medicine<br>Noeker, M<br>Zeitschrift fur Medizinische Psychologie // 2012;21(3):100-111<br>Psychologischer Psychotherapeut für Kinder, Jugendliche und Erwachsene, LWL-Dezernent für Krankenhäuser und Gesundheitswesen Landschaftsverband Westfalen-Lippe, Hörsterplatz 2, Münster, Germany 2012 //<br>DOI: 10.3233/ZMP-2012-210012	Not English or French
Pain Amplification Syndrome: A Biopsychosocial Approach<br>Namerow, L B; Kutner, E C; Wakefield, E C; Rzepski, B R; Sahl, R A<br>Seminars in Pediatric Neurology // 2016;23(3):224-230<br>Department of Pediatrics, University of Connecticut School of Medicine, Farmington, CT, United States W.B. Saunders 2016 //<br>DOI: 10.1016/j.spen.2016.10.006	Does not utilize or assess tool
An interactive assessment system for children with chronic pain<br>McCann, J; Wang, H; Zheng, H; Eccleston, C<br>IEEE-EMBS International Conference on Biomedical and Health Informatics, BHI 2012. In Conj. with the 8th Int. Symp.on Medical Devices and Biosensors and the 7th Int. Symp. on Biomedical and Health Engineering // 2012;():926-929<br>School of Computing and Mathematics, University of Ulster, Jordanstown, BT37 0QB, United Kingdom 2012 //<br>DOI: 10.1109/BHI.2012.6211739	Cannot locate full text
Enhancing daily functioning with exposure and acceptance strategies: An important stride in the development of psychological therapies for pediatric chronic pain<br>Palermo, T M<br>Pain // 2009;141(3):189-190	Does not utilize or assess tool

Division of Clinical Pain and Regional Anesthesia Research, Dept. of Anesthesiology and Peri-Operative Medicine, Oregon Health and Science University, 3181 SW Sam Jackson Park Rd., UHN-2, Portland, OR 97239, United States 2009 //<br>DOI: 10.1016/j.pain.2008.12.012	
Characterizing the pain narratives of parents of youth with chronic pain<br>Noel, M; Beals-Erickson, S E; Law, E F; Alberts, N M; Palermo, T M<br>Clinical Journal of Pain // 2016;32(10):849-858<br>Department of Psychology, University of Calgary, 2500 University Dr., N.W., Calgary, AB T2N 1N4, Canada Lippincott Williams and Wilkins 2016 //<br>DOI: 10.1097/AJP.0000000000000346	Does not include at least one of: Tool Development or Psychometric Evaluation
Paediatric chronic pain<br>Rolfe, P M<br>Anaesthesia and Intensive Care Medicine // 2016;17(11):531-535<br>Paediatric Anaesthesia and Pain Medicine, Addenbrooke's Hospital, Cambridge University Hospitals NHS Foundation Trust, United Kingdom Elsevier Ltd 2016 //<br>DOI: 10.1016/j.mpaic.2016.08.009	Not an original study
German Pain Questionnaire for Children, Adolescents and Parents (DSF-KJ): A multimodal questionnaire for diagnosis and treatment of children and adolescents suffering from chronic pain<br>Schroeder, S; Hechler, T; Denecke, H; Müller-Busch, M; Martin, A; Menke, A; Zernikow, B<br>Schmerz // 2010;24(1):23-37<br>Vodafone Stiftungsinstitut und Lehrstuhl fur Kinderschmerztherapie und Padiatrische Palliativmedizin, Vestische Kinder- und Jugendklinik Datteln, Universität Witten/Herdecke, Dr.-Friedrich-Steiner Str. 5, 45711 Datteln, Germany 2010 //<br>DOI: 10.1007/s00482-009-0864-8	Not English or French
Chronic pain problems in children and young people<br>Howard, R F<br>Continuing Education in Anaesthesia, Critical Care and Pain // 2011;11(6):219-223<br>Paediatric Anaesthesia and Pain Medicine, Clinical Lead for Pain Management, Great Ormond Street Hospital for Children, London WC1N 3JHUK, United Kingdom Oxford University Press 2011 //<br>DOI: 10.1093/bjaceaccp/mkr042	Does not utilize or assess tool
Neuroimaging of paediatric pain<br>Hartley, C; Slater, R<br>Neuroimaging of Pain // 2017;():485-506<br>Department of Paediatrics, Level 2 Children's Hospital, John Radcliffe, University of Oxford, Oxford, OX3 9DU, United Kingdom Springer International Publishing 2017 //	Does not utilize or assess tool

DOI: 10.1007/978-3-319-48046-6_18	
The Role of the Mental Health Practitioner in the Assessment and Treatment of Child and Adolescent Chronic Pain<br>Leo, R J; Srinivasan, S P; Parekh, S<br>Child and Adolescent Mental Health // 2011;16(1):2-8<br>Department of Psychiatry, State University of New York at Buffalo, School of Medicine and Biomedical Sciences, Erie County Medical Center, 462 Grider Street, Buffalo, NY 14215, United States 2011 //<br>DOI: 10.1111/j.1475-3588.2010.00578.x	Does not utilize or assess tool
Development of a Functional and Emotional Measure of Dysmenorrhea (FEMD) in Chinese University Women<br>Li, L; Huangfu, L; Chai, H; He, W; Song, H; Zou, X; Wang, W<br>Health Care for Women International // 2012;33(2):97-108<br>Department of Clinical Psychology and Psychiatry, Zhejiang University School of Medicine, Zijingang Campus, Yuhangtang Road 866, Hangzhou, Zhejiang 310058, China 2012 //<br>DOI: 10.1080/07399332.2011.603863	Not pediatric population
Assessment of chronic pain in children: Current status and emerging topics<br>Palermo, T M<br>Pain Research and Management // 2009;14(1):21-26<br>Department of Anesthesiology and Peri-Operative Medicine, Oregon Health and Science University, 3181 SW Sam Jackson Park Road, Portland, OR 97239, United States Hindawi Limited 2009 //<br>DOI: 10.1155/2009/236426	Does not utilize or assess tool
Efficacy of Internet-delivered cognitive-behavioral therapy for the management of chronic pain in children and adolescents: A systematic review and meta-analysis<br>Tang, W.-X.; Zhang, L.-F.; Ai, Y.-Q.; Li, Z.-S.<br>Medicine (United States) // 2018;97(36):<br>Department of Anaesthesiology, First Affiliated Hospital of Zhengzhou University, Longhu Road and Longxiang Seven Street Intersection, Zhengdong New District, Zhengzhou, 450000, China Lippincott Williams and Wilkins 2018	Does not utilize or assess tool
Relationship between sleep quality and nocturnal pain in ankylosing spondylitis<br>Zhang, S.-L.; Li, Y; Zhu, J; Huang, Z.-F.; Zhang, J.-L.; Huang, F<br>National Medical Journal of China // 2013;93(13):970-972<br>Department of Rheumatology, Chinese PLA General Hospital, Beijing 100853, China 2013 //<br>DOI: 10.3760/cma.j.issn.0376-2491.2013.13.004	Not English or French
Efficacy assessment in paediatric studies<br>Wang, S; Laitinen-Parkkonen, P	Not an original study

Handbook of Experimental Pharmacology // 2011;205():149-168<br>Norwegian Medicines Agency, Tønsberg Hospital Pharmacy, Sven Oftedalsvei 6, Oslo N-0950, Norway 2011 //<br>DOI: 10.1007/978-3-642-20195-0_7	
Physical and social functioning in adolescents with rheumatological conditions: A study of predictors<br>Gauntlett-Gilbert, J; Kavirayani, A; Clinch, J<br>Acta Paediatrica, International Journal of Paediatrics // 2013;102(3):e131-e136<br>Bath Centre for Pain Services, Royal National Hospital for Rheumatic Diseases, Bath BA1 1RL, United Kingdom 2013 //<br>DOI: 10.1111/apa.12094	Not exclusive chronic pain population
The Young Disability Questionnaire-Spine: Item development, pilot testing and conceptualisation of a questionnaire to measure consequences of spinal pain in children<br>Meldgaard, Emilie; Lauridsen, Henrik Hein; Hestbaek, Lise<br>BMJ Open 2021;11(5):<br>BMJ Publishing Group 2021<br>DOI: 10.1136/bmjopen-2020-045580	Does not utilize or assess tool
Psychometric Properties of the Abdominal Pain Index (API) in the Iranian Adolescent Population<br>Hoseini, Sepideh; Jafari, Mahdi; Asl Soleimani, Zahra; Qaderi Bagajan, Kaveh; Sadeghi, Meysam; Zolfaghari, Shadi<br>Pain Research and Management 2020;2020():<br>Hindawi Limited 2020<br>DOI: 10.1155/2020/2632139	Does not utilize or assess tool
The Pediatric American Pain Society Patient Outcomes Questionnaire (Pediatric APS-POQ): Development and Initial Psychometric Evaluation of a Brief and Comprehensive Measure of Pain and Pain Outcomes in Hospitalized Youth<br>Kaczynski, Karen; Ely, Elizabeth; Gordon, Debra; Vincent, Catherine; Waddell, Kristi; Wittmayer, Kimberly; Bernhofer, Esther<br>Journal of Pain 2020;21(5-6):633-647<br>Churchill Livingstone Inc. 2020<br>DOI: 10.1016/j.jpain.2019.10.003	Not chronic pain population
Feasibility of a randomized controlled trial of paediatric interdisciplinary pain management using home-based telehealth<br>Hilyard, Anna; Kingsley, Julia; Sommerfield, David; Taylor, Susan; Bear, Natasha; Gibson, Noula<br>Journal of Pain Research 2020;13():897-908<br>Dove Medical Press Ltd. 2020<br>DOI: 10.2147/JPR.S217022	Does not include at least one of: Tool Development or Psychometric Evaluation

Achievement goals, fear of failure and self-handicapping in young elite athletes with and without chronic pain<br>Molenaar, Bodile; Willems, Charlotte; Verbunt, Jeanine; Goossens, Mariëlle<br>Children 2021;8(7):<br>MDPI AG 2021<br>DOI: 10.3390/children8070591	Does not utilize or assess tool
The Bath Adolescent Pain - Parental Impact Questionnaire (BAP-PIQ): Development and preliminary psychometric evaluation of an instrument to assess the impact of parenting an adolescent with chronic pain<br>Jordan, A; Eccleston, C; McCracken, L M; Connell, H; Clinch, J<br>Pain // 2008;137(3):478-487<br>Pain Management Unit, University of Bath, Bath, BA2 7AY, United Kingdom 2008 //<br>DOI: 10.1016/j.pain.2007.10.007	Didn't include measure on children/ youth

Tool 2: PROMIS Pediatric Pain Interference Scale (n=139 excluded citations)

Citation Excluded	Reason for Exclusion
Concurrent validity of the PROMIS® pediatric global health measure<br>Forrest, C B; Tucker, C A; Ravens-Sieberer, U; Pratiwadi, R; Moon, J H; Teneralli, R E; Becker, B; Bevans, K B<br>Quality of Life Research // 2016;25(3):739-751<br>2016 //<br>DOI: 10.1007/s11136-015-1111-7	Did not utilize or asses tool
Psychometric Evaluation of the PROMIS (R) Pediatric Psychological and Physical Stress Experiences Measures<br>Bevans, Katherine B; Gardner, William; Pajer, Kathleen A; Becker, Brandon; Carle, Adam; Tucker, Carole A; Forrest, Christopher B<br>JOURNAL OF PEDIATRIC PSYCHOLOGY 07// 2018;43(6):678-692<br>JOURNALS DEPT, 2001 EVANS RD, CARY, NC 27513 USA OXFORD UNIV PRESS INC 2018 07//<br>DOI: 10.1093/jpepsy/jsy010	Not chronic pain population
From the Child's Word to Clinical Intervention: Novel, New, and Innovative Approaches to Symptoms in Pediatric Palliative Care<br>Brock, Katharine E; Wolfe, Joanne; Ullrich, Christina<br>CHILDREN-BASEL 04// 2018;5(4):<br>ST ALBAN-ANLAGE 66, CH-4052 BASEL, SWITZERLAND MDPI 2018 04//	Did not utilize or assess tool

DOI: 10.3390/children5040045	
Risk and Resilience in Pediatric Pain: The Roles of Parent and Adolescent Catastrophizing and Acceptance<br>Feinstein, Amanda B; Sturgeon, John A; Bhandari, Rashmi P; Yoon, Isabel A; Ross, Alexandra C; Huestis, Samantha E; Griffin, Anya T; Simons, Laura E<br>CLINICAL JOURNAL OF PAIN 12// 2018;34(12):1096-1105<br>TWO COMMERCE SQ, 2001 MARKET ST, PHILADELPHIA, PA 19103 USA LIPPINCOTT WILLIAMS & WILKINS 2018 12//<br>DOI: 10.1097/AJP.0000000000000639	Not original study
Pediatric analgesic clinical trial designs, measures, and extrapolation: Report of an FDA Scientific Workshop<br>Berde, C B; Walco, G A; Krane, E J; Anand, K J S; Aranda, J V; Craig, K D; Dampier, C D; Finkel, J C; Grabois, M; Johnston, C; Lantos, J; Lebel, A; Maxwell, L G; McGrath, P; Oberlander, T F; Schanberg, L E; Stevens, B; Taddio, A; Von Baeyer, C L; Yaster, M; Zempsky, W T<br>Pediatrics // 2012;129(2):354-364<br>2012 //<br>DOI: 10.1542/peds.2010-3591	Did not utilize or assess tool
Pain catastrophizing is associated with poorer health-related quality of life in pediatric patients with sickle cell disease<br>Bakshi, N; Lukombo, I; Belfer, I; Krishnamurti, L<br>Journal of Pain Research // 2018;11():947-953<br>2018 //<br>DOI: 10.2147/JPR.S151198	Did not include at least one of: Tool Development or Psychometric Evaluation
Psychometric evaluation of the ProMISVR pediatric psychological and physical stress experiences measures<br>Bevans, K B; Gardner, W; Pajer, K A; Becker, B; Carle, A; Tucker, C A; Forrest, C B<br>Journal of Pediatric Psychology // 2018;43(6):678-692<br>2018 //<br>DOI: 10.1093/jpepsy/jsy010	Not chronic pain population
The PROMIS initiative: Involvement of rehabilitation stakeholders in development and examples of applications in rehabilitation research<br>Amtmann, D; Cook, K F; Johnson, K L; Cella, D<br>Archives of Physical Medicine and Rehabilitation // 2011;92(10 SUPPL.):S12-S19<br>2011 //	Not pediatric population

DOI: 10.1016/j.apmr.2011.04.025	
Implementation of electronic patient reported outcomes in pediatric daily clinical practice: The KLIK experience<br>Haverman, L; Van Oers, H A; Limperg, P F; Hijmans, C T; Schepers, S A; Sint Nicolaas, S M; Verhaak, C M; Bouts, A H M; Fijnvandraat, K; Peters, M; Van Rossum, M A; Van Goudoever, J B; Maurice-Stam, H; Grootenhuis, M A<br>Clinical Practice in Pediatric Psychology // 2014;2(1):50-67<br>2014 //<br>DOI: 10.1037/cpp0000043	Did not utilize or assess tool
Implementation of a Mental Health Screening Program in a Pediatric Tertiary Care Setting<br>Herbert, L; Hardy, S<br>Clinical Pediatrics // 2019;58(10):1078-1084<br>2019 //<br>DOI: 10.1177/0009922819862613	Did not utilize or assess tool
Responsiveness to Change in PROMIS® Measures among Children with Asthma: A Report from the PROMIS® Pediatric Asthma Study<br>Howell, C R; Thompson, L A; Gross, H E; Reeve, B B; Dewalt, D A; Huang, I.-C.<br>Value in Health // 2016;19(2):192-201<br>2016 //<br>DOI: 10.1016/j.jval.2015.12.004	Not chronic pain population
Gaining the PROMIS perspective from children with nephrotic syndrome: A Midwest pediatric nephrology consortium study<br>Gipson, D S; Selewski, D T; Massengill, S F; Wickman, L; Messer, K L; Herreshoff, E; Corinna, B; Ferris, M E; Mahan, J D; Greenbaum, L A; MacHardy, J; Kapur, G; Chand, D H; Goebel, J; Barletta, G M; Geary, D; Kershaw, D B; Pan, C G; Gbadegesin, R; Hidalgo, G; Lane, J C; Leiser, J D; Plattner, B W; Song, P X; Thissen, D; Liu, Y; Gross, H E; DeWalt, D A<br>Health and Quality of Life Outcomes // 2013;11(1):<br>2013 //<br>DOI: 10.1186/1477-7525-11-30	Not chronic pain population
Evaluating PROMIS® instruments and methods for patient-centered outcomes research: Patient and provider voices in a substance use treatment setting<br>Johnston, K L; Lawrence, S M; Dodds, N E; Yu, L; Daley, D C; Pilkonis, P A<br>Quality of Life Research // 2016;25(3):615-624<br>2016 //<br>DOI: 10.1007/s11136-015-1131-3	Did not utilize or assess tool

Pain as a quality of care measure in juvenile idiopathic arthritis: One step forward, but is it the best foot? Comment on the article by Lovell et al<br>Connelly, M; Von Baeyer, C L; Stinson, J; Schanberg, L E<br>Arthritis Care and Research // 2011;63(9):1352-1353<br>2011 //<br>DOI: 10.1002/acr.20515	Not an original study
Importance ratings on patient-reported outcome items for survivorship care: comparison between pediatric cancer survivors, parents, and clinicians<br>Jones, C M; Baker, J N; Keesey, R M; Eliason, R J; Lanctot, J Q; Clegg, J L; Mandrell, B N; Ness, K K; Krull, K R; Srivastava, D; Forrest, C B; Hudson, M M; Robison, L L; Huang, I.-C.<br>Quality of Life Research // 2018;27(7):1877-1884<br>2018 //<br>DOI: 10.1007/s11136-018-1854-z	Does not include at least one of: Tool Development or Psychometric Evaluation
Impaired Patient-Reported Outcomes Predict Poor School Functioning and Daytime Sleepiness: The PROMIS Pediatric Asthma Study<br>Jones, C M; DeWalt, D A; Huang, I.-C.<br>Academic Pediatrics // 2017;17(8):850-854<br>2017 //<br>DOI: 10.1016/j.acap.2017.07.010	Not chronic pain population
Patient Reported Outcomes Measurement Information System and Quality of Life in Neurological Disorders Measurement System to Evaluate Quality of Life for Children and Adolescents with Neurofibromatosis Type 1 Associated Plexiform Neurofibroma<br>Lai, J.-S.; Jensen, S E; Charrow, J; Listernick, R<br>Journal of Pediatrics // 2019;206():190-196<br>2019 //<br>DOI: 10.1016/j.jpeds.2018.10.019	Does not include at least one of: Tool Development or Psychometric Evaluation
A ResearchKit app to deliver paediatric electronic consent: Protocol of an observational study in adolescents with arthritis<br>Lalloo, C; Pham, Q; Cafazzo, J; Stephenson, E; Stinson, J<br>Contemporary Clinical Trials Communications // 2020;17():<br>2020 //<br>DOI: 10.1016/j.conctc.2020.100525	Not an original study

Construction of the eight-item patient-reported outcomes measurement information system pediatric physical function scales: Built using item response theory<br>Dewitt, E M; Stucky, B D; Thissen, D; Irwin, D E; Langer, M; Varni, J W; Lai, J.-S.; Yeatts, K B; Dewalt, D A<br>Journal of Clinical Epidemiology // 2011;64(7):794-804<br>2011 //<br>DOI: 10.1016/j.jclinepi.2010.10.012	Does not utilize or assess tool
PROMIS pediatric Anger scale: An item response theory analysis<br>Irwin, D E; Stucky, B D; Langer, M M; Thissen, D; DeWitt, E M; Lai, J.-S.; Yeatts, K B; Varni, J W; De Walt, D A<br>Quality of Life Research // 2012;21(4):697-706<br>2012 //<br>DOI: 10.1007/s11136-011-9969-5	Does not utilize or assess tool
PedsQL gastrointestinal symptoms module item development: Qualitative methods<br>Varni, J W; Kay, M T; Limbers, C A; Franciosi, J P; Pohl, J F<br>Journal of Pediatric Gastroenterology and Nutrition // 2012;54(5):664-671<br>2012 //<br>DOI: 10.1097/MPG.0b013e31823c9b88	Does not utilize or assess tool
Promoting psychological flexibility in parents of adolescents with chronic pain: Pilot study of an 8-week group intervention<br>Wallace, D P; Woodford, B; Connelly, M<br>Clinical Practice in Pediatric Psychology // 2016;4(4):405-416<br>2016 //<br>DOI: 10.1037/cpp0000160	Does not include at least one of: Tool Development or Psychometric Evaluation
A systematic review of pediatric self-report symptom measures: Congruence with the theory of unpleasant symptoms<br>von Sadovszky, V; Christensen, E; Jennings, B M; Miller, S; Hosley, S; Drought, L; Lenz, E R<br>Journal for Specialists in Pediatric Nursing // 2018;23(2):<br>2018 //<br>DOI: 10.1111/jspn.12215	Not an original study
Integrating the Concept of Pain Interference into Pain Management<br>Wilson, M<br>Pain Management Nursing // 2014;15(2):499-505<br>2014 //<br>DOI: 10.1016/j.pmn.2011.06.004	Not an original study

Psychometric properties of the brief pain inventory modified for proxy report of pain interference in children with cerebral palsy with and without cognitive impairment<br>Barney, C C; Stibb, S M; Merbler, A M; Summers, R L S; Deshpande, S; Krach, L E; Symons, F J<br>Pain Reports // 2018;3(4):<br>2018 //<br>DOI: 10.1097/PR9.0000000000000666	Does not utilize or assess tool
Quantitative sensory testing is feasible and is well-tolerated in patients with sickle cell disease following a vaso-occlusive episode<br>Bakshi, N; Lukombo, I; Belfer, I; Krishnamurti, L<br>Journal of Pain Research // 2018;11():435-443<br>2018 //<br>DOI: 10.2147/JPR.S150066	Does not include at least one of: Tool Development or Psychometric Evaluation
Psychological Characteristics and Pain Frequency Are Associated With Experimental Pain Sensitivity in Pediatric Patients With Sickle Cell Disease<br>Bakshi, N; Lukombo, I; Shnol, H; Belfer, I; Krishnamurti, L<br>Journal of Pain // 2017;18(10):1216-1228<br>2017 //<br>DOI: 10.1016/j.jpain.2017.05.005	Does not include at least one of: Tool Development or Psychometric Evaluation
The CALI-9: A brief measure for assessing activity limitations in children and adolescents with chronic pain<br>Holley, A L; Zhou, C; Wilson, A C; Hainsworth, K; Palermo, T M<br>Pain // 2018;159(1):48-56<br>2018 //<br>DOI: 10.1097/j.pain.0000000000001063	Does not utilize or assess tool
Evaluating the Statistical Properties of the Pain Interference Index in Children and Adolescents with Chronic Pain<br>Holmström, L; Kemani, M K; Kanstrup, M; Wicksell, R K<br>Journal of Developmental and Behavioral Pediatrics // 2015;36(6):450-454<br>2015 //<br>DOI: 10.1097/DBP.0000000000000191	Does not utilize or assess tool
Maternal Protective Parenting Accounts for the Relationship between Pain Behaviors and Functional Disability in Adolescents<br>Lynch-Jordan, A M; Peugh, J; Cunningham, N R; Trygier, J R; Kashikar-Zuck, S	Does not utilize or assess tool

Clinical Journal of Pain // 2018;34(12):1089-1095<br>2018 //<br>DOI: 10.1097/AJP.0000000000000638	
Measuring musculoskeletal pain in infants, children, and adolescents<br>Michaleff, Z A; Kamper, S J; Stinson, J N; Hestbaek, L; Williams, C M; Campbell, P; Dunn, K M<br>Journal of Orthopaedic and Sports Physical Therapy // 2017;47(10):712-730<br>2017 //<br>DOI: 10.2519/jospt.2017.7469	Not an original study
Development of a computer-adaptive physical function instrument for social security administration disability determination<br>Ni, P; McDonough, C M; Jette, A M; Bogusz, K; Marfeo, E E; Rasch, E K; Brandt, D E; Meterko, M; Haley, S M; Chan, L<br>Archives of Physical Medicine and Rehabilitation // 2013;94(9):1661-1669<br>2013 //<br>DOI: 10.1016/j.apmr.2013.03.021	Did not utilize or assess tool
PedsQL™ sickle cell disease module: Feasibility, reliability, and validity<br>Panepinto, J A; Torres, S; Bendo, C B; Mccavit, T L; Dinu, B; Sherman-Bien, S; Bemrich-Stolz, C; Varni, J W<br>Pediatric Blood and Cancer // 2013;60(8):1338-1344<br>2013 //<br>DOI: 10.1002/pbc.24491	Did not utilize or assess tool
The emotional distress of children with cancer in China: an item response analysis of C-Ped-PROMIS Anxiety and Depression short forms<br>Liu, Y; Wang, J; Hinds, P S; Shen, N; Zhao, X; Ding, J; Yuan, C<br>Quality of Life Research // 2015;24(6):1491-1501<br>2015 //<br>DOI: 10.1007/s11136-014-0870-x	Did not utilize or assess tool
Late Effects Screening Guidelines after Hematopoietic Cell Transplantation (HCT) for Hemoglobinopathy: Consensus Statement from the Second Pediatric Blood and Marrow Transplant Consortium International Conference on Late Effects after Pediatric HCT<br>Shenoy, S; Gaziev, J; Angelucci, E; King, A; Bhatia, M; Smith, A; Bresters, D; Haight, A E; Duncan, C N; de la Fuente, J; Dietz, A C; Baker, K S; Pulsipher, M A; Walters, M C<br>Biology of Blood and Marrow Transplantation // 2018;24(7):1313-1321<br>2018 //<br>DOI: 10.1016/j.bbmt.2018.04.002	Did not utilize or assess tool

IACT - An interactive mHealth monitoring system to enhance psychotherapy for adolescents with sickle cell disease<br>Cheng, C; Brown, R C; Cohen, L L; Venugopalan, J; Stokes, T H; Wang, M D<br>Proceedings of the Annual International Conference of the IEEE Engineering in Medicine and Biology Society, EMBS // 2013;():2279-2282<br>2013 //<br>DOI: 10.1109/EMBC.2013.6609992	Does not include at least one of: Tool Development or Psychometric Evaluation
SickleREMOTE: A two-way text messaging system for pediatric sickle cell disease patients<br>Cheng, C; Brown, C; New, T; Stokes, T H; Dampier, C; Wang, M D<br>Proceedings - IEEE-EMBS International Conference on Biomedical and Health Informatics: Global Grand Challenge of Health Informatics, BHI 2012 // 2012;():408-411<br>2012 //<br>DOI: 10.1109/BHI.2012.6211602	Does not include at least one of: Tool Development or Psychometric Evaluation
Health-Related Quality of Life Components in Children With Neonatal Brachial Plexus Palsy: A Qualitative Study<br>Chang, K.W.-C.; Austin, A; Yeaman, J; Phillips, L; Kratz, A; Yang, L.J.-S.; Carlozzi, N E<br>PM and R // 2017;9(4):383-391<br>2017 //<br>DOI: 10.1016/j.pmrj.2016.08.002	Does not utilize or assess tool
Safety of Active Rehabilitation for Persistent Symptoms After Pediatric Sport-Related Concussion: A Randomized Controlled Trial<br>Chan, C; Iverson, G L; Purtzki, J; Wong, K; Kwan, V; Gagnon, I; Silverberg, N D<br>Archives of Physical Medicine and Rehabilitation // 2018;99(2):242-249<br>2018 //<br>DOI: 10.1016/j.apmr.2017.09.108	Does not utilize or assess tool
Availability of researcher-led eHealth tools for pain assessment and management: Barriers, facilitators, costs, and design<br>Higgins, K S; Tutelman, P R; Chambers, C T; Witteman, H O; Barwick, M; Corkum, P; Grant, D; Stinson, J N; Lalloo, C; Robins, S; Orji, R; Jordan, I<br>Pain Reports // 2018;3(7):<br>2018 //<br>DOI: 10.1097/PR9.0000000000000686	Not an original study

Testing the Feasibility and Psychometric Properties of a Mobile Diary (myWHI) in Adolescents and Young Adults With Headaches<br>Huguet, Anna; McGrath, Patrick J; Wheaton, Michael; Mackinnon, Sean P; Rozario, Sharlene; Tougas, Michelle E; Stinson, Jennifer N; MacLean, Cathy<br>JMIR MHEALTH AND UHEALTH // 2015;3(2):<br>59 WINNERS CIRCLE, TORONTO, ON M4L 3Y7, CANADA JMIR PUBLICATIONS, INC 2015 //<br>DOI: 10.2196/mhealth.3879	Does not utilize or assess tool
Using Rasch rating scale model to reassess the psychometric properties of the Persian version of the PedsQL TM 4.0 Generic Core Scales in school children<br>Jafari, P; Bagheri, Z; Ayatollahi, S M T; Soltani, Z<br>Health and Quality of Life Outcomes // 2012;10():<br>2012 //<br>DOI: 10.1186/1477-7525-10-27	Does not utilize or assess tool
Development and Evaluation of the PROMISA (R) Pediatric Positive Affect Item Bank, Child-Report and Parent-Proxy Editions<br>Forrest, Christopher B; Ravens-Sieberer, Ulrike; Devine, Janine; Becker, Brandon D; Teneralli, Rachel E; Moon, JeanHee; Carle, Adam C; Tucker, Carole A; Bevans, Katherine B<br>JOURNAL OF HAPPINESS STUDIES 03// 2018;19(3):699-718<br>VAN GODEWIJCKSTRAAT 30, 3311 GZ DORDRECHT, NETHERLANDS SPRINGER 2018 03//<br>DOI: 10.1007/s10902-016-9843-9	Duplicate
No Title<br>;():	Cannot locate full text
Pain assessment using the NIH Toolbox<br>Cook, Karon F; Dunn, Winnie; Griffith, James W; Morrison, M Tracy; Tanquary, Jennifer; Sabata, Dory; Victorson, David; Carey, Leeanne M; MacDermid, Joy C; Dudgeon, Brian J; Gershon, Richard C<br>NEUROLOGY 03// 2013;80(3):S49-S53<br>TWO COMMERCE SQ, 2001 MARKET ST, PHILADELPHIA, PA 19103 USA LIPPINCOTT WILLIAMS & WILKINS 2013 03//<br>DOI: 10.1212/WNL.0b013e3182872e80	Not an original study
PROMIS pediatric peer relationships scale: Development of a peer relationships item bank as part of social health measurement<br>DeWalt, D A; Thissen, D; Stucky, B D; Langer, M M; DeWitt, E M; Irwin, D E; Lai, J.-S.; Yeatts, Karin B; Gross, H E; Taylor, O; Varni, J W<br>Health Psychology // 2013;32(10):1093-1103<br>2013 //	Does not utilize or assess tool

DOI: 10.1037/a0032670	
Concurrent validity of the PROMISA (R) pediatric global health measure<br>Forrest, Christopher B; Tucker, Carole A; Ravens-Sieberer, Ulrike; Pratiwadi, Ramya; Moon, JeanHee; Teneralli, Rachel E; Becker, Brandon; Bevans, Katherine B<br>QUALITY OF LIFE RESEARCH 03// 2016;25(3):739-751<br>VAN GODEWIJCKSTRAAT 30, 3311 GZ DORDRECHT, NETHERLANDS SPRINGER 2016 03//<br>DOI: 10.1007/s11136-015-1111-7	Duplicate
Quantifying 'problematic' DIF within an IRT framework: application to a cancer stigma index<br>Edelen, M O; Stucky, B D; Chandra, A<br>Quality of Life Research // 2015;24(1):95-103<br>2015 //<br>DOI: 10.1007/s11136-013-0540-4	Does not utilize or assess tool
PedsQL eosinophilic esophagitis module: Feasibility, reliability, and validity<br>Franciosi, J P; Hommel, K A; Bendo, C B; King, E C; Collins, M H; Eby, M D; Marsolo, K; Abonia, J P; Von Tiehl, K F; Putnam, P E; Greenler, A J; Greenberg, A B; Bryson, R A; Davis, C M; Olive, A P; Gupta, S K; Erwin, E A; Klinnert, M D; Spergel, J M; Denham, J M; Furuta, G T; Rothenberg, M E; Varni, J W<br>Journal of Pediatric Gastroenterology and Nutrition // 2013;57(1):57-66<br>2013 //<br>DOI: 10.1097/MPG.0b013e31828f1fd2	Does not utilize or assess tool
Symptoms and quality of life indicators among children with chronic medical conditions<br>Kim, J; Chung, H; Amtmann, D; Salem, R; Park, R; Askew, R L<br>Disability and Health Journal // 2014;7(1):96-104<br>2014 //<br>DOI: 10.1016/j.dhjo.2013.08.007	Does not utilize or assess tool
Quality assessment of ophthalmic questionnaires: Review and recommendations<br>Khadka, J; McAlinden, C; Pesudovs, K<br>Optometry and Vision Science // 2013;90(8):720-744<br>2013 //<br>DOI: 10.1097/OPX.0000000000000001	Does not utilize or assess tool
Development of a mHealth Real-Time Pain Self-Management App for Adolescents With Cancer: An Iterative Usability Testing Study<br>Jibb, L A; Cafazzo, J A; Nathan, P C; Seto, E; Stevens, B J; Nguyen, C; Stinson, J N<br>Journal of Pediatric Oncology Nursing // 2017;34(4):283-294	Does not utilize or assess tool

2017 //<br>DOI: 10.1177/1043454217697022	
A scoping review of pain in children after traumatic brain injury: Is there more than headache?<br>Kwan, V; Vo, M; Noel, M; Yeates, K<br>Journal of Neurotrauma // 2018;35(7):877-888<br>2018 //<br>DOI: 10.1089/neu.2017.5281	Not an original study
Validation of the Sickle Cell Disease Pain Burden Interview-Youth<br>Zempsky, William T; O'hara, Emily A; Santanelli, James P; Palermo, Tonya M; New, Tamara; Smith-Whitley, Kim; Casella, James F<br>// 2013;():<br>2013 //<br>DOI: 10.1016/j.jpain.2013.03.007	Does not include at least one of: Tool Development or Psychometric Evaluation
Factorial invariance of pediatric patient self-reported fatigue across age and gender: A multigroup confirmatory factor analysis approach utilizing the PedsQLTM Multidimensional Fatigue Scale<br>Varni, J W; Beaujean, A A; Limbers, C A<br>Quality of Life Research // 2013;22(9):2581-2594<br>2013 //<br>DOI: 10.1007/s11136-013-0370-4	Does not utilize or assess tool
Development of the pediatric quality of life inventory neurofibromatosis type 1 module items for children, adolescents and young adults: qualitative methods<br>Nutakki, K; Varni, J W; Steinbrenner, S; Draucker, C B; Swigonski, N L<br>Journal of Neuro-Oncology // 2017;132(1):135-143<br>2017 //<br>DOI: 10.1007/s11060-016-2351-2	Does not utilize or assess tool
PedsQL gastrointestinal symptoms module: Feasibility, reliability, and validity<br>Varni, J W; Bendo, C B; Denham, J; Shulman, R J; Self, M M; Neigut, D A; Nurko, S; Patel, A S; Franciosi, J P; Saps, M; Verga, B; Smith, A; Yeckes, A; Heinz, N; Langseder, A; Saeed, S; Zacur, G M; Pohl, J F<br>Journal of Pediatric Gastroenterology and Nutrition // 2014;59(3):347-355<br>2014 //<br>DOI: 10.1097/MPG.0000000000000414	Does not utilize or assess tool
A qualitative study of the impact of cancer on romantic relationships, sexual relationships, and fertility: Perspectives of Canadian adolescents and parents during and after treatment	Does not utilize or assess tool

Stinson, J N; Jibb, L A; Greenberg, M; Barrera, M; Luca, S; White, M E; Gupta, A<br>Journal of Adolescent and Young Adult Oncology // 2015;4(2):84-90<br>2015 //<br>DOI: 10.1089/jayao.2014.0036	
An observational study of patient versus parental perceptions of health-related quality of life in children and adolescents with a chronic pain condition: who should the clinician believe?<br>Vetter, T R; Bridgewater, C L; McGwin Jr, G<br>Health and Quality of Life Outcomes // 2012;10():<br>2012 //<br>DOI: 10.1186/1477-7525-10-85	Does not utilize or assess tool
Somatosensory test responses and physical and psychological functioning of children and adolescents with chronic non-neuropathic pain: An exploratory study<br>Lim, S W; Gunaratne, Y; Jaaniste, T; McCormick, M; Champion, D<br>Clinical Journal of Pain // 2017;33(2):116-125<br>2017 //<br>DOI: 10.1097/AJP.0000000000000385	Does not include at least one of: Tool Development or Psychometric Evaluation
Application of item response theory to tests of substance-related associative memory<br>Shono, Y; Grenard, J L; Ames, S L; Stacy, A W<br>Psychology of Addictive Behaviors // 2014;28(3):852-862<br>2014 //<br>DOI: 10.1037/a0035877	Does not utilize or assess tool
Applying the patient-reported outcomes measurement information system to assess upper extremity function among children with congenital hand differences<br>Waljee, J F; Carlozzi, N; Franzblau, L E; Zhong, L; Chung, K C<br>Plastic and Reconstructive Surgery // 2015;136(2):200e-207e<br>2015 //<br>DOI: 10.1097/PRS.0000000000001444	Does not utilize or assess tool
Accelerating the drug delivery pipeline for acute and chronic pancreatitis-knowledge gaps and research opportunities: Overview summary of a National Institute of Diabetes and Digestive and Kidney Diseases workshop<br>Uc, A; Andersen, D K; Borowitz, D; Glesby, M J; Mayerle, J; Sutton, R; Pandol, S J<br>Pancreas // 2018;47(10):1180-1184<br>2018 //	Does not utilize or assess tool

DOI: 10.1097/MPA.0000000000001176	
Developing Item Response Theory–Based Short Forms to Measure the Social Impact of Burn Injuries<br>Marino, M E; Dore, E C; Ni, P; Ryan, C M; Schneider, J C; Acton, A; Jette, A M; Kazis, L E<br>Archives of Physical Medicine and Rehabilitation // 2018;99(3):521-528<br>2018 //<br>DOI: 10.1016/j.apmr.2017.06.037	Do not utilize or assess tool
Stigma and Pain in Adolescents Hospitalized for Sickle Cell Vasoocclusive Pain Episodes<br>Martin, S R; Cohen, L L; Mougianis, I; Griffin, A; Sil, S; Dampier, C<br>Clinical Journal of Pain // 2018;34(5):438-444<br>2018 //<br>DOI: 10.1097/AJP.0000000000000553	Does not include at least one of: Tool Development or Psychometric Evaluation
Comparing the predictive value of task performance and task-specific sensitivity during physical function testing among people with knee osteoarthritis<br>Wideman, T H; Edwards, R R; Finan, P H; Haythornthwaite, J A; Smith, M T<br>Journal of Orthopaedic and Sports Physical Therapy // 2016;46(5):346-356<br>2016 //<br>DOI: 10.2519/jospt.2016.6311	Does not utilize or assess tool
A high preoperative pain and symptom profile predicts worse pain outcomes for children after spine fusion surgery<br>Voepel-Lewis, T; Caird, M S; Tait, A R; Malviya, S; Farley, F A; Li, Y; Abbott, M D; Van Veen, T; Hassett, A L; Clauw, D J<br>Anesthesia and Analgesia // 2017;124(5):1594-1602<br>2017 //<br>DOI: 10.1213/ANE.0000000000001963	Does not include at least one of: Tool Development or Psychometric Evaluation
Development of the PedsQL (TM) sickle cell disease module items: qualitative methods<br>Panepinto, Julie A; Torres, Sylvia; Varni, James W<br>QUALITY OF LIFE RESEARCH 03// 2012;21(2):341-357<br>VAN GODEWIJCKSTRAAT 30, 3311 GZ DORDRECHT, NETHERLANDS SPRINGER 2012 03//<br>DOI: 10.1007/s11136-011-9941-4	Does not utilize or assess tool
Evaluating PROMISA (R) instruments and methods for patient-centered outcomes research: Patient and provider voices in a substance use treatment setting<br>Johnston, Kelly L; Lawrence, Suzanne M; Dodds, Nathan E; Yu, Lan; Daley, Dennis C; Pilkonis, Paul A	Duplicate

QUALITY OF LIFE RESEARCH 03// 2016;25(3):615-624<br>VAN GODEWIJCKSTRAAT 30, 3311 GZ DORDRECHT, NETHERLANDS SPRINGER 2016 03//<br>DOI: 10.1007/s11136-015-1131-3	
A method to create a standardized generic and condition-specific patient-reported outcome measure for patient care and healthcare improvement<br>Schifferdecker, K E; Yount, S E; Kaiser, K; Adachi-Mejia, A; Cella, D; Carluzzo, K L; Eisenstein, A; Kallen, M A; Greene, G J; Eton, D T; Fisher, E S<br>Quality of Life Research // 2018;27(2):367-378<br>2018 //<br>DOI: 10.1007/s11136-017-1675-5	Not chronic pain population
Longitudinal associations among asthma control, sleep problems, and health-related quality of life in children with asthma: A report from the PROMIS® Pediatric Asthma Study<br>Li, Z; Thompson, L A; Gross, H E; Shenkman, E A; Reeve, B B; DeWalt, D A; Huang, I.-C.<br>Sleep Medicine // 2016;20():41-50<br>2016 //<br>DOI: 10.1016/j.sleep.2015.12.003	Not chronic pain population
Promising insights into the health related quality of life for children with severe obesity<br>Selewski, D T; Collier, D N; MacHardy, J; Gross, H E; Pickens, E M; Cooper, A W; Bullock, S; Earls, M F; Pratt, K J; Scanlon, K; McNeill, J D; Messer, K L; Lu, Y; Thissen, D; DeWalt, D A; Gipson, D S<br>Health and Quality of Life Outcomes // 2013;11(1):<br>2013 //<br>DOI: 10.1186/1477-7525-11-29	Not chronic pain population
Acute and Chronic Pain in Children and Adolescents With Cerebral Palsy: Prevalence, Interference, and Management<br>Ostojic, K; Paget, S; Kyriagis, M; Morrow, A<br>Archives of Physical Medicine and Rehabilitation // 2020;101(2):213-219<br>2020 //<br>DOI: 10.1016/j.apmr.2019.08.475	Does not include at least one of: Tool Development or Psychometric Evaluation
Screening and assessment of chronic pain among children with cerebral palsy: a process evaluation of a pain toolbox<br>Orava, T; Provvidenza, C; Townley, A; Kingsnorth, S<br>Disability and Rehabilitation // 2019;41(22):2695-2703<br>2019 //	Not an original study

DOI: 10.1080/09638288.2018.1471524	
Development of a Smartphone Application to Monitor Pediatric Patient-Reported Outcomes<br>Wang, J; Yao, N A; Liu, Y; Geng, Z; Wang, Y; Shen, N; Zhang, X; Shen, M; Yuan, C<br>CIN - Computers Informatics Nursing // 2017;35(11):590-598<br>2017 //<br>DOI: 10.1097/CIN.0000000000000357	Not chronic pain population
HABIT efficacy and sustainability trial, a multi-center randomized controlled trial to improve hydroxyurea adherence in youth with sickle cell disease: A study protocol<br>Smaldone, A; Manwani, D; Aygun, B; Smith-Whitley, K; Jia, H; Bruzzese, J.-M.; Findley, S; Massei, J; Green, N S<br>BMC Pediatrics // 2019;19(1):<br>2019 //<br>DOI: 10.1186/s12887-019-1746-6	Not an original study
Psychological Interventions for Headache in Children and Adolescents<br>Sieberg, Christine B; Huguet, Anna; von Baeyer, Carl L; Seshia, Shashi S<br>CANADIAN JOURNAL OF NEUROLOGICAL SCIENCES 01// 2012;39(1):26-34<br>32 AVENUE OF THE AMERICAS, NEW YORK, NY 10013-2473 USA CAMBRIDGE UNIV PRESS 2012 01//<br>DOI: 10.1017/S0317167100012646	Not an original study
Establishing clinical meaning and defining important differences for Patient-Reported Outcomes Measurement Information System (PROMISA (R)) measures in juvenile idiopathic arthritis using standard setting with patients, parents, and providers<br>Morgan, Esi M; Mara, Constance A; Huang, Bin; Barnett, Kimberly; Carle, Adam C; Farrell, Jennifer E; Cook, Karon F<br>QUALITY OF LIFE RESEARCH 03// 2017;26(3):565-586<br>VAN GODEWIJCKSTRAAT 30, 3311 GZ DORDRECHT, NETHERLANDS SPRINGER 2017 03//<br>DOI: 10.1007/s11136-016-1468-2	Duplicate
Subjective well-being measures for children were developed within the PROMIS project: Presentation of first results<br>Ravens-Sieberer, U; Devine, J; Bevans, K; Riley, A W; Moon, J; Salsman, J M; Forrest, C B<br>Journal of Clinical Epidemiology // 2014;67(2):207-218<br>2014 //<br>DOI: 10.1016/j.jclinepi.2013.08.018	Does not utilize or assess tool
The impact of disease duration on quality of life in children with nephrotic syndrome: a Midwest Pediatric Nephrology Consortium study	Not chronic pain population

Selewski, D T; Troost, J P; Massengill, S F; Gbadegesin, R A; Greenbaum, L A; Shatat, I F; Cai, Y; Kapur, G; Hebert, D; Somers, M J; Trachtman, H; Pais, P; Seifert, M E; Goebel, J; Sethna, C B; Mahan, J D; Gross, H E; Herreshoff, E; Liu, Y; Song, P X; Reeve, B B; DeWalt, D A; Gipson, D S<br>Pediatric Nephrology // 2015;30(9):1467-1476<br>2015 //<br>DOI: 10.1007/s00467-015-3074-x	
Responsiveness of the PROMIS® measures to changes in disease status among pediatric nephrotic syndrome patients: A Midwest pediatric nephrology consortium study<br>Selewski, D T; Troost, J P; Cummings, D; Massengill, S F; Gbadegesin, R A; Greenbaum, L A; Shatat, I F; Cai, Y; Kapur, G; Hebert, D; Somers, M J; Trachtman, H; Pais, P; Seifert, M E; Goebel, J; Sethna, C B; Mahan, J D; Gross, H E; Herreshoff, E; Liu, Y; Carlozzi, N E; Reeve, B B; DeWalt, D A; Gipson, D S<br>Health and Quality of Life Outcomes // 2017;15(1):<br>2017 //<br>DOI: 10.1186/s12955-017-0737-2	Not chronic pain population
Developing a standardized approach to the assessment of pain in children and youth presenting to pediatric rheumatology providers: A Delphi survey and consensus conference process followed by feasibility testing<br>Stinson, J N; Connelly, M; Jibb, L A; Schanberg, L E; Walco, G; Spiegel, L R; Tse, S M L; Chalom, E C; Chira, P; Rapoff, M<br>Pediatric Rheumatology // 2012;10():<br>2012 //<br>DOI: 10.1186/1546-0096-10-7	Does not utilize or assess tool
Using the PedsQLTM 3.0 asthma module to obtain scores comparable with those of the PROMIS pediatric asthma impact scale (PAIS)<br>Thissen, D; Varni, J W; Stucky, B D; Liu, Y; Irwin, D E; DeWalt, D A<br>Quality of Life Research // 2011;20(9):1497-1505<br>2011 //<br>DOI: 10.1007/s11136-011-9874-y	Does not utilize or assess tool
Using Logistic Approximations of Marginal Trace Lines to Develop Short Assessments<br>Stucky, B D; Thissen, D; Orlando Edelen, M<br>Applied Psychological Measurement // 2013;37(1):41-57<br>2013 //<br>DOI: 10.1177/0146621612462759	Not an original study
Using PROMIS® to create clinically meaningful profiles of nephrotic syndrome patients	Not chronic pain population

Troost, J P; Gipson, D S; Carlozzi, N E; Reeve, B B; Nachman, P H; Gbadegesin, R; Wang, J; Modersitzki, F; Massengill, S; Mahan, J D; Liu, Y; Trachtman, H; Herreshoff, E G; Dewalt, D A; Selewski, D T<br>Health Psychology // 2019;38(5):410-421<br>2019 //<br>DOI: 10.1037/hea0000679	
TBI-QOL: Development and calibration of item banks to measure patient reported outcomes following traumatic brain injury<br>Tulsky, D S; Kisala, P A; Victorson, D; Carlozzi, N; Bushnik, T; Sherer, M; Choi, S W; Heinemann, A W; Chiaravalloti, N; Sander, A M; Englander, J; Hanks, R; Kolakowsky-Hayner, S; Roth, E; Gershon, R; Rosenthal, M; Cella, D<br>Journal of Head Trauma Rehabilitation // 2016;31(1):40-51<br>2016 //<br>DOI: 10.1097/HTR.0000000000000131	Does not utilize or assess tool
Feasibility of a randomized controlled trial of paediatric interdisciplinary pain management using home-based telehealth<br>Hilyard, Anna; Kingsley, Julia; Sommerfield, David; Taylor, Susan; Bear, Natasha; Gibson, Noula<br>Journal of Pain Research 2020;13():897-908<br>Dove Medical Press Ltd. 2020<br>DOI: 10.2147/JPR.S217022	Does not utilize or assess tool
The Epidemiology of Back Pain in American Children and Adolescents<br>Fabricant, Peter D.; Heath, Madison R.; Schachne, Jonathan M.; Doyle, Shevaun M.; Green, Daniel W.; Widmann, Roger F.<br>Spine 2020;45(16):1135-1142<br>Lippincott Williams and Wilkins 2020<br>DOI: 10.1097/BRS.0000000000003461	Not chronic pain population
Dutch–Flemish translation of nine pediatric item banks from the Patient-Reported Outcomes Measurement Information System (PROMIS)®<br>Haverman, L; Grootenhuis, M A; Raat, H; van Rossum, M A J; van Dulmen-den Broeder, E; Hoppenbrouwers, K; Correia, H; Cella, D; Roorda, L D; Terwee, C B<br>Quality of Life Research // 2016;25(3):761-765<br>2016 //<br>DOI: 10.1007/s11136-015-0966-y	Is a translation article
Disclosure and self-report of emotional, social, and physical health in children and adolescents with chronic pain - A qualitative study of PROMIS pediatric measures<br>Jacobson, C J; Farrell, J E; Kashikar-Zuck, S; Seid, M; Verkamp, E; Dewitt, E M	Qualitative study

Journal of Pediatric Psychology // 2013;38(1):82-93<br>2013 //<br>DOI: 10.1093/jpepsy/jss099	
PROMIS pediatric measures in pediatric oncology: Valid and clinically feasible indicators of patient-reported outcomes<br>Hinds, P S; Nuss, S L; Ruccione, K S; Withycombe, J S; Jacobs, S; Deluca, H; Faulkner, C; Liu, Y; Cheng, Y I; Gross, H E; Wang, J; Dewalt, D A<br>Pediatric Blood and Cancer // 2013;60(3):402-408<br>2013 //<br>DOI: 10.1002/pbc.24233	Not chronic pain population
Chinese Version of Pediatric Patient-Reported Outcomes Measurement Information System Short Form Measures Reliability, Validity, and Factorial Structure Assessment in Children With Cancer in China<br>Liu, Yanyan; Yuan, Changrong; Wang, Jichuan; Shen, Nanping; Shen, Min; Hinds, Pamela S<br>CANCER NURSING // 2019;42(6):430-438<br>TWO COMMERCE SQ, 2001 MARKET ST, PHILADELPHIA, PA 19103 USA LIPPINCOTT WILLIAMS & WILKINS 2019 //<br>DOI: 10.1097/NCC.0000000000000633	Not chronic pain population
Psychometric evaluation of the pediatric and parent-proxy Patient-Reported Outcomes Measurement Information System and the Neurology and Traumatic Brain Injury Quality of Life measurement item banks in pediatric traumatic brain injury<br>Bertisch, H; Rivara, F P; Kisala, P A; Wang, J; Yeates, K O; Durbin, D; Zonfrillo, M R; Bell, M J; Temkin, N; Tulsky, D S<br>Quality of Life Research // 2017;26(7):1887-1899<br>2017 //<br>DOI: 10.1007/s11136-017-1524-6	Not chronic pain population
Feasibility and acceptability of the patient-reported outcomes measurement information system measures in children and adolescents in active cancer treatment and survivorship<br>Menard, J C; Hinds, P S; Jacobs, S S; Cranston, K; Wang, J; DeWalt, D A; Gross, H E<br>Cancer Nursing // 2014;37(1):66-74<br>2014 //<br>DOI: 10.1097/NCC.0b013e3182a0e23d	Not chronic pain population
The Validity of Patient-reported Outcome Measurement Information System (PROMIS) Parent Proxy Instruments to Assess Function in Children with Talipes Equinovarus<br>Masrouha, K Z; Moses, M J; Sala, D A; Litrenta, J; Lehman, W B; Chu, A<br>Journal of Pediatric Orthopaedics // 2019;39(10):E787-E790	Not chronic pain population

2019 //<br>DOI: 10.1097/BPO.0000000000001368	
Comparative Effectiveness Research and Children with Cerebral Palsy: Identifying a Conceptual Framework and Specifying Measures<br>Gannotti, M E; Law, M; Bailes, A F; O'Neil, M E; Williams, U; Direzze, B<br>Pediatric Physical Therapy // 2016;28(1):58-69<br>2016 //<br>DOI: 10.1097/PEP.0000000000000203	Not chronic pain population
Comparability of the Patient-Reported Outcomes Measurement Information System Pediatric short form symptom measures across culture: examination between Chinese and American children with cancer<br>Liu, Y; Yuan, C; Wang, J; Brown, J G; Zhou, F; Zhao, X; Shen, M; Hinds, P S<br>Quality of Life Research // 2016;25(10):2523-2533<br>2016 //<br>DOI: 10.1007/s11136-016-1312-8	Not chronic pain population
Gaining the Patient Reported Outcomes Measurement Information System (PROMIS) perspective in chronic kidney disease: a Midwest Pediatric Nephrology Consortium study<br>Selewski, D T; Massengill, S F; Troost, J P; Wickman, L; Messer, K L; Herreshoff, E; Bowers, C; Ferris, M E; Mahan, J D; Greenbaum, L A; MacHardy, J; Kapur, G; Chand, D H; Goebel, J; Barletta, G M; Geary, D; Kershaw, D B; Pan, C G; Gbadegesin, R; Hidalgo, G; Lane, J C; Leiser, J D; Song, P X; Thissen, D; Liu, Y; Gross, H E; DeWalt, D A; Gipson, D S<br>Pediatric Nephrology // 2014;29(12):2347-2356<br>2014 //<br>DOI: 10.1007/s00467-014-2858-8	Not chronic pain population
Clinical meaning of PROMIS pain domains for children with sickle cell disease<br>Singh, A; Panepinto, J A<br>Blood Advances // 2019;3(15):2244-2249<br>2019 //<br>DOI: 10.1182/bloodadvances.2019000381	Not exclusive chronic pain population
Translation and cross-cultural adaptation of eight pediatric PROMIS® item banks into Spanish and German<br>Devine, J; Klasen, F; Moon, J; Herdman, M; Hurtado, M P; Castillo, G; Haller, A C; Correia, H; Forrest, C B; Ravens-Sieberer, U<br>Quality of Life Research // 2018;27(9):2415-2430<br>2018 //<br>DOI: 10.1007/s11136-018-1874-8	Is a translation article

Patient-Reported Outcomes Measurement Information System Tools for Collecting Patient-Reported Outcomes in Children With Juvenile Arthritis<br>Brandon, T G; Becker, B D; Bevans, K B; Weiss, P F<br>Arthritis Care and Research // 2017;69(3):393-402<br>2017 //<br>DOI: 10.1002/acr.22937	Not exclusive chronic pain population
PROMIS® pediatric self-report scales distinguish subgroups of children within and across six common pediatric chronic health conditions<br>DeWalt, D A; Gross, H E; Gipson, D S; Selewski, D T; DeWitt, E M; Dampier, C D; Hinds, P S; Huang, I.-C.; Thissen, D; Varni, J W<br>Quality of Life Research // 2015;24(9):2195-2208<br>2015 //<br>DOI: 10.1007/s11136-015-0953-3	Not exclusive chronic pain population
Patterns of symptoms and functional impairments in children with cancer<br>Buckner, T W; Wang, J; Dewalt, D A; Jacobs, S; Reeve, B B; Hinds, P S<br>Pediatric Blood and Cancer // 2014;61(7):1282-1288<br>2014 //<br>DOI: 10.1002/pbc.25029	Not exclusive chronic pain population
Pain location and widespread pain in youth with orthopaedic conditions: Exploration of the reliability and validity of a body map<br>Foxen-Craft, E; Scott, E L; Kullgren, K A; Philliben, R; Hyman, C; Dorta, M; Murphy, A; Voepel-Lewis, T<br>European Journal of Pain (United Kingdom) // 2019;23(1):57-65<br>2019 //<br>DOI: 10.1002/ejp.1282	Not exclusive chronic pain population
Development and Evaluation of the PROMIS® Pediatric Positive Affect Item Bank, Child-Report and Parent-Proxy Editions<br>Forrest, C B; Ravens-Sieberer, U; Devine, J; Becker, B D; Teneralli, R E; Moon, J H; Carle, A C; Tucker, C A; Bevans, K B<br>Journal of Happiness Studies // 2018;19(3):699-718<br>2018 //<br>DOI: 10.1007/s10902-016-9843-9	Not exclusive chronic pain population
Validation of Patient-Reported Outcomes Measurement Information System Short Forms for Use in Childhood-Onset Systemic Lupus Erythematosus<br>Jones, J T; Carle, A C; Wootton, J; Liberio, B; Lee, J; Schanberg, L E; Ying, J; Morgan DeWitt, E; Brunner, H I<br>Arthritis Care and Research // 2017;69(1):133-142	Not exclusive chronic pain population

2017 //<br>DOI: 10.1002/acr.22927	
``Asking Too Much?{"}: Randomized N-of-1 Trial Exploring Patient Preferences and Measurement Reactivity to Frequent Use of Remote Multidimensional Pain Assessments in Children and Young People With Juvenile Idiopathic Arthritis<br>Lee, Rebecca Rachael; Shoop-Worrall, Stephanie; Rashid, Amir; Thomson, Wendy; Cordingley, Lis<br>JOURNAL OF MEDICAL INTERNET RESEARCH 01// 2020;22(1):<br>130 QUEENS QUAY E, STE 1102, TORONTO, ON M5A 0P6, CANADA JMIR PUBLICATIONS, INC 2020 01//<br>DOI: 10.2196/14503	Not exclusive chronic pain population
Translation and linguistic validation of the pediatric patient-reported outcomes measurement information system measures into simplified chinese using cognitive interviewing methodology<br>Liu, Y; Hinds, P S; Wang, J; Correia, H; Du, S; Ding, J; Gao, W J; Yuan, C<br>Cancer Nursing // 2013;36(5):368-376<br>2013 //<br>DOI: 10.1097/NCC.0b013e3182962701	Is a translation article
Assessing responsiveness over time of the PROMIS® pediatric symptom and function measures in cancer, nephrotic syndrome, and sickle cell disease<br>Reeve, B B; Edwards, L J; Jaeger, B C; Hinds, P S; Dampier, C; Gipson, D S; Selewski, D T; Troost, J P; Thissen, D; Barry, V; Gross, H E; DeWalt, D A<br>Quality of Life Research // 2018;27(1):249-257<br>2018 //<br>DOI: 10.1007/s11136-017-1697-z	Not exclusive chronic pain population
Integration of Electronic Patient-Reported Outcomes (ePROs) into pediatric clinic settings across hematology/oncology/bone marrow transplant<br>Myrvik, M P; Beverung, L M; Panepinto, J A; Igler, E C; Englebert, N; Bingen, K M<br>Clinical Practice in Pediatric Psychology // 2014;2(1):39-49<br>2014 //<br>DOI: 10.1037/cpp0000052	Not exclusive chronic pain population
Establishing clinical meaning and defining important differences for Patient-Reported Outcomes Measurement Information System (PROMIS®) measures in juvenile idiopathic arthritis using standard setting with patients, parents, and providers<br>Morgan, E M; Mara, C A; Huang, B; Barnett, K; Carle, A C; Farrell, J E; Cook, K F	Not exclusive chronic pain population

Quality of Life Research // 2017;26(3):565-586<br>2017 //<br>DOI: 10.1007/s11136-016-1468-2	
Qualitative Evaluation of Pediatric Pain Behavior, Quality, and Intensity Item Candidates and the PROMIS Pain Domain Framework in Children with Chronic Pain<br>Jacobson Jr., C J; Kashikar-Zuck, S; Farrell, J; Barnett, K; Goldschneider, K; Dampier, C; Cunningham, N; Crosby, L; Dewitt, E M<br>Journal of Pain // 2015;16(12):1243-1255<br>Department of Anthropology, University of Cincinnati, College of Arts and Sciences, Cincinnati, OH, United States Churchill Livingstone Inc. 2015 //<br>DOI: 10.1016/j.jpain.2015.08.007	Qualitative study
Initial Evaluation of the Pediatric PROMIS® Health Domains in Children and Adolescents With Sickle Cell Disease<br>Dampier, C; Barry, V; Gross, H E; Lui, Y; Thornburg, C D; Dewalt, D A; Reeve, B B<br>Pediatric Blood and Cancer // 2016;63(6):1031-1037<br>2016 //<br>DOI: 10.1002/pbc.25944	Not exclusive chronic pain population
Differential item functioning in the Patient Reported Outcomes Measurement Information System Pediatric Short Forms in a sample of children and adolescents with cerebral palsy<br>Coster, W J; Ni, P; Slavin, M D; Kisala, P A; Nandakumar, R; Mulcahey, M J; Tulsky, D S; Jette, A M<br>Developmental Medicine and Child Neurology // 2016;58(11):1132-1138<br>2016 //<br>DOI: 10.1111/dmcn.13138	Not exclusive chronic pain population
Development of six PROMIS pediatrics proxy-report item banks<br>Irwin, D E; Gross, H E; Stucky, B D; Thissen, D; DeWitt, E M; Lai, J S; Amtmann, D; Khastou, L; Varni, J W; DeWalt, D A<br>Health and Quality of Life Outcomes // 2012;10():<br>2012 //<br>DOI: 10.1186/1477-7525-10-22	Not exclusive chronic pain population
Estimating minimally important difference (MID) in PROMIS pediatric measures using the scale-judgment method<br>Thissen, D; Liu, Y; Magnus, B; Quinn, H; Gipson, D S; Dampier, C; Huang, I.-C.; Hinds, P S; Selewski, D T; Reeve, B B; Gross, H E; DeWalt, D A<br>Quality of Life Research // 2016;25(1):13-23<br>2016 //	Not exclusive chronic pain population

DOI: 10.1007/s11136-015-1058-8	
The use of PROMIS and assessment center to deliver Patient-Reported Outcome Measures in clinical research<br>Gershon, R C; Rothrock, N; Hanrahan, R; Bass, M; Cella, D<br>Journal of Applied Measurement // 2010;11(3):304-314<br>2010 //	Not exclusive chronic pain population
Parental Proxy PROMIS Pain Interference Scores are only Modestly Concordant with Their Child's Scores: An Effect of Child Catastrophizing<br>Scott, E L; Foxen-Craft, E; Caird, M; Philliben, R; Desebour, T; Currier, E; Voepel-Lewis, T<br>Clinical Journal of Pain // 2020;36(1):1-7<br>2020 //<br>DOI: 10.1097/AJP.0000000000000772	Not exclusive chronic pain population
Psychometric properties of the PROMIS® pediatric scales: Precision, stability, and comparison of different scoring and administration options<br>Varni, J W; Magnus, B; Stucky, B D; Liu, Y; Quinn, H; Thissen, D; Gross, H E; Huang, I.-C.; Dewalt, D A<br>Quality of Life Research // 2014;23(4):1233-1243<br>2014 //<br>DOI: 10.1007/s11136-013-0544-0	Not exclusive chronic pain population
The role of trait mindfulness in the pain experience of adolescents<br>Petter, M; Chambers, C T; McGrath, P J; Dick, B D<br>Journal of Pain // 2013;14(12):1709-1718<br>2013 //<br>DOI: 10.1016/j.jpain.2013.08.015	Not exclusive chronic pain population
Item-level informant discrepancies between children and their parents on the PROMIS® pediatric scales<br>Varni, J W; Thissen, D; Stucky, B D; Liu, Y; Magnus, B; He, J; DeWitt, E M; Irwin, D E; Lai, J.-S.; Amtmann, D; DeWalt, D A<br>Quality of Life Research // 2015;24(8):1921-1937<br>2015 //<br>DOI: 10.1007/s11136-014-0914-2	Not exclusive chronic pain population
PROMIS Pediatric Pain Interference Scale: An Item Response Theory Analysis of the Pediatric Pain Item Bank	Not exclusive chronic pain population

Varni, James W; Stucky, Brian D; Thissen, David; Dewitt, Esi Morgan; Irwin, Debra E; Lai, Jin-Shei; Yeatts, Karin; Dewalt, Darren A<br>Journal of Pain // 2010;11(11):1109-1119<br>2010 //<br>DOI: 10.1016/j.jpain.2010.02.005	
Item-level informant discrepancies between children and their parents on the PROMISA (R) pediatric scales<br>Varni, James W; Thissen, David; Stucky, Brian D; Liu, Yang; Magnus, Brooke; He, Jason; DeWitt, Esi Morgan; Irwin, Debra E; Lai, Jin-Shei; Amtmann, Dagmar; DeWalt, Darren A<br>QUALITY OF LIFE RESEARCH 08// 2015;24(8):1921-1937<br>VAN GODEWIJCKSTRAAT 30, 3311 GZ DORDRECHT, NETHERLANDS SPRINGER 2015 08//<br>DOI: 10.1007/s11136-014-0914-2	Does not utilize or assess tool
Using item response theory to enrich and expand the PROMIS® pediatric self report banks<br>Quinn, H; Thissen, D; Liu, Y; Magnus, B; Lai, J.-S.; Amtmann, D; Varni, J W; Gross, H E; DeWalt, D A<br>Health and Quality of Life Outcomes // 2014;12(1):<br>2014 //<br>DOI: 10.1186/s12955-014-0160-x	Not exclusive chronic pain population
PROMIS® parent proxy report scales for children ages 5-7 years: An item response theory analysis of differential item functioning across age groups<br>Varni, J W; Thissen, D; Stucky, B D; Liu, Y; Magnus, B; Quinn, H; Irwin, D E; Dewitt, E M; Lai, J.-S.; Amtmann, D; Gross, H E; Dewalt, D A<br>Quality of Life Research // 2014;23(1):349-361<br>2014 //<br>DOI: 10.1007/s11136-013-0439-0	Not exclusive chronic pain population
PROMIS® parent proxy report scales: An item response theory analysis of the parent proxy report item banks<br>Varni, J W; Thissen, D; Stucky, B D; Liu, Y; Gorder, H; Irwin, D E; DeWitt, E M; Lai, J.-S.; Amtmann, D; DeWalt, D A<br>Quality of Life Research // 2012;21(7):1223-1240<br>2012 //<br>DOI: 10.1007/s11136-011-0025-2	Not exclusive chronic pain population
Psychometric properties of the PROMISA (R) pediatric scales: precision, stability, and comparison of different scoring and administration options	Not exclusive chronic pain population

Varni, James W; Magnus, Brooke; Stucky, Brian D; Liu, Yang; Quinn, Hally; Thissen, David; Gross, Heather E; Huang, I-Chan; DeWalt, Darren A<br>QUALITY OF LIFE RESEARCH 05// 2014;23(4):1233-1243<br>VAN GODEWIJCKSTRAAT 30, 3311 GZ DORDRECHT, NETHERLANDS SPRINGER 2014 05//<br>DOI: 10.1007/s11136-013-0544-0	
The Adolescent Knee Pain (AK-Pain) prognostic tool: protocol for a prospective cohort study<br>Andreucci, Alessandro; Holden, Sinead; Bach Jensen, Martin; Skovdal Rathleff, Michael<br>F1000Research 2019;8():2148-2148<br>F1000 Research Ltd 2019<br>DOI: 10.12688/f1000research.21740.1	Does not utilize or assess tool
Using nationally representative percentiles to interpret PROMIS pediatric measures<br>Carle, Adam C.; Bevans, Katherine B.; Tucker, Carole A.; Forrest, Christopher B.<br>Quality of Life Research 2021;30(4):997-1004<br>Springer Science and Business Media Deutschland GmbH 2021<br>DOI: 10.1007/s11136-020-02700-5	Not exclusive chronic pain population
Parent cognitive, behavioural, and affective factors and their relation to child pain and functioning in pediatric chronic pain: A systematic review and meta-analysis<br>Donnelly, Theresa J.; Palermo, Tonya M.; Newton-John, Toby R.O.<br>Pain 2020;161(7):1401-1419<br>Lippincott Williams and Wilkins 2020<br>DOI: 10.1097/j.pain.0000000000001833	Not an original study
Towards an Effective Patient Health Engagement System Using Cloud-Based Text Messaging Technology<br>Cheng, Chih Wen; Brown, Clark R.; Venugopalan, Janani; Wang, May D.<br>IEEE Journal of Translational Engineering in Health and Medicine 2020;8():<br>Institute of Electrical and Electronics Engineers Inc. 2020<br>DOI: 10.1109/JTEHM.2018.2868358	Not exclusive chronic pain population
Randomized clinical trial of Fibromyalgia Integrative Training (FIT teens) for adolescents with juvenile fibromyalgia – Study design and protocol<br>Kashikar-Zuck, Susmita; Briggs, Matthew S.; Bout-Tabaku, Sharon; Connelly, Mark; Daffin, Morgan; Guite, Jessica; Ittenbach, Richard; Logan, Deirdre E.; Lynch-Jordan, Anne M.; Myer, Gregory D.; Ounpuu, Sylvia; Peugh, James; Schikler, Kenneth; Sugimoto, Dai; Stinson, Jennifer N.; Ting, Tracy V.; Thomas, Staci; Williams, Sara E.; Zempsky, William<br>Contemporary Clinical Trials 2021;103():	Not exclusive chronic pain population

Elsevier Inc. 2021<br>DOI: 10.1016/j.cct.2021.106321	
Risk models for predicting the health-related quality of life of caregivers of youth with gastrointestinal concerns<br>Lynch, Mary K.; Thompson, Kathryn A.; Dimmitt, Reed A.; Barnes, Margaux J.; Goodin, Burel R.<br>Quality of Life Research 2020;29(12):3343-3351<br>Springer Science and Business Media Deutschland GmbH 2020<br>DOI: 10.1007/s11136-020-02601-7	Not exclusive chronic pain population
Pain and internalizing symptoms in youth with gastrointestinal conditions including recurrent abdominal pain, eosinophilic esophagitis, and gastroesophageal reflux disease<br>Lynch, Mary K.; Thompson, Kathryn A.; Dimmitt, Reed A.; Barnes, Margaux J.; Goodin, Burel R.<br>Children's Health Care 2021;50(1):28-43<br>Routledge 2021<br>DOI: 10.1080/02739615.2020.1810575	Not exclusive chronic pain population
Natural Language Processing and Machine Learning Methods to Characterize Unstructured Patient-Reported Outcomes: Validation Study<br>Lu, Zhaohua; Sim, Jin-ah; Wang, Jade X; Forrest, Christopher B; Krull, Kevin R; Srivastava, Deokumar; Hudson, Melissa M; Robison, Leslie L; Baker, Justin N; Huang, I-Chan<br>Journal of Medical Internet Research 2021;23(11):e26777-e26777<br>JMIR Publications Inc. 2021<br>DOI: 10.2196/26777	Not exclusive chronic pain population
Development and Psychometric Evaluation of the PROMIS Pediatric Pain Intensity Measure in Children and Adolescents with Chronic Pain<br>Mara, Constance A.; Kashikar-Zuck, Susmita; Cunningham, Natoshia; Goldschneider, Kenneth R.; Huang, Bin; Dampier, Carlton; Sherry, David D.; Crosby, Lori; Farrell Miller, Jennifer; Barnett, Kimberly; Morgan, Esi M.<br>Journal of Pain 2021;22(1):48-56<br>Churchill Livingstone Inc. 2021<br>DOI: 10.1016/j.jpain.2020.04.001	Not exclusive chronic pain population
Utility of the PROMIS pediatric pain interference scale in juvenile fibromyalgia<br>Fussner, L M; Black, W R; Lynch-Jordan, A; Morgan, E M; Ting, T V; Kashikar-Zuck, S<br>Journal of Pediatric Psychology // 2019;44(4):436-441<br>2019 //	Not exclusive chronic pain population

DOI: 10.1093/jpepsy/jsy110	
Development and validation of the self-reported PROMIS pediatric pain behavior item bank and short form scale<br>Cunningham, Natoshia R.; Kashikar-Zuck, Susmita; Mara, Constance; Goldschneider, Kenneth R.; Revicki, Dennis A.; Dampier, Carlton; Sherry, David D.; Crosby, Lori; Carle, Adam; Cook, Karon F.; Morgan, Esi M.<br>PAIN 07// 2017;158(7):1323-1331<br>2017 07//<br>DOI: 10.1097/j.pain.0000000000000914	Not exclusive chronic pain population
Pediatric chronic postsurgical pain and functional disability: A prospective study of risk factors up to one year after major surgery<br>Rosenbloom, B N; Pagé, M G; Isaac, L; Campbell, F; Stinson, J N; Wright, J G; Katz, J<br>Journal of Pain Research // 2019;12():3079-3098<br>2019 //<br>DOI: 10.2147/JPR.S210594	Not pediatric population

Tool 3 Child Activity Limitations Questionnaire (n=23 citations excluded)

Chronic non-cancer pain in children: We have a problem, but also solutions<br>Vega, E; Beaulieu, Y; Gauvin, R; Ferland, C; Stabile, S; Pitt, R; Gonzalez Cardenas, V H; Ingelmo, P M<br>Minerva Anestesiologica // 2018;84(9):1081-1092<br>Chronic Pain Service, Department of Anesthesia, Montreal Children's Hospital, McGill University Health Center, 1001 Boulevard Decarie, Montreal, QC H4A 3J1, Canada Edizioni Minerva Medica 2018 //<br>DOI: 10.23736/S0375-9393.18.12367-4	Does not utilize or assess tool
Chronic pain assessment tools for cerebral palsy: A systematic review<br>Kingsnorth, S; Orava, T; Provvidenza, C; Adler, E; Ami, N; Gresley-Jones, T; Mankad, D; Slonim, N; Fay, L; Joachimides, N; Hoffman, A; Hung, R; Fehlings, D<br>Pediatrics // 2015;136(4):e947-e960<br>Evidence to Care, Holland Bloorview Kids Rehabilitation Hospital, 150 Kilgour Rd, Toronto, ON M4G 1R8, Canada American Academy of Pediatrics 2015 //<br>DOI: 10.1542/peds.2015-0273	Not an original study
Chronic Pain Assessments in Children and Adolescents: A Systematic Literature Review of the Selection, Administration, Interpretation, and Reporting of Unidimensional Pain Intensity Scales<br>Lee, R R; Rashid, A; Ghio, D; Thomson, W; Cordingley, L<br>Pain Research and Management // 2017;2017():	Does not utilize or assess tool

NIHR Manchester Biomedical Research Centre, Central Manchester University Hospitals NHS Foundation Trust, Manchester Academic Health Science Centre, University of Manchester, Manchester, M139PT, United Kingdom Hindawi Limited 2017 //<br>DOI: 10.1155/2017/7603758	
Predicting parent health-related quality of life: evaluating conceptual models<br>Defenderfer, E K; Rybak, T M; Davies, W H; Berlin, K S<br>Quality of Life Research // 2017;26(6):1405-1415<br>Psychology Department, University of Wisconsin-Milwaukee, 2441 E Hartford Ave., 413, Milwaukee, WI 53201, United States Springer International Publishing 2017 //<br>DOI: 10.1007/s11136-016-1491-3	Not exclusive chronic pain population
School functioning and chronic pain: A review of Method s and measures<br>Gorodzinsky, A Y; Hainsworth, K R; Weisman, S J<br>Journal of Pediatric Psychology // 2011;36(9):991-1002<br>Department of Psychology, University of Wisconsin-Milwaukee, Milwaukee, WI 53211, United States 2011 //<br>DOI: 10.1093/jpepsy/jsr038	Does not utilize or assess tool
Evidence-based assessment of health-related quality of life and functional impairment in pediatric psychology<br>Palermo, T M; Long, A C; Lewandowski, A S; Drotar, D; Quittner, A L; Walker, L S<br>Journal of Pediatric Psychology // 2008;33(9):983-996<br>Oregon Health and Science University 2008 //<br>DOI: 10.1093/jpepsy/jsn038	Not an original study
Patient versus parental perceptions about pain and disability in children and adolescents with a variety of chronic pain conditions<br>Vetter, T R; Bridgewater, C L; Ascherman, L I; Madan-Swain, A; McGwin Jr., G L<br>Pain Research and Management // 2014;19(1):7-14<br>Department of Anesthesiology, University of Alabama, School of Medicine, 619 19th Street South, Birmingham, AL 35249-6810, United States Hindawi Limited 2014 //<br>DOI: 10.1155/2014/736053	Does not utilize or assess tool
Pain perception of adolescents with chronic functional pain. Adaptation and psychometric validation of the pain perception scale (SES) by geissner<br>Wager, J; Tietze, A.-L.; Denecke, H; Schroeder, S; Vocks, S; Kosfelder, J; Zernikow, B; Hechler, T<br>Schmerz // 2010;24(3):236-250<br>Vodafone Stiftungsinstitut und Lehrst. fur Kinderschmerztherapie und Padiatrische Pallia-Tivmedizin, Vestische Kinder- und Jugendklinik Datteln, Universität Witten/Herdecke, Dr.-Friedrich-Steiner Str. 5, 45711 Datteln, Germany 2010 //	Not English or French

DOI: 10.1007/s00482-010-0920-4	
Measuring adolescents' HRQoL via self reports and parent proxy reports: An evaluation of the psychometric properties of both versions of the KINDL-R instrument<br>Erhart, M; Ellert, U; Kurth, B.-M.; Ravens-Sieberer, U<br>Health and Quality of Life Outcomes // 2009;7():77-77<br>Child Public Health, Department of Psychosomatics in Children and Adolescents, University Medical Center Hamburg-Eppendorf, Martinistr 52, D-20246 Hamburg, Germany 2009 //<br>DOI: 10.1186/1477-7525-7-77	Does not utilize or assess tool
Utility of the PROMIS pediatric pain interference scale in juvenile fibromyalgia<br>Fussner, L M; Black, W R; Lynch-Jordan, A; Morgan, E M; Ting, T V; Kashikar-Zuck, S<br>Journal of Pediatric Psychology // 2019;44(4):436-441<br>2019 //<br>DOI: 10.1093/jpepsy/jsy110	Does not utilize or assess tool
Pain-related disability in adolescents suffering from chronic pain: Preliminary examination of the Pediatric Pain Disability Index (P-PDI)<br>Hübner, B; Hechler, T; Dobe, M; Damschen, U; Kosfelder, J; Denecke, H; Schroeder, S; Zernikow, B<br>Schmerz // 2009;23(1):20-32<br>Vodafone Stiftungsinstitut für Kinderschmerztherapie und Pädiatrische Palliativmedizin, Vestische Kinder- und Jugendklinik Datteln, Universität Witten/Herdecke, Datteln 2009 //<br>DOI: 10.1007/s00482-008-0730-0	Not English or French
The prevalence, impact and cost of chronic non-cancer pain in Irish primary schoolchildren (PRIME-C): Protocol for a longitudinal school-based survey<br>O'Higgins, S; Doherty, E; NicGabhainn, S; Murphy, A; Hogan, M; O'Neill, C; McGuire, B E<br>BMJ Open // 2015;5(5):<br>Centre for Pain Research, School of Psychology, National University of Ireland, Galway, Ireland BMJ Publishing Group 2015 //<br>DOI: 10.1136/bmjopen-2014-007426	Does not utilize or assess tool
Bidirectional associations between pain and physical activity in adolescents<br>Rabbitts, J A; Holley, A L; Karlson, C W; Palermo, T M<br>Clinical Journal of Pain // 2014;30(3):251-258<br>Department of Anesthesiology and Pain Medicine, Seattle Children's Hospital and University of Washington School of Medicine, 4800 Sand Point Way NE, Seattle, WA 98105, United States 2014 //<br>DOI: 10.1097/AJP.0b013e31829550c6	Does not utilize or assess tool

Pain assessment-can it be done with a computerised system? A systematic review and meta-analysis<br>Pombo, N; Garcia, N; Bousson, K; Spinsante, S; Chorbev, I<br>International Journal of Environmental Research and Public Health // 2016;13(4):<br>Instituto de Telecomunicações (Telecommunications Institute), University of Beira Interior, Covilhã, 6200-001, Portugal MDPI AG 2016 //<br>DOI: 10.3390/ijerph13040415	Not an original study
PROMIS Pediatric Pain Interference Scale: An Item Response Theory Analysis of the Pediatric Pain Item Bank<br>Varni, James W; Stucky, Brian D; Thissen, David; Dewitt, Esi Morgan; Irwin, Debra E; Lai, Jin-Shei; Yeatts, Karin; Dewalt, Darren A<br>Journal of Pain // 2010;11(11):1109-1119<br>2010 //<br>DOI: 10.1016/j.jpain.2010.02.005	Does not utilize or assess tool
The Effect of Pain Catastrophizing on Outcomes: A Developmental Perspective Across Children, Adolescents, and Young Adults With Chronic Pain<br>Feinstein, A B; Sturgeon, J A; Darnall, B D; Dunn, A L; Rico, T; Kao, M C; Bhandari, R P<br>Journal of Pain // 2017;18(2):144-154<br>2017 //<br>DOI: 10.1016/j.jpain.2016.10.009	Does not utilize or assess tool
Co-occurring chronic pain and obesity in children and adolescents: The impact on health-related quality of life<br>Hainsworth, K R; Davies, W H; Khan, K A; Weisman, S J<br>Clinical Journal of Pain // 2009;25(8):715-721<br>Medical College of Wisconsin, United States Lippincott Williams and Wilkins 2009 //<br>DOI: 10.1097/AJP.0b013e3181a3b689	Does not utilize or assess tool
The Contribution of Parent and Family Variables to the Well-Being of Youth With Arthritis<br>Knafl, K; Leeman, J; Havill, N L; Crandell, J L; Sandelowski, M<br>Journal of Family Nursing // 2015;21(4):579-616<br>University of North Carolina, Chapel Hill, United States SAGE Publications Inc. 2015 //<br>DOI: 10.1177/1074840715601475	Not an original study
The CALI-9: A brief measure for assessing activity limitations in children and adolescents with chronic pain<br>Holley, A L; Zhou, C; Wilson, A C; Hainsworth, K; Palermo, T M	Does not utilize or assess tool

Pain // 2018;159(1):48-56<br>2018 //<br>DOI: 10.1097/j.pain.0000000000001063	
Validation of a self-report questionnaire version of the Child Activity Limitations Interview (CALI): The CALI-21<br>Palermo, T M; Lewandowski, A S; Long, A C; Burant, C J<br>Pain // 2008;139(3):644-652<br>Department of Anesthesiology and Peri-Operative Medicine, Oregon Health and Science University, 3181 SW Sam Jackson Park Road, Portland, OR 97239-3098, United States 2008 //<br>DOI: 10.1016/j.pain.2008.06.022	Does not utilize or assess tool
Parent Pain Responses as Predictors of Daily Activities and Mood in Children with Juvenile Idiopathic Arthritis: The Utility of Electronic Diaries<br>Connelly, M; Anthony, K K; Sarniak, R; Bromberg, M H; Gil, K M; Schanberg, L E<br>Journal of Pain and Symptom Management // 2010;39(3):579-590<br>Children's Mercy Hospitals and Clinics, Kansas City, MO, United States 2010 //<br>DOI: 10.1016/j.jpainsymman.2009.07.013	Not exclusive chronic pain population
Use of smartphones to prospectively evaluate predictors and outcomes of caregiver responses to pain in youth with chronic disease<br>Connelly, M; Bromberg, M H; Anthony, K K; Gil, K M; Schanberg, L E<br>Pain // 2017;158(4):629-636<br>Division of Developmental and Behavioral Sciences, Children's Mercy Hospital, 2401 Gillham Rd, Kansas City, MO 64108, United States Lippincott Williams and Wilkins 2017 //<br>DOI: 10.1097/j.pain.0000000000000804	Not exclusive chronic pain population
Quantitative sensory testing in adolescents with co-occurring chronic pain and obesity: A pilot study<br>Hainsworth, Keri R.; Simpson, Pippa M.; Ali, Omar; Varadarajan, Jaya; Rusy, Lynn; Weisman, Steven J.<br>Children 2020;7(6):<br>MDPI 2020<br>DOI: 10.3390/children7060055	Does not utilize or assess tool

Tool 4 Pain Experience Questionnaire (n=26 citations excluded)

Cortical correlates of an attentional bias to painful and innocuous somatic stimuli in children with recurrent abdominal pain<br>Hermann, C; Zohsel, K; Hohmeister, J; Flor, H	Does not utilize or assess tool

Pain // 2008;136(3):397-406<br>Department of Clinical and Cognitive Neuroscience, University of Heidelberg, Central Institute of Mental Health, Square J5, D-68159 Mannheim, Germany 2008 //<br>DOI: 10.1016/j.pain.2008.01.007	
Social risk and resilience factors in adolescent chronic pain: Examining the role of parents and Peers<br>Ross, A C; Simons, L E; Feinstein, A B; Yoon, I A; Bhandari, R P<br>Journal of Pediatric Psychology // 2018;43(3):303-313<br>2018 //<br>DOI: 10.1093/jpepsy/jsx118	Does not utilize or assess tool
Toward a taxonomy of adolescents with chronic pain: Exploratory cluster and discriminant analyses of the bath adolescent pain questionnaire<br>Vowles, K E; Jordan, A; Eccleston, C<br>European Journal of Pain // 2010;14(2):214-221<br>Centre for Pain Research, School for Health, University of Bath, Norwood House, Claverton Down, Bath BA2 7AY, United Kingdom 2010 //<br>DOI: 10.1016/j.ejpain.2009.05.004	Does not utilize or assess tool
Assessment of chronic pain in children: Current status and emerging topics<br>Palermo, T M<br>Pain Research and Management // 2009;14(1):21-26<br>Department of Anesthesiology and Peri-Operative Medicine, Oregon Health and Science University, 3181 SW Sam Jackson Park Road, Portland, OR 97239, United States Hindawi Limited 2009 //<br>DOI: 10.1155/2009/236426	Not an original study
Measuring musculoskeletal pain in infants, children, and adolescents<br>Michaleff, Z A; Kamper, S J; Stinson, J N; Hestbaek, L; Williams, C M; Campbell, P; Dunn, K M<br>Journal of Orthopaedic and Sports Physical Therapy // 2017;47(10):712-730<br>2017 //<br>DOI: 10.2519/jospt.2017.7469	Not an original study
Medication usage, emotional disturbance, and pain behavior in chronic low back pain patients<br>McCreary, C; Colman, A<br>Journal of Clinical Psychology // 1984;40(1):15-19<br>U. C. L. A. School of Medicine, United States 1984 //<br>DOI: 10.1002/1097-4679(198401)40:1<15::AID-JCLP2270400103>3.0.CO;2-J	Does not utilize or assess tool
Racial and ethnic disparities in cancer pain management.	Does not utilize or assess tool

Stephenson, N; Dalton, J A; Carlson, J; Youngblood, R; Bailey, D<br>Journal of National Black Nurses' Association : JNBNA // 2009;20(1):11-18<br>East Carolina University College of Nursing, Greenville, NC, USA. 2009 //	
Assessment of pain anxiety, pain catastrophizing, and fear of pain in children and adolescents with chronic pain: A systematic review and meta-analysis<br>Fisher, E; Heathcote, L C; Eccleston, C; Simons, L E; Palermo, T M<br>Journal of Pediatric Psychology // 2018;43(3):314-325<br>Center for Child Health, Behavior, and Development, Seattle Children's Research Institute, Seattle, WA, United States Oxford University Press 2018 //<br>DOI: 10.1093/jpepsy/jsx103	Does not utilize or assess tool
The roles of ethnicity, sex, and parental pain modeling in rating of experienced and imagined pain events<br>Boissoneault, J; Bunch, J R; Robinson, M<br>Journal of Behavioral Medicine // 2015;38(5):809-816<br>Pain Research and Intervention Center of Excellence, University of Florida, Gainesville, FL, United States Springer New York LLC 2015 //<br>DOI: 10.1007/s10865-015-9650-5	Not exclusive chronic pain population
Development and preliminary validation of the child pain anxiety symptoms scale in a community sample<br>Pagé, M G; Fuss, S; Martin, A L; Escobar, E M R; Katz, J<br>Journal of Pediatric Psychology // 2010;35(10):1071-1082<br>Department of Psychology, BSB 232, York University, 4700 Keele Street, Toronto, ON M3J 1P3, Canada 2010 //<br>DOI: 10.1093/jpepsy/jsq034	Does not utilize or assess tool
Disentangling the complex relations among caregiver and adolescent responses to adolescent chronic pain<br>Vowles, K E; Cohen, L L; McCracken, L M; Eccleston, C<br>Pain // 2010;151(3):680-686<br>Interdisciplinary Musculoskeletal Pain Assessment and Community Treatment Service, Haywood Hospital, Keele University, Stoke-on-Trent, United Kingdom 2010 //<br>DOI: 10.1016/j.pain.2010.08.031	Does not utilize or assess tool
What do pain scales measure in patients with rheumatoid arthritis?<br>Curio, I; Scholz, O B<br>Der Schmerz // 1990;4(4):207-213<br>Psychologisches Institut Lehrstuhl für Klinische, und Angewandte Psychologie der Universität, Römerstraße 164, Bonn 1, W-5300, Germany Springer-Verlag 1990 //	Not English or French

DOI: 10.1007/BF02527905	
Paediatric chronic pain<br>Rolfe, P M<br>Anaesthesia and Intensive Care Medicine // 2019;20(10):539-542<br>NHS Foundation Trust, United Kingdom Elsevier Ltd 2019 //<br>DOI: 10.1016/j.mpaic.2019.07.010	Not an original study
Somatic pain sensitivity in children with recurrent abdominal pain<br>Zohsel, K; Hohmeister, J; Flor, H; Hermann, C<br>American Journal of Gastroenterology // 2008;103(6):1517-1523<br>Department of Clinical and Cognitive Neuroscience, University of Heidelberg, Central Institute of Mental Health, Mannheim, Germany 2008 //<br>DOI: 10.1111/j.1572-0241.2008.01911.x	Does not include at least one of: Tool Development and/ or Psychometric Evaluation
The impact of parental gender, catastrophizing and situational threat upon parental behaviour to child pain: A vignette study<br>Goubert, L; Vervoort, T; De Ruddere, L; Crombez, G<br>European Journal of Pain (United Kingdom) // 2012;16(8):1176-1184<br>Department of Experimental-Clinical and Health Psychology, Ghent University, Belgium 2012 //<br>DOI: 10.1002/j.1532-2149.2012.00116.x	Does not utilize or assess tool
Dyadic analysis of child and parent trait and state pain catastrophizing in the process of children's pain communication<br>Birnie, K A; Chambers, C T; Chorney, J; Fernandez, C V; McGrath, P J<br>Pain // 2016;157(4):938-948<br>Department of Psychology and Neuroscience, Dalhousie University, Halifax, NS, Canada Lippincott Williams and Wilkins 2016 //<br>DOI: 10.1097/j.pain.0000000000000461	Does not utilize or assess tool
Chronic postsurgical pain in children: Prevalence and risk factors. A prospective observational study<br>Batoz, H; Semjen, F; Bordes-Demolis, M; Bnard, A; Nouette-Gaulain, K<br>British Journal of Anaesthesia // 2016;117(4):489-496<br>CHU de Bordeaux, Service d'Anesthsie Ṕdiatrique, Hôpital Pellegrin, Place Aḿlie Raba Ĺon, Bordeaux, F-33076, France Oxford University Press 2016 //<br>DOI: 10.1093/bja/aew260	Does not utilize or assess tool

Pain and its Impact on the Functional Ability in Children Treated at the Children's Cancer Center of Lebanon<br>Madi, D; Clinton, M<br>Journal of Pediatric Nursing // 2018;39():e11-e20<br>Hariri School of Nursing, American University of Beirut, Hamra-Bliss Street, Beirut, Lebanon W.B. Saunders 2018 //<br>DOI: 10.1016/j.pedn.2017.12.004	Does not utilize or assess tool
Rasch Analysis of the Arabic Language Version of the Functional Disability Inventory<br>Madi, Dina; Clinton, Michael<br>Journal of Pediatric Oncology Nursing // 2015;32(4):230-239<br>Los Angeles, CA 2015 //<br>DOI: 10.1177/1043454214554010	Does not utilize or assess tool
Anxiety, coping, and disability: A test of mediation in a pediatric chronic pain sample<br>Kaczynski, K J; Simons, L E; Claar, R L<br>Journal of Pediatric Psychology // 2011;36(8):932-941<br>Pain Treatment Service, Children's Hospital, Boston, 300 Longwood Ave, Boston, MA 02115, United States 2011 //<br>DOI: 10.1093/jpepsy/jsr024	Does not utilize or assess tool
Evaluation of Psychometric and Linguistic Properties of the Italian Adolescent Pain Assessment Scales: A Systematic Review<br>Marti, Flavio; Paladini, Antonella; Varrassi, Giustino; Latina, Roberto<br>PAIN AND THERAPY 06// 2018;7(1):77-104<br>GEWERBESTRASSE 11, CHAM, CH-6330, SWITZERLAND SPRINGER INTERNATIONAL PUBLISHING AG 2018 06//<br>DOI: 10.1007/s40122-018-0093-x	Not an original study
Age-related differences among adults coping with pain: Evaluation of a developmental life-context model<br>Lachapelle, D L; Hadjistavropoulos, T<br>Canadian Journal of Behavioural Science // 2005;37(2):123-137<br>University of New Brunswick, Canada American Psychological Association Inc. 2005 //<br>DOI: 10.1037/h0087250	Does not utilize or assess tool
Influence of culture on cancer pain management in hispanic patients<br>Juarez, G; Ferrell, B; Borneman, T<br>Cancer Practice // 1998;6(5):262-269<br>City of Hope National Medical Center, Duarte, CA, United States 1998 //	Does not utilize or assess tool

DOI: 10.1046/j.1523-5394.1998.00020.x	
The relationship between pain experience with mindfulness and psychological hardiness in chronic patients<br>Shaykh, Ameneh; Anvari, Najla<br>MEDICAL SCIENCE // 2018;22(93):468-472<br>KANYAKUMARI DISTRICT, TAMILNADU, 00000, INDIA DISCOVERY PUBLICATION 2018 //	Does not utilize or assess tool
Sex Differences in Prior Pain Experience<br>Stutts, L A; McCulloch, R C; Chung, K; Robinson, M E<br>Journal of Pain // 2009;10(12):1226-1230<br>Center for Pain Research and Behavioral Health, Department of Clinical and Health Psychology, University of Florida, PO Box 100165, Gainesville, FL 32610-0165, United States 2009 //<br>DOI: 10.1016/j.jpain.2009.04.016	Does not utilize or assess tool
Parent cognitive, behavioural, and affective factors and their relation to child pain and functioning in pediatric chronic pain: A systematic review and meta-analysis<br>Donnelly, Theresa J.; Palermo, Tonya M.; Newton-John, Toby R.O.<br>Pain 2020;161(7):1401-1419<br>Lippincott Williams and Wilkins 2020<br>DOI: 10.1097/j.pain.0000000000001833	Not an original study

Tool 5 Pain Interference Index (n=217 citations excluded)

Patient-reported outcomes of pain and physical functioning in neurofibromatosis clinical trials<br>Wolters, P L; Martin, S; Merker, V L; Tonsgard, J H; Solomon, S E; Baldwin, A; Bergner, A L; Walsh, K; Thompson, H L; Gardner, K L; Hingtgen, C M; Schorry, E; Dudley, W N; Franklin, B<br>Neurology // 2016;87(7):S4-S12<br>Pediatric Oncology Branch, National Cancer Institute, National Institutes of Health, Bethesda, MD, United States Lippincott Williams and Wilkins 2016 //<br>DOI: 10.1212/WNL.0000000000002927	Not an original study
Anxiety, coping, and disability: A test of mediation in a pediatric chronic pain sample<br>Kaczynski, K J; Simons, L E; Claar, R L<br>Journal of Pediatric Psychology // 2011;36(8):932-941<br>Pain Treatment Service, Children's Hospital, Boston, 300 Longwood Ave, Boston, MA 02115, United States 2011 //<br>DOI: 10.1093/jpepsy/jsr024	Does not utilize or assess tool

Latent Class Analysis of the Short and Long Forms of the Chronic Pain Acceptance Questionnaire: Further Examination of Patient Subgroups<br>Rovner, G; Vowles, K E; Gerdle, B; Gillanders, D<br>Journal of Pain // 2015;16(11):1095-1105<br>Division of Rehabilitation Medicine, Section for Highly Specialized Pain Rehabilitation, Department of Clinical Sciences, Danderyd Hospital, Karolinska Institutet, Stockholm, Sweden Churchill Livingstone Inc. 2015 //<br>DOI: 10.1016/j.jpain.2015.07.007	Does not utilize or assess tool
The effectiveness of acceptance and commitment therapy (ACT) on the anxiety, depression and psychological well-being of patients with hypothyroidism<br>Fakharian, N; Samari Safa, J; Ghezelsefloo, M<br>Iranian Journal of Endocrinology and Metabolism // 2019;21(2):65-72<br>Department of Guidance & Counseling, Faculty of Humanities, Islamic Azad University Khomeinishahr Branch, Isfahan, Iran Endocrine Research Center 2019 //	Not English or French
Systematic review and meta-analysis of psychological therapies for children with chronic pain<br>Fisher, E; Heathcote, L; Palermo, T M; De C. Williams, A C; Lau, J; Eccleston, C<br>Journal of Pediatric Psychology // 2014;39(8):763-782<br>Centre for Pain Research, University of Bath, Bath, BA2 7AY, United Kingdom Oxford University Press 2014 //<br>DOI: 10.1093/jpepsy/jsu008	Does not utilize or assess tool
Pain Acceptance in Adolescents: Development of a Short Form of the CPAQ-A<br>Gauntlett-Gilbert, J; Alamire, B; Duggan, G B<br>Journal of Pediatric Psychology // 2019;44(4):453-462<br>2019 //<br>DOI: 10.1093/jpepsy/jsy090	Does not utilize or assess tool
Internet-Delivered Acceptance and Values-Based Exposure Treatment for Fibromyalgia: A Pilot Study<br>Ljótsson, B; Atterlöf, E; Lagerlöf, M; Andersson, E; Jernelöv, S; Hedman, E; Kemani, M; Wicksell, R K<br>Cognitive Behaviour Therapy // 2014;43(2):93-104<br>Department of Clinical Neuroscience, Division of Psychology, Karolinska Institutet, Stockholm, Sweden Taylor and Francis A.S. 2014 //<br>DOI: 10.1080/16506073.2013.846401	Does not utilize or assess tool
Acceptance of pain in adolescents with chronic pain: Validation of an adapted assessment instrument and preliminary correlation analyses<br>McCracken, L M; Gauntlett-Gilbert, J; Eccleston, C<br>European Journal of Pain // 2010;14(3):316-320	Does not utilize or assess tool

Bath Centre for Pain Services, Royal National Hospital for Rheumatic Diseases, Centre for Pain Research, Bath, BA1 1RL, United Kingdom 2010 //<br>DOI: 10.1016/j.ejpain.2009.05.002	
The psychological flexibility model: A basis for integration and progress in psychological approaches to chronic pain management<br>McCracken, L M; Morley, S<br>Journal of Pain // 2014;15(3):221-234<br>2014 //<br>DOI: 10.1016/j.jpain.2013.10.014	Does not utilize or assess tool
Processes of change in psychological flexibility in an interdisciplinary group-based treatment for chronic pain based on Acceptance and Commitment Therapy<br>McCracken, L M; Gutiérrez-Martínez, O<br>Behaviour Research and Therapy // 2011;49(4):267-274<br>2011 //<br>DOI: 10.1016/j.brat.2011.02.004	Does not utilize or assess tool
Somatoform and related disorders<br>Elena Garralda, M; Rask, C U<br>Rutter's Child and Adolescent Psychiatry: Sixth Edition // 2015;():1035-1054<br>Academic Unit of Child and Adolescent Psychiatry, Imperial College London, London, United Kingdom John Wiley and Sons Ltd 2015 //<br>DOI: 10.1002/9781118381953.ch72	Does not utilize or assess tool
The use of functional neuroimaging to evaluate psychological and other non-pharmacological treatments for clinical pain<br>Jensen, K B; Berna, C; Loggia, M L; Wasan, A D; Edwards, R R; Gollub, R L<br>Neuroscience Letters // 2012;520(2):156-164<br>Department of Psychiatry, Massachusetts General Hospital (MGH), Harvard Medical School (HMS), Boston, MA, United States 2012 //<br>DOI: 10.1016/j.neulet.2012.03.010	Does not utilize or assess tool
One-day behavioral intervention in depressed migraine patients: Effects on headache<br>Dindo, L; Recober, A; Marchman, J; O'Hara, M W; Turvey, C<br>Headache // 2014;54(3):528-538<br>Department of Psychiatry, University of Iowa College of Medicine, Psychiat. Res. MEB 2-203, Iowa City, IA 52242-1000, United States Blackwell Publishing Inc. 2014 //	Does not utilize or assess tool

DOI: 10.1111/head.12258	
Acceptance and commitment therapy (ACT) to foster resilience in pediatric chronic illness<br>Ernst, M M; Mellon, M W<br>Child and Adolescent Resilience Within Medical Contexts: Integrating Research and Practice // 2016;():193-207<br>Department of Pediatrics, Division of Behavioral Medicine and Clinical Psychology, Cincinnati Children's Hospital Medical Center, University of Cincinnati College of Medicine, Cincinnati, OH, United States Springer International Publishing 2016 //<br>DOI: 10.1007/978-3-319-32223-0_11	Does not utilize or assess tool
Pain Intensity, Psychological Inflexibility, and Acceptance of Pain as Predictors of Functioning in Adolescents with Juvenile Idiopathic Arthritis: A Preliminary Investigation<br>Feinstein, Amanda B; Forman, Evan M; Masuda, Akihiko; Cohen, Lindsey L; Herbert, James D; Moorthy, L Nandini; Goldsmith, Donald P<br>JOURNAL OF CLINICAL PSYCHOLOGY IN MEDICAL SETTINGS 09// 2011;18(3):291-298<br>233 SPRING ST, NEW YORK, NY 10013 USA SPRINGER/PLENUM PUBLISHERS 2011 09//<br>DOI: 10.1007/s10880-011-9243-6	Does not utilize or assess tool
The Co-occurrence of Pediatric Chronic Pain and Anxiety: A Theoretical Review of a Developmentally Informed Shared Vulnerability Model<br>Jastrowski Mano, K E; O'bryan, E M; Gibler, R C; Beckmann, E<br>Clinical Journal of Pain // 2019;35(12):989-1002<br>Department of Psychology, University of Cincinnati, 5130D Edwards One, ML 0376, Cincinnati, OH 45221-0376, United States Lippincott Williams and Wilkins 2019 //<br>DOI: 10.1097/AJP.0000000000000763	Does not utilize or assess tool
Acceptation and Commitment Therapy (ACT) and Mindfulness, a model of psychological flexibility for chronic pain<br>Masselin-Dubois, A<br>Douleurs // 2016;17(5):233-251<br>2016 //<br>DOI: 10.1016/j.douler.2016.08.003	Cannot locate full text
A Case Study: Acceptance and Commitment Therapy for Pediatric Sickle Cell Disease<br>Masuda, Akihiko; Cohen, Lindsey L; Wicksell, Rikard K; Kemani, Mike K; Johnson, Alcuin<br>JOURNAL OF PEDIATRIC PSYCHOLOGY 05// 2011;36(4):398-408<br>JOURNALS DEPT, 2001 EVANS RD, CARY, NC 27513 USA OXFORD UNIV PRESS INC 2011 05//<br>DOI: 10.1093/jpepsy/jsq118	Does not utilize or assess tool

Comparing the effectiveness of mindfulness-based stress reduction and multidisciplinary intervention programs for chronic pain: A randomized comparative trial<br>Wong, S.Y.-S.; Chan, F.W.-K.; Wong, R.L.-P.; Chu, M.-C.; Kitty Lam, Y.-Y.; Mercer, S W; Ma, S H<br>Clinical Journal of Pain // 2011;27(8):724-734<br>School of Public Health and Primary Care, Centre for Population and Health Sciences, University of Glasgow, United Kingdom Lippincott Williams and Wilkins 2011 //<br>DOI: 10.1097/AJP.0b013e3182183c6e	Does not utilize or assess tool
Evolving the future: Toward a science of intentional change<br>Wilson, D S; Hayes, S C; Biglan, A; Embry, D D<br>Behavioral and Brain Sciences // 2014;89(3):1-99<br>SUNY, Departments of Biology and Anthropology, Binghamton University, Binghamton, NY 13903, United States Cambridge University Press 2014 //<br>DOI: 10.1017/S0140525X13001593	Does not utilize or assess tool
Acceptance and Commitment Therapy (ACT): The foundation of the therapeutic model and an overview of its contribution to the treatment of patients with chronic physical diseases<br>Prevedini, A B; Presti, G; Rabitti, E; Miselli, G; Moderato, P<br>Giornale Italiano di Medicina del Lavoro ed Ergonomia // 2011;33(1 SUPPL. A):A53-A63<br>IULM University-Milan-Italy, IESCUM, ACT-Italia, Italy 2011 //	Does not utilize or assess tool
Physician Complicity in the Transformation of Pain Medicine from a "Profession" to a "Business": Strategies for Reversing a Growing Trend<br>Schatman, M E<br>Pain Medicine (United States) // 2012;13(9):1149-1151<br>Foundation for Ethics in Pain Care, Bellevue, WA, United States Blackwell Publishing Inc. 2012 //<br>DOI: 10.1111/j.1526-4637.2012.01464.x	Does not utilize or assess tool
The efficacy of group-based acceptance and commitment therapy on psychological capital and school engagement: A pilot study among Chinese adolescents<br>Fang, S; Ding, D<br>Journal of Contextual Behavioral Science // 2020;16():134-143<br>Department of Psychology, Anhui Normal University, Wuhu, China Elsevier Inc. 2020 //<br>DOI: 10.1016/j.jcbs.2020.04.005	Does not utilize or assess tool
Risk and Resilience in Pediatric Pain<br>Feinstein, A B; Sturgeon, J A; Bhandari, R P; Yoon, I A; Ross, A C; Huestis, S E; Griffin, A T; Simons, L E<br>Clinical Journal of Pain // 2018;34(12):1096-1105	Does not utilize or assess tool

2018 //<br>DOI: 10.1097/AJP.0000000000000639	
Pain and its Impact on the Functional Ability in Children Treated at the Children's Cancer Center of Lebanon<br>Madi, D; Clinton, M<br>Journal of Pediatric Nursing // 2018;39():e11-e20<br>Hariri School of Nursing, American University of Beirut, Hamra-Bliss Street, Beirut, Lebanon W.B. Saunders 2018 //<br>DOI: 10.1016/j.pedn.2017.12.004	Does not utilize or assess tool
Pain and emotion: A biopsychosocial review of recent research<br>Lumley, M A; Cohen, J L; Borszcz, G S; Cano, A; Radcliffe, A M; Porter, L S; Schubiner, H; Keefe, F J<br>Journal of Clinical Psychology // 2011;67(9):942-968<br>Wayne State University, United States 2011 //<br>DOI: 10.1002/jclp.20816	Does not utilize or assess tool
Catastrophizing, pain, and functional outcomes for children with chronic pain: A meta-analytic review<br>Miller, M M; Meints, S M; Hirsh, A T<br>Pain // 2018;159(12):2442-2460<br>Department of Anesthesiology, Pain Management Center, Brigham and Women's Hospital, Harvard Medical School, Chestnut Hill, MA, United States Lippincott Williams and Wilkins 2018 //<br>DOI: 10.1097/j.pain.0000000000001342	Does not utilize or assess tool
Toward understanding acceptance and psychological flexibility in chronic pain<br>McCracken, L M<br>Pain // 2010;149(3):420-421<br>Centre for Pain Services, Royal National Hospital for Rheumatic Diseases, Centre for Pain Research, Bath, BA1 1RL, United Kingdom 2010 //<br>DOI: 10.1016/j.pain.2010.02.036	Does not utilize or assess tool
Pain Beliefs and Quality of Life in Young People with Disabilities and Bothersome Pain<br>Miró, J; Solé, E; Gertz, K; Jensen, M P; Engel, J M<br>Clinical Journal of Pain // 2017;33(11):998-1005<br>Pediatric Pain URV-Fundación Grünenthal, Unit for the Study and Treatment of Pain, ALGOS, Catalonia, Spain Lippincott Williams and Wilkins 2017 //<br>DOI: 10.1097/AJP.0000000000000482	Does not utilize or assess tool

Quantitative and qualitative testing of DARWeb: An online self-guided intervention for children with functional abdominal pain and their parents<br>Nieto, R; Boixadós, M; Hernández, E; Beneitez, I; Huguet, A; McGrath, P<br>Health Informatics Journal // 2019;25(4):1511-1527<br>Universitat Oberta de Catalunya, Spain SAGE Publications Ltd 2019 //<br>DOI: 10.1177/1460458218779113	Does not utilize or assess tool
A systematic review of randomised controlled trials using psychological interventions for children and adolescents with medically unexplained symptoms: A focus on mental health outcomes<br>O'Connell, C; Shafran, R; Bennett, S<br>Clinical Child Psychology and Psychiatry // 2020;25(1):273-290<br>Department of Applied Psychology, Canterbury Christ Church University, Kent, United Kingdom SAGE Publications Ltd 2020 //<br>DOI: 10.1177/1359104519855415	Does not utilize or assess tool
Pain in Children<br>Palermo, T M; Koh, J L; Zeltzer, L K<br>Clinical Pain Management: A Practical Guide // 2010;():319-325<br>Seattle Children's Hospital, University of Washington School of Medicine, Seattle, United States Wiley-Blackwell 2010 //<br>DOI: 10.1002/9781444329711.ch38	Does not utilize or assess tool
A Mindfulness Program Adapted for Adolescents with Chronic Pain: Feasibility, Acceptability, and Initial Outcomes<br>Ruskin, D A; Gagnon, M M; Kohut, S A; Stinson, J N; Walker, K S<br>Clinical Journal of Pain // 2017;33(11):1019-1029<br>Department of Anesthesia and Pain Medicine, Hospital for Sick Children, 555 University Avenue, Toronto, ON M5G 1X8, Canada Lippincott Williams and Wilkins 2017 //<br>DOI: 10.1097/AJP.0000000000000490	Does not utilize or assess tool
A group-based, acceptance & commitment therapy intervention for chronic pain<br>Cosio, D<br>Social Work with Groups // 2019;():<br>Department of Anesthesiology/Pain Clinic, Jesse Brown VA Medical Center, Chicago, IL, United States Routledge 2019 //<br>DOI: 10.1080/01609513.2019.1604290	Does not utilize or assess tool
A meta-analysis of the efficacy of acceptance and commitment therapy for children<br>Fang, S; Ding, D<br>Journal of Contextual Behavioral Science // 2020;15():225-234<br>Department of Psychology, Anhui Normal University, Wuhu, China Elsevier Inc. 2020 //	Does not utilize or assess tool

DOI: 10.1016/j.jcbs.2020.01.007	
Acute and chronic pain in children: Role of the parents?<br>Goubert, L; Vervoort, T<br>Tijdschrift voor Geneeskunde // 2014;70(21):1240-1248<br>Vakgroep Experimenteel-Klinische en Gezondheidspsychologie, Faculteit Psychologie en Pedagogische Wetenschappen, Universiteit Gent, Henri Dunantlaan 2, Gent, 9000, Belgium Tijdschrift voor Geneeskunde 2014 //<br>DOI: 10.2143/TVG.70.21.2001721	Cannot locate full text
The Effectiveness of Acceptance and Commitment Therapy for Adolescent Mental Health: Swedish and Australian Pilot Outcomes<br>Livheim, F; Hayes, L; Ghaderi, A; Magnusdottir, T; Högfeldt, A; Rowse, J; Turner, S; Hayes, S C; Tengström, A<br>Journal of Child and Family Studies // 2015;24(4):1016-1030<br>Department of Clinical Neuroscience, Karolinska Institutet, Stockholm, Sweden Springer New York LLC 2015 //<br>DOI: 10.1007/s10826-014-9912-9	Does not utilize or assess tool
Ecological system influences in the treatment of pediatric chronic pain<br>Logan, D E; BKin, L E; Feinstein, A B; Sieberg, C B; Sparling, P; Cohen, L L; Conroy, C; Driesman, D; Masuda, A<br>Pain Research and Management // 2012;17(6):407-411<br>Department of Psychiatry, Harvard Medical School, Division of Pain Medicine, Boston, MA, United States Hindawi Limited 2012 //<br>DOI: 10.1155/2012/289504	Does not utilize or assess tool
Role of psychological flexibility in parents of adolescents with chronic pain: Development of a measure and preliminary correlation analyses<br>McCracken, L M; Gauntlett-Gilbert, J<br>Pain // 2011;152(4):780-785<br>Centre for Pain Services, Royal National Hospital for Rheumatic Diseases, University of Bath, Bath, United Kingdom 2011 //<br>DOI: 10.1016/j.pain.2010.12.001	Does not utilize or assess tool
Pain-related anxiety in children and adolescents: Mind the gap<br>McCracken, L M<br>Pain // 2011;152(9):1938-1939<br>Centre for Pain Services, Royal National Hospital for Rheumatic Diseases, University of Bath, Bath BA1 1RL, United Kingdom Elsevier B.V. 2011 //<br>DOI: 10.1016/j.pain.2011.04.007	Does not utilize or assess tool

Interventions to improve the mental health of children and young people with long-term physical conditions: Linked evidence syntheses<br>Moore, D A; Nunns, M; Shaw, L; Rogers, M; Walker, E; Ford, T; Garside, R; Ukoumunne, O; Titman, P; Shafran, R; Heyman, I; Anderson, R; Dickens, C; Viner, R; Bennett, S; Logan, S; Lockhart, F; Coon, J T<br>Health Technology Assessment // 2019;23(22):1-164<br>Evidence Synthesis & Modelling for Health Improvement, University of Exeter Medical School, Exeter, United Kingdom NIHR Journals Library 2019 //<br>DOI: 10.3310/hta23220	Does not utilize or assess tool
The Tampa Scale of Kinesiophobia: Structural Validation among Adolescents with Idiopathic Scoliosis Undergoing Spinal Fusion Surgery<br>Ye, D.-L.; Plante, I; Roy, M; Ouellet, J A; Ferland, C E<br>Physical and Occupational Therapy in Pediatrics // 2020;():<br>Shriners Hospitals for Children-Canada, Montreal, Canada Taylor and Francis Ltd 2020 //<br>DOI: 10.1080/01942638.2020.1720054	Does not utilize or assess tool
Special considerations in conducting clinical trials of chronic pain management interventions in children and adolescents and their families<br>Palermo, T M; Kashikar-Zuck, S; Friedrichsdorf, S J; Powers, S W<br>Pain Reports // 2019;4(3):<br>Center for Child Health, Behavior, and Development, Seattle Children's Research Institute, Seattle, WA, United States Lippincott Williams and Wilkins 2019 //<br>DOI: 10.1097/PR9.0000000000000649	Does not utilize or assess tool
Reducing Stress and Enhancing Academic Buoyancy among Adolescents Using a Brief Web-based Program Based on Acceptance and Commitment Therapy: A Randomized Controlled Trial<br>Puolakanaho, A; Lappalainen, R; Lappalainen, P; Muotka, J S; Hirvonen, R; Eklund, K M; Ahonen, T P S; Kiuru, N<br>Journal of Youth and Adolescence // 2019;48(2):287-305<br>Department of Psychology, University of Jyväskylä, P.O. Box 35, Jyväskylä, 40014, Finland Springer New York LLC 2019 //<br>DOI: 10.1007/s10964-018-0973-8	Does not utilize or assess tool
Fear-avoidance beliefs and parental responses to pain in adolescents with chronic pain<br>Wilson, A C; Lewandowski, A S; Palermo, T M<br>Pain Research and Management // 2011;16(3):178-182<br>Child Development and Rehabilitation Center, Oregon Health and Science University, CDRC, 3181 Southwest Sam Jackson Park Road, Portland, OR 97239, United States Hindawi Limited 2011 //<br>DOI: 10.1155/2011/296298	Does not utilize or assess tool

Adolescent acceptance of pain: Confirmatory factor analysis and further validation of the chronic pain acceptance Questionnaire, Adolescent version<br>Wallace, D P; Harbeck-Weber, C; Whiteside, S P H; Harrison, T E<br>Journal of Pain // 2011;12(5):591-599<br>Children's Mercy Hospitals and Clinics, Developmental and Behavioral Sciences, 2401 Gillham Road, Kansas City, MO 64108, United States 2011 //<br>DOI: 10.1016/j.jpain.2010.11.004	Does not utilize or assess tool
Mindfulness Based Interventions for Youth<br>Zack, S; Saekow, J; Kelly, M; Radke, A<br>Journal of Rational - Emotive and Cognitive - Behavior Therapy // 2014;32(1):44-56<br>Department of Psychiatry and Behavioral Sciences, Stanford School of Medicine, 401 Quarry Rd., Stanford, CA, 94305, United States Springer New York LLC 2014 //<br>DOI: 10.1007/s10942-014-0179-2	Does not utilize or assess tool
Psychological flexibility as a mediator of improvement in Acceptance and Commitment Therapy for patients with chronic pain following whiplash<br>Wicksell, R K; Olsson, G L; Hayes, S C<br>European Journal of Pain // 2010;14(10):1059.e1-1059.e11<br>Behavior Medicine Pain Treatment Service, Karolinska University Hospital, 171 76 Stockholm, Sweden Blackwell Publishing Ltd 2010 //<br>DOI: 10.1016/j.ejpain.2010.05.001	Does not utilize or assess tool
Effectiveness of interdisciplinary interventions in paediatric chronic pain management: a systematic review and subset meta-analysis<br>Liossi, C; Johnstone, L; Lilley, S; Caes, L; Williams, G; Schoth, D E<br>British Journal of Anaesthesia // 2019;123(2):e359-e371<br>University of Southampton, School of Psychology, Southampton, United Kingdom Elsevier Ltd 2019 //<br>DOI: 10.1016/j.bja.2019.01.024	Does not utilize or assess tool
A case report and literature review of autism and attention deficit hyperactivity disorder in paediatric chronic pain<br>Wiwe Lipsker, C; von Heijne, M; Bölte, S; Wicksell, R K<br>Acta Paediatrica, International Journal of Paediatrics // 2018;107(5):753-758<br>Functional Area Medical Psychology, Functional Unit Behavioural Medicine, Karolinska University Hospital, Stockholm, Sweden Blackwell Publishing Ltd 2018 //<br>DOI: 10.1111/apa.14220	Does not utilize or assess tool

A validation of the pain interference index in adults with long-standing pain<br>Kemani, M K; Zetterqvist, V; Kanstrup, M; Holmström, L; Wicksell, R K<br>Acta Anaesthesiologica Scandinavica // 2016;60(2):250-258<br>Behavioural Medicine Pain Treatment Services, Pain Center, Karolinska University Hospital, Stockholm, 171 76, Sweden<br>Blackwell Munksgaard 2016 //<br>DOI: 10.1111/aas.12599	Not pediatric population
Efficacy and cost-effectiveness of acceptance and commitment therapy and applied relaxation for longstanding pain: A Randomized Controlled Trial<br>Kemani, M K; Olsson, G L; Lekander, M; Hesser, H; Andersson, E; Wicksell, R K<br>Clinical Journal of Pain // 2015;31(11):1004-1016<br>Behavioral Medicine Pain Treatment Services, Karolinska University Hospital, Stockholm, 171 76, Sweden Lippincott Williams and Wilkins 2015 //<br>DOI: 10.1097/AJP.0000000000000203	Does not utilize or assess tool
Prevalence of autism traits and attention-deficit hyperactivity disorder symptoms in a clinical sample of children and adolescents with chronic pain<br>Lipsker, C W; Bölte, S; Hirvikoski, T; Lekander, M; Holmström, L; Wicksell, R K<br>Journal of Pain Research // 2018;11():2827-2836<br>Functional Area Medical Psychology, Functional Unit Behavior Medicine, Karolinska University Hospital, Stockholm, Sweden<br>Dove Medical Press Ltd. 2018 //<br>DOI: 10.2147/JPR.S177534	Does not utilize or assess tool
Lessons learned from a mindfulness-based intervention with chronically ill youth<br>Lagor, A F; Williams, D J; Lerner, J B; McClure, K S<br>Clinical Practice in Pediatric Psychology // 2013;1(2):146-158<br>Department of Psychology, La Salle University, 1900 West Olney Avenue, Box 275, Philadelphia, PA 19141, United States<br>American Psychological Association Inc. 2013 //<br>DOI: 10.1037/cpp0000015	Does not utilize or assess tool
Pharmacodynamic Study of Miransertib in Individuals with Proteus Syndrome<br>Keppler-Noreuil, K M; Sapp, J C; Lindhurst, M J; Darling, T N; Burton-Akright, J; Bagheri, M; Dombi, E; Gruber, A; Jarosinski, P F; Martin, S; Nathan, N; Paul, S M; Savage, R E; Wolters, P L; Schwartz, B; Widemann, B C; Biesecker, L G<br>American Journal of Human Genetics // 2019;104(3):484-491<br>Medical Genomics and Metabolic Genetics Branch, National Human Genome Research Institute, NIH, Bethesda, MD 20892, United States Cell Press 2019 //<br>DOI: 10.1016/j.ajhg.2019.01.015	Not pediatric population

Clinical report: One year of treatment of Proteus syndrome with miransertib (ARQ 092)<br>Biesecker, L G; Edwards, M; O'Donnell, S; Doherty, P; MacDougall, T; Tith, K; Kazakin, J; Schwartz, B<br>Cold Spring Harbor Molecular Case Studies // 2020;6(1):<br>Medical Genomics and Metabolic Genetics Branch, National Human Genome Research Institute, National Institutes of Health, Bethesda, MD 20892, United States Cold Spring Harbor Laboratory Press 2020 //<br>DOI: 10.1101/mcs.a004549	Does not utilize or assess tool
Essentials of acceptance and commitment therapy<br>Batten, S V<br>Essentials of Acceptance and Commitment Therapy // 2011;():1-125<br>United States Department of Veterans Affairs (VA), United States SAGE Publications Inc. 2011 //<br>DOI: 10.4135/9781446251843	Not an original study
Fear reduction in patients with chronic pain: A learning theory perspective<br>Hollander, M D; De Jong, J R; Volders, S; Goossens, M E; Smeets, R J; Vlaeyen, J W<br>Expert Review of Neurotherapeutics // 2010;10(11):1733-1745<br>University Medical Centre Maastricht, Maastricht, Netherlands 2010 //<br>DOI: 10.1586/ern.10.115	Cannot locate full text
Exploring sleep problems in young children with cerebral palsy - A population-based study<br>Löwing, K; Gyllensvärd, M; Tedroff, K<br>European Journal of Paediatric Neurology // 2020;():<br>Department of Women's & Children's Health, Karolinska Institutet, Stockholm, Sweden W.B. Saunders Ltd 2020 //<br>DOI: 10.1016/j.ejpn.2020.06.006	Does not utilize or assess tool
Mindfulness and meditation for adolescents: Practices and programs<br>Wisner, B L<br>Mindfulness and Meditation for Adolescents: Practices and Programs // 2017;():1-174<br>Palgrave Macmillan 2017 //<br>DOI: 10.1057/978-1-349-95207-6	Not an original study
Acceptance and Commitment Therapy for children and adolescents with physical health concerns<br>Wicksell, R K; Kanstrup, M; Kemani, M K; Holmström, L; Olsson, G L<br>Current Opinion in Psychology // 2015;2():1-5<br>Behavior Medicine Pain Treatment Service, Karolinska University Hospital, Sweden Elsevier 2015 //<br>DOI: 10.1016/j.copsyc.2014.12.029	Does not utilize or assess tool
Mindfulness-based cognitive therapy for posttraumatic stress disorder	Not an original study

Sears, R M; Chard, K M<br>Mindfulness-Based Cognitive Therapy for Posttraumatic Stress Disorder // 2016;():1-187<br>American Board of Professional Psychology (ABPP), United States wiley 2016 //<br>DOI: 10.1002/9781118691403	
Comparing group-based acceptance and commitment therapy (ACT) with enhanced usual care for adolescents with functional somatic syndromes: A study protocol for a randomised trial<br>Kallesøe, K H; Schröder, A; Wicksell, R K; Fink, P; Ørnbøl, E; Rask, C U<br>BMJ Open // 2016;6(9):<br>Research Clinic for Functional Disorders and Psychosomatics, Aarhus University Hospital, Aarhus, Denmark BMJ Publishing Group 2016 //<br>DOI: 10.1136/bmjopen-2016-012743	Not an original study
A review of acceptance and commitment therapy (ACT) empirical evidence: Correlational, experimental psychopathology, component and outcome studies<br>Ruiz, F J<br>International Journal of Psychology and Psychological Therapy // 2010;10(1):125-162<br>Universidad de Almería, Spain 2010 //	Not English or French
Psychological issues in the management of pain<br>Covington, E C; Kotz, M M<br>The ASAM Principles of Addiction Medicine: Fifth Edition // 2014;():<br>Neurological Center for Pain, Cleveland Clinic Foundation, Cleveland, OH, United States Wolters Kluwer Health Adis (ESP) 2014 //	Not an original study
Development of Nurse-Led Pain Management Programmes: Meeting a Community need<br>Burrows, D<br>Advancing Nursing Practice in Pain Management // 2010;():143-161<br>PainConsultants Limited, United Kingdom Wiley-Blackwell 2010 //<br>DOI: 10.1002/9781444318722.ch9	Does not utilize or assess tool
Behavior Therapy: The Second and Third Waves<br>Ferguson, K E; O'Donohue, W<br>International Encyclopedia of the Social & Behavioral Sciences: Second Edition // 2015;():431-436<br>St. Peter Family Medicine Residency ProgramWA, United States Elsevier Inc. 2015 //<br>DOI: 10.1016/B978-0-08-097086-8.21090-8	Not an original study
Developing a stoma acceptance questionnaire to improve motivation to adhere to enterostoma self-care<br>Bagnasco, A; Watson, R; Zanini, M; Catania, G; Aleo, G; Sasso, L<br>Journal of Preventive Medicine and Hygiene // 2017;58(2):E190-E194	Does not utilize or assess tool

Department of Health Sciences, University of Genoa, Via Pastore 1, Genoa, 16132, Italy Pacini Editore S.p.A. 2017 //	
A Unified, Transdiagnostic Treatment for Adolescents With Chronic Pain and Comorbid Anxiety and Depression<br>Allen, L B; Tsao, J C I; Seidman, L C; Ehrenreich-May, J; Zeltzer, L K<br>Cognitive and Behavioral Practice // 2012;19(1):56-67<br>David Geffen School of Medicine, UCLA, United States 2012 //<br>DOI: 10.1016/j.cbpra.2011.04.007	Does not utilize or assess tool
Group CBT-yoga protocol targeting pain-related and internalizing symptoms in youth<br>Allen, T M; Wren, A A; Anderson, L M; Sabholk, A; Mauro, C F<br>Clinical Practice in Pediatric Psychology // 2018;6(1):7-18<br>Duke University, 2608 Erwin Road Suite 300, Durham, NC 27705, United States American Psychological Association Inc. 2018 //<br>DOI: 10.1037/cpp0000206	Does not utilize or assess tool
Pediatric fear-avoidance model of chronic pain: Foundation, application and future directions<br>Asmundson, G J G; Noel, M; Petter, M; Parkerson, H A<br>Pain Research and Management // 2012;17(6):397-405<br>Department of Psychology, University of Regina, 3737 Wascana Parkway, Regina, SK S4S 0A2, Canada Hindawi Limited 2012 //<br>DOI: 10.1155/2012/908061	Does not utilize or assess tool
Cross-Cultural Adaptation and Psychometric Properties of the European Portuguese Version of the Central Sensitization Inventory in Adolescents With Musculoskeletal Chronic Pain<br>Andias, R; Silva, A G<br>Pain Practice // 2020;20(5):480-490<br>School of Health Sciences, University of Aveiro, Aveiro, Portugal Blackwell Publishing Inc. 2020 //<br>DOI: 10.1111/papr.12875	Does not utilize or assess tool
Departing from the essential features of a high quality systematic review of psychotherapy: A response to Öst (2014) and recommendations for improvement<br>Atkins, P W B; Ciarrochi, J; Gaudiano, B A; Bricker, J B; Donald, J; Rovner, G; Smout, M; Livheim, F; Lundgren, T; Hayes, S C<br>Behaviour Research and Therapy // 2017;97():259-272<br>Institute for Positive Psychology and Education, Australian Catholic University, Strathfield, NSW 2135, Australia Elsevier Ltd 2017 //<br>DOI: 10.1016/j.brat.2017.05.016	Does not utilize or assess tool

Guided internet-delivered acceptance and commitment therapy for chronic pain patients: A randomized controlled trial<br>Buhrman, M; Skoglund, A; Husell, J; Bergström, K; Gordh, T; Hursti, T; Bendelin, N; Furmark, T; Andersson, G<br>Behaviour Research and Therapy // 2013;51(6):307-315<br>Department of Psychology, Uppsala University, Uppsala, Sweden Elsevier Ltd 2013 //<br>DOI: 10.1016/j.brat.2013.02.010	Does not utilize or assess tool
Children with chronic and life-limiting health conditions<br>Edwards, M; Baños, I<br>Cognitive Behaviour Therapy for Children and Families, Third Edition // 2013;():159-175<br>Paediatric Psychology Service, Evelina Children's Hospital, London, United Kingdom Cambridge University Press 2013 //<br>DOI: 10.1017/CBO9781139344456.016	Does not utilize or assess tool
Pain therapy with children and adolescents severely disabled due to chronic pain: Long-term outcome after inpatient pain therapy<br>Dobe, M; Hechler, T; Behlert, J; Kosfelder, J; Zernikow, B<br>Schmerz // 2011;25(4):411-422<br>Vodafone Stiftungsinstitut und Lehrstuhl fur Kinderschmerztherapie und Padiatrische Palliativmedizin, Vestische Kinder- und Jugendklinik Datteln, Universität Witten/Herdecke, Dr.-Friedrich-Steiner-Str. 5, Datteln 45711, Germany 2011 //<br>DOI: 10.1007/s00482-011-1051-2	Not English or French
Acceptance and commitment therapy<br>Ducasse, D; Fond, G<br>Encephale // 2015;41(1):1-9<br>Université Montpellier 1, Montpellier, 34000, France Elsevier Masson SAS 2015 //<br>DOI: 10.1016/j.encep.2013.04.017	Does not utilize or assess tool
A randomized controlled trial of strong minds: A school-based mental health program combining acceptance and commitment therapy and positive psychology<br>Burckhardt, R; Manicavasagar, V; Batterham, P J; Hadzi-Pavlovic, D<br>Journal of School Psychology // 2016;57():41-52<br>School of Psychiatry, University of NSW, Black Dog Institute, Australia Elsevier Ltd 2016 //<br>DOI: 10.1016/j.jsp.2016.05.008	Does not utilize or assess tool
Improving the mental health of adolescents with epilepsy through a group cognitive behavioral therapy program<br>Carbone, L; Plegue, M; Barnes, A; Shellhaas, R<br>Epilepsy and Behavior // 2014;39():1-1	Does not utilize or assess tool

Department of Social Work, University of Michigan Health System, Ann Arbor, MI, United States Academic Press Inc. 2014 //<br>DOI: 10.1016/j.yebeh.2014.07.024	
Treatments addressing pain-related fear and anxiety in patients with chronic musculoskeletal pain: A preliminary review<br>Bailey, K M; Carleton, R N; Vlaeyen, J W S; Asmundson, G J G<br>Cognitive Behaviour Therapy // 2010;39(1):46-63<br>Department of Psychology and the Anxiety and Illness Behaviours Laboratory, University of Regina, Regina, SK S4S 0A2, Canada 2010 //<br>DOI: 10.1080/16506070902980711	Does not utilize or assess tool
The effectiveness of group-based acceptance and commitment therapy on pain-related anxiety, acceptance of pain and pain intensity in patients with chronic pain<br>Anvari, M H; Ebrahimi, A; Neshatdoost, H T; Afshar, H; Abedi, A<br>Journal of Isfahan Medical School // 2014;32(295):1156-1165<br>Department of Psychology, School of Psychology and Education Sciences, University of Isfahan, Isfahan, Iran Isfahan University of Medical Sciences(IUMS) 2014 //	Does not utilize or assess tool
Psykosomatisk smärtdiagnos bör byggas på fastställda kriterier: Kan ge möjligheter till bättre vård<br>Alfvén, G<br>Lakartidningen // 2012;109(5):224-227<br>Hallunda Barn-och ungdomsmedicinsk mottagning (BUMM), Astrid Lindgrens barnsjukhus, Norsborg, Sweden 2012 //	Not English or French
Sex differences in the efficacy of psychological therapies for the management of chronic and recurrent pain in children and adolescents: A systematic review and meta-Analysis<br>Boerner, K E; Eccleston, C; Chambers, C T; Keogh, E<br>Pain // 2017;158(4):569-582<br>Department of Psychology and Neuroscience, Dalhousie University, 5850/5980 University Avenue, Halifax, NS B3K 6R8, Canada Lippincott Williams and Wilkins 2017 //<br>DOI: 10.1097/j.pain.0000000000000803	Does not utilize or assess tool
Resilience factors in children with juvenile idiopathic arthritis and their parents: The role of child and parent psychological flexibility<br>Beeckman, M; Hughes, S; Van Ryckeghem, D; Van Hoecke, E; Dehoorne, J; Joos, R; Goubert, L<br>Pain Medicine (United States) // 2019;20(6):1120-1131<br>Department of Experimental, Clinical and Health Psychology, Ghent University, H. Dunantlaan 2, Ghent, 9000, Belgium Oxford University Press 2019 //<br>DOI: 10.1093/pm/pny181	Does not utilize or assess tool

Psychological Interventions for Children with Functional Somatic Symptoms: A Systematic Review and Meta-Analysis<br>Bonvanie, I J; Kallesøe, K H; Janssens, K A M; Schröder, A; Rosmalen, J G M; Rask, C U<br>Journal of Pediatrics // 2017;187():272-281.e17<br>University Medical Center of Groningen, Interdisciplinary Center Psychopathology and Emotion Regulation, University of Groningen, Groningen, Netherlands Mosby Inc. 2017 //<br>DOI: 10.1016/j.jpeds.2017.03.017	Does not utilize or assess tool
Psychological therapies for the management of chronic and recurrent pain in children and adolescents<br>Eccleston, C; Palermo, T M; Williams, A C C; Lewandowski Holley, A; Morley, S; Fisher, E; Law, E<br>Cochrane Database of Systematic Reviews // 2014;2017(10):<br>University of Bath, Centre for Pain Research, Claverton Down, Bath, United Kingdom John Wiley and Sons Ltd 2014 //<br>DOI: 10.1002/14651858.CD003968.pub4	Does not utilize or assess tool
A systematic review of the use of acceptance and commitment therapy in supporting parents<br>Byrne, G; Ghráda, Á N Í; O'Mahony, T; Brennan, E<br>Psychology and Psychotherapy: Theory, Research and Practice // 2020;():<br>Health Service Executive, Dublin, Ireland Wiley-Blackwell 2020 //<br>DOI: 10.1111/papt.12282	Does not utilize or assess tool
Acceptance-based therapy: the potential to augment behavioral interventions in the treatment of type 2 diabetes<br>Cardel, M I; Ross, K M; Butryn, M; Donahoo, W T; Eastman, A; Dillard, J R; Grummon, A; Hopkins, P; Whigham, L D; Janicke, D<br>Nutrition and Diabetes // 2020;10(1):<br>Department of Health Outcomes and Biomedical Informatics, University of Florida, Gainesville, FL 32611, United States Springer Nature 2020 //<br>DOI: 10.1038/s41387-020-0106-9	Does not utilize or assess tool
Measurement framework for the Environmental influences on Child Health Outcomes research program<br>Blackwell, C K; Wakschlag, L S; Gershon, R C; Cella, D<br>Current Opinion in Pediatrics // 2018;30(2):276-284<br>Department of Medical Social Sciences, Feinberg School of Medicine, Northwestern University, 633 N. Saint Clair Street, 19th Floor, Chicago, IL 60611, United States Lippincott Williams and Wilkins 2018 //<br>DOI: 10.1097/MOP.0000000000000606	Does not utilize or assess tool
Comparison of motivational interviewing with acceptance and commitment therapy: A conceptual and clinical review<br>Bricker, J; Tollison, S	Does not utilize or assess tool

Behavioural and Cognitive Psychotherapy // 2011;39(5):541-559<br>University of Washington, Fred Hutchinson Cancer Research Center, 1100 Fairview Avenue North, M3-B232, Seattle, WA 98195, United States 2011 //<br>DOI: 10.1017/S1352465810000901	
Comparing paths to quality of life: Contributions of ACT and cognitive therapy intervention targets in two highly anxious samples<br>Berghoff, C R; Forsyth, J P; Ritzert, T R; Sheppard, S C<br>Journal of Contextual Behavioral Science // 2014;3(2):89-97<br>University at Albany, State University of New York, 1400 Washington Ave., Albany, NY 12222, United States Elsevier Inc. 2014 //<br>DOI: 10.1016/j.jcbs.2014.04.001	Does not utilize or assess tool
A Functional Contextualist Approach to Cultural Evolution: An Introduction to Part IV<br>Biglan, A<br>The Wiley Handbook of Contextual Behavioral Science // 2015;():383-397<br>Oregon Research Institute, United States Wiley Blackwell 2015 //<br>DOI: 10.1002/9781118489857.ch19	Does not utilize or assess tool
Coping and acceptance in chronic childhood conditions<br>Gauntlett-Gilbert, J; Connell, H<br>Psychologist // 2012;25(3):198-201<br>Bath Centre for Pain Services, University of Bath, United Kingdom 2012 //	Does not utilize or assess tool
Psychological Flexibility as a Resilience Factor in Individuals With Chronic Pain<br>Gentili, C; Rickardsson, J; Zetterqvist, V; Simons, L E; Lekander, M; Wicksell, R K<br>Frontiers in Psychology // 2019;10():<br>Functional Area Medical Psychology, Functional Unit Behavior Medicine, Karolinska University Hospital, Stockholm, Sweden Frontiers Media S.A. 2019 //<br>DOI: 10.3389/fpsyg.2019.02016	Not pediatric population
Chronic pain in children and young people<br>Goddard, J M<br>Current Opinion in Supportive and Palliative Care // 2011;5(2):158-163<br>Sheffield Children's Hospital, Western Bank, Sheffield, United Kingdom 2011 //<br>DOI: 10.1097/SPC.0b013e328345832d	Does not utilize or assess tool
ACTsmart – development and feasibility of digital Acceptance and Commitment Therapy for adults with chronic pain	Does not utilize or assess tool

Gentili, C; Zetterqvist, V; Rickardsson, J; Holmström, L; Simons, L E; Wicksell, R K<br>npj Digital Medicine // 2020;3(1):<br>Functional Unit Behavioral Medicine, Function Area Medical Psychology, Karolinska University Hospital, Stockholm, Sweden<br>Nature Research 2020 //<br>DOI: 10.1038/s41746-020-0228-4	
Acceptance of premonitory urges and tics<br>Gev, E; Pilowsky-Peleg, T; Fennig, S; Benaroya-Milshtein, N; Woods, D W; Piacentini, J; Apter, A; Steinberg, T<br>Journal of Obsessive-Compulsive and Related Disorders // 2016;10():78-83<br>Matta and Harry Freund Neuropsychiatry Tourette Syndrome and Tic Disorders Clinic, Schneider Children's Medical Center of Israel, Petach Tikva, 49202, Israel Elsevier B.V. 2016 //<br>DOI: 10.1016/j.jocrd.2016.06.001	Does not utilize or assess tool
Psychologycal and behavioral changes in chronic pain<br>Gavrilov, V; Pavlov, G<br>Anaesthesiology and Intensive Care // 2018;47(2):14-21<br>City Clinic Oncology Centre - Sofia, Dept of Anesthesiology and Intensive Care, Bulgaria Medical Information Center 2018 //	Does not locate full text
Acceptance and commitment therapy - Do we know enough? Cumulative and sequential meta-analyses of randomized controlled trials<br>Hacker, T; Stone, P; Macbeth, A<br>Journal of Affective Disorders // 2016;190():551-565<br>Universitätsmedizin Rostock, Klinik und Poliklinik für Psychiatrie und Psychotherapie Gehlsheimer, Straße 20 i, Rostock, 18147, Germany Elsevier 2016 //<br>DOI: 10.1016/j.jad.2015.10.053	Does not utilize or assess tool
Psychological treatment for adolescent depression: Perspectives on the past, present, and future<br>Hayes, L; Bach, P A; Boyd, C P<br>Behaviour Change // 2010;27(1):1-18<br>School of Behavioural and Social Sciences and Humanities, University of Ballarat, Australia Australian Academic Press 2010 //<br>DOI: 10.1375/bech.27.1.1	Does not utilize or assess tool
Psychosocial perspectives in the treatment of pediatric chronic pain<br>Carter, B D; Threlkeld, B M<br>Pediatric Rheumatology // 2012;10():<br>Division of Child, Adolescent and Family Psychiatry, University of Louisville School of Medicine, Bingham Clinic, 200 East Chestnut Street, Louisville, KY, 40202, United States 2012 //	Does not utilize or assess tool

DOI: 10.1186/1546-0096-10-15	
Development and Validation of the English Pain Interference Index and Pain Interference Index-Parent Report<br>Martin, S; Nelson Schmitt, S; Wolters, P L; Abel, B; Toledo-Tamula, M A; Baldwin, A; Wicksell, R K; Merchant, M; Widemann, B<br>Pain Medicine (United States) // 2015;16(2):367-373<br>National Cancer Institute, National Institutes of Health, Bethesda, MD, United States Blackwell Publishing Inc. 2015 //<br>DOI: 10.1111/pme.12620	Not pediatric population
Comparing the effectiveness of mindfulness-based stress reduction and multidisciplinary intervention programs for chronic pain: A randomized comparative trial<br>Wong, S.Y.-S.; Chan, F.W.-K.; Wong, R.L.-P.; Chu, M.-C.; Kitty Lam, Y.-Y.; Mercer, S W; Ma, S H<br>Clinical Journal of Pain // 2011;27(8):724-734<br>School of Public Health and Primary Care, Centre for Population and Health Sciences, University of Glasgow, United Kingdom Lippincott Williams and Wilkins 2011 //<br>DOI: 10.1097/AJP.0b013e3182183c6e	Does not utilize or assess tool
Mindfulness and meditation for adolescents: Practices and programs<br>Wisner, B L<br>Mindfulness and Meditation for Adolescents: Practices and Programs // 2017;():1-174<br>Palgrave Macmillan 2017 //<br>DOI: 10.1057/978-1-349-95207-6	Not AN original study
Children with chronic and life-limiting health conditions<br>Edwards, M; Baños, I<br>Cognitive Behaviour Therapy for Children and Families, Third Edition // 2013;():159-175<br>Paediatric Psychology Service, Evelina Children's Hospital, London, United Kingdom Cambridge University Press 2013 //<br>DOI: 10.1017/CBO9781139344456.016	
The effectiveness of group-based acceptance and commitment therapy on pain-related anxiety, acceptance of pain and pain intensity in patients with chronic pain<br>Anvari, M H; Ebrahimi, A; Neshatdoost, H T; Afshar, H; Abedi, A<br>Journal of Isfahan Medical School // 2014;32(295):1156-1165<br>Department of Psychology, School of Psychology and Education Sciences, University of Isfahan, Isfahan, Iran Isfahan University of Medical Sciences(IUMS) 2014 //	Does not utilize or assess tool
Acceptance and commitment therapy - Do we know enough? Cumulative and sequential meta-analyses of randomized controlled trials<br>Hacker, T; Stone, P; Macbeth, A	Does not utilize or assess tool

Journal of Affective Disorders // 2016;190():551-565<br>Universitätsmedizin Rostock, Klinik und Poliklinik für Psychiatrie und Psychotherapie Gehlsheimer, Straße 20 i, Rostock, 18147, Germany Elsevier 2016 //<br>DOI: 10.1016/j.jad.2015.10.053	
Applications and adaptations of Acceptance and Commitment Therapy (ACT) for adolescents<br>Halliburton, A E; Cooper, L D<br>Journal of Contextual Behavioral Science // 2015;4(1):1-11<br>Virginia Polytechnic Institute and State University (Virginia Tech), 109 Williams Hall, Blacksburg, VA 24060, United States Elsevier Inc. 2015 //<br>DOI: 10.1016/j.jcbs.2015.01.002	Does not utilize or assess tool
A comparative study of 2 manual-based self-help interventions, acceptance and commitment therapy and applied relaxation, for persons with chronic pain<br>Thorsell, J; Finnes, A; Dahl, J; Lundgren, T; Gybrant, M; Gordh, T; Buhrman, M<br>Clinical Journal of Pain // 2011;27(8):716-723<br>2011 //<br>DOI: 10.1097/AJP.0b013e318219a933	Does not utilize or assess tool
Further validation of the Chronic Pain Acceptance Questionnaire for Adolescents in a broader paediatric context<br>Connolly, S; Ferreira, N; McGarrigle, L; DeAmicis, L<br>Journal of Contextual Behavioral Science // 2019;12():314-321<br>University of Edinburgh, Department of Clinical and Health Psychology, United Kingdom Elsevier Inc. 2019 //<br>DOI: 10.1016/j.jcbs.2018.12.005	Does not utilize or assess tool
Cognitive-behavioral therapy, behavioral therapy, and related treatments in children<br>Kendall, P C; Peterman, J S; Cummings, C M<br>Rutter's Child and Adolescent Psychiatry: Sixth Edition // 2015;():496-509<br>Department of Psychology, Temple University, Philadelphia, PA, United States John Wiley and Sons Ltd 2015 //<br>DOI: 10.1002/9781118381953.ch38	Does not utilize/ assess tool
Circles of engagement: Childhood pain and parent brain<br>Simons, L E; Goubert, L; Vervoort, T; Borsook, D<br>Neuroscience and Biobehavioral Reviews // 2016;68():537-546<br>Center for Pain and the Brain, Boston Children's Hospital (BCH), Boston, United States Elsevier Ltd 2016 //<br>DOI: 10.1016/j.neubiorev.2016.06.020	Does not utilize/ assess tool

Health-related quality of life in youth with abdominal pain: An examination of optimism and pain self-efficacy<br>Tomlinson, Rachel M.; Bax, Kevin C.; Ashok, Dhandapani; McMurtry, C. Meghan<br>Journal of Psychosomatic Research 2021;147():<br>Elsevier Inc. 2021<br>DOI: 10.1016/j.jpsychores.2021.110531	Does not utilize/ assess tool
The psychological flexibility model: A basis for integration and progress in psychological approaches to chronic pain management<br>McCracken, L M; Morley, S<br>Journal of Pain // 2014;15(3):221-234<br>2014 //<br>DOI: 10.1016/j.jpain.2013.10.014	Does not utilize/ assess tool
The use of functional neuroimaging to evaluate psychological and other non-pharmacological treatments for clinical pain<br>Jensen, K B; Berna, C; Loggia, M L; Wasan, A D; Edwards, R R; Gollub, R L<br>Neuroscience Letters // 2012;520(2):156-164<br>Department of Psychiatry, Massachusetts General Hospital (MGH), Harvard Medical School (HMS), Boston, MA, United States<br>2012 //<br>DOI: 10.1016/j.neulet.2012.03.010	Does not utilize/ assess tool
The Co-occurrence of Pediatric Chronic Pain and Anxiety: A Theoretical Review of a Developmentally Informed Shared Vulnerability Model<br>Jastrowski Mano, K E; O'bryan, E M; Gibler, R C; Beckmann, E<br>Clinical Journal of Pain // 2019;35(12):989-1002<br>Department of Psychology, University of Cincinnati, 5130D Edwards One, ML 0376, Cincinnati, OH 45221-0376, United States<br>Lippincott Williams and Wilkins 2019 //<br>DOI: 10.1097/AJP.0000000000000763	Does not utilize/ assess tool
Clinical report: One year of treatment of Proteus syndrome with miransertib (ARQ 092)<br>Biesecker, L G; Edwards, M; O'Donnell, S; Doherty, P; MacDougall, T; Tith, K; Kazakin, J; Schwartz, B<br>Cold Spring Harbor Molecular Case Studies // 2020;6(1):<br>Medical Genomics and Metabolic Genetics Branch, National Human Genome Research Institute, National Institutes of Health, Bethesda, MD 20892, United States Cold Spring Harbor Laboratory Press 2020 //<br>DOI: 10.1101/mcs.a004549	Does not utilize/ assess tool
Development of Nurse-Led Pain Management Programmes: Meeting a Community need	Does not utilize/ assess tool

Burrows, D<br>Advancing Nursing Practice in Pain Management // 2010;():143-161<br>PainConsultants Limited, United Kingdom Wiley-Blackwell 2010 //<br>DOI: 10.1002/9781444318722.ch9	
A randomized controlled trial of strong minds: A school-based mental health program combining acceptance and commitment therapy and positive psychology<br>Burckhardt, R; Manicavasagar, V; Batterham, P J; Hadzi-Pavlovic, D<br>Journal of School Psychology // 2016;57():41-52<br>School of Psychiatry, University of NSW, Black Dog Institute, Australia Elsevier Ltd 2016 //<br>DOI: 10.1016/j.jsp.2016.05.008	Does not utilize/ assess tool
Psychological interventions for patients with cancer: Psychological flexibility and the potential utility of Acceptance and Commitment Therapy<br>Hulbert-Williams, N J; Storey, L; Wilson, K G<br>European Journal of Cancer Care // 2015;24(1):15-27<br>Department of Psychology, University of Chester, Chester, United Kingdom Blackwell Publishing Ltd 2015 //<br>DOI: 10.1111/ecc.12223	Does not utilize/ assess tool
Exploring changes in valued action in the presence of chronic debilitating pain in acceptance and commitment therapy for youth - A single-subject design study<br>Kemani, M K; Olsson, G L; Holmström, L; Wicksell, R K<br>Frontiers in Psychology // 2016;7(DEC):<br>Functional Unit Behavioral Medicine, Karolinska University Hospital, Stockholm, Sweden Frontiers Research Foundation 2016 //<br>DOI: 10.3389/fpsyg.2016.01984	Does not utilize/ assess tool
Prevalence, identification, and interference of pain in young children with cerebral palsy: a population-based study<br>Tedroff, K; Gyllensvärd, M; Löwing, K<br>Disability and Rehabilitation // 2019;():<br>Department of Women's and Children's Health, Karolinska Institutet, Stockholm, Sweden Taylor and Francis Ltd 2019 //<br>DOI: 10.1080/09638288.2019.1665719	Not exclusive chronic pain population
Systematic review and meta-analysis of psychological therapies for children with chronic pain<br>Fisher, E; Heathcote, L; Palermo, T M; De C. Williams, A C; Lau, J; Eccleston, C<br>Journal of Pediatric Psychology // 2014;39(8):763-782<br>Centre for Pain Research, University of Bath, Bath, BA2 7AY, United Kingdom Oxford University Press 2014 //	Does not utilize/ assess tool

DOI: 10.1093/jpepsy/jsu008	
Internet-Delivered Acceptance and Values-Based Exposure Treatment for Fibromyalgia: A Pilot Study<br>Ljótsson, B; Atterlöf, E; Lagerlöf, M; Andersson, E; Jernelöv, S; Hedman, E; Kemani, M; Wicksell, R K<br>Cognitive Behaviour Therapy // 2014;43(2):93-104<br>Department of Clinical Neuroscience, Division of Psychology, Karolinska Institutet, Stockholm, Sweden Taylor and Francis A.S. 2014 //<br>DOI: 10.1080/16506073.2013.846401	Does not utilize/ assess tool
Processes of change in psychological flexibility in an interdisciplinary group-based treatment for chronic pain based on Acceptance and Commitment Therapy<br>McCracken, L M; Gutiérrez-Martínez, O<br>Behaviour Research and Therapy // 2011;49(4):267-274<br>2011 //<br>DOI: 10.1016/j.brat.2011.02.004	Does not utilize/ assess tool
Pain Intensity, Psychological Inflexibility, and Acceptance of Pain as Predictors of Functioning in Adolescents with Juvenile Idiopathic Arthritis: A Preliminary Investigation<br>Feinstein, Amanda B; Forman, Evan M; Masuda, Akihiko; Cohen, Lindsey L; Herbert, James D; Moorthy, L Nandini; Goldsmith, Donald P<br>JOURNAL OF CLINICAL PSYCHOLOGY IN MEDICAL SETTINGS 09// 2011;18(3):291-298<br>233 SPRING ST, NEW YORK, NY 10013 USA SPRINGER/PLENUM PUBLISHERS 2011 09//<br>DOI: 10.1007/s10880-011-9243-6	Does not utilize/ assess tool
Ecological system influences in the treatment of pediatric chronic pain<br>Logan, D E; BKin, L E; Feinstein, A B; Sieberg, C B; Sparling, P; Cohen, L L; Conroy, C; Driesman, D; Masuda, A<br>Pain Research and Management // 2012;17(6):407-411<br>Department of Psychiatry, Harvard Medical School, Division of Pain Medicine, Boston, MA, United States Hindawi Limited 2012 //<br>DOI: 10.1155/2012/289504	Does not utilize/ assess tool
An mhealth intervention for persons with diabetes type 2 based on acceptance and commitment therapy principles: Examining treatment fidelity<br>Nes, A A G; Van Dulmen, S; Brembo, E A; Eide, H<br>Journal of Medical Internet Research // 2018;20(7):<br>Netherlands Institute for Health Services Research, Utrecht, Netherlands Journal of Medical Internet Research 2018 //<br>DOI: 10.2196/mhealth.9942	Does not utilize/ assess tool

Effectiveness of Acceptance and Commitment-Based Therapy (ACT Rehab) on quality of life, severity and duration of pain; in women with chronic low back pain<br>Mousavi, S M; Mujembari, A K; Abharian, P H; Pashang, S<br>Iranian Rehabilitation Journal // 2018;16(1):103-110<br>Department of Psychology, Karaj Branch, Islamic Azad University, Karaj, Iran University of Social Welfare and Rehabilitation Sciences 2018 //<br>DOI: 10.29252/NRIP.IRJ.16.1.103	Does not utilize/ assess tool
Rebuttal of Atkins et al. (2017) critique of the Öst (2014) meta-analysis of ACT<br>Öst, L.-G.<br>Behaviour Research and Therapy // 2017;97():273-281<br>Department of Psychology, Stockholm University, Sweden Elsevier Ltd 2017 //<br>DOI: 10.1016/j.brat.2017.08.008	Does not utilize/ assess tool
Evaluation of an intensive interdisciplinary pain treatment based on acceptance and commitment therapy for adolescents with chronic pain and their parents: A nonrandomized clinical trial<br>Kemani, M K; Kanstrup, M; Jordan, A; Caes, L; Gauntlett-Gilbert, J<br>Journal of Pediatric Psychology // 2018;43(9):981-994<br>Functional Area Medical Psychology, Functional Unit Behavioral Medicine, Karolinska University Hospital, Stockholm, 171 76, Sweden Oxford University Press 2018 //<br>DOI: 10.1093/jpepsy/jsy031	Does not utilize/ assess tool
Depression and Disability in Migraine: The Role of Pain Acceptance and Values-Based Action<br>Dindo, L; Recober, A; Marchman, J; O'Hara, M; Turvey, C<br>International Journal of Behavioral Medicine // 2015;22(1):109-117<br>2015 //<br>DOI: 10.1007/s12529-014-9390-x	Does not utilize/ assess tool
The children's health & illness recovery program (CHIRP): Feasibility and preliminary efficacy in a clinical sample of adolescents with chronic pain and fatigue<br>Carter, B D; Kronenberger, W G; Threlkeld, B; Townsend, A; Pruitt, A<br>Clinical Practice in Pediatric Psychology // 2013;1(2):184-195<br>University of Louisville Medical School-Pediatrics, Division of Child Psychiatry and Psychology, University of Louisville School of Medicine, 200 E. Chestnut Street, Louisville, KY 40202, United States American Psychological Association Inc. 2013 //<br>DOI: 10.1037/cpp0000012	Does not utilize/ assess tool
Psychological processing in chronic pain: A neural systems approach<br>Simons, L E; Elman, I; Borsook, D	Does not utilize/ assess tool

Neuroscience and Biobehavioral Reviews // 2014;39():61-78<br>Center for Pain and the Brain, P.A.I.N. Group, Boston Children's Hospital, United States 2014 //<br>DOI: 10.1016/j.neubiorev.2013.12.006	
A Case Study: Acceptance and Commitment Therapy for Pediatric Sickle Cell Disease<br>Masuda, Akihiko; Cohen, Lindsey L; Wicksell, Rikard K; Kemani, Mike K; Johnson, Alcuin<br>JOURNAL OF PEDIATRIC PSYCHOLOGY 05// 2011;36(4):398-408<br>JOURNALS DEPT, 2001 EVANS RD, CARY, NC 27513 USA OXFORD UNIV PRESS INC 2011 05//<br>DOI: 10.1093/jpepsy/jsq118	Does not utilize/ assess tool
Evolving the future: Toward a science of intentional change<br>Wilson, D S; Hayes, S C; Biglan, A; Embry, D D<br>Behavioral and Brain Sciences // 2014;89(3):1-99<br>SUNY, Departments of Biology and Anthropology, Binghamton University, Binghamton, NY 13903, United States Cambridge University Press 2014 //<br>DOI: 10.1017/S0140525X13001593	Does not utilize/ assess tool
A Mindfulness Program Adapted for Adolescents with Chronic Pain: Feasibility, Acceptability, and Initial Outcomes<br>Ruskin, D A; Gagnon, M M; Kohut, S A; Stinson, J N; Walker, K S<br>Clinical Journal of Pain // 2017;33(11):1019-1029<br>Department of Anesthesia and Pain Medicine, Hospital for Sick Children, 555 University Avenue, Toronto, ON M5G 1X8, Canada Lippincott Williams and Wilkins 2017 //<br>DOI: 10.1097/AJP.0000000000000490	Does not utilize/ assess tool
Psychological issues in the management of pain<br>Covington, E C; Kotz, M M<br>The ASAM Principles of Addiction Medicine: Fifth Edition // 2014;():<br>Neurological Center for Pain, Cleveland Clinic Foundation, Cleveland, OH, United States Wolters Kluwer Health Adis (ESP) 2014 //	Not an original study
Acceptance and Commitment Therapy with children and adolescents: A review<br>Moreno, P M; Blasco, R Q<br>International Journal of Psychology and Psychological Therapy // 2019;19(2):173-188<br>Universidad de Granada, Spain Universidad de Almeria 2019 //	Not English or French
Toward a taxonomy of adolescents with chronic pain: Exploratory cluster and discriminant analyses of the bath adolescent pain questionnaire<br>Vowles, K E; Jordan, A; Eccleston, C<br>European Journal of Pain // 2010;14(2):214-221	Does not utilize/ assess tool

Centre for Pain Research, School for Health, University of Bath, Norwood House, Claverton Down, Bath BA2 7AY, United Kingdom 2010 //<br>DOI: 10.1016/j.ejpain.2009.05.004	
The Fear of Pain Questionnaire (FOPQ): Assessment of Pain-Related Fear Among Children and Adolescents With Chronic Pain<br>Simons, Laura E; Sieberg, Christine B; Carpino, Elizabeth; Logan, Deirdre; Berde, Charles<br>Journal of Pain // 2011;12(6):677-686<br>2011 //<br>DOI: 10.1016/j.jpain.2010.12.008	Does not utilize/ assess tool
A group-based, acceptance & commitment therapy intervention for chronic pain<br>Cosio, David<br>Social Work with Groups 2020;43(4):334-346<br>Routledge 2020<br>DOI: 10.1080/01609513.2019.1604290	Does not utilize or assess tool
Understanding How Perfectionism Impacts Intensive Interdisciplinary Pain Treatment Outcomes: A Nonrandomized Trial<br>Randall, Edin T.; Cole-Lewis, Yasmin C.; Petty, Carter R.; Jervis, Kelsey N.<br>Journal of Pediatric Psychology 2021;46(3):351-362<br>Oxford University Press 2021<br>DOI: 10.1093/jpepsy/jsaa111	Does not utilize/ assess tool
Is Acceptance and Commitment Training or Therapy (ACT) a Method that Applied Behavior Analysts Can and Should Use?<br>Dixon, Mark R; Hayes, Steven C; Stanley, Caleb; Law, Stu; Thouraya Al-Nasser, &<br>;():<br>DOI: 10.1007/s40732-020-00436-9/Published	Does not utilize/ assess tool
Pain Acceptance in Adolescents: Development of a Short Form of the CPAQ-A<br>Gauntlett-Gilbert, J; Alamire, B; Duggan, G B<br>Journal of Pediatric Psychology // 2019;44(4):453-462<br>2019 //<br>DOI: 10.1093/jpepsy/jsy090	Does not utilize/ assess tool
Acceptance and Commitment Therapy (ACT): The foundation of the therapeutic model and an overview of its contribution to the treatment of patients with chronic physical diseases	Does not utilize/ assess tool

Prevedini, A B; Presti, G; Rabitti, E; Miselli, G; Moderato, P<br>Giornale Italiano di Medicina del Lavoro ed Ergonomia // 2011;33(1 SUPPL. A):A53-A63<br>IULM University-Milan-Italy, IESCUM, ACT-Italia, Italy 2011 //	
Quantitative and qualitative testing of DARWeb: An online self-guided intervention for children with functional abdominal pain and their parents<br>Nieto, R; Boixadós, M; Hernández, E; Beneitez, I; Huguet, A; McGrath, P<br>Health Informatics Journal // 2019;25(4):1511-1527<br>Universitat Oberta de Catalunya, Spain SAGE Publications Ltd 2019 //<br>DOI: 10.1177/1460458218779113	Does not utilize/ assess tool
Adolescent acceptance of pain: Confirmatory factor analysis and further validation of the chronic pain acceptance Questionnaire, Adolescent version<br>Wallace, D P; Harbeck-Weber, C; Whiteside, S P H; Harrison, T E<br>Journal of Pain // 2011;12(5):591-599<br>Children's Mercy Hospitals and Clinics, Developmental and Behavioral Sciences, 2401 Gillham Road, Kansas City, MO 64108, United States 2011 //<br>DOI: 10.1016/j.jpain.2010.11.004	Does not utilize/ assess tool
Effectiveness of interdisciplinary interventions in paediatric chronic pain management: a systematic review and subset meta-analysis<br>Liossi, C; Johnstone, L; Lilley, S; Caes, L; Williams, G; Schoth, D E<br>British Journal of Anaesthesia // 2019;123(2):e359-e371<br>University of Southampton, School of Psychology, Southampton, United Kingdom Elsevier Ltd 2019 //<br>DOI: 10.1016/j.bja.2019.01.024	Does not utilize/ assess tool
A case report and literature review of autism and attention deficit hyperactivity disorder in paediatric chronic pain<br>Wiwe Lipsker, C; von Heijne, M; Bölte, S; Wicksell, R K<br>Acta Paediatrica, International Journal of Paediatrics // 2018;107(5):753-758<br>Functional Area Medical Psychology, Functional Unit Behavioural Medicine, Karolinska University Hospital, Stockholm, Sweden Blackwell Publishing Ltd 2018 //<br>DOI: 10.1111/apa.14220	Does not utilize or assess tool
#52 - Ruiz 2010<br>A review of acceptance and commitment therapy (ACT) empirical evidence: Correlational, experimental psychopathology, component and outcome studies	Not English or French

Ruiz, F J<br>International Journal of Psychology and Psychological Therapy // 2010;10(1):125-162<br>Universidad de Almería, Spain 2010 //	
Acceptance and commitment therapy for parental management of childhood asthma: An RCT<br>Chong, Y.-Y.; Mak, Y.-W.; Leung, S.-P.; Lam, S.-Y.; Loke, A Y<br>Pediatrics // 2019;143(2):<br>School of Nursing, Hong Kong Polytechnic University, 11 Yuk Choi Road, Hung Hom, Kowloon, Hong Kong Special Administrative Region, Hong Kong American Academy of Pediatrics 2019 //<br>DOI: 10.1542/peds.2018-1723	Does not utilize/ assess tool
Adolescent chronic pain-related functioning: Concordance and discordance of mother-proxy and self-report ratings<br>Cohen, L L; Vowles, K E; Eccleston, C<br>European Journal of Pain // 2010;14(8):882-886<br>Department of Psychology, Georgia State University, United States 2010 //<br>DOI: 10.1016/j.ejpain.2010.01.005	Does not utilize/ assess tool
Effectiveness and cost-effectiveness of a guided and unguided internet-based acceptance and commitment therapy for chronic pain: Study protocol for a three-armed randomised controlled trial<br>Lin, J; Lüking, M; Ebert, D D; Buhrman, M; Andersson, G; Baumeister, H<br>Internet Interventions // 2015;2(1):7-16<br>Department of Rehabilitation Psychology and Psychotherapy, Institute of Psychology, University of Freiburg, Germany Elsevier 2015 //<br>DOI: 10.1016/j.invent.2014.11.005	Does not utilize/ assess tool
Predicting and preventing chronic postsurgical pain and disability<br>Wicksell, R K; Olsson, G L<br>Anesthesiology // 2010;113(6):1260-1261<br>Behavioral Medicine Pain Treatment Service, Karolinska University Hospital, Stockholm, Sweden Lippincott Williams and Wilkins 2010 //<br>DOI: 10.1097/ALN.0b013e3181da89f8	Does not utilize/ assess tool
Randomized controlled trial of an Internet-delivered family cognitive-behavioral therapy intervention for children and adolescents with chronic pain<br>Palermo, T M; Wilson, A C; Peters, M; Lewandowski, A; Somhegyi, H<br>Pain // 2009;146(1-2):205-213<br>Department of Anesthesiology and Perioperative Medicine, Oregon Health and Science University, Portland, OR, United States 2009 //	Does not utilize/ assess tool

DOI: 10.1016/j.pain.2009.07.034	
Collaborating with pediatricians and gastroenterologists: A biopsychosocial approach to treatment of gastrointestinal disorders<br>Clendaniel, L D; Hyman, P E; Courtney, J C<br>Pediatricians and Pharmacologically Trained Psychologists: Practitioner's Guide to Collaborative Treatment // 2011;():199-229<br>Department of Psychology, Children's Hospital of New Orleans, New Orleans, LA, United States Springer New York 2011 //<br>DOI: 10.1007/978-1-4419-7780-9_12	Does not utilize/ assess tool
Psychological variables potentially implicated in opioid-related mortality as observed in clinical practice<br>Passik, S D; Lowery, A<br>Pain Medicine // 2011;12(SUPPL. 2):S36-S42<br>Department of Psychiatry and Anesthesiology, Vanderbilt University Medical Center, Psychosomatic Medicine, Nashville, TN, United States Blackwell Publishing Inc. 2011 //<br>DOI: 10.1111/j.1526-4637.2011.01130.x	Does not utilize/ assess tool
Acceptation and Commitment Therapy (ACT) and Mindfulness, a model of psychological flexibility for chronic pain<br>Masselin-Dubois, A<br>Douleurs // 2016;17(5):233-251<br>2016 //<br>DOI: 10.1016/j.douler.2016.08.003	Cannot locate full text
Pain therapy with children and adolescents severely disabled due to chronic pain: Long-term outcome after inpatient pain therapy<br>Dobe, M; Hechler, T; Behlert, J; Kosfelder, J; Zernikow, B<br>Schmerz // 2011;25(4):411-422<br>Vodafone Stiftungsinstitut und Lehrstuhl fur Kinderschmerztherapie und Padiatrische Palliativmedizin, Vestische Kinder- und Jugendklinik Datteln, Universität Witten/Herdecke, Dr.-Friedrich-Steiner-Str. 5, Datteln 45711, Germany 2011 //<br>DOI: 10.1007/s00482-011-1051-2	Not English or French
Psychological therapies for the management of chronic and recurrent pain in children and adolescents<br>Eccleston, C; Palermo, T M; Williams, A C C; Lewandowski Holley, A; Morley, S; Fisher, E; Law, E<br>Cochrane Database of Systematic Reviews // 2014;2017(10):<br>University of Bath, Centre for Pain Research, Claverton Down, Bath, United Kingdom John Wiley and Sons Ltd 2014 //<br>DOI: 10.1002/14651858.CD003968.pub4	Does not utilize/ assess tool

A systematic review of the use of acceptance and commitment therapy in supporting parents<br>Byrne, G; Ghráda, Á N Í; O'Mahony, T; Brennan, E<br>Psychology and Psychotherapy: Theory, Research and Practice // 2020;():<br>Health Service Executive, Dublin, Ireland Wiley-Blackwell 2020 //<br>DOI: 10.1111/papt.12282	Does not utilize/ assess tool
Measurement framework for the Environmental influences on Child Health Outcomes research program<br>Blackwell, C K; Wakschlag, L S; Gershon, R C; Cella, D<br>Current Opinion in Pediatrics // 2018;30(2):276-284<br>Department of Medical Social Sciences, Feinberg School of Medicine, Northwestern University, 633 N. Saint Clair Street, 19th Floor, Chicago, IL 60611, United States Lippincott Williams and Wilkins 2018 //<br>DOI: 10.1097/MOP.0000000000000606	Does not utilize/ assess tool
Psychologycal and behavioral changes in chronic pain<br>Gavrilov, V; Pavlov, G<br>Anaesthesiology and Intensive Care // 2018;47(2):14-21<br>City Clinic Oncology Centre - Sofia, Dept of Anesthesiology and Intensive Care, Bulgaria Medical Information Center 2018 //	Cannot locate full text
Acceptance and Commitment Therapy for the Treatment of Adolescent Depression: A Pilot Study in a Psychiatric Outpatient Setting<br>Hayes, L; Boyd, C P; Sewell, J<br>Mindfulness // 2011;2(2):86-94<br>Psychology, University of Ballarat and Ballarat Health Services CAMHS, P.O. Box 663, Mount Helen, VIC, 3353, Australia 2011 //<br>DOI: 10.1007/s12671-011-0046-5	Does not utilize/ assess tool
Processes of change in Acceptance and Commitment Therapy and Applied Relaxation for long-standing pain<br>Kemani, M K; Hesser, H; Olsson, G L; Lekander, M; Wicksell, R K<br>European Journal of Pain (United Kingdom) // 2016;20(4):521-531<br>Behavioural Medicine Pain Treatment Services, Karolinska University Hospital, Stockholm, Sweden Blackwell Publishing Ltd 2016 //<br>DOI: 10.1002/ejp.754	Not pediatric population
Enhancing daily functioning with exposure and acceptance strategies: An important stride in the development of psychological therapies for pediatric chronic pain<br>Palermo, T M<br>Pain // 2009;141(3):189-190	Does not utilize/ assess tool

Division of Clinical Pain and Regional Anesthesia Research, Dept. of Anesthesiology and Peri-Operative Medicine, Oregon Health and Science University, 3181 SW Sam Jackson Park Rd., UHN-2, Portland, OR 97239, United States 2009 //<br>DOI: 10.1016/j.pain.2008.12.012	
Psychological therapies for the management of chronic and recurrent pain in children and adolescents<br>Fisher, E; Law, E; Dudeney, J; Palermo, T M; Stewart, G; Eccleston, C<br>Cochrane Database of Systematic Reviews // 2018;2018(9):<br>Pain Research Unit, Churchill Hospital, Cochrane Pain, Palliative and Supportive Care Group, Oxford, United Kingdom John Wiley and Sons Ltd 2018 //<br>DOI: 10.1002/14651858.CD003968.pub5	Does not utilize/ assess tool
Exploring cultural competence in acceptance and commitment therapy outcomes<br>Woidneck, M R; Pratt, K M; Gundy, J M; Nelson, C R; Twohig, M P<br>Professional Psychology: Research and Practice // 2012;43(3):227-233<br>Department of Psychology, Utah State University, United States 2012 //<br>DOI: 10.1037/a0026235	Does not utilize/ assess tool
Usage activity, perceived usefulness, and satisfaction in a web-based acceptance and commitment therapy program among Finnish ninth-grade adolescents<br>Hämäläinen, Tetta; Kaipainen, Kirsikka; Lappalainen, Päivi; Puolakanaho, Anne; Keinonen, Katariina; Lappalainen, Raimo; Kiuru, Noona<br>Internet Interventions 2021;25():<br>Elsevier B.V. 2021<br>DOI: 10.1016/j.invent.2021.100421	Not chronic pain population
Feasibility of group-based acceptance and commitment therapy for adolescents (AHEAD) with multiple functional somatic syndromes: A pilot study<br>Kallesøe, Karen Hansen; Schröder, Andreas; Wicksell, Rikard K.; Preuss, Tua; Jensen, Jens Søndergaard; Rask, Charlotte Ulrikka<br>BMC Psychiatry 2020;20(1):<br>BioMed Central Ltd 2020<br>DOI: 10.1186/s12888-020-02862-z	Does not utilize/ assess tool
The effectiveness of acceptance and commitment therapy (ACT) on the anxiety, depression and psychological well-being of patients with hypothyroidism<br>Fakharian, N; Samari Safa, J; Ghezelsefloo, M<br>Iranian Journal of Endocrinology and Metabolism // 2019;21(2):65-72	Not English or French

Department of Guidance & Counseling, Faculty of Humanities, Islamic Azad University Khomeinishahr Branch, Isfahan, Iran Endocrine Research Center 2019 //	
Pain Beliefs and Quality of Life in Young People with Disabilities and Bothersome Pain<br>Miró, J; Solé, E; Gertz, K; Jensen, M P; Engel, J M<br>Clinical Journal of Pain // 2017;33(11):998-1005<br>Pediatric Pain URV-Fundación Grünenthal, Unit for the Study and Treatment of Pain, ALGOS, Catalonia, Spain Lippincott Williams and Wilkins 2017 //<br>DOI: 10.1097/AJP.0000000000000482	Does not utilize/ assess tool
Psychological flexibility as a mediator of improvement in Acceptance and Commitment Therapy for patients with chronic pain following whiplash<br>Wicksell, R K; Olsson, G L; Hayes, S C<br>European Journal of Pain // 2010;14(10):1059.e1-1059.e11<br>Behavior Medicine Pain Treatment Service, Karolinska University Hospital, 171 76 Stockholm, Sweden Blackwell Publishing Ltd 2010 //<br>DOI: 10.1016/j.ejpain.2010.05.001	Does not utilize/ assess tool
A validation of the pain interference index in adults with long-standing pain<br>Kemani, M K; Zetterqvist, V; Kanstrup, M; Holmström, L; Wicksell, R K<br>Acta Anaesthesiologica Scandinavica // 2016;60(2):250-258<br>Behavioural Medicine Pain Treatment Services, Pain Center, Karolinska University Hospital, Stockholm, 171 76, Sweden Blackwell Munksgaard 2016 //<br>DOI: 10.1111/aas.12599	Not pediatric population
Pediatric fear-avoidance model of chronic pain: Foundation, application and future directions<br>Asmundson, G J G; Noel, M; Petter, M; Parkerson, H A<br>Pain Research and Management // 2012;17(6):397-405<br>Department of Psychology, University of Regina, 3737 Wascana Parkway, Regina, SK S4S 0A2, Canada Hindawi Limited 2012 //<br>DOI: 10.1155/2012/908061	Does not utilize/ assess tool
Psykosomatisk smärtdiagnos bör byggas på fastställda kriterier: Kan ge möjligheter till bättre vård<br>Alfvén, G<br>Lakartidningen // 2012;109(5):224-227<br>Hallunda Barn-och ungdomsmedicinsk mottagning (BUMM), Astrid Lindgrens barnsjukhus, Norsborg, Sweden 2012 //	Not English or French
Sex differences in the efficacy of psychological therapies for the management of chronic and recurrent pain in children and adolescents: A systematic review and meta-Analysis<br>Boerner, K E; Eccleston, C; Chambers, C T; Keogh, E	Does not utilize or assess tool

Pain // 2017;158(4):569-582<br>Department of Psychology and Neuroscience, Dalhousie University, 5850/5980 University Avenue, Halifax, NS B3K 6R8, Canada Lippincott Williams and Wilkins 2017 //<br>DOI: 10.1097/j.pain.0000000000000803	
Using Acceptance and Commitment Therapy to Help Young People Develop and Grow to Their Full Potential<br>Hayes, L; Ciarrochi, J<br>Promoting Psychological Well-Being in Children and Families // 2015;():102-122<br>Orygen Youth Health Research Centre, University of Melbourne, Australia Palgrave Macmillan 2015 //<br>DOI: 10.1057/9781137479969_7	Cannot locate full text
The effectiveness of acceptance and commitment therapy for children with chronic pain on the quality of life on 7 to 12 year-old children<br>Ghomian, S; Shairi, M R<br>International Journal of Pediatrics // 2014;2(3):47-55<br>Faculty of Humanities, Shahed University, Tehran, Iran Mashhad University of Medical Sciences 2014 //<br>DOI: 10.22038/ijp.2014.2995	Does not utilize/ assess tool
Family and parent influences on pediatric chronic pain<br>Palermo, T M; Valrie, C R; Karlson, C W<br>American Psychologist // 2014;69(2):142-152<br>Department of Anesthesiology and Pain Medicine, University of Washington, Center for Child Health, Behavior, and Development, Seattle Children's Research Institute, Seattle, WA, United States 2014 //<br>DOI: 10.1037/a0035216	Does not utilize/ assess tool
Customized CBT via internet for adolescents with pain and emotional distress: A pilot study<br>Flink, I K; Sfyrkou, C; Persson, B<br>Internet Interventions // 2016;4():43-50<br>Center for Health and Medical Psychology (CHAMP), Institution of Law, Psychology and Social Work, Örebro University, Örebro, Sweden Elsevier B.V. 2016 //<br>DOI: 10.1016/j.invent.2016.03.002	Does not utilize/ assess tool
Latent Class Analysis of the Short and Long Forms of the Chronic Pain Acceptance Questionnaire: Further Examination of Patient Subgroups<br>Rovner, G; Vowles, K E; Gerdle, B; Gillanders, D<br>Journal of Pain // 2015;16(11):1095-1105	Does not utilize/ assess tool

Division of Rehabilitation Medicine, Section for Highly Specialized Pain Rehabilitation, Department of Clinical Sciences, Danderyd Hospital, Karolinska Institutet, Stockholm, Sweden Churchill Livingstone Inc. 2015 //<br>DOI: 10.1016/j.jpain.2015.07.007	
Acceptance-based therapy: the potential to augment behavioral interventions in the treatment of type 2 diabetes<br>Cardel, M I; Ross, K M; Butryn, M; Donahoo, W T; Eastman, A; Dillard, J R; Grummon, A; Hopkins, P; Whigham, L D; Janicke, D<br>Nutrition and Diabetes // 2020;10(1):<br>Department of Health Outcomes and Biomedical Informatics, University of Florida, Gainesville, FL 32611, United States Springer Nature 2020 //<br>DOI: 10.1038/s41387-020-0106-9	Does not utilize/ assess tool
The efficacy of Acceptance and Commitment Therapy: An updated systematic review and meta-analysis<br>Öst, L.-G.<br>Behaviour Research and Therapy // 2014;61():105-121<br>Department of Clinical Neuroscience, Division of Psychology, Karolinska Institutet, Stockholm, SE-171 77, Sweden Elsevier Ltd 2014 //<br>DOI: 10.1016/j.brat.2014.07.018	Does not utilize/ assess tool
Living Life With My Child's Pain: The Parent Pain Acceptance Questionnaire (PPAQ)<br>Smith, A M; Sieberg, C B; Odell, S; Randall, E; Simons, L E<br>Clinical Journal of Pain // 2015;31(7):633-641<br>Department of Anesthesiology, Perioperative and Pain Medicine, Division of Pain Medicine, Boston Children's Hospital, 333 Longwood Avenue, 5th floor, Boston, MA 02115, United States Lippincott Williams and Wilkins 2015 //<br>DOI: 10.1097/AJP.0000000000000140	Does not utilize/ assess tool
Does Pain Intensity Matter? The Relation between Coping and Quality of Life in Pediatric Patients with Chronic Pain<br>Yetwin, A K; Mahrer, N E; John, C; Gold, J I<br>Journal of Pediatric Nursing // 2018;40():7-13<br>Department of Anesthesiology Critical Care Medicine, Children's Hospital Los Angeles, United States W.B. Saunders 2018 //<br>DOI: 10.1016/j.pedn.2018.02.003	Does not utilize/ assess tool
Gender differences in the relation between functioning and values-based living in youth with sickle cell disease.<br>Martin, Sarah R; Cohen, Lindsey L; Welkom, Josie S; Feinstein, Amanda; Masuda, Aki; Griffin, Anya<br>Clinical Practice in Pediatric Psychology // 2016;4(1):11-22	Does not utilize/ assess tool

Cohen, Lindsey L.: Department of Psychology, Georgia State University, Atlanta, GA, US, 30302-5010, llcohen@gsu.edu<br>Educational Publishing Foundation 2016 //<br>DOI: 10.1037/cpp0000127	
The management of young people who self-harm by New Zealand Infant, Child and Adolescent Mental Health Services: cutting-edge or cutting corners?<br>Thabrew, H; Gandeza, E; Bahr, G; Bettany, D; Bampton, C; Cooney, E; Coleman, N; Tiatia-Seath, J<br>Australasian Psychiatry // 2018;26(2):152-159<br>University of Auckland, Auckland, New Zealand SAGE Publications Inc. 2018 //<br>DOI: 10.1177/1039856217748248	Does not utilize/ assess tool
Disentangling the complex relations among caregiver and adolescent responses to adolescent chronic pain<br>Vowles, K E; Cohen, L L; McCracken, L M; Eccleston, C<br>Pain // 2010;151(3):680-686<br>Interdisciplinary Musculoskeletal Pain Assessment and Community Treatment Service, Haywood Hospital, Keele University, Stoke-on-Trent, United Kingdom 2010 //<br>DOI: 10.1016/j.pain.2010.08.031	Does not utilize/ assess tool
Case formulation in persistent pain in children and adolescents: The application of the nonlinear dynamic systems perspective<br>Sinclair, C; Meredith, P; Strong, J<br>British Journal of Occupational Therapy // 2018;81(12):727-732<br>Children's Pain Management Clinic, Royal Children's Hospital, Melbourne, Australia SAGE Publications Inc. 2018 //<br>DOI: 10.1177/0308022618802722	Does not utilize/ assess tool
Somatoform and related disorders<br>Elena Garralda, M; Rask, C U<br>Rutter's Child and Adolescent Psychiatry: Sixth Edition // 2015;():1035-1054<br>Academic Unit of Child and Adolescent Psychiatry, Imperial College London, London, United Kingdom John Wiley and Sons Ltd 2015 //<br>DOI: 10.1002/9781118381953.ch72	Does not utilize/ assess tool
Acceptance and commitment therapy (ACT) to foster resilience in pediatric chronic illness<br>Ernst, M M; Mellon, M W<br>Child and Adolescent Resilience Within Medical Contexts: Integrating Research and Practice // 2016;():193-207<br>Department of Pediatrics, Division of Behavioral Medicine and Clinical Psychology, Cincinnati Children's Hospital Medical Center, University of Cincinnati College of Medicine, Cincinnati, OH, United States Springer International Publishing 2016 //	Does not utilize/ assess tool

DOI: 10.1007/978-3-319-32223-0_11	
Catastrophizing, pain, and functional outcomes for children with chronic pain: A meta-analytic review<br>Miller, M M; Meints, S M; Hirsh, A T<br>Pain // 2018;159(12):2442-2460<br>Department of Anesthesiology, Pain Management Center, Brigham and Women's Hospital, Harvard Medical School, Chestnut Hill, MA, United States Lippincott Williams and Wilkins 2018 //<br>DOI: 10.1097/j.pain.0000000000001342	Does not utilize/ assess tool
A group-based, acceptance & commitment therapy intervention for chronic pain<br>Cosio, D<br>Social Work with Groups // 2019;():<br>Department of Anesthesiology/Pain Clinic, Jesse Brown VA Medical Center, Chicago, IL, United States Routledge 2019 //<br>DOI: 10.1080/01609513.2019.1604290	Does not utilize/ assess tool
Mindfulness Based Interventions for Youth<br>Zack, S; Saekow, J; Kelly, M; Radke, A<br>Journal of Rational - Emotive and Cognitive - Behavior Therapy // 2014;32(1):44-56<br>Department of Psychiatry and Behavioral Sciences, Stanford School of Medicine, 401 Quarry Rd., Stanford, CA, 94305, United States Springer New York LLC 2014 //<br>DOI: 10.1007/s10942-014-0179-2	Does not utilize/ assess tool
Acceptance and Commitment Therapy as a Unified Model of Behavior Change<br>Hayes, S C; Pistorello, J; Levin, M E<br>The Counseling Psychologist // 2012;40(7):976-1002<br>University of Nevada, Reno, United States 2012 //<br>DOI: 10.1177/0011000012460836	Does not utilize/ assess tool
A meta-analysis of dropout rates in acceptance and commitment therapy<br>Ong, C W; Lee, E B; Twohig, M P<br>Behaviour Research and Therapy // 2018;104():14-33<br>Department of Psychology, Utah State University, 2810 Old Main Hill, Logan, UT 84322, United States Elsevier Ltd 2018 //<br>DOI: 10.1016/j.brat.2018.02.004	Does not utilize/ assess tool
Fear of pain in the context of intensive pain rehabilitation among children and adolescents with neuropathic pain: Associations with treatment response<br>Simons, L E; Kaczynski, K J; Conroy, C; Logan, D E<br>Journal of Pain // 2012;13(12):1151-1161	Does not utilize/ assess tool

Department of Anesthesiology, Perioperative and Pain Medicine, Children's Hospital Boston, Harvard Medical School, Boston, MA, United States 2012 //<br>DOI: 10.1016/j.jpain.2012.08.007	
Acceptance and commitment therapy for fibromyalgia: A randomized controlled trial<br>Wicksell, R K; Kemani, M; Jensen, K; Kosek, E; Kadetoff, D; Sorjonen, K; Ingvar, M; Olsson, G L<br>European Journal of Pain (United Kingdom) // 2013;17(4):599-611<br>Karolinska University Hospital, Behavior Medicine Pain Treatment Service, Stockholm, Sweden 2013 //<br>DOI: 10.1002/j.1532-2149.2012.00224.x	Does not utilize/ assess tool
Acceptance and commitment therapy for psychological and physiological illnesses: A systematic review for social workers<br>Montgomery, K L; Kim, J S; Franklin, C<br>Health and Social Work // 2011;36(3):169-181<br>School of Social Work, University of Texas at Austin, 1 University Station D3 500, Austin, TX 78712, United States National Association of Social Workers 2011 //<br>DOI: 10.1093/hsw/36.3.169	Does not utilize/ assess tool
Measuring parent beliefs about child acceptance of pain: A preliminary validation of the Chronic Pain Acceptance Questionnaire, parent report<br>Simons, Laura E; Sieberg, Christine B; Kaczynski, Karen J<br>PAIN 10// 2011;152(10):2294-2300<br>TWO COMMERCE SQ, 2001 MARKET ST, PHILADELPHIA, PA 19103 USA LIPPINCOTT WILLIAMS & WILKINS 2011 10//<br>DOI: 10.1016/j.pain.2011.06.018	Does not utilize/ assess tool
Acceptance, well-being and goals in adolescents with chronic illness: A daily process analysis<br>Casier, A; Goubert, L; Gebhardt, W A; Baets, F D; Aken, S V; Matthys, D; Crombez, G<br>Psychology and Health // 2013;28(11):1337-1351<br>Department of Experimental-Clinical and Health Psychology, Ghent University, Ghent, Belgium 2013 //<br>DOI: 10.1080/08870446.2013.809083	Does not utilize/ assess tool
Complex Regional Pain Syndromes I and II (Reflex Sympathetic Dystrophy, Causalgia)<br>Sethna, N F; Logan, D<br>Neuromuscular Disorders of Infancy, Childhood, and Adolescence: A Clinician's Approach // 2015;():976-983<br>Psychological Services for Pain Medicine, Mayo Family Pediatric Pain Rehabilitation Center, Boston Children's Hospital, Boston, MA, United States Elsevier Inc. 2015 //<br>DOI: 10.1016/B978-0-12-417044-5.00048-2	Cannot locate full text

Cognitive-behavioural therapy in children and adolescents with chronic pain<br>Stropnik, Staša; Krkoč, Vesna<br>Psiholoska Obzorja 2020;29():32-41<br>Slovenian Psychologists' Association 2020<br>DOI: 10.20419/2020.29.508	Cannot locate full text
One-day behavioral intervention in depressed migraine patients: Effects on headache<br>Dindo, L; Recober, A; Marchman, J; O'Hara, M W; Turvey, C<br>Headache // 2014;54(3):528-538<br>Department of Psychiatry, University of Iowa College of Medicine, Psychiat. Res. MEB 2-203, Iowa City, IA 52242-1000, United States Blackwell Publishing Inc. 2014 //<br>DOI: 10.1111/head.12258	Does not utilize/ assess tool
Pain and emotion: A biopsychosocial review of recent research<br>Lumley, M A; Cohen, J L; Borszcz, G S; Cano, A; Radcliffe, A M; Porter, L S; Schubiner, H; Keefe, F J<br>Journal of Clinical Psychology // 2011;67(9):942-968<br>Wayne State University, United States 2011 //<br>DOI: 10.1002/jclp.20816	Does not utilize/ assess tool
A systematic review of randomised controlled trials using psychological interventions for children and adolescents with medically unexplained symptoms: A focus on mental health outcomes<br>O'Connell, C; Shafran, R; Bennett, S<br>Clinical Child Psychology and Psychiatry // 2020;25(1):273-290<br>Department of Applied Psychology, Canterbury Christ Church University, Kent, United Kingdom SAGE Publications Ltd 2020 //<br>DOI: 10.1177/1359104519855415	Does not utilize/ assess tool
Role of psychological flexibility in parents of adolescents with chronic pain: Development of a measure and preliminary correlation analyses<br>McCracken, L M; Gauntlett-Gilbert, J<br>Pain // 2011;152(4):780-785<br>Centre for Pain Services, Royal National Hospital for Rheumatic Diseases, University of Bath, Bath, United Kingdom 2011 //<br>DOI: 10.1016/j.pain.2010.12.001	Does not utilize/ assess tool
Fear-avoidance beliefs and parental responses to pain in adolescents with chronic pain<br>Wilson, A C; Lewandowski, A S; Palermo, T M<br>Pain Research and Management // 2011;16(3):178-182<br>Child Development and Rehabilitation Center, Oregon Health and Science University, CDRC, 3181 Southwest Sam Jackson Park Road, Portland, OR 97239, United States Hindawi Limited 2011 //	Does not utilize/ assess tool

DOI: 10.1155/2011/296298	
Efficacy and cost-effectiveness of acceptance and commitment therapy and applied relaxation for longstanding pain: A Randomized Controlled Trial<br>Kemani, M K; Olsson, G L; Lekander, M; Hesser, H; Andersson, E; Wicksell, R K<br>Clinical Journal of Pain // 2015;31(11):1004-1016<br>Behavioral Medicine Pain Treatment Services, Karolinska University Hospital, Stockholm, 171 76, Sweden Lippincott Williams and Wilkins 2015 //<br>DOI: 10.1097/AJP.0000000000000203	Does not utilize/ assess tool
Developing a stoma acceptance questionnaire to improve motivation to adhere to enterostoma self-care<br>Bagnasco, A; Watson, R; Zanini, M; Catania, G; Aleo, G; Sasso, L<br>Journal of Preventive Medicine and Hygiene // 2017;58(2):E190-E194<br>Department of Health Sciences, University of Genoa, Via Pastore 1, Genoa, 16132, Italy Pacini Editore S.p.A. 2017 //	Does not utilize/ assess tool
Psychological Flexibility as a Resilience Factor in Individuals With Chronic Pain<br>Gentili, C; Rickardsson, J; Zetterqvist, V; Simons, L E; Lekander, M; Wicksell, R K<br>Frontiers in Psychology // 2019;10():<br>Functional Area Medical Psychology, Functional Unit Behavior Medicine, Karolinska University Hospital, Stockholm, Sweden Frontiers Media S.A. 2019 //<br>DOI: 10.3389/fpsyg.2019.02016	Not pediatric population
Pilot Randomized Trial of Integrated Cognitive-Behavioral Therapy and Neuromuscular Training for Juvenile Fibromyalgia: The FIT Teens Program<br>Kashikar-Zuck, S; Black, W R; Pfeiffer, M; Peugh, J; Williams, S E; Ting, T V; Thomas, S; Kitchen, K; Myer, G D<br>Journal of Pain // 2018;19(9):1049-1062<br>Department of Pediatrics, University of Cincinnati College of Medicine and Cincinnati Children's Hospital Medical Center, Cincinnati, Ohio, United States Churchill Livingstone Inc. 2018 //<br>DOI: 10.1016/j.jpain.2018.04.003	Does not utilize/ assess tool
Pain assessment methods and interventions used by pediatric psychologists: A survey by the pain special interest group of the society of pediatric psychology<br>Junghans-Rutelonis, A N; Weiss, K E; Tamula, M A; Karvounides, D; Harbeck-Weber, C; Martin, S<br>Professional Psychology: Research and Practice // 2017;48(6):445-452<br>Department of Pain, Palliative Care and Integrative Medicine at Children's Hospitals and Clinics of Minnesota, United States American Psychological Association Inc. 2017 //<br>DOI: 10.1037/pro0000156	Does not utilize/ assess tool
Perceived oral health and care of children with juvenile idiopathic arthritis: A qualitative study	Does not utilize/ assess tool

Leksell, E; Hallberg, U; Magnusson, B; Ernberg, M; Hedenberg-Magnusson, B<br>Journal of Oral and Facial Pain and Headache // 2015;29(3):223-230<br>Department of Odontology Umeå University Umeå, Sweden Quintessence Publishing Co. Inc. 2015 //<br>DOI: 10.11607/ofph.1293	
Acceptance and Commitment Therapy for children: A systematic review of intervention studies<br>Swain, J; Hancock, K; Dixon, A; Bowman, J<br>Journal of Contextual Behavioral Science // 2015;4(2):73-85<br>Department of Psychological Medicine, The Children's Hospital at Westmead, Sydney, NSW 2145, Australia Elsevier Inc. 2015 //<br>DOI: 10.1016/j.jcbs.2015.02.001	Does not utilize/ assess tool
Contribution of kinesophobia and catastrophic thinking to upper-extremity-specific disability<br>Das De, S; Vranceanu, A.-M.; Ring, D C<br>Journal of Bone and Joint Surgery - Series A // 2013;95(1):76-81<br>Department of Orthopaedic Surgery, Yawkey Center 2100, Massachusetts General Hospital, 55 Fruit Street, Boston, MA 02114, United States Journal of Bone and Joint Surgery Inc. 2013 //<br>DOI: 10.2106/JBJS.L.00064	Does not utilize/ assess tool
Impact of acceptance-based nursing intervention on postsurgical recovery: Preliminary findings<br>Fernández, M D; Luciano, C; Valdivia-Salas, S<br>Spanish Journal of Psychology // 2012;15(3):1361-1370<br>Hospital Torrccárdenas, Spain Cambridge University Press 2012 //<br>DOI: 10.5209/rev-SJOP.2012.v15.n3.39421	Does not utilize/ assess tool
Title: Psychological mediators in the relationship between paediatric chronic pain and adjustment: an investigation of acceptance, catastrophising and kinesiophobia<br>;():	Does not utilize/ assess tool
Mindfulness predicts current risk of opioid analgesic misuse in chronic low back pain patients receiving opioid therapy<br>Villarreal, Yolanda R.; Stotts, Angela L.; Paniagua, Samantha Megan; Rosen, Kristen; Eckmann, Maxim; Suchting, Robert; Potter, Jennifer Sharpe<br>Journal of Contextual Behavioral Science 2020;18():111-116<br>Elsevier Inc. 2020<br>DOI: 10.1016/j.jcbs.2020.08.011	Does not utilize/ assess tool
Essentials of acceptance and commitment therapy<br>Batten, S V	Not an original study

Essentials of Acceptance and Commitment Therapy // 2011;():1-125<br>United States Department of Veterans Affairs (VA), United States SAGE Publications Inc. 2011 //<br>DOI: 10.4135/9781446251843	
Fear reduction in patients with chronic pain: A learning theory perspective<br>Hollander, M D; De Jong, J R; Volders, S; Goossens, M E; Smeets, R J; Vlaeyen, J W<br>Expert Review of Neurotherapeutics // 2010;10(11):1733-1745<br>University Medical Centre Maastricht, Maastricht, Netherlands 2010 //<br>DOI: 10.1586/ern.10.115	Cannot locate full text
Psychological and nonpsychological interventions for chronic pediatric pain<br>Hermann, C<br>Pain 2012 Refresher Courses: 14th World Congress on Pain // 2015;():<br>Department of Clinical Psychology and Psychotherapy, Justus-Liebig University, Otto-Behaghel-Str. 10F, Giessen, D-35394, Germany Wolters Kluwer Health Adis (ESP) 2015 //	Cannot locate full text
Review: Effectiveness of mindfulness in improving mental health symptoms of children and adolescents: A meta-analysis<br>Kallapiran, K; Koo, S; Kirubakaran, R; Hancock, K<br>Child and Adolescent Mental Health // 2015;20(4):182-194<br>The Children's Hospital at Westmead, The Sydney Children's Hospital Network, Locked Bag 4001, Westmead, NSW 2145, Australia Blackwell Publishing Ltd 2015 //<br>DOI: 10.1111/camh.12113	Does not utilize/ assess tool
Cognitive behavioral therapy for pediatric chronic pain: The problem, research, and practice<br>Noel, M; Petter, M; Parker, J A; Chambers, C T<br>Journal of Cognitive Psychotherapy // 2012;26(2):143-156<br>Department of Psychology, Dalhousie University, and Centre for Pediatric Pain Research, IWK Health Centre, Halifax, NS, Canada Springer Publishing Company 2012 //<br>DOI: 10.1891/0889-8391.26.2.143	Does not utilize/ assess tool
Implementing psychological therapies for gastrointestinal disorders in pediatrics<br>Reed, Bonney; Buzenski, Jessica; van Tilburg, Miranda A.L.<br>Expert Review of Gastroenterology and Hepatology 2020;14(11):1061-1067<br>Taylor and Francis Ltd. 2020<br>DOI: 10.1080/17474124.2020.1806055	Does not utilize/ assess tool
Improved pain acceptance and interference following outpatient interdisciplinary pediatric chronic pain treatment	Does not utilize/ assess tool

Salamon, Katherine S.; Dutta, Richa Aggarwal; Hildenbrand, Aimee K.<br>Psychology and Health 2022;():<br>Routledge 2022<br>DOI: 10.1080/08870446.2021.2024540	
Acute and chronic pain in children: Role of the parents?<br>Goubert, L; Vervoort, T<br>Tijdschrift voor Geneeskunde // 2014;70(21):1240-1248<br>Vakgroep Experimenteel-Klinische en Gezondheidspsychologie, Faculteit Psychologie en Pedagogische Wetenschappen, Universiteit Gent, Henri Dunantlaan 2, Gent, 9000, Belgium Tijdschrift voor Geneeskunde 2014 //<br>DOI: 10.2143/TVG.70.21.2001721	Cannot locate full text
The Effectiveness of Acceptance and Commitment Therapy for Adolescent Mental Health: Swedish and Australian Pilot Outcomes<br>Livheim, F; Hayes, L; Ghaderi, A; Magnusdottir, T; Högfeldt, A; Rowse, J; Turner, S; Hayes, S C; Tengström, A<br>Journal of Child and Family Studies // 2015;24(4):1016-1030<br>Department of Clinical Neuroscience, Karolinska Institutet, Stockholm, Sweden Springer New York LLC 2015 //<br>DOI: 10.1007/s10826-014-9912-9	Does not utilize/ assess tool
Mindfulness-based cognitive therapy for posttraumatic stress disorder<br>Sears, R M; Chard, K M<br>Mindfulness-Based Cognitive Therapy for Posttraumatic Stress Disorder // 2016;():1-187<br>American Board of Professional Psychology (ABPP), United States wiley 2016 //<br>DOI: 10.1002/9781118691403	Not an original study
Acceptance and commitment therapy<br>Ducasse, D; Fond, G<br>Encephale // 2015;41(1):1-9<br>Université Montpellier 1, Montpellier, 34000, France Elsevier Masson SAS 2015 //<br>DOI: 10.1016/j.encep.2013.04.017	Does not utilize/ assess tool
ACTsmart – development and feasibility of digital Acceptance and Commitment Therapy for adults with chronic pain<br>Gentili, C; Zetterqvist, V; Rickardsson, J; Holmström, L; Simons, L E; Wicksell, R K<br>npj Digital Medicine // 2020;3(1):<br>Functional Unit Behavioral Medicine, Function Area Medical Psychology, Karolinska University Hospital, Stockholm, Sweden Nature Research 2020 //<br>DOI: 10.1038/s41746-020-0228-4	Does not utilize/ assess tool

Acceptance, cognitive restructuring, and distraction as coping strategies for acute pain<br>Kohl, A; Rief, W; Glombiewski, J A<br>Journal of Pain // 2013;14(3):305-315<br>Department of Clinical Psychology and Psychotherapy, Philipps-University of Marburg, Gutenbergstrasse 18, Marburg, Germany 2013 //<br>DOI: 10.1016/j.jpain.2012.12.005	Does not utilize/ assess tool
The Effects of Acceptance of Thoughts, Mindful Awareness of Breathing, and Spontaneous Coping on an Experimentally Induced Pain Task<br>Forsyth, L; Hayes, L L<br>Psychological Record // 2014;64(3):447-455<br>School of Social Sciences and Psychology, University of Western Sydney, Sydney, NSW 2751, Australia Springer International Publishing 2014 //<br>DOI: 10.1007/s40732-014-0010-6	Does not utilize/ assess tool
Exercise and Acceptance and Commitment Therapy for Chronic Pain: A Case Series with One-Year Follow-Up<br>Casey, M.-B.; Cotter, N; Kelly, C; Mc Elchar, L; Dunne, C; Neary, R; Lowry, D; Hearty, C; Doody, C<br>Musculoskeletal Care // 2020;():<br>2020 //<br>DOI: 10.1002/msc.1444	Does not utilize/ assess tool
Toward understanding acceptance and psychological flexibility in chronic pain<br>McCracken, L M<br>Pain // 2010;149(3):420-421<br>Centre for Pain Services, Royal National Hospital for Rheumatic Diseases, Centre for Pain Research, Bath, BA1 1RL, United Kingdom 2010 //<br>DOI: 10.1016/j.pain.2010.02.036	Does not utilize/ assess tool
Pain in Children<br>Palermo, T M; Koh, J L; Zeltzer, L K<br>Clinical Pain Management: A Practical Guide // 2010;():319-325<br>Seattle Children's Hospital, University of Washington School of Medicine, Seattle, United States Wiley-Blackwell 2010 //<br>DOI: 10.1002/9781444329711.ch38	Does not utilize/ assess tool
Prevalence of autism traits and attention-deficit hyperactivity disorder symptoms in a clinical sample of children and adolescents with chronic pain<br>Lipsker, C W; Bölte, S; Hirvikoski, T; Lekander, M; Holmström, L; Wicksell, R K	Does not utilize or assess tool

Journal of Pain Research // 2018;11():2827-2836<br>Functional Area Medical Psychology, Functional Unit Behavior Medicine, Karolinska University Hospital, Stockholm, Sweden<br>Dove Medical Press Ltd. 2018 //<br>DOI: 10.2147/JPR.S177534	
Group CBT-yoga protocol targeting pain-related and internalizing symptoms in youth<br>Allen, T M; Wren, A A; Anderson, L M; Sabholk, A; Mauro, C F<br>Clinical Practice in Pediatric Psychology // 2018;6(1):7-18<br>Duke University, 2608 Erwin Road Suite 300, Durham, NC 27705, United States American Psychological Association Inc. 2018 //<br>DOI: 10.1037/cpp0000206	Does not utilize/ assess tool
Cross-Cultural Adaptation and Psychometric Properties of the European Portuguese Version of the Central Sensitization Inventory in Adolescents With Musculoskeletal Chronic Pain<br>Andias, R; Silva, A G<br>Pain Practice // 2020;20(5):480-490<br>School of Health Sciences, University of Aveiro, Aveiro, Portugal Blackwell Publishing Inc. 2020 //<br>DOI: 10.1111/papr.12875	Does not utilize/ assess tool
Acceptance and Commitment Therapy versus Tinnitus Retraining Therapy in the treatment of tinnitus: A randomised controlled trial<br>Westin, V Z; Schulin, M; Hesser, H; Karlsson, M; Noe, R Z; Olofsson, U; Stalby, M; Wisung, G; Andersson, G<br>Behaviour Research and Therapy // 2011;49(11):737-747<br>Department of Behavioural Sciences and Learning, Linköping University, 581 83 Linköping, Sweden 2011 //<br>DOI: 10.1016/j.brat.2011.08.001	Does not utilize/ assess tool
Family-based interventions for children and adolescents with functional somatic symptoms: a systematic review<br>Hulgaard, D; Dehlholm-Lambertsen, G; Rask, C U<br>Journal of Family Therapy // 2019;41(1):4-28<br>Department of Child and Adolescent Psychiatry, Mental Health Services in the Region of Southern Denmark, Odense, Denmark Blackwell Publishing Ltd 2019 //<br>DOI: 10.1111/1467-6427.12199	Does not utilize/ assess tool
Acceptance & Commitment Therapy for ME/CFS (Chronic Fatigue Syndrome) – A feasibility study<br>Jonsjö, M A; Wicksell, R K; Holmström, L; Andreasson, A; Olsson, G L<br>Journal of Contextual Behavioral Science // 2019;12():89-97<br>Behavior Medicine, Karolinska University Hospital, Stockholm, Sweden Elsevier Inc. 2019 //	Does not utilize/ assess tool

DOI: 10.1016/j.jcbs.2019.02.008	
Efficacy of adding interoceptive exposure to intensive interdisciplinary treatment for adolescents with chronic pain: A randomized controlled trial<br>Flack, F; Stahlschmidt, L; Dobe, M; Hirschfeld, G; Strasser, A; Michalak, J; Wager, J; Zernikow, B<br>Pain // 2018;159(11):2223-2233<br>German Paediatric Pain Centre, Children's and Adolescents' Hospital, Department of Children's Pain Therapy and Paediatric Palliative Care, Faculty of Health, School of Medicine, Witten/Herdecke University, Dr.-Friedrich Steiner Str 5, Datteln, 45711, Germ Lippincott Williams and Wilkins 2018 //<br>DOI: 10.1097/j.pain.0000000000001321	Does not utilize/ assess tool
Quality of life and academic functioning 6 years after paediatric referral for chronic pain<br>Knook, L M E; Lijmer, J G; Konijnenberg, A Y; Hordijk, P M; Van Engeland, H<br>Acta Paediatrica, International Journal of Paediatrics // 2012;101(9):957-963<br>Altrecht Eating Disorders Rintveld, Altrecht Mental Health Institute, Oude Arnhemseweg 260, 3705 BK, Zeist, Netherlands 2012 //<br>DOI: 10.1111/j.1651-2227.2012.02766.x	Does not utilize/ assess tool
The empirically supported status of acceptance and commitment therapy: An update<br>Smout, M F; Hayes, L; Atkins, P W B; Klausen, J; Duguid, J E<br>Clinical Psychologist // 2012;16(3):97-109<br>Centre for Treatment of Anxiety and Depression, 30 Anderson St, Thebarton, SA 5031, Australia 2012 //<br>DOI: 10.1111/j.1742-9552.2012.00051.x	Does not utilize/ assess tool
Multidisciplinary Treatment for Adolescents with Chronic Pain and/or Fatigue: Who Will Benefit?<br>Westendorp, T; Verbunt, J A; de Groot, I J M; Remerie, S C; ter Steeg, A; Smeets, R.J.E.M.<br>Pain Practice // 2017;17(5):633-642<br>Rijndam Rehabilitation, Rotterdam, Netherlands Blackwell Publishing Inc. 2017 //<br>DOI: 10.1111/papr.12495	Does not utilize/ assess tool
Pediatric complex regional pain syndrome: A review<br>Weissmann, R; Uziel, Y<br>Pediatric Rheumatology // 2016;14(1):<br>Meir Medical Center, Pediatric Rheumatology Unit, Department of Pediatrics, 49 Tshernichovsky St., Kfar Saba, 44281, Israel BioMed Central Ltd. 2016 //<br>DOI: 10.1186/s12969-016-0090-8	Does not utilize/ assess tool

Acceptance and commitment therapy in youth with neurofibromatosis type 1 (NF1) and chronic pain and their parents: A pilot study of feasibility and preliminary efficacy<br>Martin, S; Wolters, P L; Toledo-Tamula, M A; Schmitt, S N; Baldwin, A; Starosta, A; Gillespie, A; Widemann, B<br>American Journal of Medical Genetics, Part A // 2016;170(6):1462-1470<br>Pediatric Oncology Branch, National Cancer Institute, Montgomery County, Bethesda, MD, United States Wiley-Liss Inc. 2016 //<br>DOI: 10.1002/ajmg.a.37623	Not exclusive chronic pain population
Pain and its Impact on the Functional Ability in Children Treated at the Children's Cancer Center of Lebanon<br>Madi, D; Clinton, M<br>Journal of Pediatric Nursing // 2018;39():e11-e20<br>Hariri School of Nursing, American University of Beirut, Hamra-Bliss Street, Beirut, Lebanon W.B. Saunders 2018 //<br>DOI: 10.1016/j.pedn.2017.12.004	Does not utilize/ assess tool
A meta-analysis of the efficacy of acceptance and commitment therapy for children<br>Fang, S; Ding, D<br>Journal of Contextual Behavioral Science // 2020;15():225-234<br>Department of Psychology, Anhui Normal University, Wuhu, China Elsevier Inc. 2020 //<br>DOI: 10.1016/j.jcbs.2020.01.007	Does not utilize/ assess tool
Comparing group-based acceptance and commitment therapy (ACT) with enhanced usual care for adolescents with functional somatic syndromes: A study protocol for a randomised trial<br>Kallesøe, K H; Schröder, A; Wicksell, R K; Fink, P; Ørnbøl, E; Rask, C U<br>BMJ Open // 2016;6(9):<br>Research Clinic for Functional Disorders and Psychosomatics, Aarhus University Hospital, Aarhus, Denmark BMJ Publishing Group 2016 //<br>DOI: 10.1136/bmjopen-2016-012743	Not an original study
Treatments addressing pain-related fear and anxiety in patients with chronic musculoskeletal pain: A preliminary review<br>Bailey, K M; Carleton, R N; Vlaeyen, J W S; Asmundson, G J G<br>Cognitive Behaviour Therapy // 2010;39(1):46-63<br>Department of Psychology and the Anxiety and Illness Behaviours Laboratory, University of Regina, Regina, SK S4S 0A2, Canada 2010 //<br>DOI: 10.1080/16506070902980711	Does not utilize/ assess tool

Comparison of motivational interviewing with acceptance and commitment therapy: A conceptual and clinical review<br>Bricker, J; Tollison, S<br>Behavioural and Cognitive Psychotherapy // 2011;39(5):541-559<br>University of Washington, Fred Hutchinson Cancer Research Center, 1100 Fairview Avenue North, M3-B232, Seattle, WA 98195, United States 2011 //<br>DOI: 10.1017/S1352465810000901	Does not utilize/ assess tool
Chronic pain in children and young people<br>Goddard, J M<br>Current Opinion in Supportive and Palliative Care // 2011;5(2):158-163<br>Sheffield Children's Hospital, Western Bank, Sheffield, United Kingdom 2011 //<br>DOI: 10.1097/SPC.0b013e328345832d	Does not utilize/ assess tool
Acceptance and values-based treatment of adolescents with chronic pain: Outcomes and their relationship to acceptance<br>Gauntlett-Gilbert, J; Connell, H; Clinch, J; Mccracken, L M<br>Journal of Pediatric Psychology // 2013;38(1):72-81<br>2013 //<br>DOI: 10.1093/jpepsy/jss098	Does not utilize/ assess tool
Acceptance and Commitment Therapy (ACT) for Adolescents: Outcomes of a Large-Sample, School-Based, Cluster-Randomized Controlled Trial<br>Van der Gucht, K; Griffith, J W; Hellemans, R; Bockstaele, M; Pascal-Claes, F; Raes, F<br>Mindfulness // 2017;8(2):408-416<br>Faculty of Psychology and Educational Sciences, University of Leuven, Tiensestraat 102, Leuven, 3000, Belgium Springer New York LLC 2017 //<br>DOI: 10.1007/s12671-016-0612-y	Does not utilize/ assess tool
The research journey of acceptance and commitment therapy (ACT)<br>Hooper, N; Larsson, A<br>The Research Journey of Acceptance and Commitment Therapy (ACT) // 2015;():1-212<br>2015 //<br>DOI: 10.1057/9781137440174	Cannot locate full text
Clinical utility and validity of the Functional Disability Inventory among a multicenter sample of youth with chronic pain<br>Kashikar-Zuck, S; Flowers, S R; Claar, R L; Guite, J W; Logan, D E; Lynch-Jordan, A M; Palermo, T M; Wilson, A C	Does not utilize/ assess tool

Pain // 2011;152(7):1600-1607<br>Division of Behavioral Medicine and Clinical Psychology, Cincinnati Children's Hospital Medical Center, University of Cincinnati College of Medicine, Cincinnati, OH, United States 2011 //<br>DOI: 10.1016/j.pain.2011.02.050	
Psychological therapy of migraine: Systematic review<br>Fritsche, G; Kröner-Herwig, B; Kropp, P; Niederberger, U; Haag, G<br>Schmerz // 2013;27(3):263-274<br>Neurologische Klinik, Universität Essen, Hufelandstr. 55, 45122 Essen, Germany 2013 //<br>DOI: 10.1007/s00482-013-1319-9	Does not utilize/ assess tool
Cognitive Behavioral Therapy increases pain-evoked activation of the prefrontal cortex in patients with fibromyalgeia<br>Jensen, K B; Kosek, E; Wicksell, R; Kemani, M; Olsson, G; Merle, J V; Kadetoff, D; Ingvar, M<br>Pain // 2012;153(7):1495-1503<br>Department of Psychiatry, Massachusetts General Hospital, Harvard Medical School, Boston, MA, United States 2012 //<br>DOI: 10.1016/j.pain.2012.04.010	Does not utilize/ assess tool
Photographs of Daily Activities-Youth English: Validating a targeted assessment of worry and anticipated pain<br>Simons, L E; Pielech, M; McAvoy, S; Conroy, C; Hogan, M; Verbunt, J A; Goossens, M E<br>Pain // 2017;158(5):912-921<br>Department of Anesthesiology, Perioperative and Pain Medicine, Stanford University School of Medicine, 1070 Arastradero Rd, Palo Alto, CA 94304, United States Lippincott Williams and Wilkins 2017 //<br>DOI: 10.1097/j.pain.0000000000000855	Does not utilize/ assess tool
The Psychological Inflexibility in Pain Scale (PIPS) - Statistical properties and model fit of an instrument to assess change processes in pain related disability<br>Wicksell, R K; Lekander, M; Sorjonen, K; Olsson, G L<br>European Journal of Pain // 2010;14(7):771.e1-771.e14<br>2010 //<br>DOI: 10.1016/j.ejpain.2009.11.015	Does not utilize/ assess tool
Age moderates response to acceptance and commitment therapy vs. cognitive behavioral therapy for chronic pain<br>Wetherell, J L; Petkus, A J; Alonso-Fernandez, M; Bower, E S; Steiner, A R W; Afari, N<br>International Journal of Geriatric Psychiatry // 2016;31(3):302-308<br>VA San Diego Healthcare System, San Diego, CA, United States John Wiley and Sons Ltd 2016 //	Does not utilize/ assess tool

DOI: 10.1002/gps.4330	
Acceptance and Commitment Therapy for the Treatment of Posttraumatic Stress Among Adolescents<br>Woidneck, M R; Morrison, K L; Twohig, M P<br>Behavior Modification // 2014;38(4):451-476<br>Utah State University, Logan, United States SAGE Publications Inc. 2014 //<br>DOI: 10.1177/0145445513510527	Does not utilize/ assess tool
One-day behavioral treatment for patients with comorbid depression and migraine: A pilot study<br>Dindo, L; Recober, A; Marchman, J N; Turvey, C; O'Hara, M W<br>Behaviour Research and Therapy // 2012;50(9):537-543<br>University of Iowa College of Medicine, Department of Psychiatry, MEB 2-203, Iowa City, IA 52242, United States 2012 //<br>DOI: 10.1016/j.brat.2012.05.007	Does not utilize/ assess tool
A randomized, controlled trial of acceptance and commitment therapy and cognitive-behavioral therapy for chronic pain<br>Wetherell, J L; Afari, N; Rutledge, T; Sorrell, J T; Stoddard, J A; Petkus, A J; Solomon, B C; Lehman, D H; Liu, L; Lang, A J; Hampton Atkinson, J<br>Pain // 2011;152(9):2098-2107<br>VA San Diego Healthcare System, Dept. 111N-1, 3350 La Jolla Village Drive, San Diego, CA 92161, United States 2011 //<br>DOI: 10.1016/j.pain.2011.05.016	Does not utilize/ assess tool
Selumetinib in children with inoperable plexiform neurofibromas<br>Gross, A M; Wolters, P L; Dombi, E; Baldwin, A; Whitcomb, P; Fisher, M J; Weiss, B; Kim, A; Bornhorst, M; Shah, A C; Martin, S; Roderick, M C; Pichard, D C; Carbonell, A; Paul, S M; Therrien, J; Kapustina, O; Heisey, K; Wade Clapp, D; Zhang, C; Peer, C J; Figg, W D; Smith, M; Glod, J; Blakeley, J O; Steinberg, S M; Venzon, D J; Austin Doyle, L; Widemann, B C<br>New England Journal of Medicine // 2020;382(15):1430-1442<br>Pediatric Oncology BranchMD, United States Massachussetts Medical Society 2020 //<br>DOI: 10.1056/NEJMoa1912735	Not exclusive chronic pain population
Departing from the essential features of a high quality systematic review of psychotherapy: A response to Öst (2014) and recommendations for improvement<br>Atkins, P W B; Ciarrochi, J; Gaudiano, B A; Bricker, J B; Donald, J; Rovner, G; Smout, M; Livheim, F; Lundgren, T; Hayes, S C<br>Behaviour Research and Therapy // 2017;97():259-272<br>Institute for Positive Psychology and Education, Australian Catholic University, Strathfield, NSW 2135, Australia Elsevier Ltd 2017 //<br>DOI: 10.1016/j.brat.2017.05.016	Does not utilize/ assess tool

Resilience factors in children with juvenile idiopathic arthritis and their parents: The role of child and parent psychological flexibility<br>Beeckman, M; Hughes, S; Van Ryckeghem, D; Van Hoecke, E; Dehoorne, J; Joos, R; Goubert, L<br>Pain Medicine (United States) // 2019;20(6):1120-1131<br>Department of Experimental, Clinical and Health Psychology, Ghent University, H. Dunantlaan 2, Ghent, 9000, Belgium Oxford University Press 2019 //<br>DOI: 10.1093/pm/pny181	Does not utilize/ assess tool
Psychological Interventions for Children with Functional Somatic Symptoms: A Systematic Review and Meta-Analysis<br>Bonvanie, I J; Kallesøe, K H; Janssens, K A M; Schröder, A; Rosmalen, J G M; Rask, C U<br>Journal of Pediatrics // 2017;187():272-281.e17<br>University Medical Center of Groningen, Interdisciplinary Center Psychopathology and Emotion Regulation, University of Groningen, Groningen, Netherlands Mosby Inc. 2017 //<br>DOI: 10.1016/j.jpeds.2017.03.017	Does not utilize/ assess tool
Acceptance of premonitory urges and tics<br>Gev, E; Pilowsky-Peleg, T; Fennig, S; Benaroya-Milshtein, N; Woods, D W; Piacentini, J; Apter, A; Steinberg, T<br>Journal of Obsessive-Compulsive and Related Disorders // 2016;10():78-83<br>Matta and Harry Freund Neuropsychiatry Tourette Syndrome and Tic Disorders Clinic, Schneider Children's Medical Center of Israel, Petach Tikva, 49202, Israel Elsevier B.V. 2016 //<br>DOI: 10.1016/j.jocrd.2016.06.001	Does not utilize/ assess tool
Acceptance and Commitment Therapy versus Cognitive Behavior Therapy for Children With Anxiety: Outcomes of a Randomized Controlled Trial<br>Hancock, K M; Swain, J; Hainsworth, C J; Dixon, A L; Koo, S; Munro, K<br>Journal of Clinical Child and Adolescent Psychology // 2018;47(2):296-311<br>Department of Psychological Medicine, The Children’s Hospital at Westmead, Australia Routledge 2018 //<br>DOI: 10.1080/15374416.2015.1110822	Does not utilize/ assess tool
Acceptance and Commitment Therapy (ACT): Advances and Applications with Children, Adolescents, and Families<br>Coyne, L W; McHugh, L; Martinez, E R<br>Child and Adolescent Psychiatric Clinics of North America // 2011;20(2):379-399<br>Psychology Department, Early Childhood Research Clinic, Suffolk University, 41 Temple Street, Boston, MA 02114, United States 2011 //<br>DOI: 10.1016/j.chc.2011.01.010	Does not utilize/ assess tool

Risk severity moderated effectiveness of pain treatment in adolescents<br>Vuorimaa, H; Leppänen, L; Kautiainen, H; Mikkelsson, M; Hietanen, M; Vilen, H; Pohjankoski, H<br>Scandinavian Journal of Pain // 2019;19(2):287-298<br>Department of Pediatrics, Päijät-Häme Central Hospital, Keskussairaalankatu 7, Lahti, 15850, Finland De Gruyter 2019 //<br>DOI: 10.1515/sjpain-2018-0312	Does not utilize/ assess tool
Anxiety, coping, and disability: A test of mediation in a pediatric chronic pain sample<br>Kaczynski, K J; Simons, L E; Claar, R L<br>Journal of Pediatric Psychology // 2011;36(8):932-941<br>Pain Treatment Service, Children's Hospital, Boston, 300 Longwood Ave, Boston, MA 02115, United States 2011 //<br>DOI: 10.1093/jpepsy/jsr024	Does not utilize/ assess tool
Physician Complicity in the Transformation of Pain Medicine from a "Profession" to a "Business": Strategies for Reversing a Growing Trend<br>Schatman, M E<br>Pain Medicine (United States) // 2012;13(9):1149-1151<br>Foundation for Ethics in Pain Care, Bellevue, WA, United States Blackwell Publishing Inc. 2012 //<br>DOI: 10.1111/j.1526-4637.2012.01464.x	Does not utilize/ assess tool
The efficacy of group-based acceptance and commitment therapy on psychological capital and school engagement: A pilot study among Chinese adolescents<br>Fang, S; Ding, D<br>Journal of Contextual Behavioral Science // 2020;16():134-143<br>Department of Psychology, Anhui Normal University, Wuhu, China Elsevier Inc. 2020 //<br>DOI: 10.1016/j.jcbs.2020.04.005	Does not utilize/ assess tool
Lessons learned from a mindfulness-based intervention with chronically ill youth<br>Lagor, A F; Williams, D J; Lerner, J B; McClure, K S<br>Clinical Practice in Pediatric Psychology // 2013;1(2):146-158<br>Department of Psychology, La Salle University, 1900 West Olney Avenue, Box 275, Philadelphia, PA 19141, United States American Psychological Association Inc. 2013 //<br>DOI: 10.1037/cpp0000015	Does not utilize/ assess tool
Pharmacodynamic Study of Miransertib in Individuals with Proteus Syndrome<br>Keppler-Noreuil, K M; Sapp, J C; Lindhurst, M J; Darling, T N; Burton-Akright, J; Bagheri, M; Dombi, E; Gruber, A; Jarosinski, P F; Martin, S; Nathan, N; Paul, S M; Savage, R E; Wolters, P L; Schwartz, B; Widemann, B C; Biesecker, L G<br>American Journal of Human Genetics // 2019;104(3):484-491	Not pediatric population

Medical Genomics and Metabolic Genetics Branch, National Human Genome Research Institute, NIH, Bethesda, MD 20892, United States Cell Press 2019 //<br>DOI: 10.1016/j.ajhg.2019.01.015	
Acceptance and Commitment Therapy for children and adolescents with physical health concerns<br>Wicksell, R K; Kanstrup, M; Kemani, M K; Holmström, L; Olsson, G L<br>Current Opinion in Psychology // 2015;2():1-5<br>Behavior Medicine Pain Treatment Service, Karolinska University Hospital, Sweden Elsevier 2015 //<br>DOI: 10.1016/j.copsyc.2014.12.029	Does not utilize/ assess tool
Improving the mental health of adolescents with epilepsy through a group cognitive behavioral therapy program<br>Carbone, L; Plegue, M; Barnes, A; Shellhaas, R<br>Epilepsy and Behavior // 2014;39():1-1<br>Department of Social Work, University of Michigan Health System, Ann Arbor, MI, United States Academic Press Inc. 2014 //<br>DOI: 10.1016/j.yebeh.2014.07.024	Does not utilize/ assess tool
Coping and acceptance in chronic childhood conditions<br>Gauntlett-Gilbert, J; Connell, H<br>Psychologist // 2012;25(3):198-201<br>Bath Centre for Pain Services, University of Bath, United Kingdom 2012 //	Does not utilize/ assess tool
Physical therapy and functional rehabilitation<br>Jaremko, K; Hsu, B<br>Complex Regional Pain Syndrome: Past, Present and Future // 2015;():145-193<br>Jefferson Medical College, Philadelphia, PA, United States Nova Science Publishers, Inc. 2015 //	Cannot locate full text
Practice-based evidence for outpatient, acceptance & commitment therapy for veterans with chronic, non-cancer pain<br>Cosio, D<br>Journal of Contextual Behavioral Science // 2016;5(1):23-32<br>Jesse Brown VA Medical Center, Anesthesiology/Pain Clinic, #124, Chicago, IL 60612, United States Elsevier Inc. 2016 //<br>DOI: 10.1016/j.jcbs.2015.12.002	Does not utilize/ assess tool
Insomnia in paediatric chronic pain and its impact on depression and functional disability<br>Kanstrup, M; Holmström, L; Ringström, R; Wicksell, R K<br>European Journal of Pain (United Kingdom) // 2014;18(8):1094-1102<br>Behavior Medicine Pain Treatment Services, Karolinska University Hospital, Solna, Sweden Blackwell Publishing Ltd 2014 //<br>DOI: 10.1002/j.1532-2149.2013.00450.x	Does not utilize/ assess tool

The role of parent psychological flexibility in relation to adolescent chronic pain: Further instrument development<br>Wallace, D P; McCracken, L M; Weiss, K E; Harbeck-Weber, C<br>Journal of Pain // 2015;16(3):235-246<br>Pain Management, Children's Mercy Kansas City, 2401 Gillham Road, Kansas City, MO 64108, United States Churchill Livingstone Inc. 2015 //<br>DOI: 10.1016/j.jpain.2014.11.013	Does not utilize/ assess tool
Fear of pain in children and adolescents with neuropathic pain and complex regional pain syndrome<br>Simons, L E<br>Pain // 2016;157():S90-S97<br>Division of Pain Medicine, Department of Anesthesiology, Perioperative and Pain Medicine, Boston Children's Hospital, 21 Autumn St, Boston, MA 02215, United States Lippincott Williams and Wilkins 2016 //<br>DOI: 10.1097/j.pain.0000000000000377	Does not utilize/ assess tool
Behavior Therapy: The Second and Third Waves<br>Ferguson, K E; O'Donohue, W<br>International Encyclopedia of the Social & Behavioral Sciences: Second Edition // 2015;():431-436<br>St. Peter Family Medicine Residency ProgramWA, United States Elsevier Inc. 2015 //<br>DOI: 10.1016/B978-0-08-097086-8.21090-8	Not an original study
A Functional Contextualist Approach to Cultural Evolution: An Introduction to Part IV<br>Biglan, A<br>The Wiley Handbook of Contextual Behavioral Science // 2015;():383-397<br>Oregon Research Institute, United States Wiley Blackwell 2015 //<br>DOI: 10.1002/9781118489857.ch19	Does not utilize/ assess tool
Acceptance- and mindfulness-based interventions for the treatment of chronic pain: a meta-analytic review<br>Veehof, M M; Trompetter, H R; Bohlmeijer, E T; Schreurs, K M G<br>Cognitive Behaviour Therapy // 2016;45(1):5-31<br>Department of Psychology, Health & Technology, University of Twente, Enschede, Netherlands Routledge 2016 //<br>DOI: 10.1080/16506073.2015.1098724	Does not utilize/ assess tool
Nonpharmacologic Treatment of Pain<br>Agoston, A M; Sieberg, C B<br>Seminars in Pediatric Neurology // 2016;23(3):220-223<br>Department of Psychiatry, Boston Children's Hospital, Boston, MA, United States W.B. Saunders 2016 //	Does not utilize/ assess tool

DOI: 10.1016/j.spen.2016.10.005	
Psychological treatments for pediatric headache<br>Krner-Herwig, B<br>Expert Review of Neurotherapeutics // 2011;11(3):403-410<br>Department of Clinical Psychology and Psychotherapy, Georg-Elias-Müller Institute of Psychology, Georg-August-University of Gttingen, Gosslerstr., 1437073 Gttingen, Germany 2011 //<br>DOI: 10.1586/ern.11.10	Does not utilize/ assess tool
Systematic review: Issues in measuring clinically meaningful change in self-reported chronic pediatric pain intensity<br>Lavigne, J V<br>Journal of Pediatric Psychology // 2016;41(7):715-734<br>Department of Child and Adolescent Psychiatry, Ann and Robert H. Lurie Children's Hospital of Chicago, 225 East Chicago Avenue, Chicago, IL 60611, United States Oxford University Press 2016 //<br>DOI: 10.1093/jpepsy/jsv161	Does not utilize/ assess tool
Collaborating on evolving the future<br>Wilson, D S; Hayes, S C; Biglan, A; Embry, D D<br>Behavioral and Brain Sciences // 2014;37(4):438-460<br>Departments of Biology and Anthropology, SUNY Binghamton, Binghamton, NY 13903, United States Cambridge University Press 2014 //<br>DOI: 10.1017/S0140525X14000016	Does not utilize/ assess tool
Implementing an acceptance and commitment therapy group protocol with veterans using VA’s stepped care model of pain management<br>Cosio, D; Schafer, T<br>Journal of Behavioral Medicine // 2015;38(6):984-997<br>Anesthesiology/Pain Clinic #124, Jesse Brown VA Medical Center, Chicago, IL 60612, United States Springer New York LLC 2015 //<br>DOI: 10.1007/s10865-015-9647-0	Does not utilize/ assess tool
Coaching of patients with an isolated minimally displaced fracture of the radial head immediately increases range of motion<br>Teunis, T; Thornton, E R; Guitton, T G; Vranceanu, A.-M.; Ring, D<br>Journal of Hand Therapy // 2016;29(3):314-319<br>Orthopaedic Hand and Upper Extremity Service, Massachusetts General Hospital, Harvard Medical School, Boston, MA, United States Hanley and Belfus Inc. 2016 //	Does not utilize/ assess tool

DOI: 10.1016/j.jht.2016.02.003	
Psychological treatments and psychotherapies in the neurorehabilitation of pain: Evidences and recommendations from the italian consensus conference on pain in neurorehabilitation<br>Castelnuovo, G; Giusti, E M; Manzoni, G M; Saviola, D; Gatti, A; Gabrielli, S; Lacerenza, M; Pietrabissa, G; Cattivelli, R; Spatola, C A M; Corti, S; Novelli, M; Villa, V; Cottini, A; Lai, C; Pagnini, F; Castelli, L; Tavola, M; Torta, R; Arreghini, M; Zanini, L; Brunani, A; Capodaglio, P; D'Aniello, G E; Scarpina, F; Brioschi, A; Priano, L; Mauro, A; Riva, G; Repetto, C; Regalia, C; Molinari, E; Notaro, P; Paolucci, S; Sandrini, G; Simpson, S G; Wiederhold, B; Tamburin, S; Agostini, M; Alfonsi, E; Aloisi, A M; Alvisi, E; Aprile, I; Armando, M; Avenali, M; Azicnuda, E; Barale, F; Bartolo, M; Bergamaschi, R; Berlangieri, M; Berlincioni, V; Berliocchi, L; Berra, E; Berto, G; Bonadiman, S; Bonazza, S; Bressi, F; Brugnera, A; Brunelli, S; Buzzi, M G; Cacciatori, C; Calvo, A; Cantarella, C; Caraceni, A; Carone, R; Carraro, E; Casale, R; Castellazzi, P; Castino, A; Cerbo, R; Chiò, A; Ciotti, C; Cisari, C; Coraci, D; Toffola, E D; Defazio, G; De Icco, R; Del Carro, U; Dell'Isola, A; De Tanti, A; D'Ippolito, M; Fazzi, E; Ferrari, A; Ferrari, S; Ferraro, F; Formaglio, F; Formisano, R; Franzoni, S; Gajofatto, F; Gandolfi, M; Gardella, B; Geppetti, P; Giammò, A; Gimigliano, R; Greco, E; Ieraci, V; Invernizzi, M; Jacopetti, M; La Cesa, S; Lobba, D; Magrinelli, F; Mandrini, S; Manera, U; Marchettini, P; Marchioni, E; Mariotto, S; Martinuzzi, A; Masciullo, M; Mezzarobba, S; Miotti, D; Modenese, A; Molinari, M; Monaco, S; Morone, G; Nappi, R; Negrini, S; Pace, A; Padua, L; Pagliano, E; Palmerini, V; Pazzaglia, C; Pecchioli, C; Picelli, A; Porro, C A; Porru, D; Romano, M; Roncari, L; Rosa, R; Saccavini, M; Sacerdote, P; Schenone, A; Schweiger, V; Scivoletto, G; Smania, N; Solaro, C; Spallone, V; Springhetti, I; Tassorelli, C; Tinazzi, M; Togni, R; Torre, M; Traballesi, M; Tramontano, M; Truini, A; Tugnoli, V; Turolla, A; Vallies, G; Verzini, E; Vottero, M; Zerbinati, P; Neurorehabilitation, Italian Consensus Conference on Pain in<br>Frontiers in Psychology // 2016;7(FEB):<br>Psychology Research Laboratory, Istituto Auxologico Italiano IRCCS, San Giuseppe Hospital, Verbania, Italy Frontiers Media S.A. 2016 //<br>DOI: 10.3389/fpsyg.2016.00115	Does not utilize/ assess tool

Tool 6 Pain Related Problems List for Adolescents

Evaluation of Psychometric and Linguistic Properties of the Italian Adolescent Pain Assessment Scales: A Systematic Review<br>Marti, Flavio; Paladini, Antonella; Varrassi, Giustino; Latina, Roberto<br>PAIN AND THERAPY 06// 2018;7(1):77-104<br>GEWERBESTRASSE 11, CHAM, CH-6330, SWITZERLAND SPRINGER INTERNATIONAL PUBLISHING AG 2018 06//<br>DOI: 10.1007/s40122-018-0093-x	Not an original study
Chronic pain in adolescents: Psychosocial consequences, predictors and intervention<br>Hunfeld, J A M; Merlijn, V.P.B.M.<br>Tijdschrift voor Kindergeneeskunde // 2008;76(4):172-179<br>Afdeling Medische Psychologie en Psychotherapie, Erasmus MC, Rotterdam, Netherlands Bohn Stafleu van Loghum 2008 //	Cannot locate full text

DOI: 10.1007/BF03078200	
Early signaling, referral, and treatment of adolescent chronic pain: A study protocol<br>Voerman, J S; Remerie, S; de Graaf, L E; van de Looij-Jansen, P; Westendorp, T; van Elderen, I; de Waart, F; Passchier, J; van Berkel, A D; de Klerk, C<br>BMC Pediatrics // 2012;12():<br>Department of Medical Psychology and Psychotherapy, Erasmus MC University Medical Hospital, PO Box 2040, Rotterdam, CA, 3000, Netherlands 2012 //<br>DOI: 10.1186/1471-2431-12-66	Does not utilize/ assess tool
Effects of a Guided Internet-Delivered Self-Help Intervention for Adolescents with Chronic Pain<br>Voerman, J S; Remerie, S; Westendorp, T; Timman, R; Busschbach, J J V; Passchier, J; De Klerk, C<br>Journal of Pain // 2015;16(11):1115-1126<br>Department of Psychiatry, Section Medical Psychology and Psychotherapy, Erasmus MC, University Medical Centre, P.O. Box 2040, Rotterdam, 3000 CA, Netherlands Churchill Livingstone Inc. 2015 //<br>DOI: 10.1016/j.jpain.2015.07.011	Does not utilize/ assess tool
Measuring Pain in Adolescents<br>Ameringer, S<br>Journal of Pediatric Health Care // 2009;23(3):201-204<br>2009 //<br>DOI: 10.1016/j.pedhc.2009.01.006	Not an original study
